# Modular three-component synthesis, photophysics, and electronic structure of solid-state and aggregate luminescent α-sulfenylated aroyl-*S*,*N*-ketene acetals†

**DOI:** 10.1039/d6ra04851j

**Published:** 2026-08-12

**Authors:** Julius Krenzer, Paul London, Sarah Merzenich, Vera Vasylyeva, Thomas J. J. Müller

**Affiliations:** a Institut für Organische Chemie und Makromolekulare Chemie, Heinrich-Heine-Universität Düsseldorf Universitätsstraße 1 D-40225 Düsseldorf Germany ThomasJJ.Mueller@uni-duesseldorf.de; b Abteilung für Crystal Engineering, Institut für Anorganische Chemie und Strukturchemie, Heinrich-Heine-Universität Düsseldorf Universitätsstrasse 1 D-40225 Düsseldorf Germany

## Abstract

α-Sulfenylated aroyl-*S*,*N*-ketene acetals are accessible from acyl chlorides, benzothiazolium salts, and *N*-thiosuccinimides *via* consecutive one-pot three-component synthesis in a modular fashion. The resulting merocyanines are prone to equilibrating *E*–*Z*-isomerization in solution, whereas preferred configurations can be found in the crystal structures. TD-DFT calculations indicate that the longest wavelength absorption bands of the chromophores in solution can be rationalized as HOMO–LUMO charge-transfer transitions of the energetically favored *E*-configurations. The α-sulfenylated aroyl-*S*,*N*-ketene acetals exhibit pronounced solid-state emission, with emission colors ranging from blue to orange and emission upon induced aggregation is observed in acetonitrile/water mixtures with significant quantum yields at water contents of 80%.

## Abstract 2

A modular one-pot three-component synthesis affords a-sulfenylated aroyl-*S*,*N*-ketene acetals. These merocyanines show *E*/*Z*-isomerization, charge-transfer absorption, and strong solid-state and aggregation-induced emission from blue to orange.

## Graphical abstract

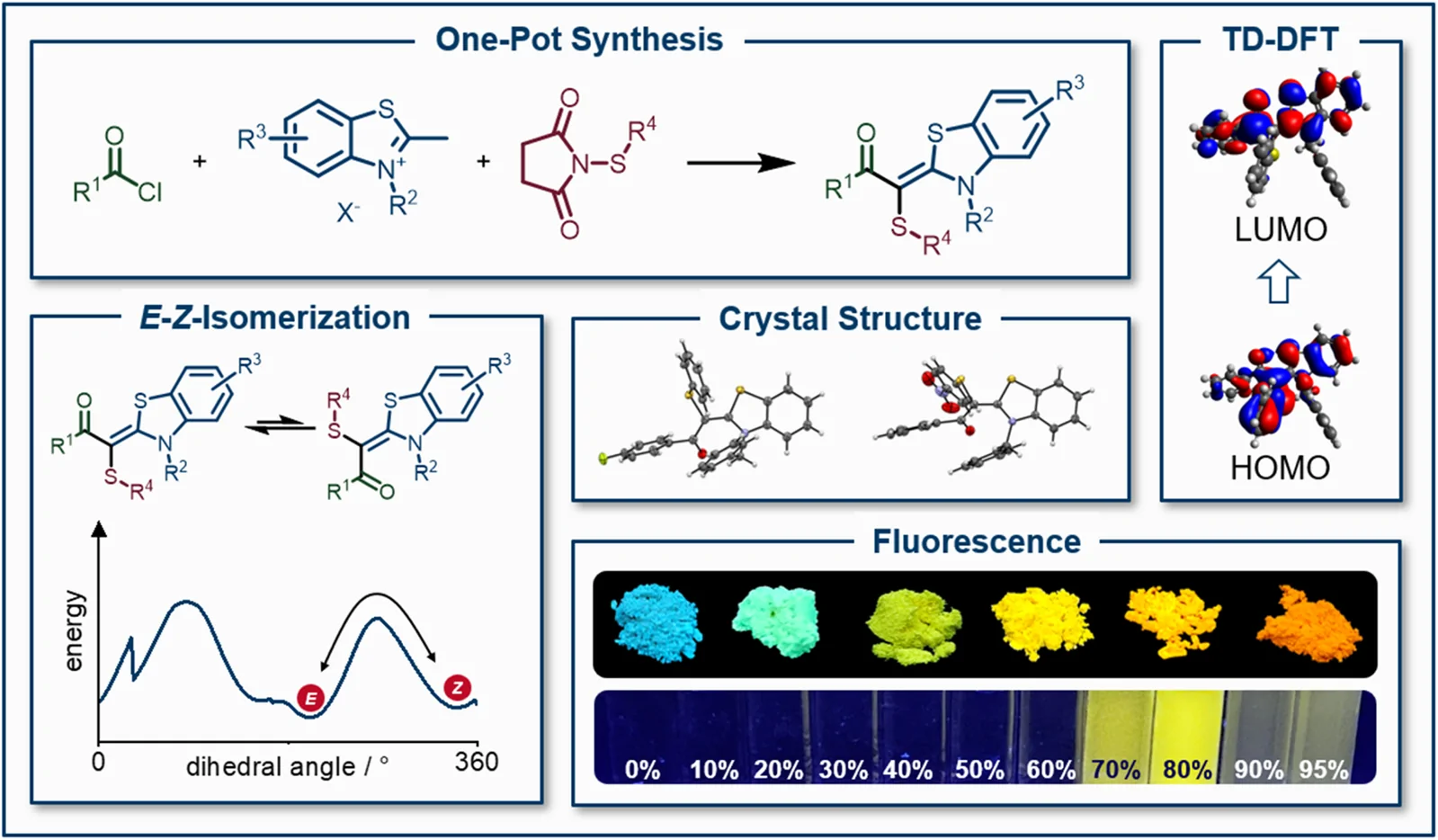

## Introduction

Functional organic dyes are key components in modern materials science, with applications in optoelectronics, sensing, and imaging.^1,2^ Their structural diversity enables precise tuning of photophysical properties; however, many conventional fluorophores suffer from aggregation-caused quenching (ACQ) in the solid state.^3,4^ In contrast, aggregation-induced emission (AIE) relies on luminogens that are weakly emissive in solution but become highly luminescent upon induced aggregation, offering a powerful strategy for the design of solid-state emitters.^5–7^

Aroyl-*S*,*N*-ketene acetals, developed extensively by our group, have emerged as a versatile class of AIEgens.^8^ These heterocyclic merocyanines feature pronounced push–pull electronic structures and are readily accessible *via* a modular, base-mediated condensation reaction.^9^ They exhibit tunable solid-state emission as well as pronounced AIE characteristics.^10^ Structural diversification, including alkynylated, triazole-linked, and multichromophoric derivatives have set the stage for fine control of emission properties and aggregation behavior.^11–15^

Despite these advances, further functionalization of the aroyl-*S*,*N*-ketene acetal scaffold remains an important objective. The enhanced nucleophilicity of the α-carbonyl position, *i.e.* the α-position of the *S*,*N*-ketene acetal moiety, has already been utilized to modify the aroyl-*S*,*N*-ketene acetals by acylation with a second acyl chloride,^16^ still offers potential for further modifications and for fine-tuning of the inherent photophysical properties. Herein, we report sulfenylated aroyl-*S*,*N*-ketene acetals which are readily accessible by electrophilic α-sulfenylation in a consecutive one-pot-fashion, as a new class of functional dyes, expanding the structural scope of this chromophore and providing new insights into structure–emission relationships.

## Results and discussion

### Synthesis

**Scheme 1:**
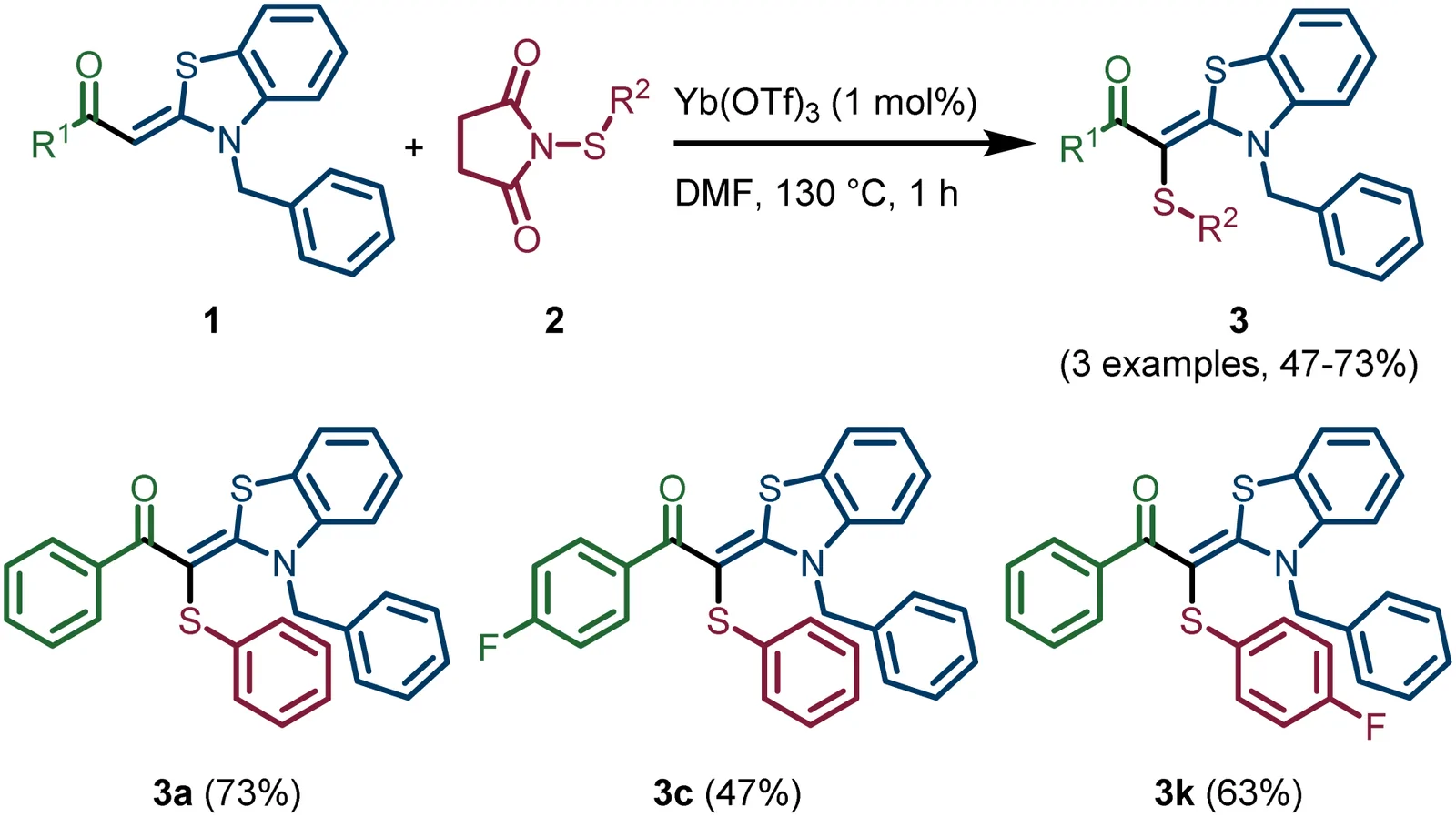
Synthesis of the α-sulfenylated aroyl-*S*,*N*-ketene acetals 3 in a single step reaction.

In the literature, a wide variety of methods for α-sulfenylation of enaminones with different sulfur-containing reagents are described, including disulfides,^17^ thiols,^18^ sulfenyl chlorides,^19^ and *N*-thiosuccinimides.^20^ Especially *N*-thiosuccinimides are versatile and highly selective electrophilic sulfenylation reagents. Their compatibility with multicomponent reactions renders them particularly valuable in organic synthesis.^21^ Since aroyl-*S*,*N*-ketene acetals bear a highly nucleophilic enaminone moiety, α-sulfenylation of these merocyanines with *N*-thiosuccinimides based upon previously reported conditions^21^ can be envisioned. In *N*-thiosuccinimides, the sulfur atom is electrophilic. Coordination of the Lewis acid catalyst to the carbonyl groups of the succinimide moiety and of the aroyl-*S*,*N*-ketene acetal enhances the polarization of the N–S bond of the former (*N*-thiosuccinimide) and the reactivity of the latter (aroyl-*S*,*N*-ketene acetal). Subsequent nucleophilic attack of the aroyl *S*,*N*-ketene acetal at the activated sulfur center is followed by deprotonation to afford the α-sulfenylated products. Aroyl-*S*,*N*-ketene acetals 1 were reacted with *N-*thiosuccinimides 2 under Lewis-acid catalysis at a temperature of 130 °C for 1 h, three examples of the α-sulfenylated aroyl-*S*,*N*-ketene acetals 3 were obtained after purification in moderate to good yields (Scheme 1).

This Lewis-acid catalyzed α-sulfenylation encouraged us to embed into a consecutive three-component synthesis of α-sulfenylated aroyl-*S*,*N*-ketene acetals 3 starting from acyl chlorides 4 and 2-methylbenzothiazolium salts 5. For optimization of the reaction conditions, the synthesis of the 4-fluorothiophenyl-substituted derivative 3k was employed as a model reaction, and the reaction progress was monitored by ^19^F NMR spectroscopy. First, a suitable solvent for the formation of the aroyl-*S*,*N*-ketene acetals 1 and the subsequent sulfenylation has to be identified. While only very limited product formation was observed in DMF, dichloromethane, or acetonitrile, 1,4-dioxane proved to be a viable solvent for both transformations (Table 1). In the first step, the optimized conditions for the synthesis of aroyl-*S*,*N*-ketene acetals can be applied.^9^ Subsequently, an equimolar amount of *N*-thiosuccinimide and 1 mol% ytterbium(iii) triflate are added, and stirring the reaction mixture at 120 °C for 2 h gives rise to an excellent ^19^F NMR spectroscopic yield of 96% and an isolated yield of 79%.

**Table 1:** Influence of different reaction conditions on the formation of α-sulfenylated aroyl-*S*,*N*-ketene acetal 3k

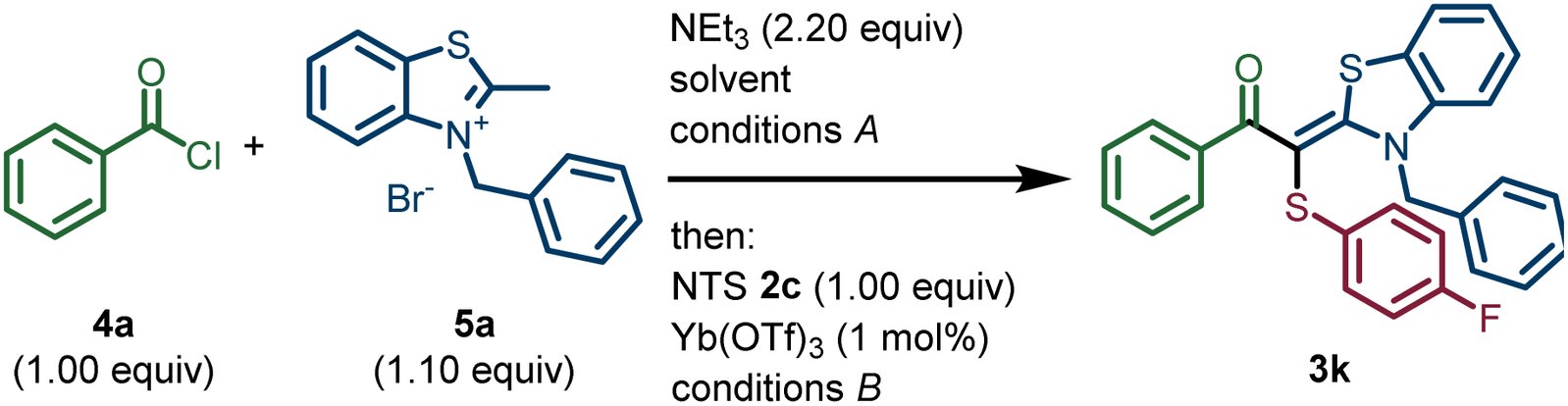				
Entry	Solvent	A	B	Yield^a^
1	DMF	rt, 1 h	120 °C, 2 h	28%
2	DCM	rt, 1 h	120 °C, 2 h	2%
3	Acetonitrile	rt, 1 h	120 °C, 2 h	4%
**4**	**1,4-Dioxane**	**rt, 1 h**	**120 °C, 2 h**	**96%**
5^b^	1,4-Dioxane	rt, 1 h	120 °C, 2 h	89%
6^c^	1,4-Dioxane	rt, 1 h	120 °C, 2 h	86%
7^d^	1,4-Dioxane	—	Rt, 1 h	28%
			Then: 120 °C, 2 h	
8^d^	1,4-Dioxane	—	120 °C, 3 h	39%

a Determined by ^19^F NMR spectroscopy.

b Addition of 0.3 equiv. of acetic acid in the second step.

c Addition of 1 mol% of AlCl_3_ instead of Yb(OTf)_3_ in the second step.

d Performed in a domino fashion with all reactants and catalyst present from the outset of the process.

**Scheme 2:**
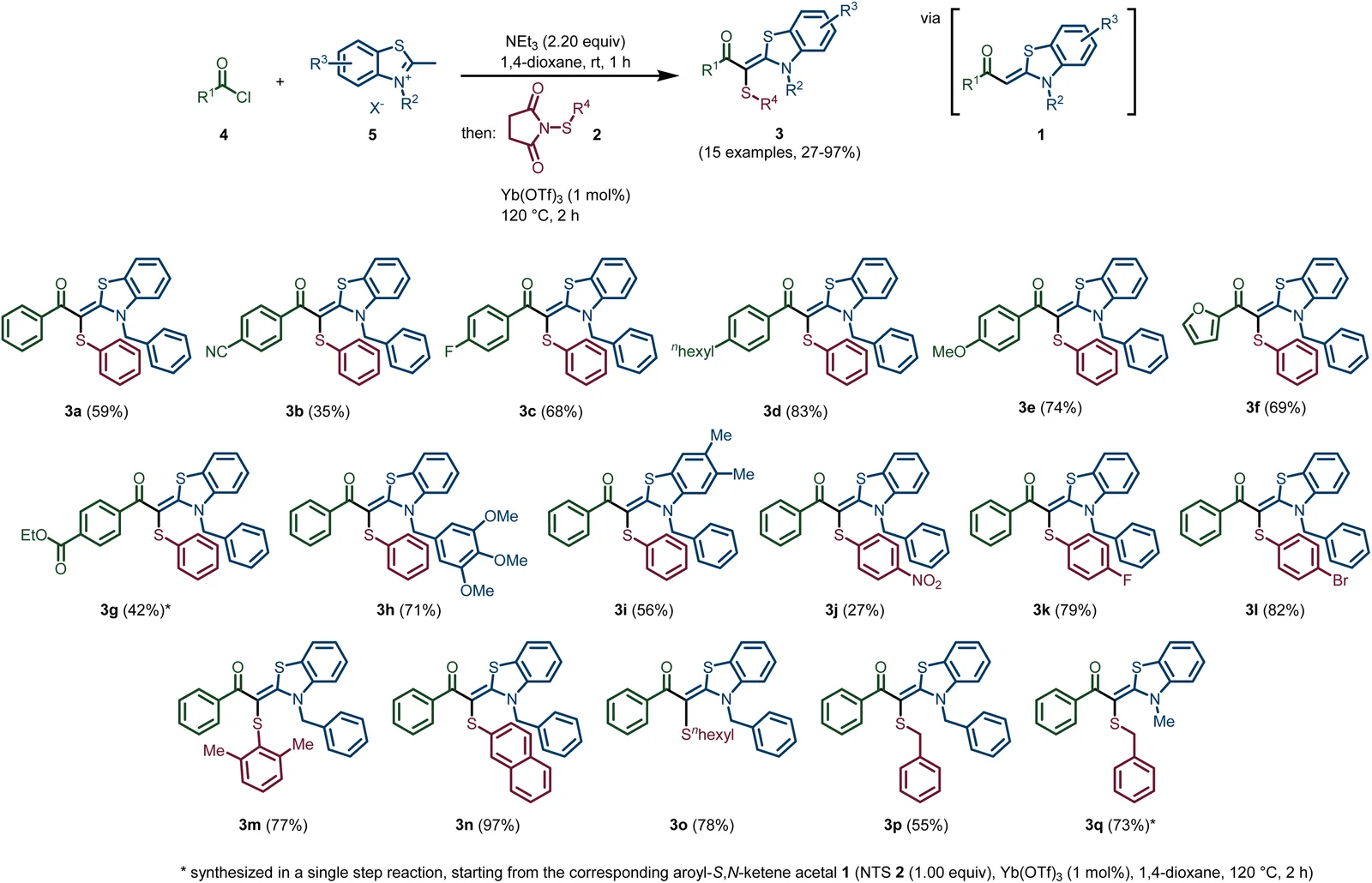
Consecutive three-component condensation–sulfenylation synthesis of α-sulfenylated aroyl-*S*,*N*-ketene acetals 3.

Furthermore, modifications, such as the addition of 0.3 equivalents of acetic acid to neutralize the excess of triethylamine (Table 1, entry 5), replacing of the Lewis acid ytterbium(iii) triflate with aluminum(iii) chloride (Table 1, entry 6) were tested. In addition, the presence of all reactants from the outset, *i.e.* in a domino fashion, was investigated, first upon stirring at room temperature for 1 h followed by heating at 120 °C for 2 h (Table 1, entry 7), and upon directly heating at 120 °C for 3 h (Table 1, entry 8). However, these latter conditions give rise to lower yields and side product formation. Therefore, condensation at room temperature for 1 h in 1,4-dioxane followed by α-sulfenylation at 120 °C for 2 h can be considered as optimal conditions for the consecutive three-component sequence (Table 1, entry 4), which then were adopted as the standard protocol for the synthesis of α-sulfenylated aroyl-*S*,*N*-ketene acetals 3 (Scheme 2).

The consecutive three-component condensation–sulfenylation protocol provides a concise, modular synthesis of 15 α-sulfenylated aroyl-*S*,*N*-ketene acetals 3 with yields after isolation ranging from 27 to 97%. The average yield per bond-forming step of this novel three-component synthesis can be estimated to 52–98%. While the isolated yield of the unsubstituted product 3a was higher under stepwise conditions, the one-pot protocol provided superior yields for derivatives 3c and 3k. The lowest yield is found for the electron-deficient 4-nitrothiophenyl derivative 3j, which can be attributed to the diminished reactivity of the corresponding *N*-thiosuccinimide 2b due to the strongly electron-withdrawing nitro group. Likewise, the intermediate in the synthesis of 3b bearing an electron-deficient benzoyl moiety exhibits diminished reactivity, resulting in a lower yield as a consequence of the decreased nucleophilicity of the corresponding aroyl-*S*,*N*-ketene acetal. All other examples can be isolated in good to excellent yields. With respect to the acyl chloride component 4, *para*-substituted benzoyl chlorides bearing both electron-donating and electron-withdrawing substituents (3a–e), as well as a 2-furoyl chloride (3f), can be successfully employed. In the sulfenylation step, both aromatic and aliphatic thioethers can be efficiently introduced (3j–p). Substitution at the benzothiazole core or at the *N*-benzyl substituent is well tolerated (3h and 3i), however, the *N*-methyl-substituted product 3q was not accessible through the one-pot protocol (3q). Since the photophysical properties of the ester-substituted derivative 3g and the *N*-methyl derivative 3q were of particular interest, both compounds were synthesized in two consecutive single-step reactions, involving the initial formation of the corresponding aroyl-*S*,*N*-ketene acetals 1 followed by α-sulfenylation.

### Structure

The structure of α-sulfenylated aroyl-*S*,*N*-ketene acetals 3 was unambiguously assigned by extensive NMR studies, mass spectrometry, and IR spectroscopy. In addition, the molecular composition of the new compounds was confirmed by combustion analysis or HRMS (HPLC). However, an unequivocal confirmation of the proposed *E*-configuration of the central double bond was not possible by these methods. Therefore, single-crystal X-ray diffraction analyses were performed on six selected derivatives.

**Fig. 1:**
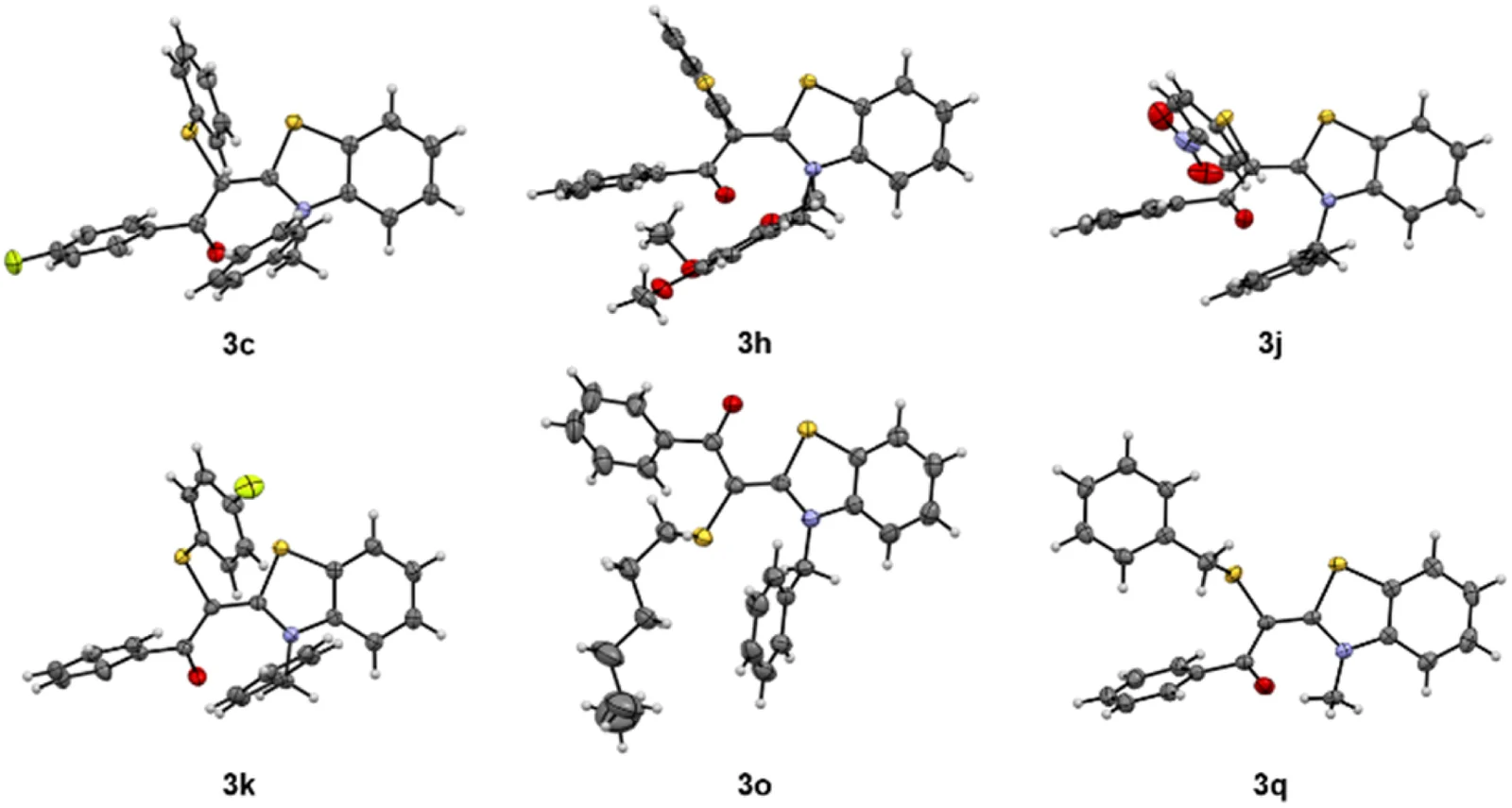
Molecular structures of α-sulfenylated aroyl-*S*,*N*-ketene acetals 3 (thermal ellipsoids are shown with 50% probability).

Contrary to expectations, five out of six crystal structures show a *Z*-configuration of the central double bond (Fig. 1). The only compound crystallizing in *E*-configuration is derivative 3o, which bears an *n*-hexylthio substituent. It is evident that the configuration has a pronounced influence on the planarity of the system. The *Z*-configured molecular structures show a strong torsion of the aromatic ring of the aroyl unit relative to the benzothiazole moiety with a torsion angle *α* between 75.4° and 86.6° (Table 2). This distortion can be attributed to steric interactions involving the sulfenyl substituents, which prohibit a planar arrangement of the aroyl moiety. Furthermore, the centrally located tetrasubstituted double bond likewise adopts a non-planar conformation. The torsional deformation of the double bond *β* amounts to 19.5–22.7°. The crystal structure of 3o, the only compound crystallizing in the *E*-configuration, exhibits a less pronounced distortion. The torsion angle *α* amounts to 37.6°, whereas the twist of the double bond *β* is reduced to 11.2°. The torsion reduces the conjugation within the chromophoric system, which is also reflected in an increased bond length alternation between the C–C (*d*_1_) single and C=C (*d*_2_) double bond connecting the aroyl and benzothiazole units. For the *Z*-configured crystal structures, the difference ranges from 0.051 to 0.095 Å, whereas for 3o a more pronounced bond length equalization is observed, with a difference of only 0.036 Å (Table 2). To validate that the observed torsion originates from steric effects rather than crystal-packing interactions, TD-DFT calculations were performed. The calculations were carried out using the program package Gaussian 16 (ref. 22) with the B3LYP/6-31G**^23,24^ functional and basis set and confirm the highly twisted structure (Table 2). The torsion angles *α* and *β* are found to be comparable in solution, and the more pronounced bond-length alternation in the *Z*-configuration is reproduced by the calculations as well.

**Table 2:** Comparison of bond length alternation (*d*_1_−*d*_2_) and torsion angles (*α* and *β*) based on crystal structure and TD-DFT data of α-sulfenylated aroyl-*S*,*N*-ketene acetals 3 (Gaussian 16, B3LYP/6-31G**, PCM acetonitrile, *T* = 298 K)

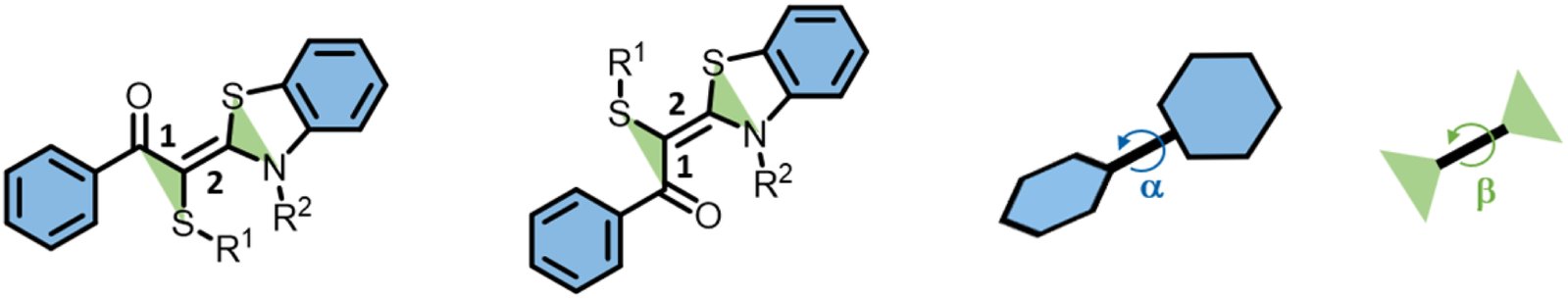						
Compound	Crystal			TD-DFT calculation		
	*d*_1_−*d*_2_	*α*	*β*	*d*_1_−*d*_2_	*α*	*β*
3c (*Z*)	0.054 Å	86.6°	22.7°	0.054 Å	79.4°	21.6°
3h (*Z*)	0.067 Å	78.2°	19.5°	0.065 Å	82.8°	20.1°
3j (*Z*)	0.051 Å	75.9°	20.7°	0.062 Å	82.7°	21.3°
3k (*Z*)	0.056 Å	80.5°	19.9°	0.065 Å	82.5°	20.3°
3o (*E*)	0.036 Å	37.6°	11.2°	0.043 Å	47.9°	16.0°
3q (*Z*)	0.095 Å	75.4°	21.7°	0.062 Å	80.2°	23.8°

**Scheme 3:**
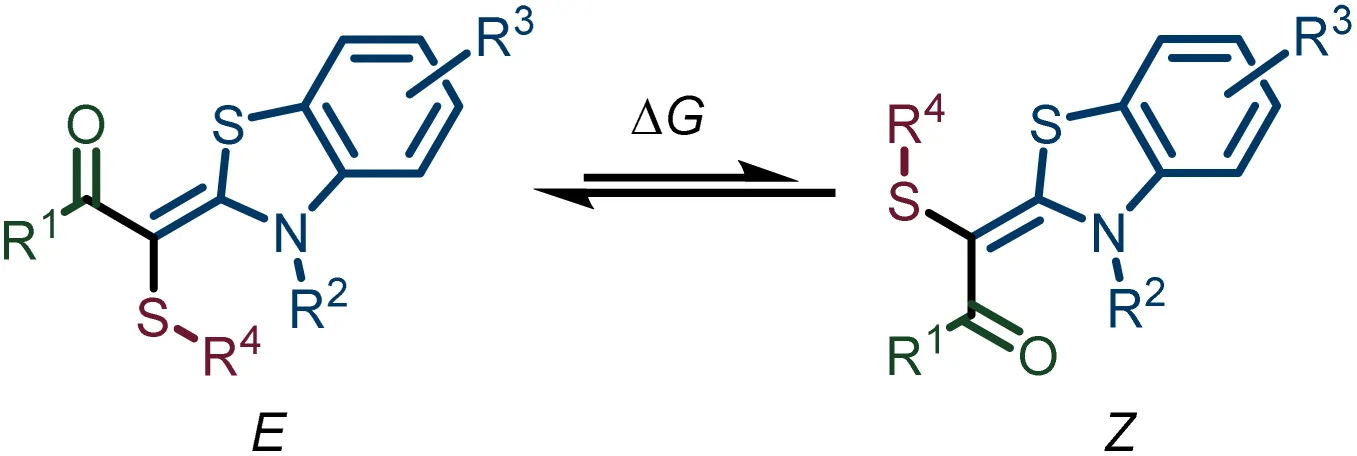
*E*–*Z* isomerization of compound 3c.

To elucidate whether the configuration is established during the synthesis and remains stable under ambient conditions or undergoes continuous isomerization in solution (Scheme 3), the rotational barrier of the C=C double bond was determined by a potential energy scan (Gaussian 16, B3LYP/6 31G**, PCM acetonitrile/ethanol). The optimized geometry of the chromophore in the *E*-configuration was employed as the initial structure. The potential energy scan of derivative 3c reveals a rotational barrier of 40.5 kJ mol^−1^ in ethanol and 40.1 kJ mol^−1^ in acetonitrile (see SI, Fig. S21). Based on the Eyring equation,^25^ the rate constant for the *E* → *Z* isomerization at room temperature was calculated to *k* = 4.9 × 10^5^ s^−1^ in ethanol and *k* = 5.8 × 10^5^ s^−1^ in acetonitrile. Thus, the rotational barrier is readily overcome at room temperature as seen by the appearance of a single set of signals, indicating rapid isomerization in solution. This hypothesis is further supported by low-temperature NMR spectroscopic study of compound 3a, where signal splitting into two distinct sets is observed below −30 °C (see SI, Fig. S2).

Subsequently, the calculated ground-state energies of all derivatives in both *E*- and *Z*-configuration were compared in order to identify the thermodynamically more stable isomer (Table 3). Assuming a Boltzmann distribution, the equilibrium constant *K* at room temperature can be derived from the difference in the computed Gibbs free enthalpies (Δ*G*).

**Table 3:** Comparison of the TD-DFT-calculated minimum structures of *E*- and *Z*-configured α-sulfenylated aroyl-*S*,*N*-ketene acetals 3 (Gaussian 16, B3LYP/6-31G**, PCM acetonitrile, *T* = 298 K)

Compound	More stable isomer	Δ*G* (*E*–*Z*)/kJ mol^−1^	*K* (*E*/*Z*)
3a	*E*	−7.69	22.3
3b	*E*	−8.55	31.5
3c	*E*	−2.29	2.46
3d	*E*	−6.47	13.6
3e	*E*	−6.40	13.2
3f	*Z*	1.73	0.498
3g	*E*	−11.3	96.0
3h	*Z*	5.72	0.100
3i	*E*	−7.90	24.2
3j	*E*	−8.69	33.3
3k	*E*	−7.57	21.2
3l	*E*	−9.33	43.1
3m	*E*	−3.40	3.95
3n	*E*	−6.19	12.2
3o	*E*	−10.2	62.0
3p	*E*	−3.23	3.68
3q	*Z*	1.47	0.553

The calculations indicate that, for most α-sulfenylated aroyl-*S*,*N*-ketene acetals 3, the *E*-configuration represents the energetic minimum. Exceptions are derivatives 3f, 3h and 3q, for which the *Z*-configuration is slightly favored. The observation that derivatives 3c, 3j, and 3k crystallize in the *Z-*configuration is most likely attributable to packing effects. As demonstrated above, rotation around the double bond is feasible, resulting in the presence of a certain fraction of *Z*-configured molecules in solution. Although this fraction is relatively small, approximately 3–5% according to the calculations for dyes 3j, and 3k, it appears to be sufficient for the crystallization process. For derivative 3o, this fraction is even lower, which may account for its crystallization in the *E*-configuration. In addition, the presence of an alkyl substituent gives rise to significantly different intermolecular interactions and packing effects.

### Photophysical properties

In solution α-sulfenylated aroyl-*S*,*N*-ketene acetals 3 show single broad absorption bands extending into the visible region, with absorption maxima in a narrow range from 371 to 394 nm (Table 4). Comparison of the absorption properties of chromophores 3 with those of the aroyl-*S*,*N*-ketene acetals 1, which have been extensively investigated in previous studies,^10,26^ reveals that the longest wavelength absorption maxima remain largely unaffected by the thioether substituent (see SI, Table S3). For instance, α-sulfenylated aroyl-*S*,*N*-ketene acetal 3a displays an absorption maximum at 371 nm, and the corresponding aroyl-*S*,*N*-ketene acetal exhibits a maximum at 376 nm. However, the molar extinction coefficient of dye 3a is significantly reduced (*ε* = 16 500 L mol^−1^ cm^−1^) compared to that of the unsubstituted dye 1a (*ε* = 39 100 L mol^−1^ cm^−1^). This decrease can be attributed to the torsion of the chromophoric system induced by incorporation of the thioether moiety. The resulting reduction in planarity of the π-system lowers the probability of photon absorption.

**Table 4:** Photophysical properties of all α-sulfenylated aroyl-*S*,*N*-ketene acetals 3 (absorption in acetonitrile: *T* = 298 K, *c* = 10^−5^ M; solid-state emission and absolute overall solid-state quantum yields: *T* = 298 K, *λ*_exc_ = *λ*_max,abs_)

Compound	R^1^	R^4^	*λ*_max,abs_/nm (*ε*/L mol^−1^ cm^−1^)	*λ*_max,em, solid state_/nm	*Φ*_em, solid state_
3a^a^	C_6_H_5_	C_6_H_5_	371 (16 500)	586	0.06
3b^a^	4-NC-C_6_H_4_	C_6_H_5_	373 (18 500)	567	0.07
3c^a^	4-F-C_6_H_4_	C_6_H_5_	373 (26 400)	582	0.07
3d^a^	4-^*n*^Hexyl-C_6_H_4_	C_6_H_5_	374 (28 400)	526	0.15
3e^a^	4-MeO-C_6_H_4_	C_6_H_5_	377 (25 800)	635	0.12
3f^a^	2-Furyl	C_6_H_5_	391 (31 300)	471	0.04
3g^a^	4-EtO_2_C–C_6_H_4_	C_6_H_5_	371 (27 400)	600	0.02
3h^b^	C_6_H_5_	C_6_H_5_	374 (24 200)	500	0.08
3i^c^	C_6_H_5_	C_6_H_5_	377 (30 500)	544	0.08
3j^a^	C_6_H_5_	4-O_2_N–C_6_H_4_	371 (38 700)	607	0.14
3k^a^	C_6_H_5_	4-F-C_6_H_4_	372 (27 400)	575	0.04
3l^a^	C_6_H_5_	4-Br-C_6_H_4_	371 (26 100)	507	0.01
3m^a^	C_6_H_5_	2,6-Xylyl	394 (29 600)	577	0.01
3n^a^	C_6_H_5_	2-Naphthyl	371 (27 300)	536	0.25
3o^a^	C_6_H_5_	^*n*^Hexyl	378 (26 700)	544	0.02
3p^a^	C_6_H_5_	Benzyl	377 (29 000)	526	0.01
3q^d^	C_6_H_5_	Benzyl	381 (20 900)	592	0.01

a R^2^ = benzyl.

b R^2^ = 3,4,5-trimethoxybenzyl.

c R^3^ = 5,6-dimethyl.

d R^2^ = methyl.

The influence of the aroyl substituent R^1^ on the absorption properties is generally minor (3b–g); only the furan substituent (3f) leads to a pronounced bathochromic shift to 391 nm. This red shift can also be observed for the corresponding aroyl-*S*,*N*-ketene acetal bearing a furan substituent (see SI, Table S3). A similar trend is observed for aromatic thioethers (3j–n), where only the sterically demanding xylyl substituent (3m) induces a shift to longer wavelengths. Incorporation of alkyl-substituted thioethers (3o–q) results in a slight bathochromic shift as well. The most pronounced bathochromic shift of those of alkyl-substituted thioethers was observed for compound 3q, which, according to calculations, preferentially adopts the *Z-*configuration, leading to modified electronic properties.

For the majority of α-sulfenylated aroyl-*S*,*N*-ketene acetals 3, no emission can be detected in acetonitrile. Only derivatives 3b, 3j, 3o, and 3p show an emission band with a maximum between 546 and 552 nm; however, the corresponding intensities are very low.

**Fig. 2:**
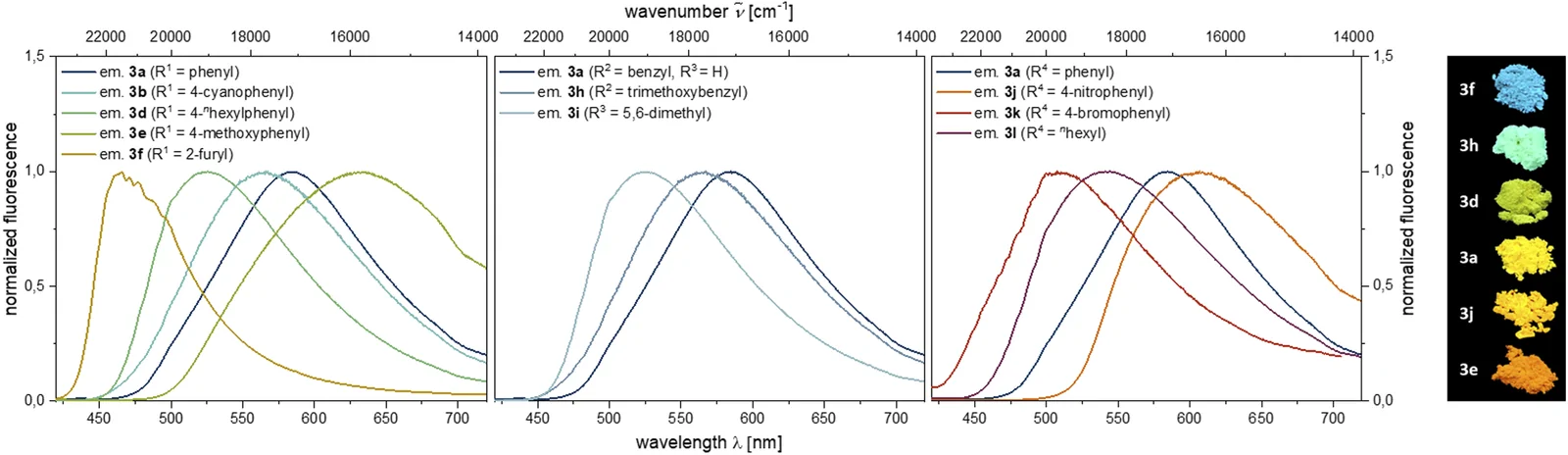
Comparison of the normalized solid-state emission spectra of dyes 3a, 3b, 3d, 3e, 3h, 3i, 3j, 3k and 3l (*T* = 298 K, *λ*_exc_ = *λ*_max,abs_) and solid-state fluorescence of dyes 3f, 3h, 3d, 3a, 3fj and 3e under the UV-lamp (*λ*_exc_ = 365 nm).

In contrast to the absence of emission in solution, chromophores 3 exhibit pronounced solid-state emission. This emission is significantly more sensitive to variation of the substituents. The α-sulfenylated aroyl-*S*,*N*-ketene acetals 3 display emission maxima in the range of 471 to 635 nm, corresponding to emission colors spanning from blue to orange (Fig. 2). Overall, a substantial bathochromic shift is observed in comparison to the solid-state emission of the aroyl-*S*,*N*-ketene acetals 1. Compound 3a emits with a maximum at 586 nm, whereas the corresponding aroyl-*S*,*N*-ketene acetal shows an emission maximum at 477 nm.^10^

However, no clear correlation between the electronic properties of the aroyl substituent and the position of the emission maximum can be established. Both the electron-withdrawing cyano substituent (3b) and the electron-donating *n*-hexyl substituent (3d) induce hypsochromic shifts. The most pronounced hypsochromic shift is observed for the furan derivative (3f). In contrast, the electron-rich methoxy-substituted derivative (3e) exhibits the most strongly bathochromically shifted emission maximum at 635 nm (Fig. 2, left). Dimethyl substitution at the benzothiazole core (3i), as well as the introduction of methoxy substituents at the benzyl moiety (3h), results in blue-shifted solid-state emission (Fig. 2, center).

The nature of the thioether substituent likewise influences the emission properties. The 4-bromophenyl derivative 3l exhibits the most pronounced hypsochromically shifted emission maximum at 507 nm, whereas the 4-nitrophenyl derivative 3j shows the most strongly bathochromically shifted maximum at 607 nm. Alkyl substitution (3p and 3o), as well as naphthyl substitution (3n), leads to a blue shift of the solid-state emission compared to a phenyl-substitution (Fig. 2, right). The highest quantum yields are observed for the unsubstituted derivative 3a and the naphthyl-substituted chromophore 3n (0.15 and 0.25, respectively, Table 4).

### Calculated electronic structure

**Fig. 3:**
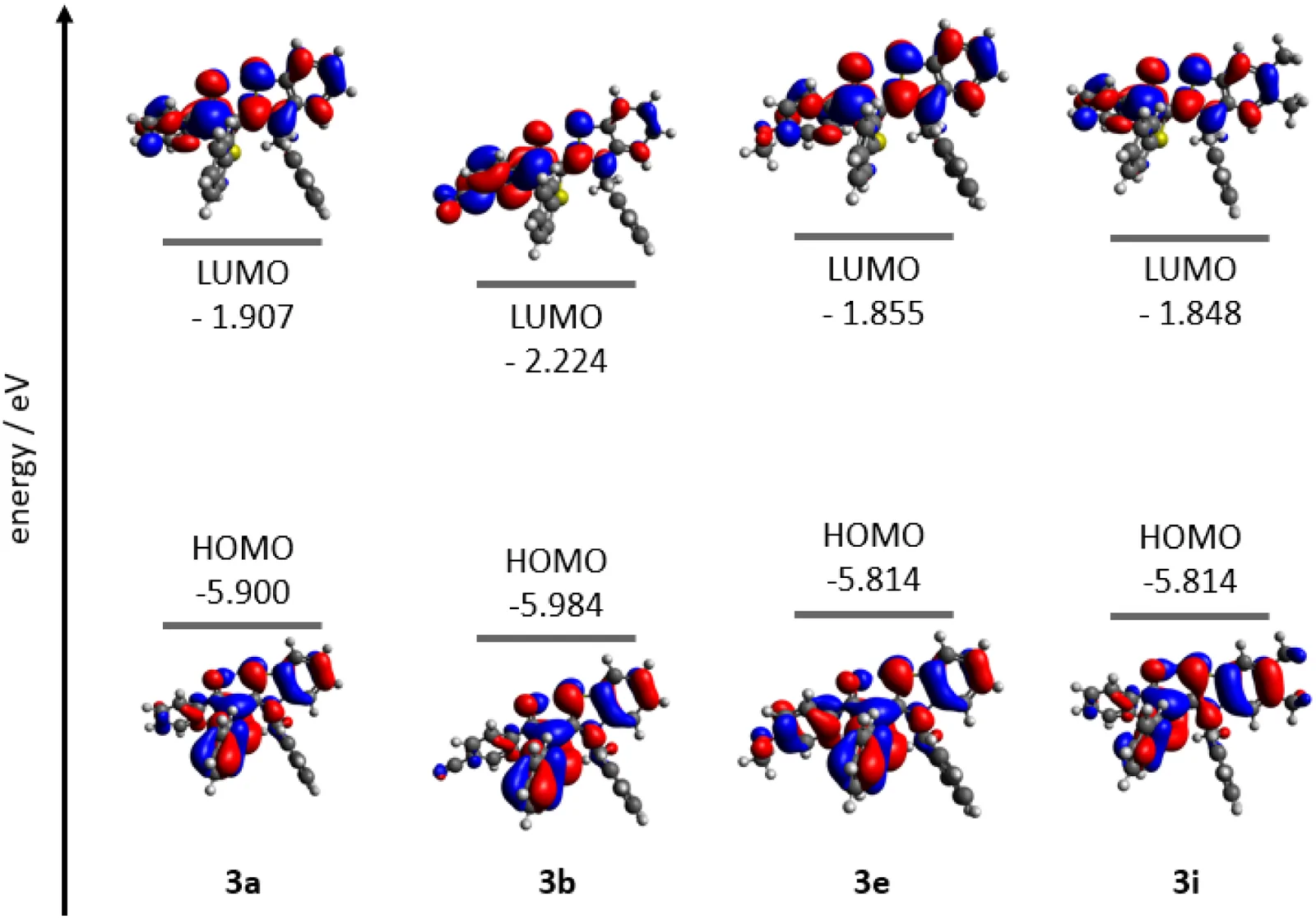
Kohn–Sham frontier molecular orbitals of selected *E*-configured α-sulfenylated aroyl-*S*,*N*-ketene acetals 3 and their energies (Gaussian 16, B3LYP/6-31G**, PCM acetonitrile).

In order to assign the experimentally observed longest wavelength absorption maxima to the corresponding electronic transitions and to gain deeper insight into the electronic structure, additional TD-DFT calculations were performed (Table 5). The B3LYP/6-31G** functional was chosen in accordance with our previous investigations of aroy-*S*,*N*-ketene acetals, allowing a direct comparison of the calculated electronic properties within this class of chromophores. Nevertheless, it should be noted that conventional TD-DFT-functionals may have limitations in accurately describing charge-transfer excitations. Therefore, the calculated transition energies are mainly considered for qualitative interpretation of the electronic structures and relative trends. Since the chromophores 3 may exist in two configurations in solution, the theoretical absorption maxima for both configurations were calculated. For the electroneutral and electron-rich thioethers, the computed longest wavelength transitions of the *E*-configured molecules are in good agreement with the experimentally observed absorption and can be attributed to HOMO–LUMO transitions. The HOMO is predominantly localized on the thioether moiety and the donor part of the merocyanine system, whereas the LUMO also exhibits significant electron density on the acceptor moiety, the aroyl fragment (Fig. 3). Therefore, HOMO–LUMO transitions can be considered as charge transfer transitions.

**Table 5:** TD-DFT calculations on the UV/vis absorption maxima of α-sulfenylated aroyl-*S*,*N*-ketene acetals 3 (Gaussian 16, B3LYP/6-31G**, PCM acetonitrile)

Compound	*λ*_max,abs(exp)_/nm (*ε*/L mol^−1^ cm^−1^)	*λ*_max,abs(calcd)_/nm (oscillator strength), most dominant contribution	
		*E*-Configuration of dye 3	*Z*-Configuration of dye 3
3a	371 (16 500)	377 (0.092), HOMO → LUMO (73%)	409 (0.275), HOMO → LUMO (91%)
3b	373 (18 500)	397 (0.132), HOMO → LUMO (81%)	437 (0.247), HOMO → LUMO (94%)
3c	373 (26 400)	377 (0.107), HOMO → LUMO (75%)	405 (0.270), HOMO → LUMO (92%)
3d	374 (28 400)	378 (0.136), HOMO → LUMO (77%)	408 (0.321), HOMO → LUMO (92%)
3e	377 (25 800)	379 (0.192), HOMO → LUMO (83%)	409 (0.349), HOMO → LUMO (94%)
3f	391 (31 300)	387 (0.196), HOMO → LUMO (72%)	412 (0.356), HOMO → LUMO (92%)
3g	371 (27 400)	393 (0.136), HOMO → LUMO (80%)	434 (0.263), HOMO → LUMO (94%)
3h	374 (24 200)	378 (0.093), HOMO → LUMO (76%)	391 (0.254), HOMO → LUMO (85%)
3i	377 (30 500)	373 (0.136), HOMO−1 → LUMO (49%)	410 (0.343), HOMO → LUMO (92%)
3j	371 (38 700)	408 (0.325), HOMO−1 → LUMO (98%)	397 (0.373), HOMO−1 → LUMO (89%)
3k	372 (27 400)	378 (0.093), HOMO → LUMO (76%)	410 (0.275), HOMO → LUMO (92%)
3l	371 (26 100)	374 (0.118), HOMO → LUMO (78%)	408 (0.289), HOMO → LUMO (93%)
3m	394 (29 600)	390 (0.373), HOMO → LUMO (96%)	446 (0.230), HOMO → LUMO (98%)
3n	371 (27 300)	379 (0.098), HOMO → LUMO (90%)	403 (0.157), HOMO → LUMO (92%)
3o	378 (26 700)	365 (0.309), HOMO → LUMO (67%)	406 (0.362), HOMO → LUMO (94%)
3p	377 (29 000)	361 (0.374), HOMO → LUMO (69%)	404 (0.402), HOMO → LUMO (93%)
3q	381 (20 900)	357 (0.478), HOMO → LUMO (70%)	394 (0.420), HOMO → LUMO (95%)

An exception is derivative 3j, where the LUMO is entirely localized on the thioether unit. This behavior is attributed to the strongly electron-withdrawing nitro group, which lowers the energy of the corresponding molecular orbital. The computed longest wavelength absorption in this case arises from a HOMO−1–LUMO transition and deviates significantly from the experimentally determined absorption maximum (Table 5). Therefore, only electroneutral and electron-rich thioethers can be conclusively compared on the basis of the TD-DFT calculations.

For dye 3i, the longest wavelength transition proceeds approximately equally from HOMO and HOMO−1; the latter is predominantly localized on the sulfenyl moiety and the HOMO is more strongly localized on the benzothiazole moiety. This effect is caused by the two methyl substituents, which increase the electron density and raise the energy of the corresponding molecular orbital.

The calculated absorption maxima of the *Z*-configured α-sulfenylated aroyl-*S*,*N*-ketene acetals 3 deviate more significantly from the experimental values and are consistently bathochromically shifted. This behavior can be attributed to the more pronounced torsion and the increased bond-length alternation in the *Z*-configuration, as discussed above. It can therefore be concluded that the observed absorption predominantly originates from the *E*-configured chromophores. This finding is consistent with the computational results indicating that α-sulfenylated aroyl-*S*,*N*-ketene acetals 3 are preferentially represented by the *E*-configuration in solution, which also governs their photophysical properties. The only exception is the *N*-methyl derivative 3q, for which the absorption is better described by the calculated transitions of the *Z*-configuration. As discussed above, this isomer is predicted to be more stable in solution, and the results are therefore consistent. In contrast, for chromophores 3f and 3h, which are also calculated to be more stable in the *Z*-configuration, this preference is not reflected by the computed absorption data.

In comparison to the aroyl-*S*,*N*-ketene acetals 1, whose longest wavelength absorption oscillator strengths exceed 0.5,^10^*E*-configured α-sulfenylated aroyl-*S*,*N*-ketene acetals 3 display lower oscillator strengths, in accordance with the experimentally observed lower extinction coefficients. In most cases, the oscillator strengths are found to be below 0.2; only derivatives 3m, 3o, 3p, and 3q exhibit higher oscillator strengths in the range of 0.3–0.5.

### Aggregation induced emission

Nearly all *N*-benzyl-aroyl-*S*,*N*-ketene acetals exhibit aggregation-induced emission (AIE) behavior in ethanol–water mixtures.^8^ The novel chromophores 3 are non-emissive in solution, yet, they display pronounced solid-state emission, and AIE studies were conducted for all compounds. Because of the limited solubility of dyes 3 in ethanol, acetonitrile was chosen as an organic solvent, with water used to induce aggregation.

With the exception of the *n*-hexylthio derivative 3o, all α-sulfenylated *N*-benzyl-aroyl-*S*,*N*-ketene acetals 3a–n and 3p exhibit emission upon induced aggregation. Even the *N*-methyl-substituted derivative 3q shows an increase in emission intensity upon aggregation; however, this effect is less pronounced than for the *N*-benzyl derivatives (see SI). This observation is consistent with previous findings for aroyl-*S*,*N*-ketene acetals, which indicate that the *N*-benzyl substituent plays a crucial role in AIE behavior.^10^

**Fig. 4:**
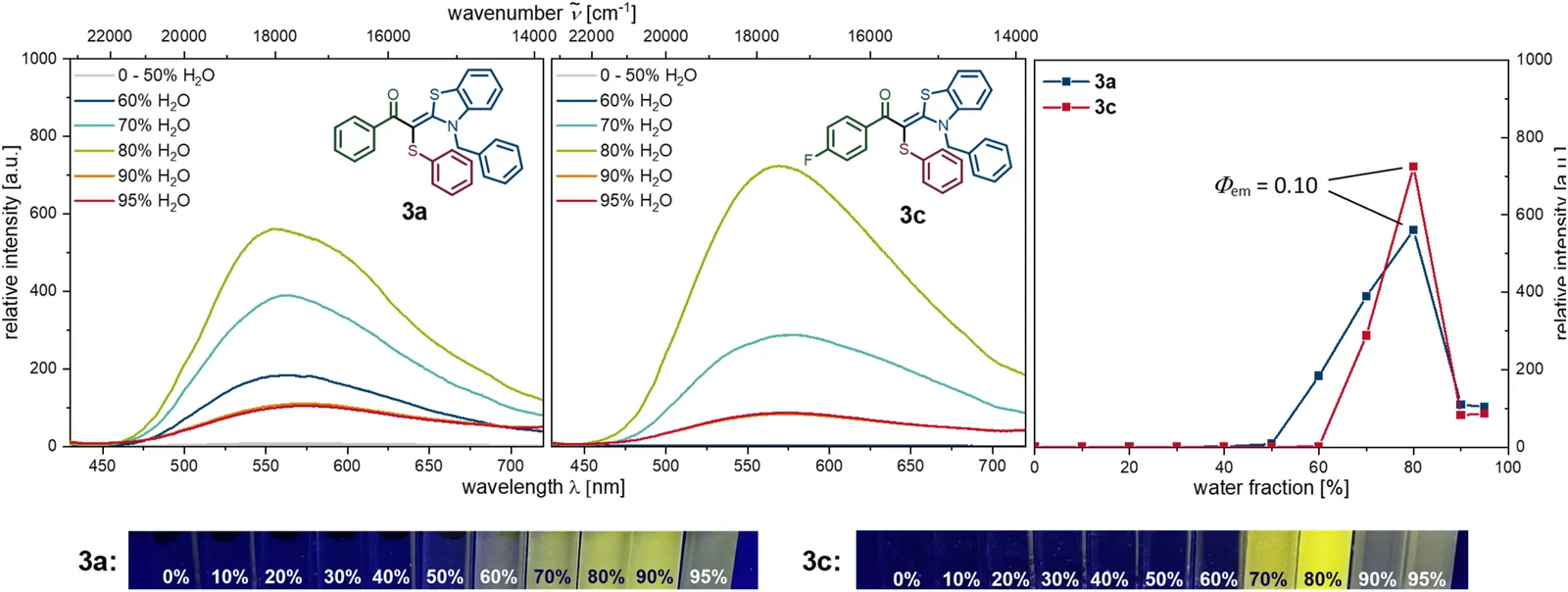
(Top) Emission spectra of 3a (left) and 3c (middle) in acetonitrile–water mixtures with increasing water fraction (*T* = 298 K, *c* = 10^−6^ M, *λ*_exc_ = *λ*_max,abs_) and emission intensity *versus* water fraction of 3a and 3c (right); (below) 3a (left) and 3c (right) in acetonitrile–water mixtures under the UV-lamp (*λ*_exc_ = 365 nm).

The most pronounced AIE characteristics are observed for compounds 3a and 3c (Fig. 4), where no measurable emission in solution is detected. For dye 3a, emission becomes apparent at a water content of 60%, whereas for dye 3c, emission is observed from 70% water content onward, reaching a maximum in both cases at a water content of 80% with an emission quantum yield amounting to 0.10. At higher water fractions, the emission intensity decreases due to coagulation. The emission maxima in the aggregated state are similar to those observed in the solid state and are located at 555 and 570 nm, respectively.

The other α-sulfenylated *N*-benzyl-derivatives display significantly less pronounced AIE behavior, making chromophores 3a and 3c particularly noteworthy as efficient AIE emitters within this novel series aroyl-*S*,*N*-ketene acetals.

## Conclusions

The three-component condensation–sulfenylation reaction provides a simple and modular approach to the novel class of α-sulfenylated aroyl-*S*,*N*-ketene acetals. α-Sulfenylation of intermediary formed aroyl-*S*,*N*-ketene acetals furnishes various aromatic and aliphatic thioethers and results in new AIEgens with altered properties. The introduction of the α-sulfenyl group weakens the C=C double bond, enabling free rotation in solution according to TD-DFT calculation. Photophysical studies reveal a significant bathochromic shift of the emission compared to parent aroyl-*S*,*N*-ketene acetals, with solid-state emission colors ranging from blue to orange and quantum yields of up to 0.25. In particular, derivatives 3a and 3c display enhanced AIE characteristics, demonstrating that α-sulfenyl functionalization provides access to red-shifted emissive materials without compromising emission efficiency. These findings underline that α-functionalization to the fully substituted double bond of the underlying chromophore opens additional opportunities for fine-tuning the electronic properties of aroyl-*S*,*N*-ketene acetal AIEgens. The combination of tuneable solid-state emission and pronounced aggregation-induced emission highlights the potential of α-sulfenylated aroyl-*S*,*N*-ketene acetals as promising AIEgens, especially upon encapsulation for increased emission efficiency and for future applications in solid-state optoelectronic devices, sensing systems and bioimaging-related technologies. Further studies embedding the chromophores in polymer microparticles for emission enhancement and closer inspection of intersystem crossing pathways are currently underway.

## Author contributions

Methodologic and synthetic studies, analytical characterization of the synthetic samples, photophysics (steady-state absorption and emission spectroscopy) by J. K. and P. L., and quantum chemical calculation by J. K., who compiled the obtained data. V. V. and S. M. performed the X-ray structure analysis, J. K. interpreted and compiled the data for Hirshfeld analysis. The conceptualization, project administration, funding acquisition, data interpretation and discussion, reviewing and editing by T. J. J. M. The writing of the first draft was completed by J. K. All authors have read and agreed to the published version of the manuscript.

## Conflicts of interest

There are no conflicts to declare.

## Supplementary Material

RA-016-D6RA04851J-s001

RA-016-D6RA04851J-s002

## Data Availability

CCDC 2553034–2553039 contain the supplementary crystallographic data for this paper.^40*a*–*f*^ The data supporting this article have been included as a part of the supplementary information (SI). Ref. 9, 10, 22–24 and 26–39 from the manuscript are cited in the SI as ref. 1–19. Supplementary information: synthetic, analytic, and spectroscopic details of compounds 3, as well as details on the TD-DFT-calculations. See DOI: https://doi.org/10.1039/d6ra04851j.
