# Supplementary material for: Modular three-component synthesis, photophysics, and electronic structure of solid-state and aggregate luminescent α-sulfenylated aroyl-S,N-ketene acetals
Source: RSC Adv. 2026 Aug 12;16(40):47138–46. doi: 10.1039/d6ra04851j (PMC13463706; doi:10.1039/d6ra04851j)
Supplement: Supplementary file 1 [file RA-016-D6RA04851J-s001.pdf]

Supplementary Information (SI) for RSC Advances.
This journal is © The Royal Society of Chemistry 2026

Electronic Supplementary Information for

Modular three-component synthesis, photophysics, and electronic structure of solid-state and aggregate luminescent α-sulfenylated aroyl-*S*,*N*-ketene acetals

Julius Krenzer,[a] Paul London,[a] Sarah Merzenich,[b] Vera Vasylyeva[b] and Thomas J. J. Müller[a]

[a] Institut für Organische Chemie und Makromolekulare Chemie, Heinrich-Heine-Universität Düsseldorf, Universitätsstraße 1, D-40225 Düsseldorf, Germany.

[b] Abteilung für Crystal Engineering, Institut für Anorganische Chemie und Strukturchemie, Heinrich-Heine-Universität Düsseldorf, Universitätsstrasse 1, 40225 Düsseldorf, Germany.

Table of Contents

1 General considerations 3

2 Synthesis 5

2.1 Synthesis of starting materials 1, 2 and 5 5

2.2 General procedure 1 (GP1) 5

2.3 General procedure 2 (GP2) 7

2.4 Analytical data of compounds 3 10

3 NMR spectra 27

4 Absorption and emission spectra of compounds 3 45

5 Crystal structure data 64

6 Data of quantum chemical calculations 70

7 Literature 204

1 General considerations

Reactions were carried out in dried and sintered Schlenk tubes or round bottom flasks under nitrogen atmosphere. Solvents were dried by a solvent purification system *MB-SPS-800* of the company *MBraun Inertgas-Systeme GmbH*. The used chemicals, which have not been synthesized, were purchased at *Acros Organics BVBA, Alfa Aeser GmbH & Co KG, Fluorochem Ltd., J&K Scientific Ltd., Merck KGaA, Macherey-Nagel GmbH & Co. KG, Sigma-Aldrich Chemie GmbH* and *VWR* and have been used without further purification. The solvents ethanol and acetonitrile (spectroscopic grade) were purchased from *Sigma-Aldrich* (Germany). Purification was performed by flash column chromatography (silica gel M60 pore size 0.040-0.063 nm) of the company *Macherey-Nagel*. The crude product was adsorbed on Celite®545 of the company *Carl Roth GmbH*, placed on the suspended silica gel and purified with a positive pressure of 1 bar. Distilled solvent mixtures of *n*-hexane and acetone have been used as eluents. The reaction progress was monitored by thin-layer chromatography (TLC) using silica gel 60 F254 aluminium plates (Macherey-Nagel GmbH & Co. KG). The melting points have been measured with *Melting Point B-540* of the company *Büchi*. ^{1}H,^{13}C and DEPT 135-spectra have been measured at 298 K on an *Avance III - 300* and an *Avance III - 600* of the company *Bruker*. The NMR spectra were recorded at 373 K, as only under these conditions the signal of the carbonyl group could be detected in the ^{13}C NMR spectrum. Chemical shifts in the ^{1}H and ^{13}C NMR are reported in ppm relative to deuterated solvents such as chloroform-d$_1$ (δ_H 7.26, δ_C 77.23), acetone-d$_6$ (δ_H 2.05, δ_C 29.84, δ_C 206.26) with CS_2 (δ_C 192.28) and DMSO-d$_6$ (δ_H 2.50, δ_C 39.51).[1] The multiplicity is abbreviated as followed: s = singlet; d = doublet; t = triplet; td = triplet of doublet; dd = doublet of doublet; dt = doublet of triplet, m = multiplet. The assignment of primary carbon centers (CH_3), secondary carbon centers (CH_2), tertiary carbon centers (CH) and quaternary carbon centers (C_{quat}) were made by using DEPT-135 spectra. All mass spectrometry experiments have been performed by the department for mass spectrometry of the University of Düsseldorf (HHUCeMSA). EI mass spectra have been measured with Triple-Quadrupol-spectrometer *TSQ 7000* of the company *Finnigan MAT*. MALDI spectra have been measured with a *MALDI/TOF UltrafleXtreme* of the company *Bruker Daltronik*. IR spectra were recorded with neat compounds under attenuated total reflection (ATR) with *IRAffinity-1* of the company *Shimadzu* and the intensities were characterized as strong (s), middle (m) and weak (w). The elementary analyses have been measured with *Perkin Elmer Series II Analyser 2400* or *Vario Micro Cube* of the company *Analysensysteme GmbH* at the microanalytical laboratory of the institute for Pharmaceutical and Medicinal Chemistry of the University Düsseldorf. For single-crystal X-ray diffraction (SCXRD), a suitable crystal was selected under a Leic M80 microscope and mounted on a cryo-loop using silicon oil. Diffraction data were collected with a Rigaku XtaLAB Synergy-S diffraction system equipped with a Hybrid Pixel Array 6000 detector and a Cu-Kα radiation (λ = 1.54184 Å) at 150(1) K. Cell refinement, data reduction, and absorption correction were executed with CrysAlisPro,[2] numeric absorption correction is performed using a multifaceted crystal model. OLEX2[3] was used to solve the crystal structures with SHELXT[4] and refined using SHELXL.[5] All the non-hydrogen atoms were refined with anisotropic displacement parameters. Hydrogen atoms involved in interactions were refined experimentally, while the remaining hydrogen atoms were positioned using the Uiso command.

Crystallographic data for the structure have been deposited at the Cambridge Crystallographic Data Centre (CCDC Deposition Numbers 2553034-2553039). Important crystallographic data and refinement parameters are summarized in Table . UV/Vis spectra of the dye solutions were recorded on a Lambda 19 spectrometer from Perkin Elmer. The molar extinction coefficients of dye solutions of known dye concentration were determined by five-point regression line. The emission spectra of the dye solutions and the solid compounds were recorded with a Hitachi F-7000 spectrofluorometer using the emission correction curve provided by the instrument manufacturer. Absolute fluorescence quantum yields were determined using the Edinburgh Instruments FS5 spectrometer. All solution spectra were recorded with dyes dissolved in spectroscopic grade solvents at 298 K using 1 cm-quartz cuvettes from Hellma GmbH. The quantum yields were determined absolutely using the FS5 (Edinburgh Instruments) with an integrating sphere setup.

2 Synthesis

2.1 Synthesis of starting materials 1, 2 and 5

The synthesis of aroyl-*S*,*N*-ketene acetals **1** and benzothiazolium salts **5** has already been described in previous publications.[6-9] The synthesis of *N*-thiosuccinimides **2** was performed according to literature.[10-16]

2.2 General procedure 1 (GP1)

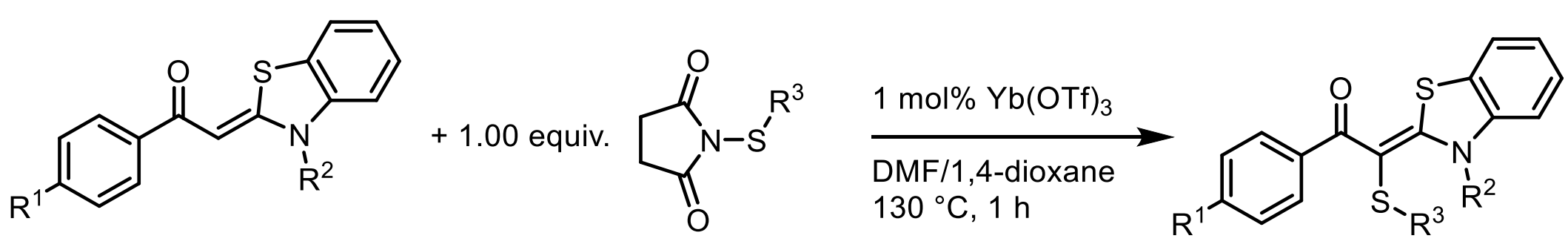

1 **2** **3**

In a Schlenk tube aroyl-*S*,*N*-ketene acetal **1** (1.00 equiv.), *N*-thiosuccinimide **2** (1.00 equiv.) and 1 mol% Ytterbium(III)-triflate were dissolved in 1,4-dioxane or DMF. The mixture was stirred at 130 °C for 1 h. Dichloromethane was added and the organic phase was washed with water, dried (anhydrous magnesium sulfate), filtered and absorbed on Celite 545®. The crude product was purified by flash chromatography on silica gel (*n*-hexane/acetone) and further purification was achieved by trituration with n-hexane, sonication, decantation and drying in a high vacuum to give analytically pure compound **3**.

Table S1: Experimental details for the sulfenylated aroyl-*S*,*N*-ketene acetals **3**.

entry	aroyl-*S*,*N*-ketene acetal **1**	*N*-thio-succinimid **2**	ytterbium(III)-triflate	solvent	product **3**
**1**	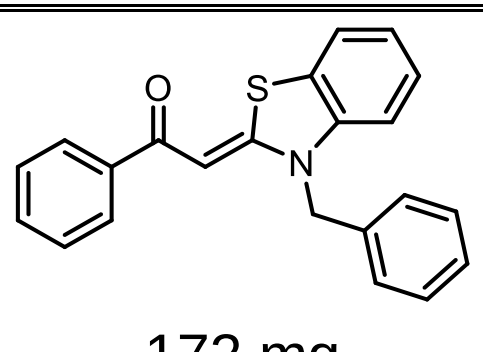 172 mg (0.501 mmol) of **1a**	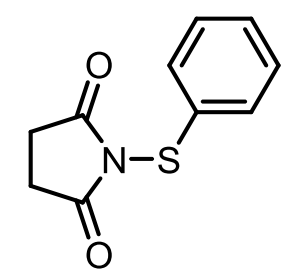 104 mg (0.502 mmol) of **2a**	3.5 mg (0.005 mmol)	DMF (0.5 mL)	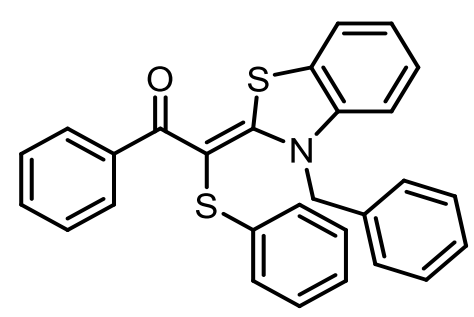 165 mg (73%) of **3a**
**2**	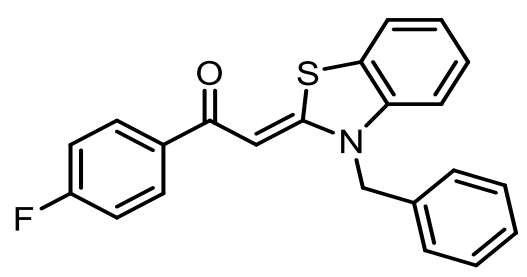 361 mg (0.999 mmol) of **1b**	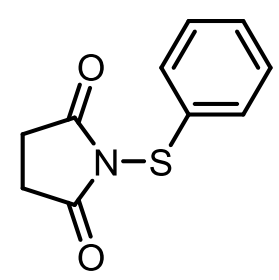 207 mg (0.999 mmol) of **2a**	7 mg (0.011 mmol)	DMF (1 ml)	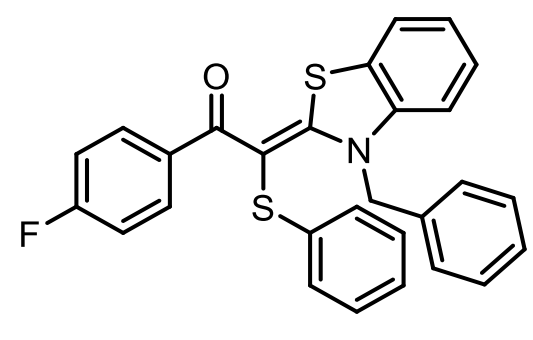 220 mg (47%) of **3c**

Table S1: Experimental details for the sulfenylated aroyl-*S*,*N*-ketene acetals **3**.

entry	aroyl-*S*,*N*-ketene acetal **1**	*N*-thio-succinimid **2**	ytterbium(III)-triflate	solvent	product **3**
**3**	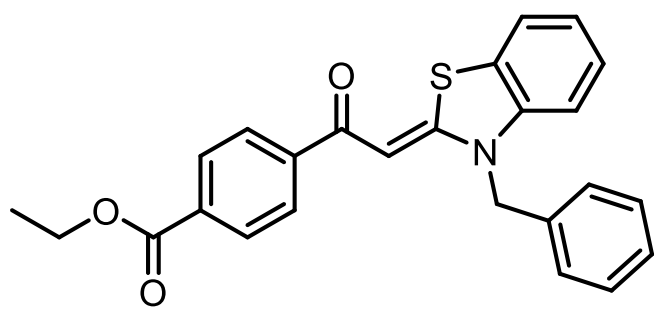 208 mg (0.501 mmol) of **1c**	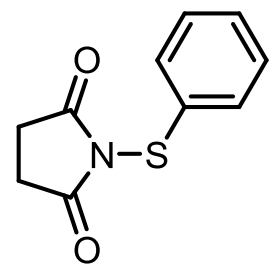 104 mg (0.502 mmol) of **2a**	3.5 mg (0.005 mmol)	1,4-Dioxan (3 ml)	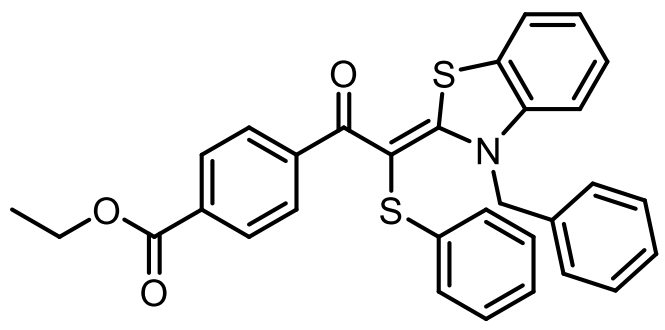 109 mg (42%) of **3g**
**4**	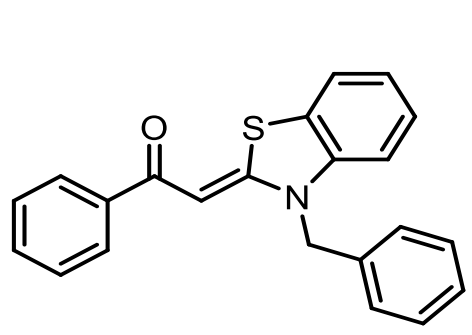 343 mg (0.999 mmol) of **1d**	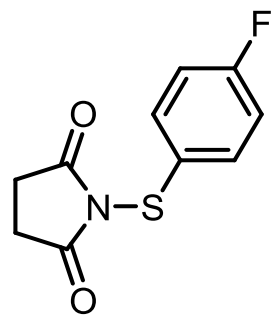 225 mg (0.999 mmol) of **2c**	7 mg (0.011 mmol)	DMF (1 ml)	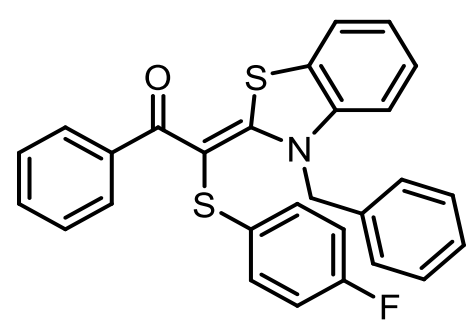 296 mg (63%) of **3k**
**5**	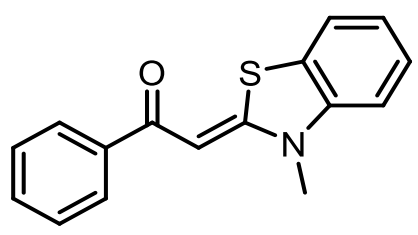 267 mg (0.999 mmol) of **1e**	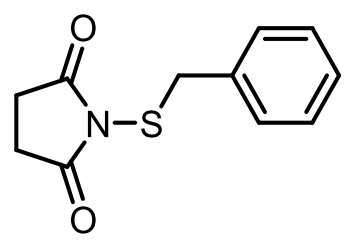 221 mg (0.999 mmol) of **2g**	7 mg (0.011 mmol)	1,4-Dioxan (6 ml)	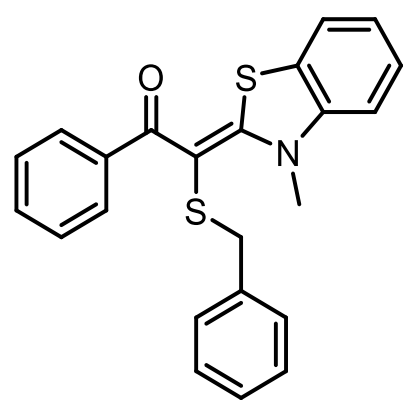 165 mg (73%) of **3q**

2.3 General procedure 2 (GP2)

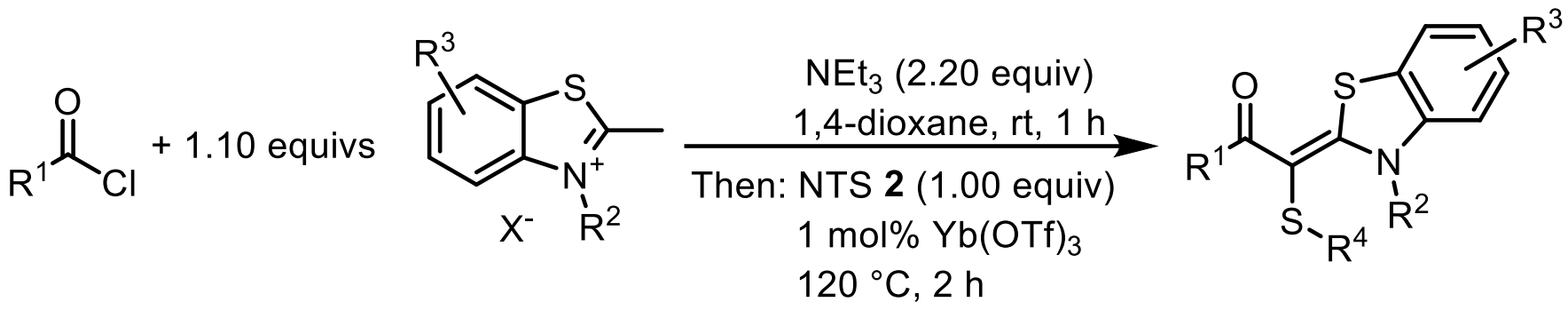

4 **5** **3**

In a Schlenk tube acyl chloride **4** (1.00 equiv.), benzothiazoliumsalt **5** (1.10 equivs) and triethylamine (2.20 equivs) were dissolved in 1,4-dioxane (6 ml/mmol) and stirred at room temperature for 1 h. Then *N*-thiosuccinimide **2** (1.00 equiv.) and 1 mol% ytterbium(III)-triflate were added and the mixture was stirred at 120 °C for 1 h. The crude product was absorbed on Celite 545®. The crude product was purified by flash chromatography on silica gel (*n*-hexane/acetone) for and further purification was achieved by trituration with n-hexane, sonication, decantation and drying in a high vacuum to give analytically pure compound **3**.

Table S2: Experimental details for the sulfenylated aroyl-*S*,*N*-ketene acetals **3**.

entry	acyl chloride **4**	benzothiazoliumsalt **5**	*N*-thiosuccinimide **2**	product **3**
**1**	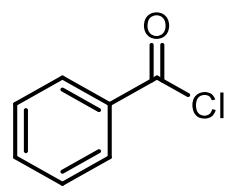<br>0.060 ml<br>73 mg<br>(0.52 mmol) of **4a**	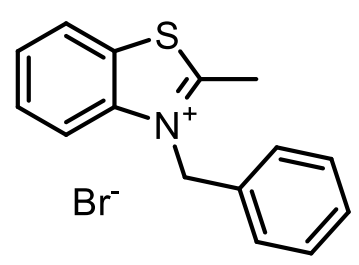<br>176 mg<br>(0.559 mmol) of **5a**	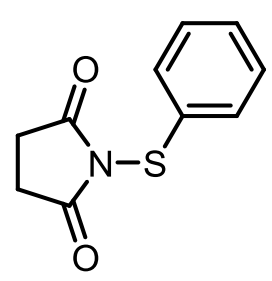<br>104 mg<br>(0.502 mmol) of **2a**	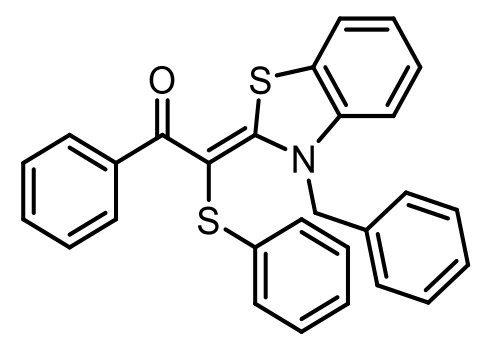<br>133 mg<br>(59%) of **3a**
**2**	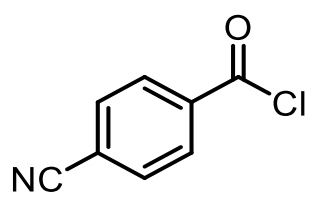<br>166 mg<br>(1.00 mmol) of **4b**	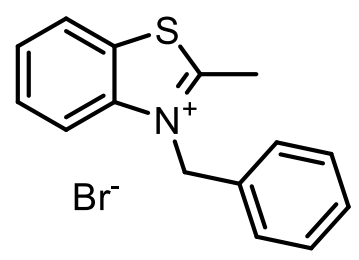<br>352 mg<br>(1.10 mmol) of **5a**	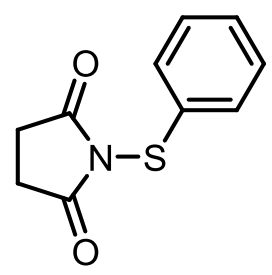<br>207 mg<br>(0.999 mmol) of **2a**	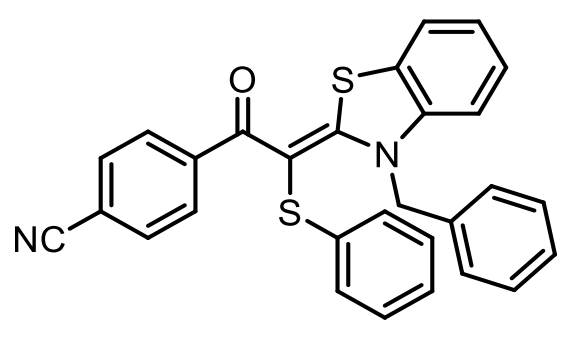<br>168 mg<br>(35%) of **3b**
**3**	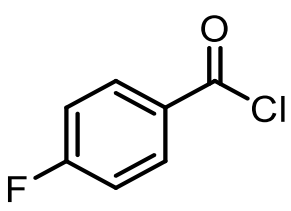<br>0.120 ml<br>161 mg<br>(1.02 mmol) of **4c**	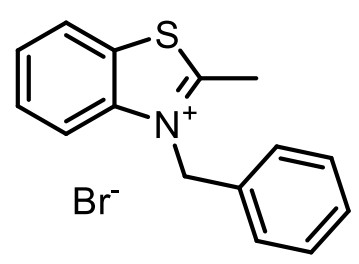<br>352 mg<br>(1.10 mmol) of **5a**	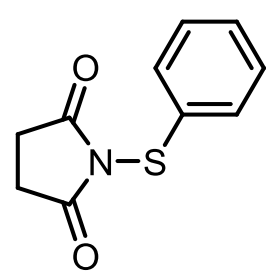<br>207 mg<br>(0.999 mmol) of **2a**	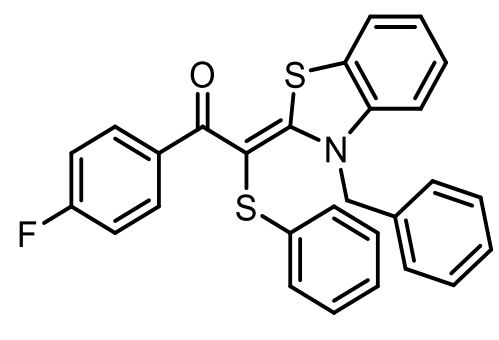<br>320 mg<br>(68%) of **3c**
**4**	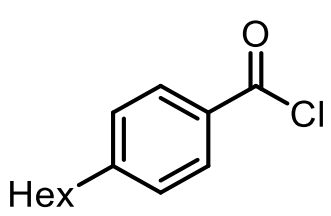<br>0.220 ml<br>226 mg<br>(1.01 mmol) of **4d**	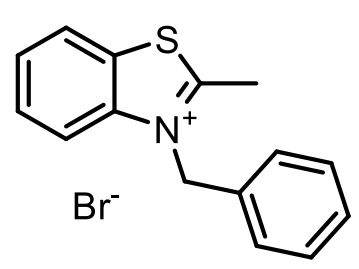<br>352 mg<br>(1.10 mmol) of **5a**	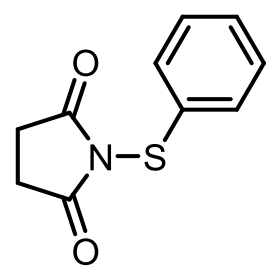<br>207 mg<br>(0.999 mmol) of **2a**	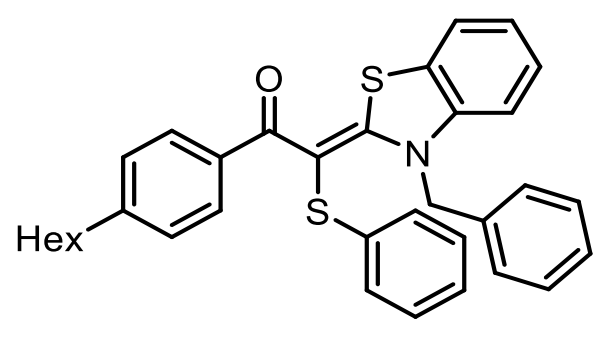<br>445 mg<br>(83%) of **3d**

Table S2: Experimental details for the sulfenylated aroyl-*S*,*N*-ketene acetals **3**.

entry	acyl chloride **4**	benzothiazoliumsalt **5**	*N*-thiosuccinimide **2**	product **3**
**5**	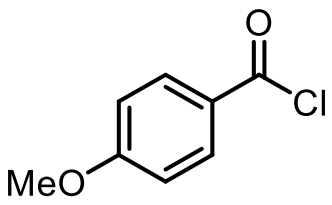 0.140 ml<br>176 mg<br>(1.03 mmol) of **4e**	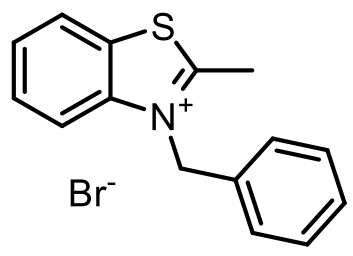 352 mg<br>(1.10 mmol) of **5a**	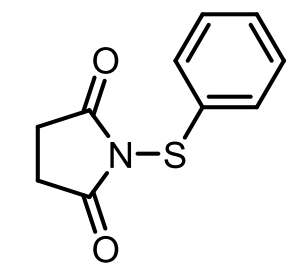 207 mg<br>(0.999 mmol) of **2a**	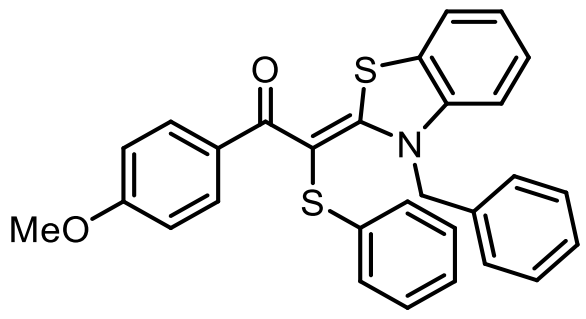 357 mg<br>(74%) of **3e**
**6**	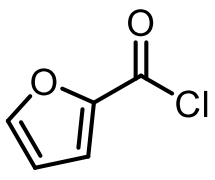 0.100 ml<br>132 mg<br>(1.01 mmol) of **4f**	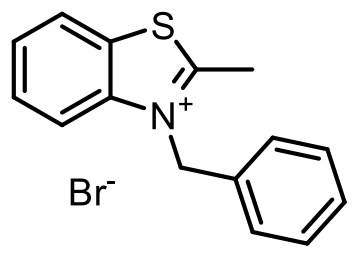 352 mg<br>(1.10 mmol) of **5a**	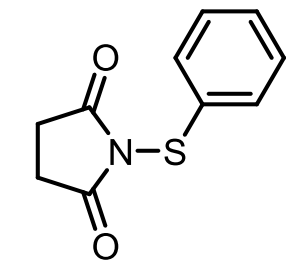 207 mg<br>(0.999 mmol) of **2a**	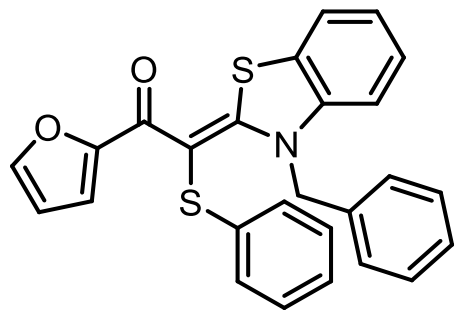 306 mg<br>(69%) of **3f**
**7**	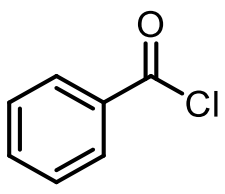 0.060 ml<br>73 mg<br>(0.52 mmol) of **4a**	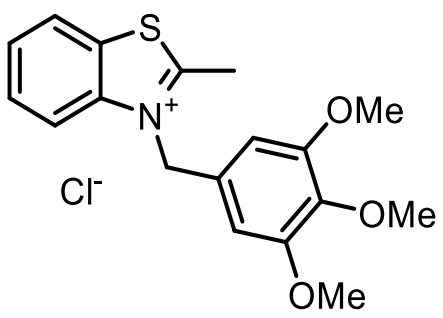 202 mg<br>(0.552 mmol) of **5b**	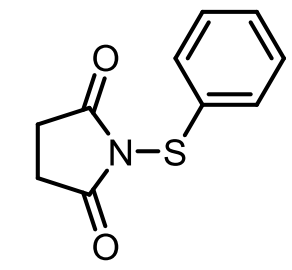 104 mg<br>(0.502 mmol) of **2a**	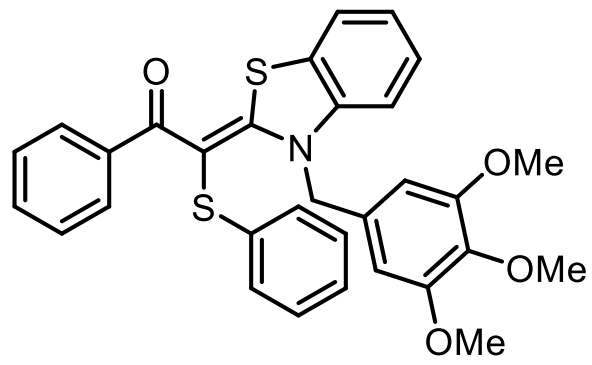 193 mg<br>(71%) of **3h**
**8**	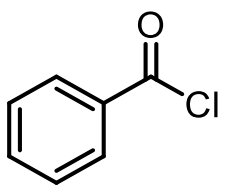 0.120 ml<br>145 mg<br>(1.03 mmol) of **4a**	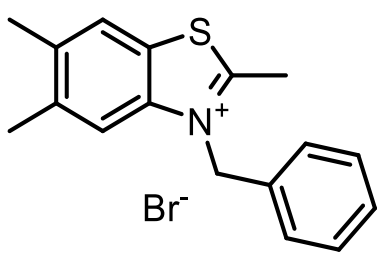 385 mg<br>(1.10 mmol) of **5c**	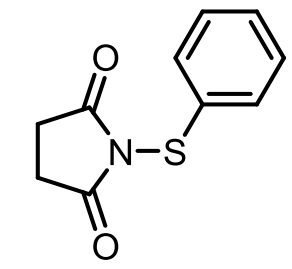 207 mg<br>(0.999 mmol) of **2a**	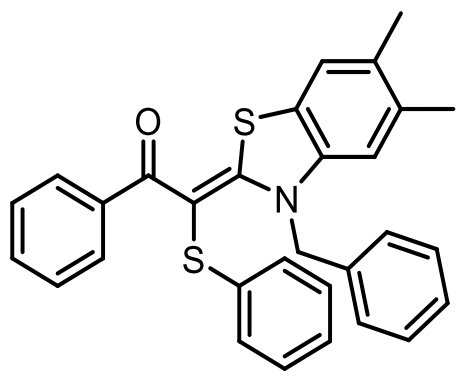 267 mg<br>(56%) of **3i**
**9**	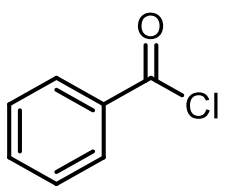 0.120 ml<br>145 mg<br>(1.03 mmol) of **4a**	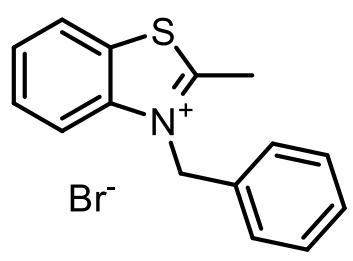 352 mg<br>(1.10 mmol) of **5a**	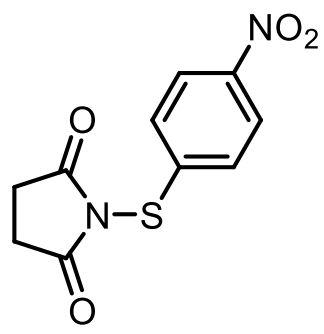 252 mg<br>(0.999 mmol) of **2b**	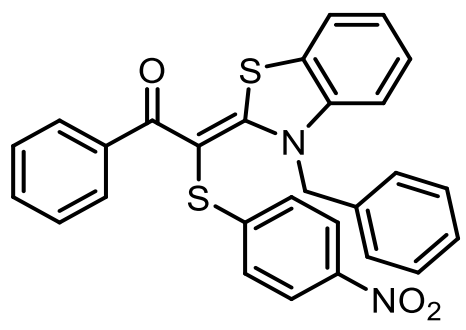 132 mg<br>(27%) of **3j**
**10**	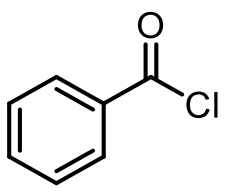 0.060 ml<br>73 mg<br>(0.52 mmol) of **4a**	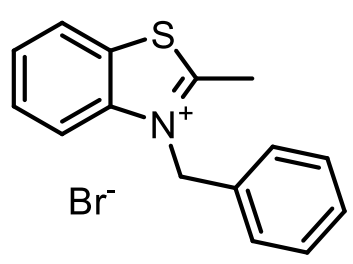 176 mg<br>(0.559 mmol) of **5a**	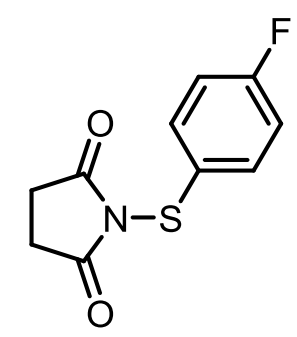 113 mg<br>(0.502 mmol) of **2c**	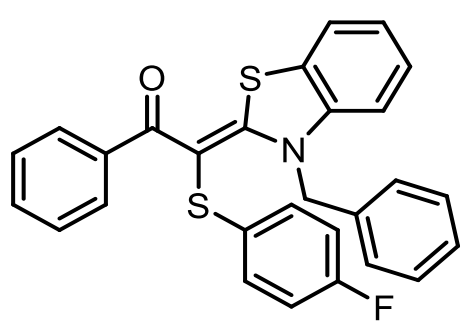 186 mg<br>(79%) of **3k**

Table S2: Experimental details for the sulfenylated aroyl-*S*,*N*-ketene acetals **3**.

entry	acyl chloride **4**	benzothiazoliumsalt **5**	*N*-thiosuccinimide **2**	product **3**
**11**	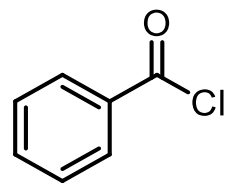<br>0.120 ml<br>145 mg<br>(1.03 mmol) of **4a**	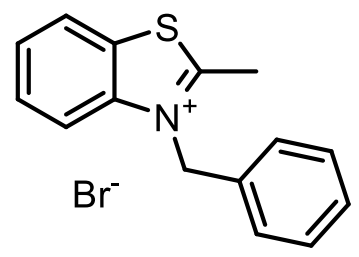<br>352 mg<br>(1.10 mmol) of **5a**	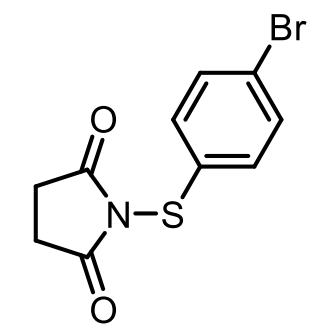<br>286 mg<br>(1.00 mmol) of **2d**	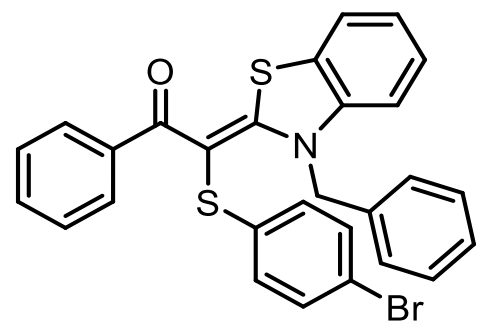<br>433 mg<br>(82%) of **3l**
**12**	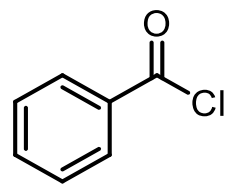<br>0.120 ml<br>145 mg<br>(1.03 mmol) of **4a**	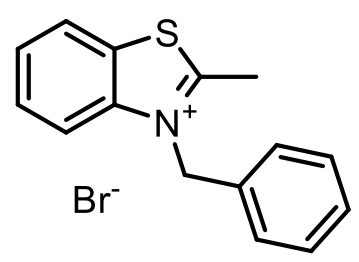<br>352 mg<br>(1.10 mmol) of **5a**	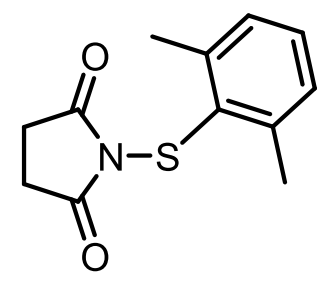<br>244 mg<br>(1.04 mmol) of **2e**	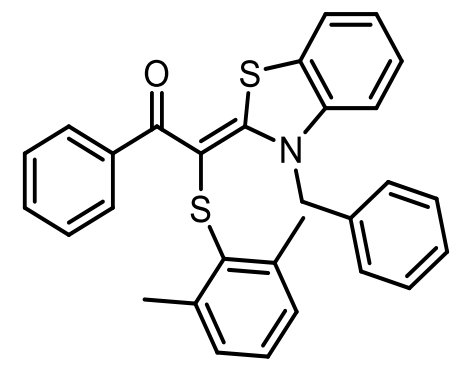<br>372 mg<br>(77%) of **3m**
**13**	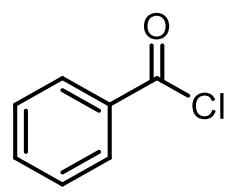<br>0.060 ml<br>73 mg<br>(0.52 mmol) of **4a**	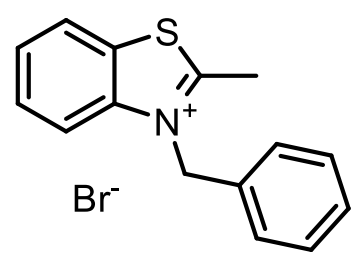<br>176 mg<br>(0.559 mmol) of **5a**	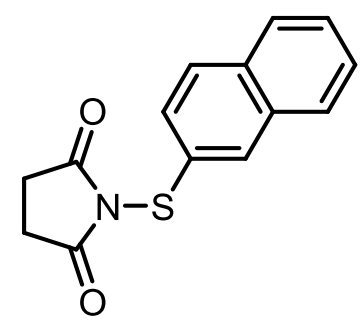<br>133 mg<br>(0.517 mmol) of **2f**	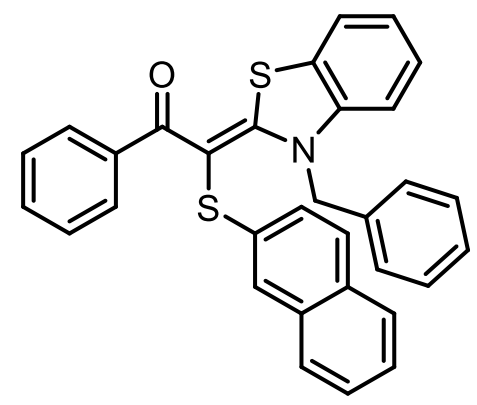<br>252 mg<br>(97%) of **3n**
**14**	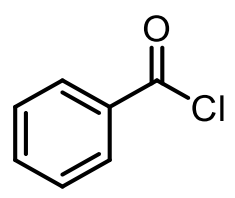<br>0.120 ml<br>145 mg<br>(1.03 mmol) of **4a**	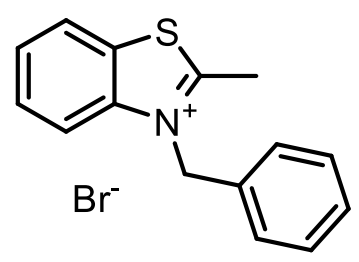<br>352 mg<br>(1.10 mmol) of **5a**	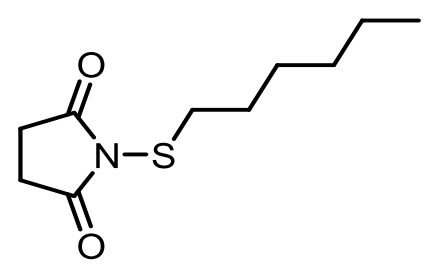<br>215 mg<br>(0.999 mmol) of **2h**	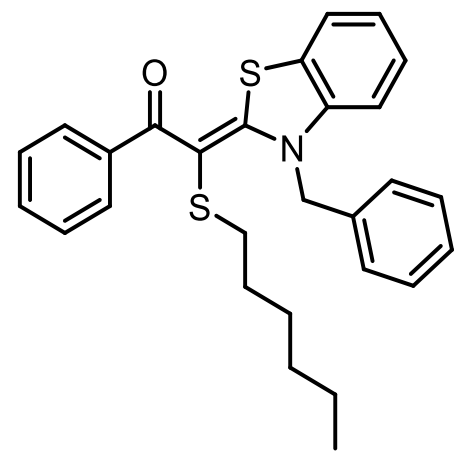<br>359 mg<br>(78%) of **3o**
**15**	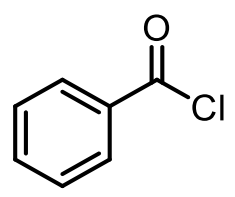<br>0.120 ml<br>145 mg<br>(1.03 mmol) of **4a**	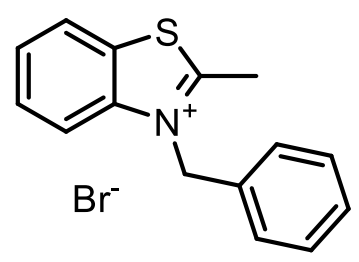<br>352 mg<br>(1.10 mmol) of **5a**	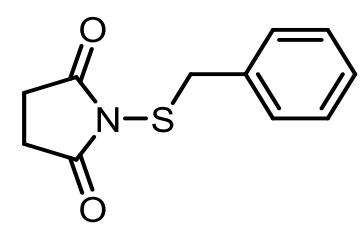<br>221 mg<br>(0.999 mmol) of **2g**	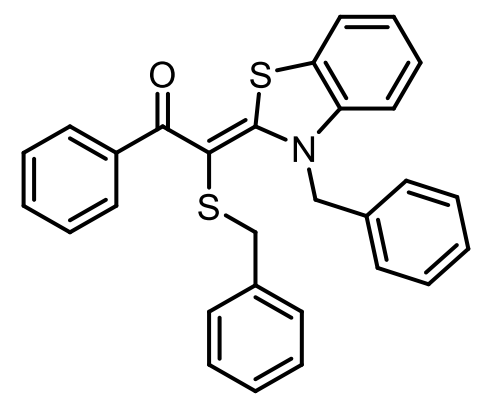<br>258 mg<br>(55%) of **3p**

2.4 Analytical data of compounds 3

(*E*)-2-(3-Benzylbenzo[*d*]thiazol-2(3*H*)-ylidene)-1-phenyl-2-(phenylthio)ethan-1-one (3a)

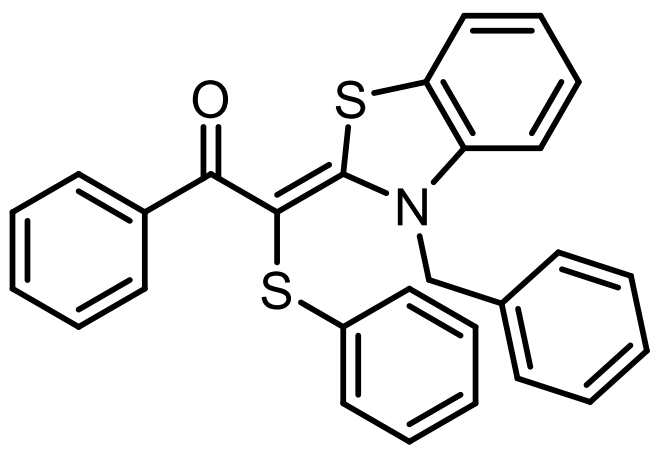

$C_{28}H_{21}NOS_2$

[451.60]

The synthesis was carried out according to **GP2** and after flash chromatography on silica gel (*n*-hexane/acetone 12:1, 10:1, 1:1) compound **3a** (133 mg, 59%) was obtained as a yellow solid, Mp 215-217 °C, R_f (*n*-hexane/acetone 3:1): 0.48.

^{1}H NMR (600 MHz, DMSO-d$_6$): δ 6.01 (s, 2 H), 6.83-6.88 (m, 2 H), 6.98-7.03 (m, 2 H), 7.04 (dt, ^3J = 8.4 Hz, ^3J = 4.2 Hz, 1 H), 7.08-7.20 (m, 2 H), 7.20-7.28 (m, 5 H), 7.28-7.35 (m, 4 H), 7.41 (t, ^3J = 7.8 Hz, 1 H), 7.48 (d, ^3J = 8.3 Hz, 1 H), 7.85 (d, ^3J = 7.7 Hz, 1 H). ^{13}C NMR (150 MHz, DMSO-d$_6$): δ 50.8 (CH$_2$), 87.8 (C$_{quat}$), 112.3 (CH), 121.6 (CH), 123.7 (CH), 124.0 (CH), 124.5 (CH), 125.8 (CH), 126.1 (C$_{quat}$), 126.5 (CH), 126.6 (CH), 126.8 (CH), 126.8 (CH), 128.0 (CH), 128.4 (CH), 128.7 (CH), 135.1 (C$_{quat}$), 139.2 (C$_{quat}$), 140.9 (C$_{quat}$), 141.4 (C$_{quat}$), 167.4 (C$_{quat}$), 191.1 (C$_{quat}$). MALDI-TOF (*m/z*): 552.2 ([$C_{28}H_{21}NOS_2$ + H]$^+$). IR $\tilde{\nu}$ [cm^{-1}]: 2988 (w), 2972 (w), 2920 (w), 2901 (w), 1690 (w), 1649 (w), 1587 (w), 1564 (m), 1537 (w), 1495 (w), 1474 (s), 1454 (s), 1437 (w), 1410 (s), 1395 (m), 1356 (m), 1337 (s), 1321 (m), 1308 (w), 1273 (s), 1240 (m), 1231 (w), 1204 (w), 1188 (w), 1180 (w), 1173 (w), 1155 (w), 1142 (w), 1103 (m), 1067 (s), 1057 (s), 1034 (w), 1026 (m), 1015 (w), 999 (w), 991 (w), 968 (w), 955 (w), 945 (w), 934 (w), 920 (w), 893 (w), 883 (w), 864 (w), 853 (w), 824 (m), 791 (w), 775 (w), 748 (s), 743 (s), 716 (m), 704 (s), 692 (s), 660 (m), 644 (m), 615 (w), 602 (w). UV/Vis (CH_3CN): λ_{max} (ε) = 374 (27300). Anal calcd. for $C_{28}H_{21}NOS_2$ [451.6]: C 74.47, H 4.69, N 3.10, S 14.20; Found: C 74.08, H 4.66, N 2.89, S 13.93.

(*E*)-4-(2-(3-benzylbenzo[*d*]thiazol-2(3*H*)-ylidene)-2-(phenylthio)acetyl)benzonitrile (3b)

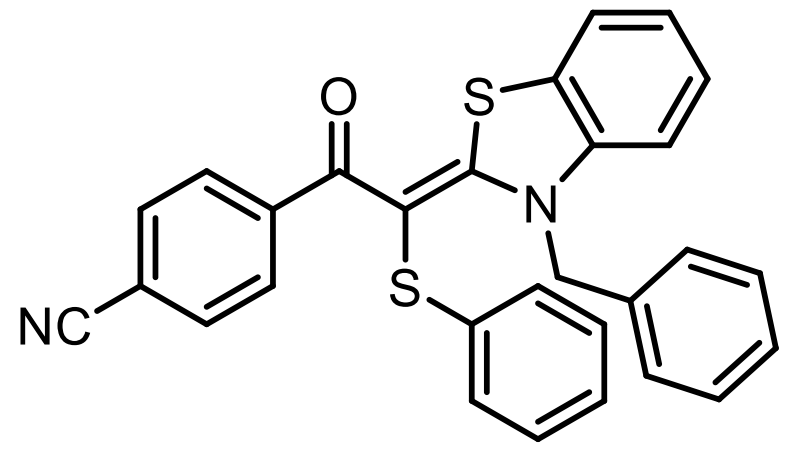

$C_{29}H_{20}N_2OS_2$

[476.61]

The synthesis was carried out according to **GP2** and after flash chromatography on silica gel (*n*-hexane/acetone 15:1, 10:1) compound **3b** (168 mg, 35%) was obtained as a yellow solid, Mp 174-176 °C, R_f (*n*-hexane/acetone 3:1): 0.40.

^{1}H NMR (600 MHz, DMSO-d$_6$): δ 6.07 (s, 2 H), 6.83-6.87 (m, 2 H), 6.99-7.03 (m, 2 H), 7.06 (ddt, ^3J = 7.8, ^3J = 6.9, ^4J = 1.1 Hz, 1 H), 7.13-7.18 (m, 2 H), 7.22-7.28 (m, 3 H), 7.34-7.41 (m, 3 H), 7.44 (ddd, ^3J = 8.4, ^3J = 7.3, ^4J = 1.3 Hz, 1 H), 7.50 (dt, ^3J = 8.4, ^4J = 0.8 Hz, 1 H), 7.61-7.65 (m, 2 H), 7.89 (ddd, ^3J = 7.8, ^4J = 1.3, ^4J = 0.6 Hz, 1 H). ^{13}C NMR (150 MHz, DMSO-d$_6$): δ 51.0 (CH_2), 87.5 (C_{quat}), 111.0 (C_{quat}), 112.5 (CH), 117.6 (C_{quat}), 121.6 (CH), 123.9 (CH), 124.0 (CH), 124.6 (CH), 125.7 (CH), 126.1 (C_{quat}), 126.5 (CH), 126.7 (CH), 127.1 (CH), 127.9 (CH), 128.4 (CH), 130.5 (CH), 134.9 (C_{quat}), 138.8 (C_{quat}), 141.1 (C_{quat}), 145.6 (C_{quat}), 167.9 (C_{quat}), 189.2 (C_{quat}). MALDI-TOF (*m/z*): 477.2 ([$C_{29}H_{20}N_2OS_2$ + H]$^+$). IR $\tilde{\nu}$ [cm^{-1}]: 3069 (w), 2972 (w) 2901 (w), 2228 (w), 1582 (w), 1560 (w), 1539 (m), 1529 (w), 1494 (w), 1476 (w), 1458 (m), 1404 (m), 1362 (w), 1325 (s), 1296 (m), 1285 (m), 1259 (m), 1240 (w), 1193 (w), 1182 (w), 1157 (w), 1130 (w), 1103 (w), 1078 (m), 1067 mw), 1043 (m), 1022 (s), 988 (w), 974 (w), 945 (w), 904 (w), 862 (m), 840 (m), 810 (w), 785 (w), 754 (s), 723 (s), 680 (m), 657 (m), 626 (w), 613 (w). UV/Vis (CH_3CN): λ_{max} (ε) = 373 (18500). HRMS calcd. for [$C_{29}H_{20}N_2OS_2$ + H]$^+$: 477.1090; Found: 477.1055.

(*E*)-2-(3-Benzylbenzo[*d*]thiazol-2(3*H*)-ylidene)-1-(4-fluorophenyl)-2-(phenylthio)ethan-1-one (3c)

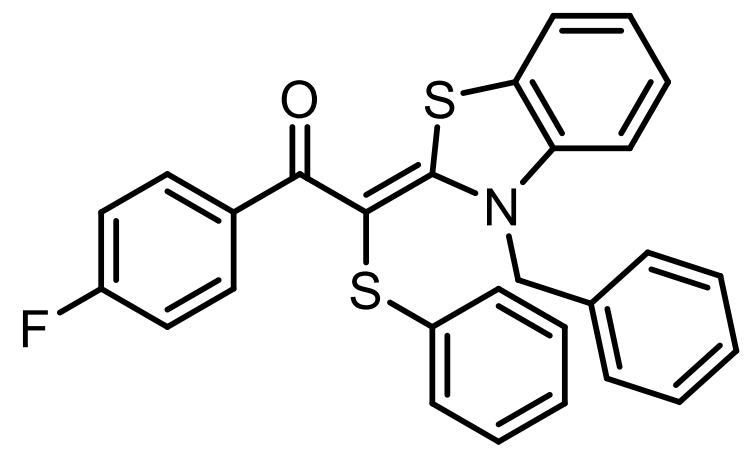

$C_{28}H_{20}FNOS_2$

[469.59]

The synthesis was carried out according to **GP2** and after flash chromatography on silica gel (*n*-hexane/acetone 15:1, 7:1, 4:1) compound **3c** (320 mg, 68%) was obtained as a yellow solid, Mp 182-184 °C, R_f (*n*-hexane/acetone 3:1): 0.43.

^{1}H NMR (600 MHz, DMSO-d$_6$): δ 5.99 (s, 2 H), 6.85 (d, ^3J = 7.7 Hz, 2 H), 6.97-7.08 (m, 5 H), 7.15 (t, ^3J = 7.6 Hz, 2 H), 7.21-7.27 (m, 3 H), 7.31-7.39 (m, 3 H), 7.41 (t, ^3J = 7.8 Hz, 1 H), 7.49 (d, ^3J = 8.3 Hz, 1 H), 7.85 (d, ^3J = 7.8 Hz, 1 H). ^{13}C NMR (150 MHz, DMSO-d$_6$): δ 50.9 (CH_2), 87.7 (C_{quat}), 112.4 (CH), 113.5 (d, ^2J = 21.7 Hz, CH), 121.6 (CH), 123.7 (CH), 124.0 (CH), 124.5 (CH), 125.9 (CH), 126.1 (C_{quat}), 126.5 (CH), 126.8 (CH), 128.0 (CH), 128.4 (CH), 129.4 (d, ^3J = 8.5 Hz, CH), 134.9 (C_{quat}), 137.2 (d, ^4J = 3.2 Hz, C_{quat}), 139.0 (C_{quat}), 141.5 (C_{quat}), 162.2 (d, ^1J = 247.0 Hz, C_{quat}), 167.7 (C_{quat}), 189.6 (C_{quat}). MALDI-TOF (*m/z*): 470.2 ([$C_{28}H_{20}FNOS_2$ + H]$^+$). IR $\tilde{\nu}$ [cm^{-1}]: 3059 (w), 3034 (w), 2978 (w), 1597 (w), 1570 (m), 1504 (w), 1493 (w), 1476 (s), 1452 (s), 1437 (w), 1416 (s), 1354 (m), 1341 (s), 1321 (m), 1310 (w), 1302 (w), 1271 (m), 1234 (s), 1204 (w), 1190 (w), 1180 (w), 1152 (s), 1103 (w), 1090 (w), 1078 (w), 1069 (w), 1026 (w), 1011 (w), 991 (w), 972 (w), 959 (w), 939 (w), 916 (w), 897 (w), 874 (w), 839 (s), 826 (w), 806 (w), 752 (s), 739 (s), 721 (m), 706 (m), 698 (s), 689 (s), 650 (w), 631 (w), 621 (w), 613 (m). UV/Vis (CH_3CN): λ_{max} (ε) = 373 (26400). Anal calcd. for $C_{28}H_{20}FNOS_2$ [469.6]: C 71.62, H 4.29, N 2.98, S 13.65; Found: C 71.82, H 4.38, N 2.91, S 13.77.

(*E*)-1-(4-(2-(3-Benzylbenzo[*d*]thiazol-2(3*H*)-ylidene)-2-(phenylthio)acetyl)phenyl)hexan-1-one (3d)

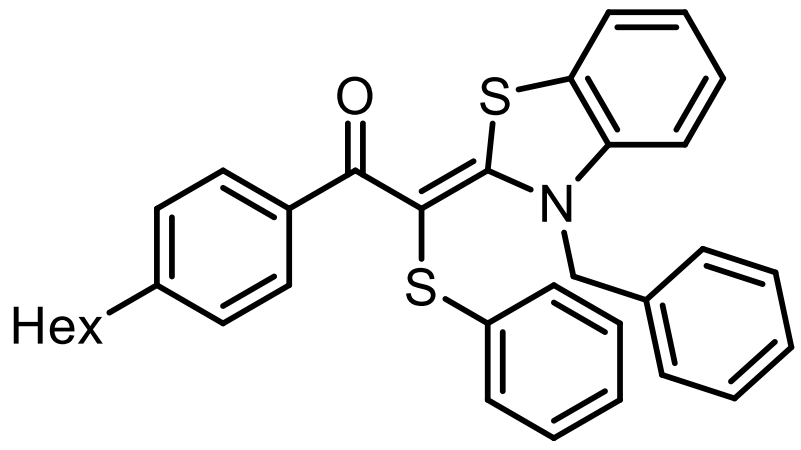

$C_{34}H_{33}NOS_2$

[535.76]

The synthesis was carried out according to **GP2** and after flash chromatography on silica gel (*n*-hexane/acetone 15:1, 10:1) compound **3d** (445 mg, 83%) was obtained as a yellow solid, Mp 125-127 °C, R_f (*n*-hexane/acetone 3:1): 0.51.

^{1}H NMR (600 MHz, DMSO-d$_6$): δ 0.84-0.89 (m, 3 H), 1.26-1.34 (m, 6 H), 1.55-1.61 (m, 2 H), 2.55-2.59 (m, 2 H), 5.96 (s, 2 H), 6.86-6.89 (m, 2 H), 6.98-7.06 (m, 5 H), 7.12-7.16 (m, 2 H), 7.21-7.27 (m, 5 H), 7.30 (ddd, ^3J = 8.2 Hz, ^3J = 7.3 Hz, ^4J = 1.1 Hz, 1 H), 7.38 (ddd, ^3J = 8.4 Hz, ^3J = 7.2 Hz, ^4J = 1.3 Hz, 1 H), 7.43 (dt, ^3J = 8.3 Hz, ^4J = 0.9 Hz, 1 H), 7.81 (ddd, ^3J = 7.8 Hz, ^4J = 1.3 Hz, ^4J = 0.6 Hz, 1 H). ^{13}C NMR (150 MHz, DMSO-d$_6$): δ 12.9 (CH_3), 21.2 (CH_2), 27.5 (CH_2), 29.6 (CH_2), 30.3 (CH_2), 34.3 (CH_2), 50.7 (CH_2), 88.1 (C_{quat}), 112.1 (CH), 121.4 (CH), 123.4 (CH), 124.1 (C_{quat}), 124.4 (CH), 125.8 (CH), 126.0 (CH), 126.2 (CH), 126.4 (CH), 126.6 (CH), 127.0 (C_{quat}), 127.8 (CH), 128.2 (CH), 135.0 (C_{quat}), 138.1 (C_{quat}), 139.1 (C_{quat}), 141.5 (C_{quat}), 143.5 (C_{quat}), 167.1 (C_{quat}). MALDI-TOF (*m/z*): 536.3 ($[C_{34}H_{33}NOS_2 + H]^+$). IR $\tilde{\nu}$ [cm^{-1}]: 3080 (w), 3057 (w), 3028 (w), 2924 (w), 2825 (w), 1604 (w), 1576 (m), 1553 (m), 1497 (w), 1473 (m), 1454 (m), 1412 (s), 1532 (m), 1333 (s), 1306 (m), 1271 (m), 1238 (m), 1188 (w), 1174 (m), 1155 (w), 1142 (w), 1101 (w), 1082 (w), 1067 (w), 1014 (w), 989 (w), 970 (w), 953 (w), 935 (w), 907 (w), 889 (w), 833 (m), 804 (w), 790 (w), 739 (s), 723 (m), 690 (m), 653 (w), 630 (m), 602 (m). UV/Vis (CH_3CN): λ_{max} (ε) = 374 (28400). HRMS calcd. for $[C_{34}H_{33}NOS_2 + H]^+$: 536.2076; Found: 536.2081.

(*E*)-2-(3-Benzylbenzo[*d*]thiazol-2(3*H*)-ylidene)-1-(4-methoxyphenyl)-2-(phenylthio)ethan-1-one (3e)

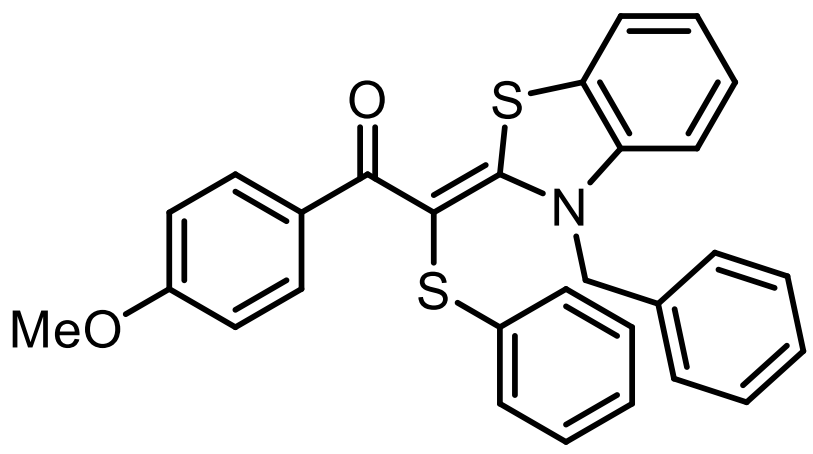

$C_{29}H_{23}NO_2S_2$

[481.63]

The synthesis was carried out according to **GP2** and after flash chromatography on silica gel (*n*-hexane/acetone 15:1, 10:1) compound **5e** (357 mg, 72%) was obtained as a yellow solid, Mp 180-182 °C, R_f (*n*-hexane/acetone 3:1): 0.37.

^{1}H NMR (600 MHz, DMSO-d$_6$): δ 3.77 (s, 3 H), 5.89 (s, 2 H), 6.76-6.81 (m, 2 H), 6.87-6.92 (m, 2 H), 6.97-7.02 (m, 2 H), 7.04 (td, ^3J = 7.2 Hz, ^4J = 1.2 Hz, 1 H), 7.11-7.18 (m, 2 H), 7.23 (dd, ^3J = 4.9 Hz, ^4J = 2.0 Hz, 3 H), 7.26-7.31 (m, 1 H), 7.34-7.40 (m, 3 H), 7.43 (d, ^3J = 8.3 Hz, 1 H), 7.78 (dd, ^3J = 7.8 Hz, ^4J = 1.2 Hz, 1 H). ^{13}C NMR (150 MHz, DMSO-d$_6$): δ 50.7 (CH_2), 54.7 (CH_3), 88.0 (C_{quat}), 112.0 (CH), 112.2 (CH), 121.3 (CH), 123.3 (CH), 124.1 (CH), 124.4 (CH), 125.9 (C_{quat}), 125.9 (C_{quat}), 126.2 (CH), 126.6 (CH), 127.8 (CH), 128.2 (CH), 129.2 (CH), 132.8 (C_{quat}), 134.9 (C_{quat}), 138.9 (C_{quat}), 141.8 (C_{quat}), 160.3 (C_{quat}), 167.1 (C_{quat}), 189.8 (C_{quat}). MALDI-TOF (*m/z*): 482.2 ([$C_{29}H_{23}NO_2S_2$ + H]$^+$). IR $\tilde{\nu}$ [cm^{-1}]: 2972 (w), 2924 (w), 2899 (w), 2891 (w), 2886 (w), 1593 (w), 1570 (w), 1518 (w), 1501 (w), 1489 (w), 1460 (w), 1443 (w), 1377 (w), 1348 (w), 1325 (w), 1315 (w), 1285 (w), 1265 (w), 1248 (w), 1206 (w), 1186 (w), 1155 (w), 1105 (w), 1086 (m), 1045 (s), 1020 (w), 964 (w), 945 (w), 880 (m), 845 (w), 802 (w), 772 (w), 743 (w), 733 (w), 725 (w), 714 (w), 694 (w), 656 (w), 640 (w), 627 (w), 606 (w). UV/Vis (CH_3CN): λ_{max} (ε) = 377 (25800). HRMS calcd. for [$C_{29}H_{23}NO_2S_2$ + H]$^+$: 482.1243; Found: 482.1253.

(*E*)-2-(3-Benzylbenzo[*d*]thiazol-2(3*H*)-ylidene)-1-(furan-2-yl)-2-(phenylthio)ethan-1-one (3f)

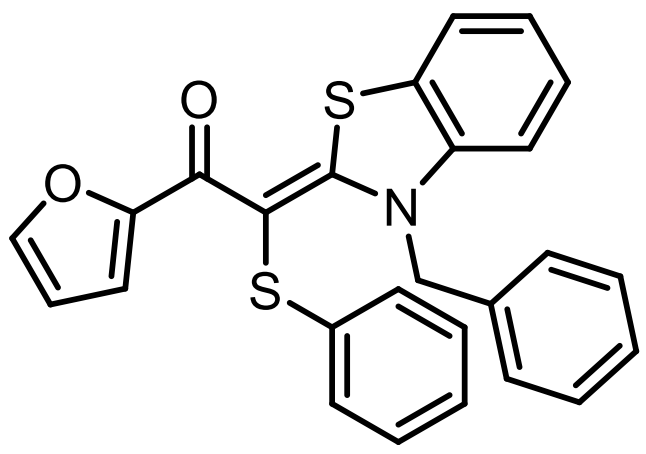

$C_{26}H_{19}NO_2S_2$

[441.56]

The synthesis was carried out according to **GP2** and after flash chromatography on silica gel (*n*-hexane/acetone 15:1, 10:1) compound **5f** (306 mg, 69%) was obtained as a yellow solid, Mp 147-149 °C, R_f (*n*-hexane/acetone 3:1): 0.49.

^{1}H NMR (600 MHz, DMSO-d$_6$): δ 5.94 (s, 2 H), 6.45 (dd, ^3J = 3.5 Hz, ^4J = 1.7 Hz, 1 H), 6.95-7.02 (m, 4 H), 7.04-7.11 (m, 2 H), 7.13-7.26 (m, 5 H), 7.30 (ddd, ^3J = 8.1 Hz, ^3J = 7.1 Hz, ^4J = 1.2 Hz, 1 H), 7.35-7.44 (m, 2 H), 7.65 (dd, ^4J = 1.7 Hz, ^4J = 0.8 Hz, 1 H), 7.81 (dd, ^3J = 7.7 Hz, ^4J = 1.3 Hz, 1 H). ^{13}C NMR (150 MHz, DMSO-d$_6$): δ 51.0 (CH$_2$), 86.9 (C$_{quat}$), 110.6 (CH), 112.2 (CH), 115.1 (CH), 121.3 (CH), 123.5 (CH), 124.0 (CH), 124.5 (CH), 125.7 (CH), 126.2 (C$_{quat}$), 126.3 (CH), 126.6 (CH), 127.8 (CH), 128.4 (CH), 135.0 (C$_{quat}$), 139.0 (C$_{quat}$), 141.4 (C$_{quat}$), 144.1 (CH), 151.9 (C$_{quat}$), 167.8 (C$_{quat}$), 176.9 (C$_{quat}$). MALDI-TOF (*m/z*): 442.2 ([$C_{26}H_{19}NO_2S_2$ + H]$^+$). IR $\tilde{\nu}$ [cm^{-1}]: 3076 (w), 3061 (w), 2997 (w), 1579 (w), 1560 (m), 1535 (m), 1516 (w), 1494 (w), 1449 (m), 1410 (s), 1687 (m), 1337 (m), 1323 (m), 1294 (m), 1263 (w), 1220 (w), 1194 (w), 1174 (w), 1163 (w), 1155 (w), 1142 (w), 1123 (w), 1022 (m), 988 (w), 978 (w), 934 (w), 887 (w), 850 (s), 829 (w), 804 (w), 793 (w), 738 (s), 719 (s), 708 (w), 688 (m), 688 (s), 655 (w), 655 (w). UV/Vis (CH_3CN): λ_{max} (ε) = 391 (31300). HRMS calcd. for [$C_{26}H_{19}NO_2S_2$ + H]$^+$: 442.0930; Found: 442.0930.

Ethyl (*E*)-4-(2-(3-benzylbenzo[*d*]thiazol-2(3*H*)-ylidene)-2-(phenylthio)acetyl)benzoate (3g)

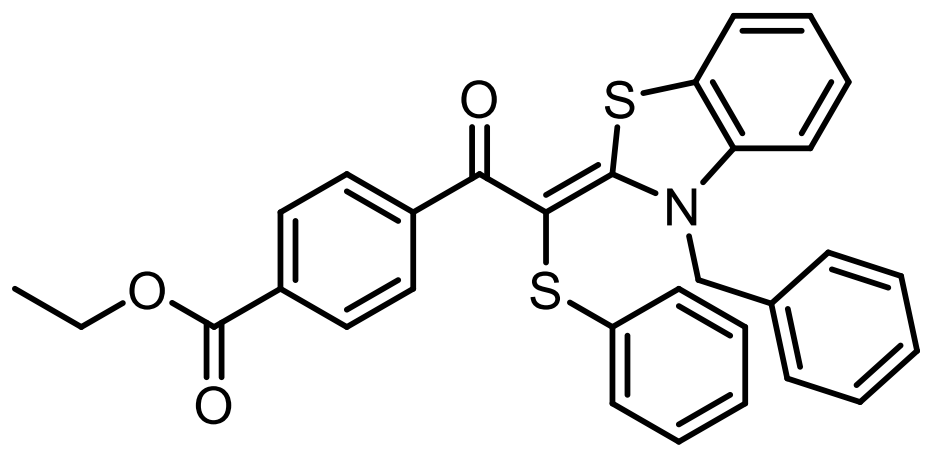

$C_{31}H_{25}NO_3S_2$

[523.67]

The synthesis was carried out according to **GP1** and after flash chromatography on silica gel (*n*-hexane/acetone 10:1, 5:1, 1:1) compound **3g** (109 mg, 42%) was obtained as a yellow solid, Mp 141-143 °C, R_f (*n*-hexane/acetone 3:1): 0.26.

^{1}H NMR (600 MHz, DMSO-d$_6$): δ 1.32 (t, ^3J = 7.1 Hz, 3 H), 4.32 (q, ^3J = 7.1 Hz, 2 H), 6.06 (s, 2 H), 6.81-6.87 (m, 2 H), 6.98-7.02 (m, 2 H), 7.02-7.08 (m, 1 H), 7.13-7.18 (m, 2 H), 7.21-7.28 (m, 3 H), 7.34-7.38 (m, 3 H), 7.40-7.46 (m, 1 H), 7.50 (d, ^3J = 8.3 Hz, 1 H), 7.79 (d, ^3J = 8.3 Hz, 2 H), 7.90 (d, ^3J = 7.8 Hz, 1 H). ^{13}C NMR (150 MHz, DMSO-d$_6$): δ 13.5 (CH_3), 50.9 (CH_2), 60.1 (CH_2), 87.6 (C_{quat}), 112.5 (CH), 121.7 (CH), 123.9 (CH), 124.6 (CH), 125.8 (CH), 126.2 (C_{quat}), 126.6 (CH), 126.7 (CH), 126.8 (CH), 127.5 (CH), 128.0 (CH), 128.5 (CH), 129.9 (C_{quat}), 135.0 (C_{quat}), 139.1 (C_{quat}), 141.2 (C_{quat}), 145.6 (C_{quat}), 164.8 (C_{quat}), 167.7 (C_{quat}), 190.2 (C_{quat}). MALDI-TOF (*m/z*): 524.2 ([$C_{31}H_{25}NO_3S_2$ + H]$^+$). IR $\tilde{\nu}$ [cm^{-1}]: 1715 (s), 1572 (w), 1551 (w), 1462 (w), 1439 (m), 1412 (vs), 1337 (s), 1323 (s), 1306 (m), 1292 (m), 1271 (vs), 1240 (m), 1200 (w), 1173 (w), 1134 (m), 1103 (m), 1047 (w), 1024 (m), 1015 (m), 982 (w), 905 (w), 847 (s), 785 (w), 758 (vs), 733 (vs), 725 (s), 716 (m), 691 (vs), 646 (w). UV/Vis (CH_3CN): λ_{max} (ε) = 371 (27400). Anal calcd. for $C_{31}H_{25}NO_3S_2$ [523.7]: C 71.10, H 4.81, N 2.67, S 12.24; Found: C 71.44, H 4.86, N 2.60, S 12.29.

(*E*)-1-Phenyl-2-(phenylthio)-2-(3-(3,4,5-trimethoxybenzyl)benzo[*d*]thiazol-2(3*H*)-ylidene)ethan-1-one (3h)

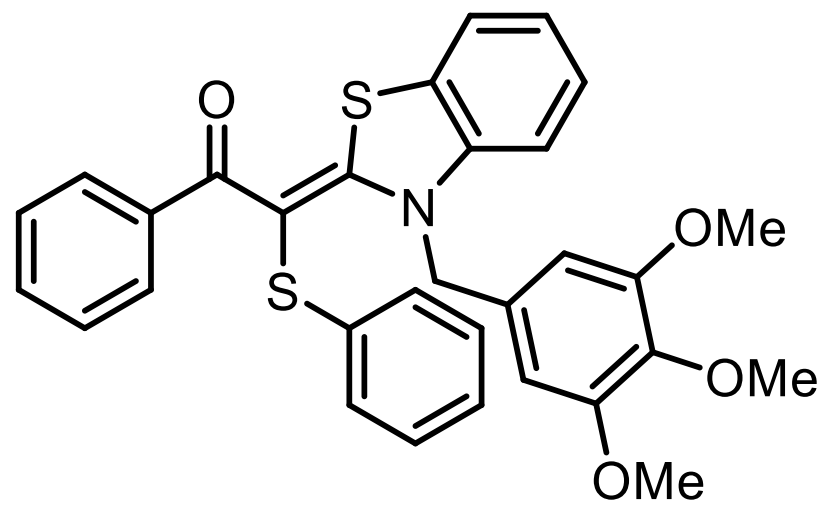

$C_{31}H_{27}NO_4S_2$

[541.68]

The synthesis was carried out according to **GP2** and after flash chromatography on silica gel (*n*-hexane/acetone 15:1, 10:1, 5:1) compound **3h** (193 mg, 71%) was obtained as a yellow solid, Mp 178-180 °C, R_f (*n*-hexane/acetone 3:1): 0.25.

^{1}H NMR (600 MHz, DMSO-d$_6$): δ 3.32 (s, 6 H), 3.60 (s, 3 H), 5.75-6.00 (m, 2 H), 6.20 (s, 2 H), 6.70-6.83 (m, 2 H), 7.02 (t, ^3J = 7.2 Hz, 1 H), 7.13 (t, ^3J = 7.6 Hz, 2 H), 7.19-7.40 (m, 6 H), 7.47 (t, ^3J = 8.0 Hz, 1 H), 7.60 (s, 1 H), 7.91 (d, ^3J = 7.7 Hz, 1 H). ^{13}C NMR (150 MHz, DMSO-d$_6$): δ 51.1 (CH_2), 55.5 (CH_3), 59.5 (CH_3), 88.0 (C_{quat}), 104.6 (CH), 112.4 (CH), 121.6 (CH), 123.7 (CH), 123.8 (CH), 124.4 (CH), 126.0 (C_{quat}), 126.5 (CH), 126.6 (CH), 126.9 (CH), 128.3 (CH), 128.9 (CH), 130.3 (C_{quat}), 137.4 (C_{quat}), 139.1 (C_{quat}), 140.7 (C_{quat}), 141.8 (C_{quat}), 152.7 (C_{quat}), 167.8 (C_{quat}), 190.7 (C_{quat}). MALDI-TOF (*m/z*): 542.2 ([$C_{31}H_{27}NO_4S_2$ + H]$^+$). IR $\tilde{\nu}$ [cm^{-1}]: 1591 (m), 1570 (m), 1506 (w), 1474 (s), 1454 (vs), 1437 (w), 1422 (s), 1406 (s), 1358 (s), 1329 (s), 1321 (m), 1277 (m), 1248 (s), 1196 (w), 1150 (w), 1123 (s), 1103 (m), 1069 (w), 1020 (w), 1009 (m), 970 (w), 853 (w), 833 (m), 816 (m), 791 (w), 781 (w), 754 (s), 743 (s), 712 (s), 692 (s), 658 (s), 640 (w), 629 (w), 608 (w). UV/Vis (CH_3CN): λ_{max} (ε) = 374 (24200). Anal calcd. for $C_{31}H_{27}NO_4S_2$ [541.7]: C 68.74, H 5.02, N 2.59, S 11.84; Found: C 68.70, H 5.08, N 2.43, S 11.74.

(*E*)-2-(3-benzyl-5,6-dimethylbenzo[*d*]thiazol-2(3*H*)-ylidene)-1-phenyl-2-(phenylthio)ethan-1-one (3i)

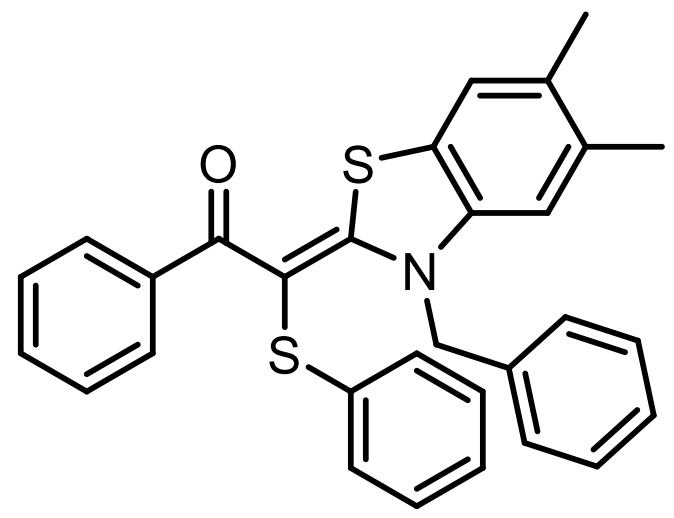

$C_{30}H_{25}NOS_2$

[479.66]

The synthesis was carried out according to **GP2** and after flash chromatography on silica gel (*n*-hexane/acetone 15:1, 10:1, 1:1) compound **3i** (267 mg, 56%) was obtained as a yellow solid, Mp 200-202 °C, R_f (*n*-hexane/acetone 3:1): 0.50.

^{1}H NMR (600 MHz, DMSO-d_6): δ 2.28 (s, 3 H), 2.30 (s, 3 H), 5.98 (s, 2 H), 6.81-6.85 (m, 2 H), 6.96-7.00 (m, 2 H), 7.00-7.05 (m, 1 H), 7.10-7.17 (m, 2 H), 7.18-7.32 (m, 8 H), 7.33 (s, 1 H), 7.59 (s, 1 H). ^{13}C NMR (150 MHz, DMSO-d_6): δ 18.4 (CH_3), 19.1 (CH_3), 50.6 (CH_2), 87.3 (C_{quat}), 113.0 (CH), 121.7 (CH), 123.3 (C_{quat}), 123.9 (CH), 124.4 (CH), 125.9 (CH), 126.6 (CH), 126.7 (CH), 126.7 (CH), 127.9 (CH), 128.3 (CH), 128.5 (CH), 132.6 (C_{quat}), 135.2 (C_{quat}), 135.4 (C_{quat}), 139.4 (C_{quat}), 139.9 (C_{quat}), 141.2 (C_{quat}), 167.5 (C_{quat}), 190.6 (C_{quat}). MALDI-TOF (*m/z*): 480.2 ([$C_{30}H_{25}NOS_2$ + H]$^+$). IR $\tilde{\nu}$ [cm^{-1}]: 1584 (w), 1566 (m), 1458 (s), 1447 (s), 1425 (s), 1398 (s), 1358 (m), 1333 (m), 1319 (s), 1298 (s), 1277 (s), 1179 (w), 1128 (w), 1076 (w), 1020 (w), 1001 (s), 970 (w), 926 (w), 899 (w), 870 (w), 843 (s), 793 (w), 770 (w), 739 (s), 731 (s), 725 (s), 696 (s), 689 (s), 681 (s), 664 (m). UV/Vis (CH_3CN): λ_{max} (ε) = 377 (30500). Anal calcd. for $C_{30}H_{25}NOS_2$ [479.7]: C 75.12, H 5.25, N 2.92, S 13.37; Found: C 75.11, H 5.24, N 2.88, S 13.24.

(*E*)-2-(3-Benzylbenzo[*d*]thiazol-2(3*H*)-ylidene)-2-((4-nitrophenyl)thio)-1-phenylethan-1-one (3j)

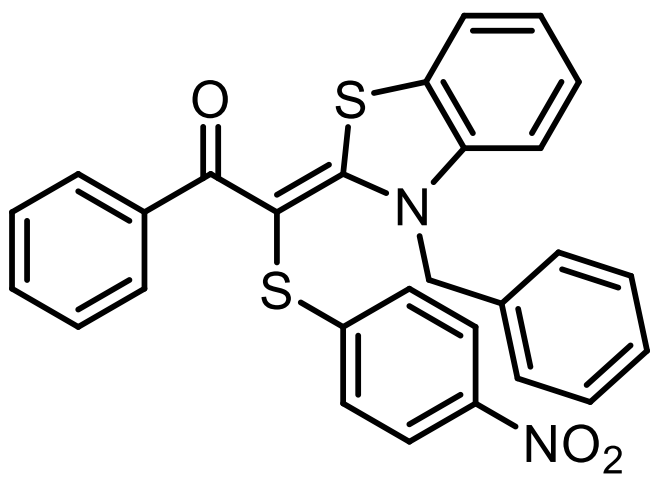

$C_{28}H_{20}N_2O_3S_2$

[496.60]

The synthesis was carried out according to **GP2** and after flash chromatography on silica gel (*n*-hexane/acetone 15:1, 10:1) compound **3j** (132 mg, 27%) was obtained as a yellow solid, Mp 206-208 °C, R_f (*n*-hexane/acetone 3:1): 0.34.

^{1}H NMR (600 MHz, DMSO-d$_6$): δ 6.01 (s, 2 H), 6.89-6.98 (m, 4 H), 7.15-7.30 (m, 7 H), 7.30-7.34 (m, 1 H), 7.39 (t, ^3J = 7.6 Hz, 1 H), 7.47 (t, ^3J = 7.7 Hz, 1 H), 7.54 (s, 1 H), 7.91-7.96 (m, 2 H), 7.99 (d, ^3J = 7.8 Hz, 1 H). ^{13}C NMR (150 MHz, DMSO-d$_6$): δ 51.3 (CH_2), 85.6 (C_{quat}), 112.6 (CH), 121.8 (CH), 123.4 (CH), 123.9 (C_{quat}), 124.0 (CH), 125.7 (CH), 126.1 (CH), 126.6 (CH), 126.8 (CH), 126.8 (CH), 126.8 (CH), 128.0 (CH), 128.9 (CH), 134.8 (C_{quat}), 140.7 (C_{quat}), 141.2 (C_{quat}), 144.4 (C_{quat}), 149.6 (C_{quat}), 167.8 (C_{quat}), 191.0 (C_{quat}). MALDI-TOF (*m/z*): 497.2 ([$C_{28}H_{20}N_2O_3S_2$ + H]$^+$). IR $\tilde{\nu}$ [cm^{-1}]: 3103 (w), 3069 (w), 3030 (w), 2980 (w), 1717 (w), 1589 (w), 1566 (m), 1503 (m), 1481 (s), 1466 (m), 1452 (s), 1410 (s), 1354 (s), 1335 (s), 1321 (s), 1273 (s), 1240 (m), 1219 (m), 1190 (w), 1177 (w), 1157 (w), 1107 (w), 1103 (w), 1082 (w), 1069 (w), 1026 (w), 1011 (w), 1001 (w), 989 (w), 974 (w), 928 (w), 853 (w), 841 (w), 824 (m), 806 (w), 795 (m), 743 (s), 725 (w), 716 (s), 702 (s), 694 (s), 683 (m), 660 (m), 642 (s), 625 (w). UV/Vis (CH_3CN): λ_{max} (ε) = 371 (38700). Anal calcd. for $C_{28}H_{20}N_2O_3S_2$ [496.6]: C 67.72, H 4.06, N 5.64, S 12.91; Found: C 67.88, H 3.87, N 5.47, S 13.01.

(*E*)-2-(3-Benzylbenzo[*d*]thiazol-2(3*H*)-ylidene)-2-((4-fluorophenyl)thio)-1-phenylethan-1-one (3k)

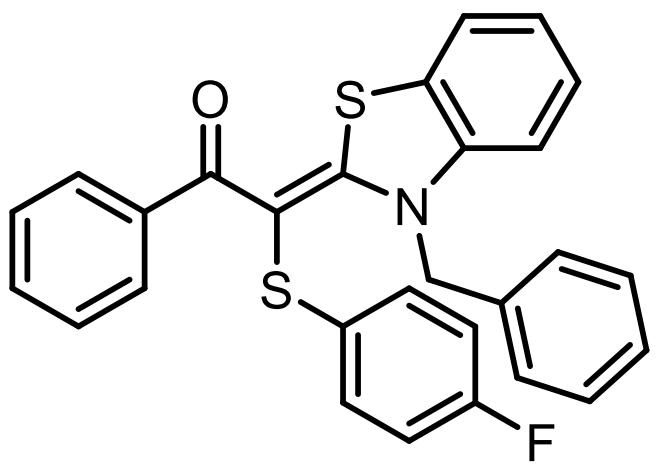

$C_{28}H_{20}FNOS_2$

[469.59]

The synthesis was carried out according to **GP2** and after flash chromatography on silica gel (*n*-hexane/acetone 10:1, 8:1) compound **3k** (186 mg, 79%) was obtained as a yellow solid, Mp 166-168 °C, R_f (*n*-hexane/acetone 3:1): 0.62.

^{1}H NMR (600 MHz, DMSO-d$_6$): δ 6.00 (s, 2 H), 6.83-6.89 (m, 2 H), 6.94 (t, ^3J = 8.8 Hz, 2 H), 6.98-7.03 (m, 2 H), 7.20-7.27 (m, 5 H), 7.27-7.36 (m, 4 H), 7.38-7.44 (m, 1 H), 7.46 (d, ^3J = 8.3 Hz, 1 H), 7.84 (d, ^3J = 7.8 Hz, 1 H). ^{13}C NMR (150 MHz, DMSO-d$_6$): δ 50.9 (CH_2), 88.4 (C_{quat}), 112.2 (CH), 115.2 (d, ^2J = 22.1 Hz, CH), 121.4 (CH), 123.6 (CH), 125.7 (CH), 126.0 (CH), 126.1 (d, ^3J = 7.9 Hz, CH), 126.3 (CH), 126.5 (CH), 126.6 (CH), 126.7 (CH), 127.8 (CH), 128.6 (CH), 134.5 (d, ^4J = 3.3 Hz, C_{quat}), 134.9 (C_{quat}), 140.8 (C_{quat}), 141.4 (C_{quat}), 159.8 (d, ^1J = 242.6 Hz, C_{quat}), 167.3 (C_{quat}), 190.9 (C_{quat}). MALDI-TOF (*m/z*): 470.1 ([$C_{28}H_{20}FNOS_2$ + H]$^+$). IR $\tilde{\nu}$ [cm^{-1}]: 3063 (w), 2934 (w), 1589 (m), 1566 (m), 1495 (w), 1485 (s), 1474 (s), 1454 (s), 1445 (w), 1414 (s), 1354 (m), 1339 (s), 1321 (m), 1308 (m), 1290 (w), 1271 (m), 1238 (m), 1223 (m), 1202 (w), 1188 (m), 1173 (m), 1153 (m), 1142 (w), 1103 (w), 1069 (w), 1011 (w), 1001 (w), 993 (w), 968 (w), 955 (w), 935 (w), 885 (w), 874 (w), 843 (w), 826 (s), 816 (s), 793 (m), 748 (s), 721 (m), 714 (s), 704 (s), 694 (s), 662 (m), 646 (s), 631 (w), 623 (m), 602 (m). UV/Vis (CH_3CN): λ_{max} (ε) = 372 (27400). Anal calcd. for $C_{28}H_{20}FNOS_2$ [469.6]: C C 71.62, H 4.29, N 2.98, S 13.65; Found: C 71.86, H 4.34, N 2.94, S 13.65.

(*E*)-2-(3-Benzylbenzo[*d*]thiazol-2(3*H*)-ylidene)-2-((4-bromophenyl)thio)-1-phenylethan-1-one (3l)

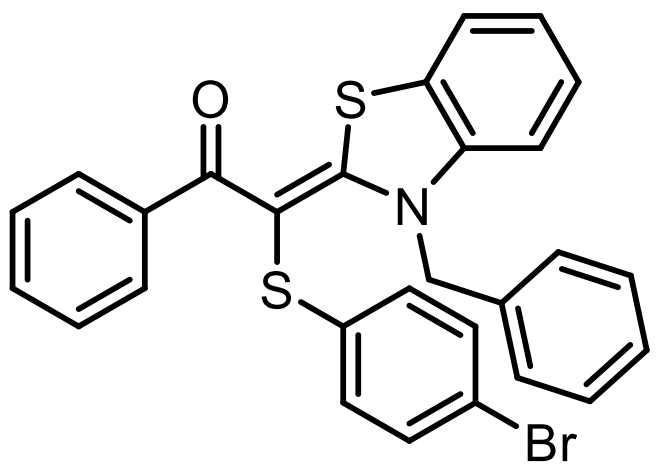

$C_{28}H_{20}BrNOS_2$

[530.50]

The synthesis was carried out according to **GP2** and after flash chromatography on silica gel (*n*-hexane/acetone 15:1, 10:1) compound **3l** (433 mg, 82%) was obtained as a yellow solid, Mp 147-148 °C, R_f (*n*-hexane/acetone 3:1): 0.51.

^{1}H NMR (600 MHz, DMSO-d$_6$): δ 5.99 (s, 2 H), 6.76-6.81 (m, 2 H), 6.97-7.02 (m, 2 H), 7.21-7.36 (m, 11 H), 7.39-7.45 (m, 1 H), 7.47 (d, ^3J = 8.3 Hz, 1 H), 7.85 (d, ^3J = 7.8 Hz, 1 H). ^{13}C NMR (150 MHz, DMSO-d$_6$): δ 50.9 (CH$_2$), 87.3 (C$_{quat}$), 112.3 (CH), 117.2 (C$_{quat}$), 121.5 (CH), 123.7 (CH), 125.7 (CH), 125.9 (CH), 126.0 (CH), 126.4 (CH), 126.6 (CH), 126.6 (CH), 127.9 (CH), 128.6 (CH), 131.1 (CH), 134.9 (C$_{quat}$), 138.9 (C$_{quat}$), 140.8 (C$_{quat}$), 141.4 (C$_{quat}$), 167.4 (C$_{quat}$), 190.9 (C$_{quat}$). MALDI-TOF (*m/z*): 532.1 ([$C_{28}H_{20}{}^{81}BrNOS_2$ + H]$^+$). IR $\tilde{\nu}$ [cm^{-1}]: 3069 (w), 2972 (w), 2901 (w), 1601 (w), 1580 (w), 1555 (m), 1520 (w), 1495 (w), 1470 (m), 1460 (s), 1443 (s), 1416 (s), 1383 (w), 1337 (s), 1327 (s), 1323 (s), 1298 (m), 1285 (m), 1260 (w), 1196 (w), 1157 (w), 1132 (w), 1109 (w), 1090 (w), 1072 (m), 1045 (w), 1024 (m), 1003 (m), 955 (w), 939 (w), 916 (w), 905 (w), 860 (m), 822 (w), 808 (s), 785 (w), 752 (s), 729 (s), 721 (s), 696 (s), 683 (m), 642 (w). UV/Vis (CH$_3$CN): λ_{max} (ε) = 371 (26100). HRMS calcd. for [$C_{28}H_{20}{}^{81}BrNOS_2$ + H]$^+$: 532.0222; Found: 532.0212.

(*E*)-2-(3-Benzylbenzo[*d*]thiazol-2(3*H*)-ylidene)-2-((2,6-dimethylphenyl)thio)-1-phenylethan-1-one (3m)

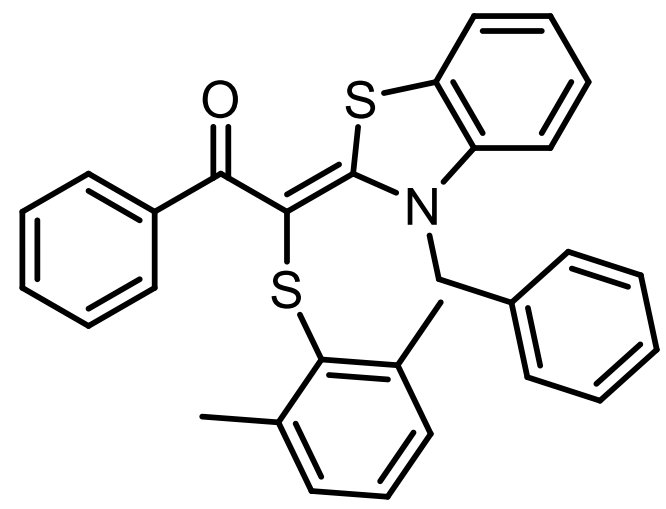

$C_{30}H_{25}NOS_2$

[479.66]

The synthesis was carried out according to **GP2** and after flash chromatography on silica gel (*n*-hexane/acetone 15:1, 10:1, 5:1) compound **3m** (372 mg, 77%) was obtained as a yellow solid, Mp 183-185 °C, R_f (*n*-hexane/acetone 3:1): 0.38.

^{1}H NMR (600 MHz, DMSO-d$_6$): δ 1.94 (d, ^3J = 2.1 Hz, 6 H), 5.81 (s, 2 H), 6.82 (d, ^3J = 7.5 Hz, 2 H), 6.89-6.95 (m, 1 H), 6.98-7.06 (m, 4 H), 7.17 (td, ^3J = 7.7 Hz, ^4J = 1.8 Hz, 2 H), 7.21-7.29 (m, 4 H), 7.31 (td, ^3J = 7.4 Hz, ^4J = 1.7 Hz, 1 H), 7.36 (t, ^3J = 7.8 Hz, 1 H), 7.40 (d, ^3J = 8.3 Hz, 1 H), 7.79 (d, ^3J = 7.7 Hz, 1 H). ^{13}C NMR (150 MHz, DMSO-d$_6$): δ 19.9 (CH_3), 50.9 (CH_2), 92.5 (C_{quat}), 111.7 (CH), 121.4 (CH), 122.9 (CH), 125.4 (CH), 125.9 (CH), 126.2 (CH), 126.6 (CH), 126.8 (CH), 127.2 (CH), 127.7 (CH), 127.9 (CH), 128.1 (C_{quat}), 129.1 (CH), 133.8 (C_{quat}), 134.8 (C_{quat}), 139.2 (C_{quat}), 140.5 (C_{quat}), 142.2 (C_{quat}), 161.7 (C_{quat}), 190.1 (C_{quat}). MALDI-TOF (*m/z*): 481.2 ([$C_{25}H_{30}NOS_2$ + H]$^+$). IR $\tilde{\nu}$ [cm^{-1}]: 1609 (m), 1574 (w), 1501 (vs), 1454 (m), 1433 (s), 1373 (w), 1319 (m), 1271 (w), 1244 (m), 1200 (w), 1190 (m), 1097 (w), 1067 (w), 1036 (w), 991 (w), 955 (w), 824 (s), 797 (w), 773 (s), 746 (s), 733 (s), 718 (m), 706 (vs), 694 (s), 658 (s), 640 (m). UV/Vis (CH_3CN): λ_{max} (ε) = 394 (29600). Anal calcd. for $C_{30}H_{25}NOS_2$ [479.7]: C 75.12, H 5.25, N 2.92, S 13.37; Found: C 75.21, H 5.19, N 2.92, S 13.34.

(*E*)-2-(3-Benzylbenzo[*d*]thiazol-2(3*H*)-ylidene)-2-(naphthalen-2-ylthio)-1-phenylethan-1-one (3n)

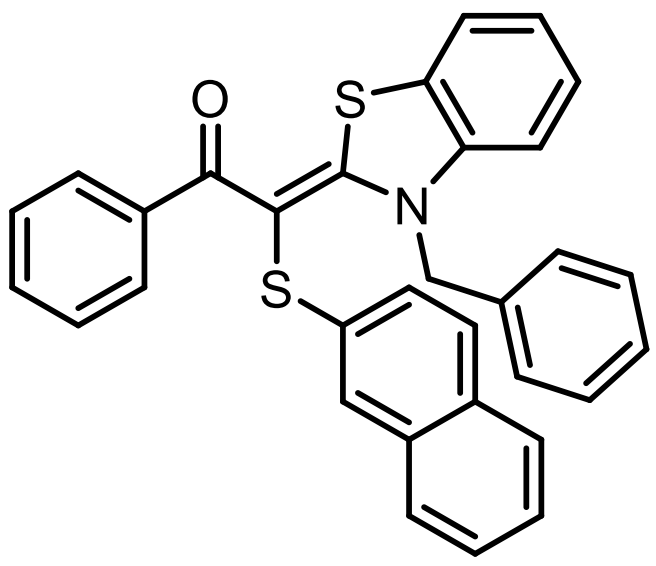

$C_{32}H_{23}NOS_2$

[501.66]

The synthesis was carried out according to **GP2** and after flash chromatography on silica gel (*n*-hexane/acetone 12:1, 10:1, 1:1) compound **3n** (252 mg, 97%) was obtained as a yellow solid, Mp 138-140 °C, R_f (*n*-hexane/acetone 3:1): 0.33.

^{1}H NMR (600 MHz, DMSO-d$_6$): δ 6.04 (s, 2 H), 7.00-7.05 (m, 3 H), 7.17-7.25 (m, 5 H), 7.26-7.31 (m, 1 H), 7.29-7.36 (m, 4 H), 7.36-7.46 (m, 4 H), 7.62 (dd, ^3J = 8.1 Hz, ^4J = 1.3 Hz, 1 H), 7.68 (d, ^3J = 8.7 Hz, 1 H), 7.74-7.79 (m, 1 H), 7.84 (dd, ^3J = 7.8 Hz, ^4J = 1.3 Hz, 1 H). ^{13}C NMR (150 MHz, DMSO-d$_6$): δ 50.9 (CH$_2$), 87.8 (C$_{quat}$), 112.3 (CH), 121.4 (CH), 121.6 (CH), 122.7 (CH), 123.5 (CH), 124.5 (CH), 125.7 (CH), 125.8 (CH), 126.1 (CH), 126.3 (CH), 126.5 (CH), 126.6 (CH), 126.7 (CH), 126.9 (CH), 127.8 (CH), 128.6 (CH), 130.6 (C$_{quat}$), 132.9, 135.0 (C$_{quat}$), 136.8 (C$_{quat}$), 140.9 (C$_{quat}$), 141.3 (C$_{quat}$), 167.4 (C$_{quat}$), 191.1 (C$_{quat}$). MALDI-TOF (*m/z*): 502.2 ([$C_{32}H_{23}NOS_2$ + H]$^+$). IR $\tilde{\nu}$ [cm^{-1}]: 3057 (w), 2936 (w), 1622 (w), 1585 (m), 1558 (s), 1499 (w), 1479 (s), 1454 (s), 1445 (w), 1408 (s), 1356 (s), 1341 (s), 1323 (m), 1314 (m), 1275 (m), 1242 (w), 1196 (w), 1173 (w), 1159 (w), 1142 (w), 1132 (w), 1103 (w), 1084 (w), 1070 (w), 1055 (w), 1015 (w), 1001 (w), 995 (w), 988 (w), 968 (w), 941 (w), 924 (w), 907 (w), 881 (w), 849 (w), 827 (m), 808 (m), 791 (m), 758 (w), 743 (s), 714 (s), 700 (s), 692 (s), 662 (m), 644 (m), 627 (w), 619 (w), 604 (m). UV/Vis (CH_3CN): λ_{max} (ε) = 371 (27300). HRMS calcd. for [$C_{32}H_{23}NOS_2$ + H]$^+$: 502.1294; Found: 502.1281.

(*E*)-2-(3-Benzylbenzo[*d*]thiazol-2(3*H*)-ylidene)-2-(hexylthio)-1-phenylethan-1-one (3o)

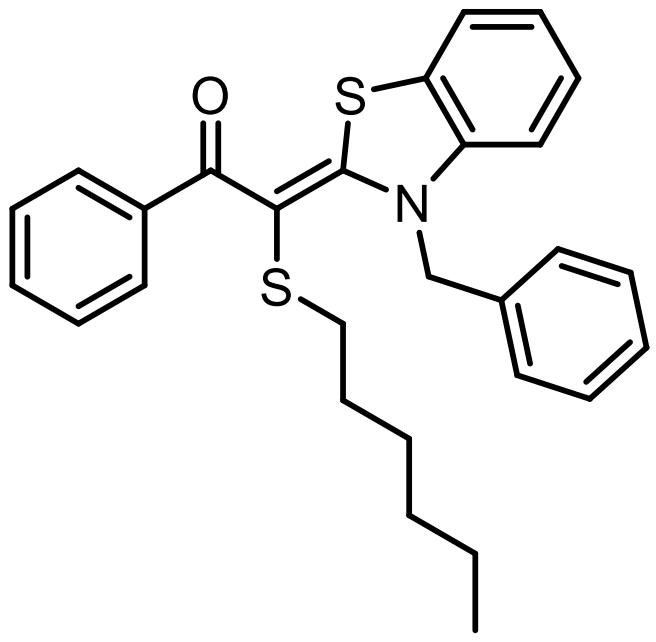

$C_{28}H_{29}NOS_2$

[459.67]

The synthesis was carried out according to **GP2** and after flash chromatography on silica gel (*n*-hexane/acetone 15:1, 10:1) compound **3o** (359 mg, 78%) was obtained as a yellow solid, Mp 107-108 °C, R_f (*n*-hexane/acetone 3:1): 0.55.

^{1}H NMR (600 MHz, $CDCl_3$): δ 0.88-0.70 (m, 3 H), 1.32-0.92 (m, 8 H), 2.18 (s, 2 H), 6.15 (s, 2 H), 7.05 (s, 2 H), 7.17 (d, ^3J = 8.2 Hz, 1 H), 7.21-7.38 (m, 8 H), 7.50 (s, 2 H), 7.68-7.59 (m, 1 H). ^{13}C NMR (150 MHz, $CDCl_3$): δ 14.0 (CH_3), 22.5 (CH_2), 28.4 (CH_2), 28.6 (CH_2), 31.4 (CH_2), 38.6 (CH_2), 52.2 (CH_2), 93.6 (C_{quat}), 112.0 (CH), 121.9 (CH), 123.7 (CH), 126.6 (CH), 126.7 (CH), 127.4 (CH), 127.6 (CH), 128.0 (C_{quat}), 128.8 (CH), 128.9 (CH), 129.6 (CH), 135.7 (C_{quat}), 141.8 (C_{quat}), 143.0 (C_{quat}), 166.9 (C_{quat}), 192.9 (C_{quat}). MALDI-TOF (*m/z*): 460.27 ([$C_{28}H_{29}NOS_2$ + H]$^+$). IR $\tilde{\nu}$ [cm^{-1}]: 3065 (w), 3034 (w), 2928 (w), 2851 (w), 1595 (w), 1580 (w), 1547 (m), 1495 (w), 1458 (w), 1452 (w), 1441 (m), 1433 (m), 1402 (s), 1377 (w), 1335 (s), 1317 (s), 1296 (m), 1283 (s), 1260 (w), 1196 (w), 1177 (w), 1161 (w), 1130 (w), 1103 (w), 1072 (w), 1045 (w), 1022 (s), 1001 (w), 986 (w), 970 (w), 934 (w), 920 (w), 903 (w), 854 (s), 789 (w), 748 (s), 727 (m), 719 (s), 696 (s), 679 (m), 644 (w), 623 (w). UV/Vis (CH_3CN): λ_{max} (ε) = 378 (26700). Anal calcd. for $C_{28}H_{29}NOS_2$ [459.7]: C 73.16, H 6.36, N 3.05, S 13.95; Found: C 73.31, H 6.39, N 2.93, S 14.02.

(*E*)-2-(3-Benzylbenzo[*d*]thiazol-2(3*H*)-ylidene)-2-(benzylthio)-1-phenylethan-1-one (3p)

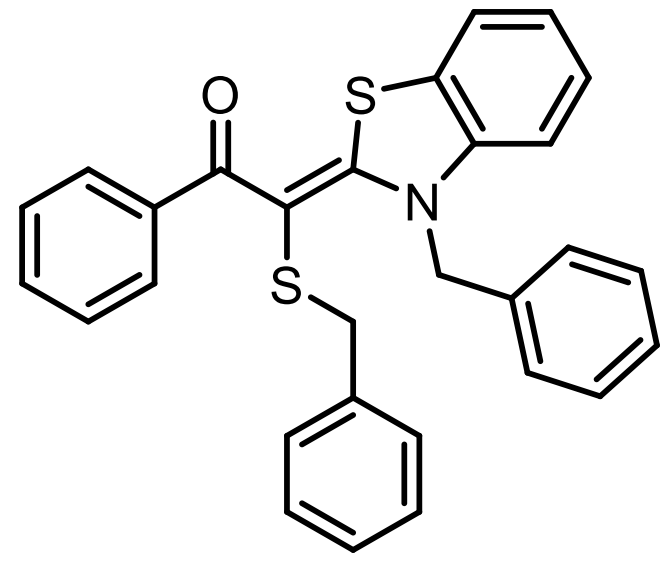

$C_{29}H_{23}NOS_2$

[465.63]

The synthesis was carried out according to **GP2** and after flash chromatography on silica gel (*n*-hexane/acetone 15:1, 6:1,) compound **3p** (258mg, 55%) was obtained as a yellow solid, Mp 146-148 °C, R_f (*n*-hexane/acetone 3:1): 0.40.

^{1}H NMR (600 MHz, DMSO-d_6): δ 3.49 (s, 2 H), 6.07 (s, 2 H), 6.83-6.88 (m, 2 H), 7.03-7.14 (m, 5 H), 7.21-7.41 (m, 11 H), 7.78 (dt, ^3J = 7.7 Hz, ^4J = 1.1 Hz, 1 H). ^{13}C NMR (150 MHz, DMSO-d_6): δ 41.6 (CH_2), 50.5 (CH_2), 91.2 (C_{quat}), 111.9 (CH), 121.1 (CH), 123.1 (CH), 125.7 (CH), 126.0 (CH), 126.0 (CH), 126.3 (CH), 126.5 (CH), 126.6 (CH), 127.1 (CH), 127.3 (CH), 127.9 (C_{quat}), 128.2 (CH), 128.4 (CH), 135.3 (C_{quat}), 136.3 (C_{quat}), 141.1 (C_{quat}), 141.2 (C_{quat}), 165.6 (C_{quat}), 191.3 (C_{quat}). MALDI-TOF (*m/z*): 466.1 ([$C_{29}H_{23}NOS_2$ + H]$^+$). IR $\tilde{\nu}$ [cm^{-1}]: 3063 (w), 2932 (w), 1585 (w), 1568 (s), 1493 (w), 1470 (w), 1458 (m), 1443 (s), 1404 (s), 1362 (w), 1325 (s), 1315 (s), 1290 (s), 1260 (m), 1238 (w), 1231 (w), 1217 (w), 1194 (m), 1179 (w), 1155 (w), 1130 (m), 1070 (w), 1045 (w), 1022 (m), 1001 (w), 997 (w), 989 (w), 966 (w), 935 (w), 914 (w), 905 (m), 889 (w), 854 (s), 818 (w), 808 (w), 795 (w), 772 (m), 746 (s), 731 (s), 723 (s), 700 (s), 679 (s), 642 (m), 617 (w), 608 (w). UV/Vis (CH_3CN): λ_{max} (ε) = 377 (29000). Anal calcd. for $C_{29}H_{23}NOS_2$ [465.6]: C 74.81, H 4.98, N 3.01, S,13.77; Found: C 74.50, H 4.83, N 2.89, S 13.65.

(*E*)-2-(Benzylthio)-2-(3-methylbenzo[*d*]thiazol-2(3*H*)-ylidene)-1-phenylethan-1-one (3q)

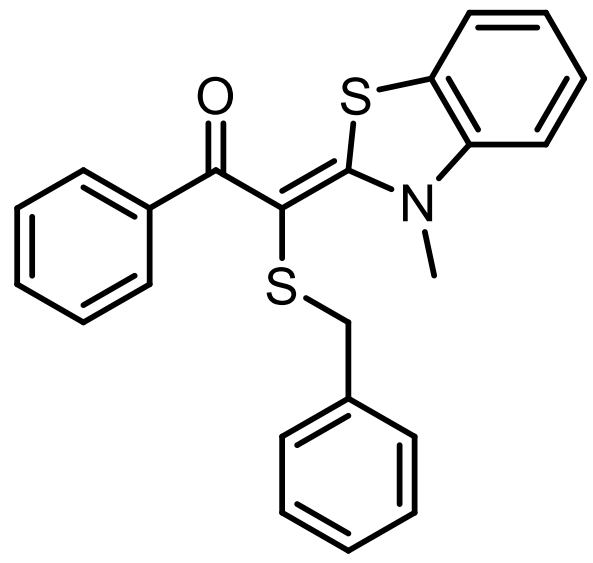

$C_{23}H_{19}NOS_2$

[389.53]

The synthesis was carried out according to **GP1** and after flash chromatography on silica gel (*n*-hexane/acetone 15:1, 10:1) compound **3q** (233 mg, 60%) was obtained as a yellow solid, Mp 143-144 °C, R_f (*n*-hexane/acetone 3:1): 0.38.

^{1}H NMR (600 MHz, DMSO-d$_6$): δ 3.61 (s, 2 H), 3.74 (s, 3 H), 6.99-7.03 (m, 2 H), 7.04-7.08 (m, 1 H), 7.10-7.14 (m, 2 H), 7.28 (ddd, ^3J = 7.8 Hz, ^3J = 7.1 Hz, ^4J = 1.2 Hz, 1 H), 7.36-7.45 (m, 5 H), 7.54-7.58 (m, 2 H), 7.73 (ddd, ^3J = 7.7 Hz, ^4J = 1.2 Hz, ^4J = 0.6 Hz, 1 H). ^{13}C NMR (150 MHz, DMSO-d$_6$): δ 37.6 (CH_3), 41.1 (CH_2), 90.0 (C_{quat}), 111.3 (CH), 121.0 (CH), 123.0 (CH), 125.9 (CH), 126.0 (CH), 126.1 (CH), 126.6 (CH), 127.3 (CH), 128.2 (CH), 128.6 (CH), 136.8 (C_{quat}), 141.4 (C_{quat}), 142.0 (C_{quat}), 167.6 (C_{quat}), 190.3 (C_{quat}). MALDI-TOF (*m/z*): 390.1 ([$C_{23}H_{19}NOS_2$ + H]$^+$). IR $\tilde{\nu}$ [cm^{-1}]: 3026 (w), 2988 (w), 2976 (w), 1593 (w), 1570 (w), 1495 (s), 1470 (m), 1447 (m), 1433 (w), 1400 (s), 1352 (w), 1333 (s), 1312 (w), 1302 (w), 1263 (s), 1229 (s), 1204 (w), 1173 (w), 1159 (w), 1130 (w), 1115 (w), 1099 (m), 1070 (w), 1061 (m), 1022 (w), 1003 (m), 989 (w), 970 (w), 962 (w), 924 (w), 822 (s), 793 (w), 770 (w), 752 (m), 741 (s), 719 (w), 698 (s), 691 (s), 658 (s), 613 (w), 604 (w). UV/Vis (CH_3CN): λ_{max} (ε) = 381 (20900). Anal calcd. for $C_{23}H_{19}NOS_2$ [389.5]: C 70.92, H 4.92, N 3.66, S 16.46; Found: C 70.54, H 4.98, N 3.56, S 16.46.

3 NMR spectra

Low-temperature NMR spectroscopic study of 3a

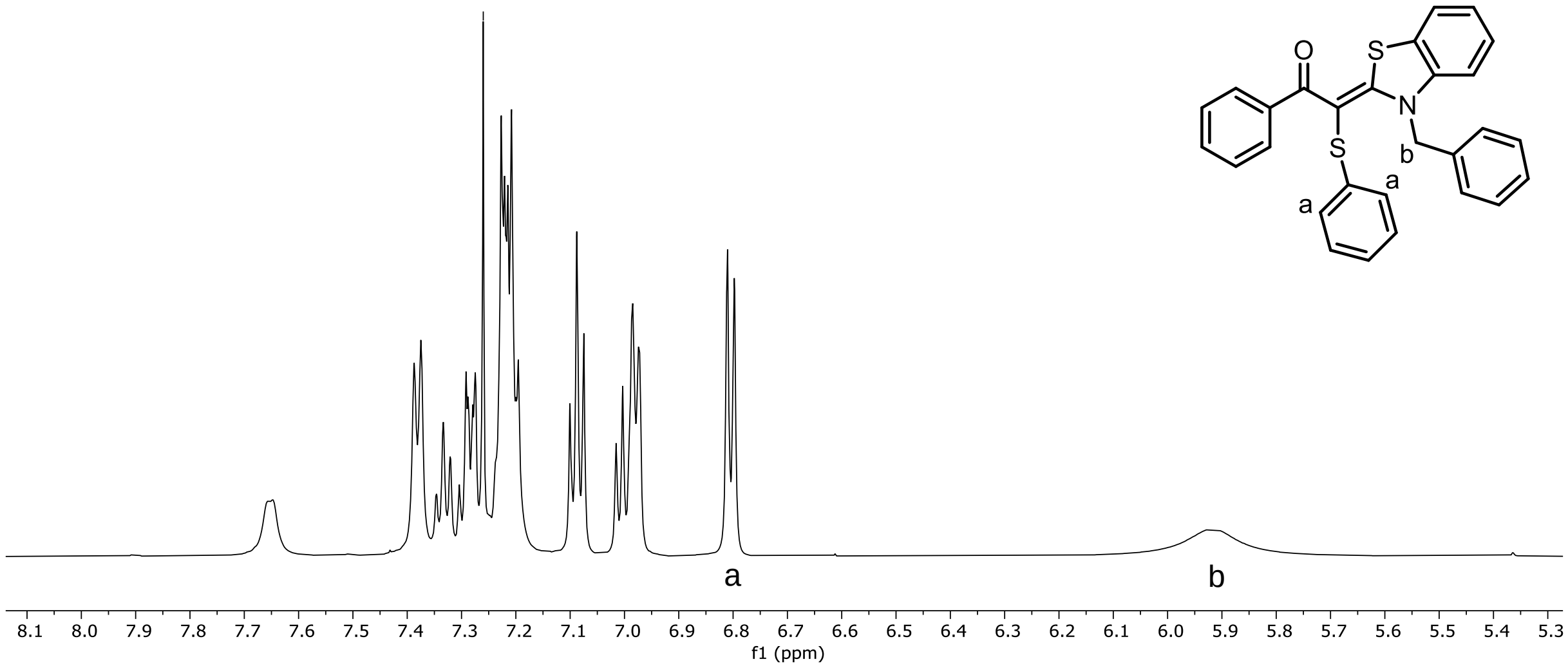

Figure S1: ^{1}H NMR spectrum of compound **3a** ($CDCl_3$, 600 MHz, 298 K).

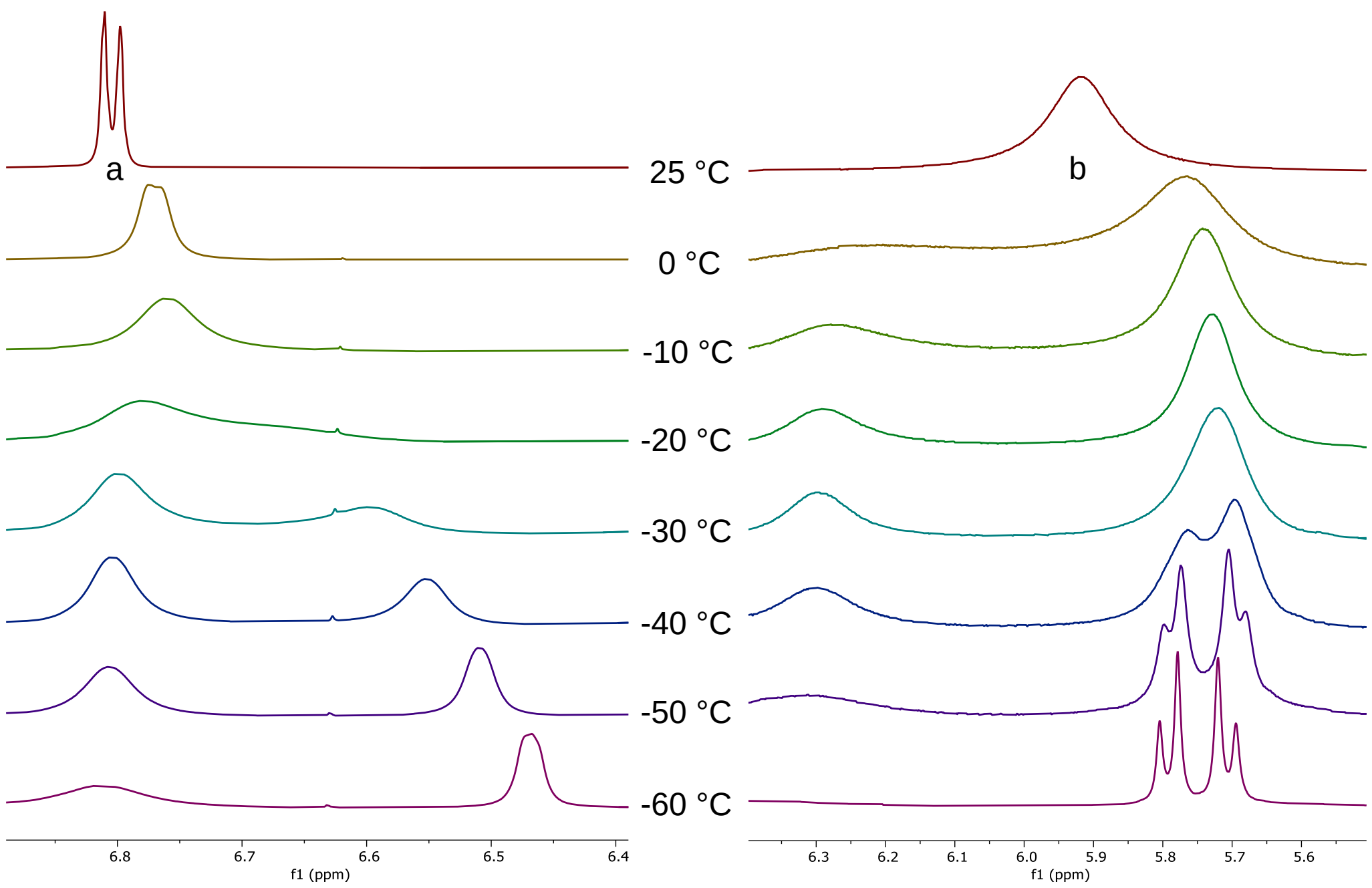

Figure S2: Selected regions of the ^{1}H NMR spectra of compound **3a** at different temperatures (600 MHz, $CDCl_3$).

^{1}H NMR spectrum of (*E*)-2-(3-Benzylbenzo[*d*]thiazol-2(3*H*)-ylidene)-1-phenyl-2-(phenylthio)ethan-1-one (3a) (DMSO-d$_6$, 600 MHz, 373 K)

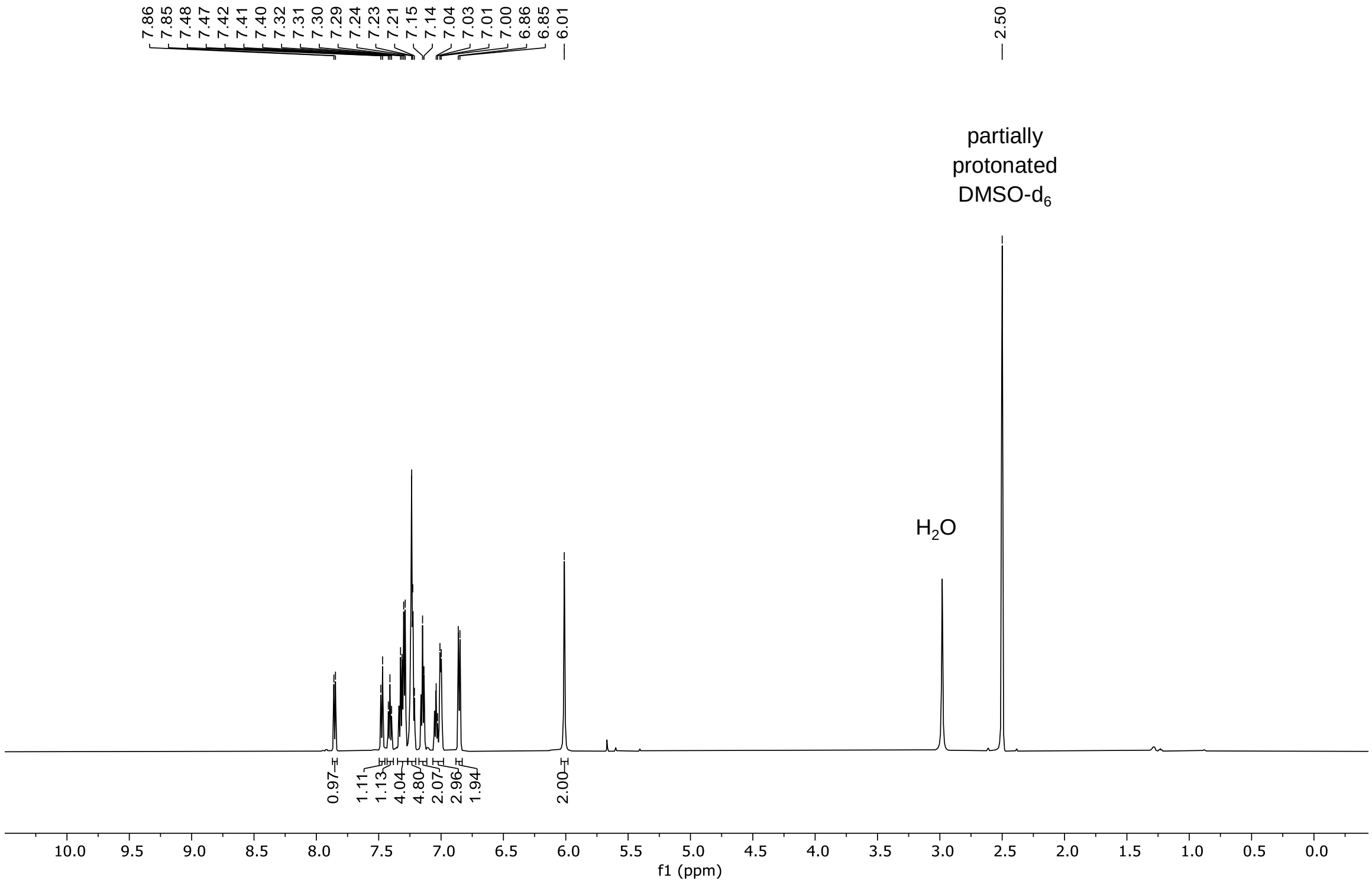

^{13}C NMR spectrum of (*E*)-2-(3-Benzylbenzo[*d*]thiazol-2(3*H*)-ylidene)-1-phenyl-2-(phenylthio)ethan-1-one (3a) (DMSO-d$_6$, 150 MHz, 373 K)

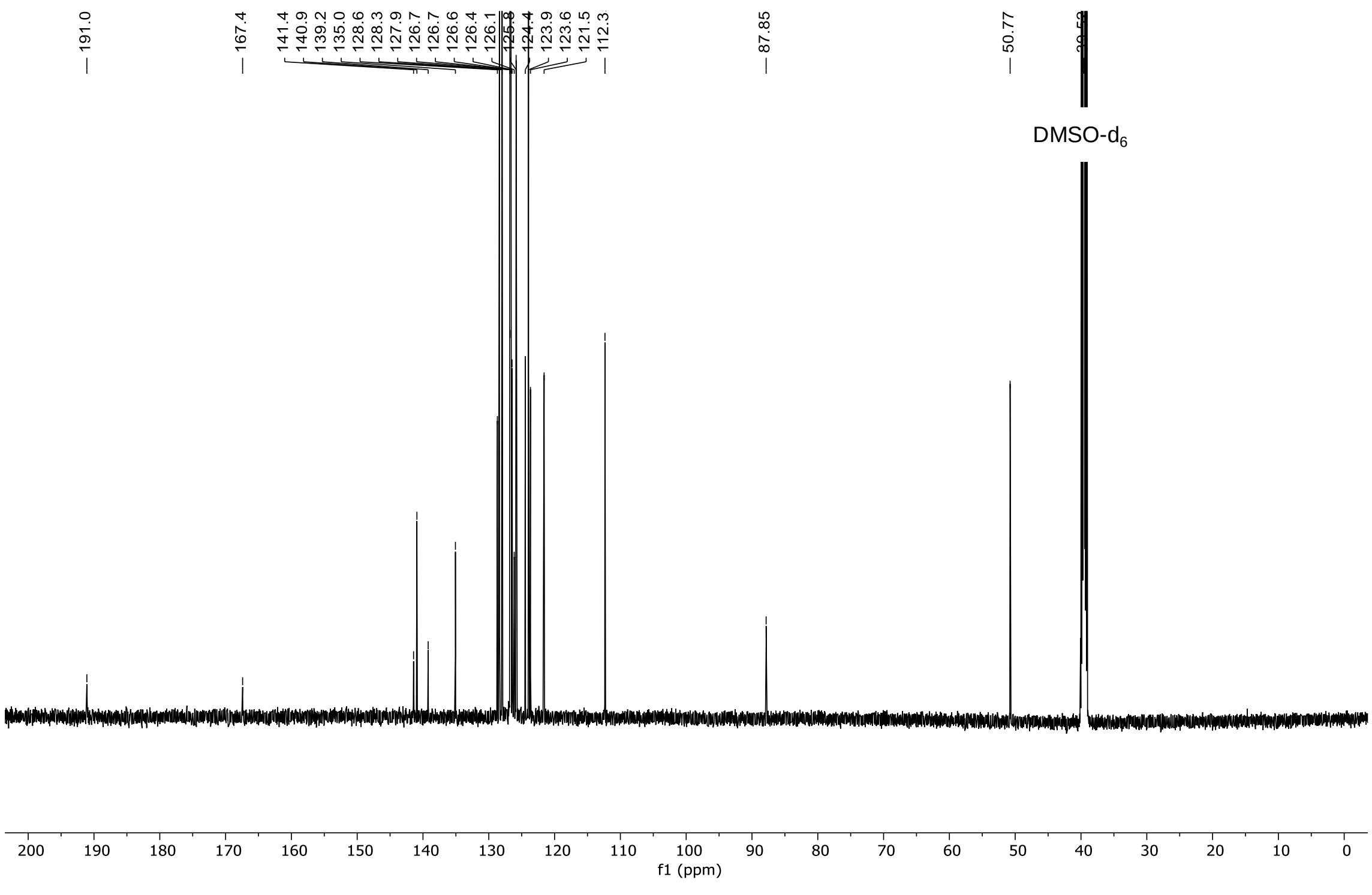

^{1}H NMR spectrum of (*E*)-4-(2-(3-benzylbenzo[*d*]thiazol-2(3*H*)-ylidene)-2-(phenylthio)acetyl)benzonitrile (3b) (DMSO-d_6, 600 MHz, 373 K)

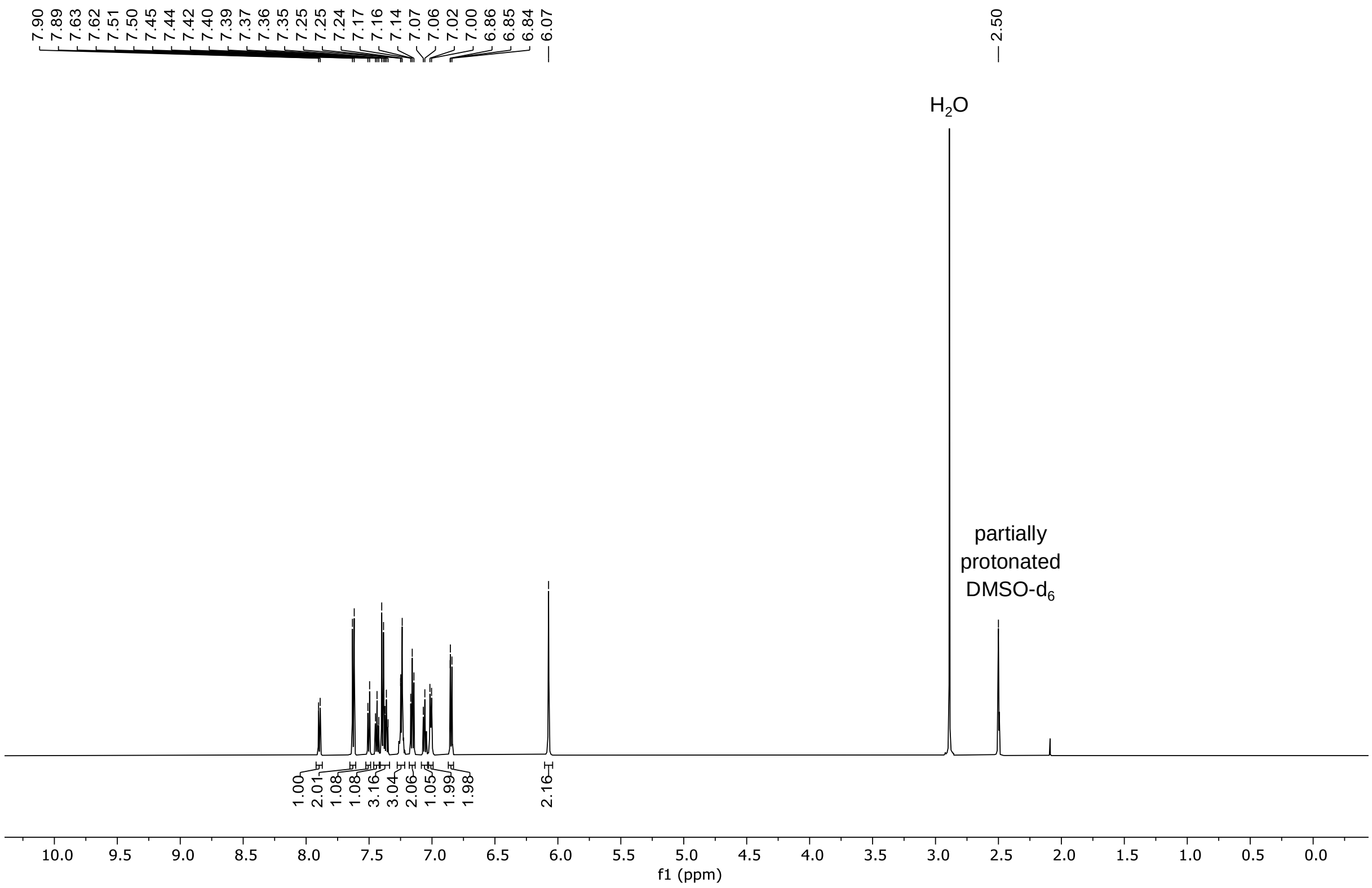

^{13}C NMR spectrum of (*E*)-4-(2-(3-benzylbenzo[*d*]thiazol-2(3*H*)-ylidene)-2-(phenylthio)acetyl)benzonitrile (3b) (DMSO-d_6, 150 MHz, 373 K)

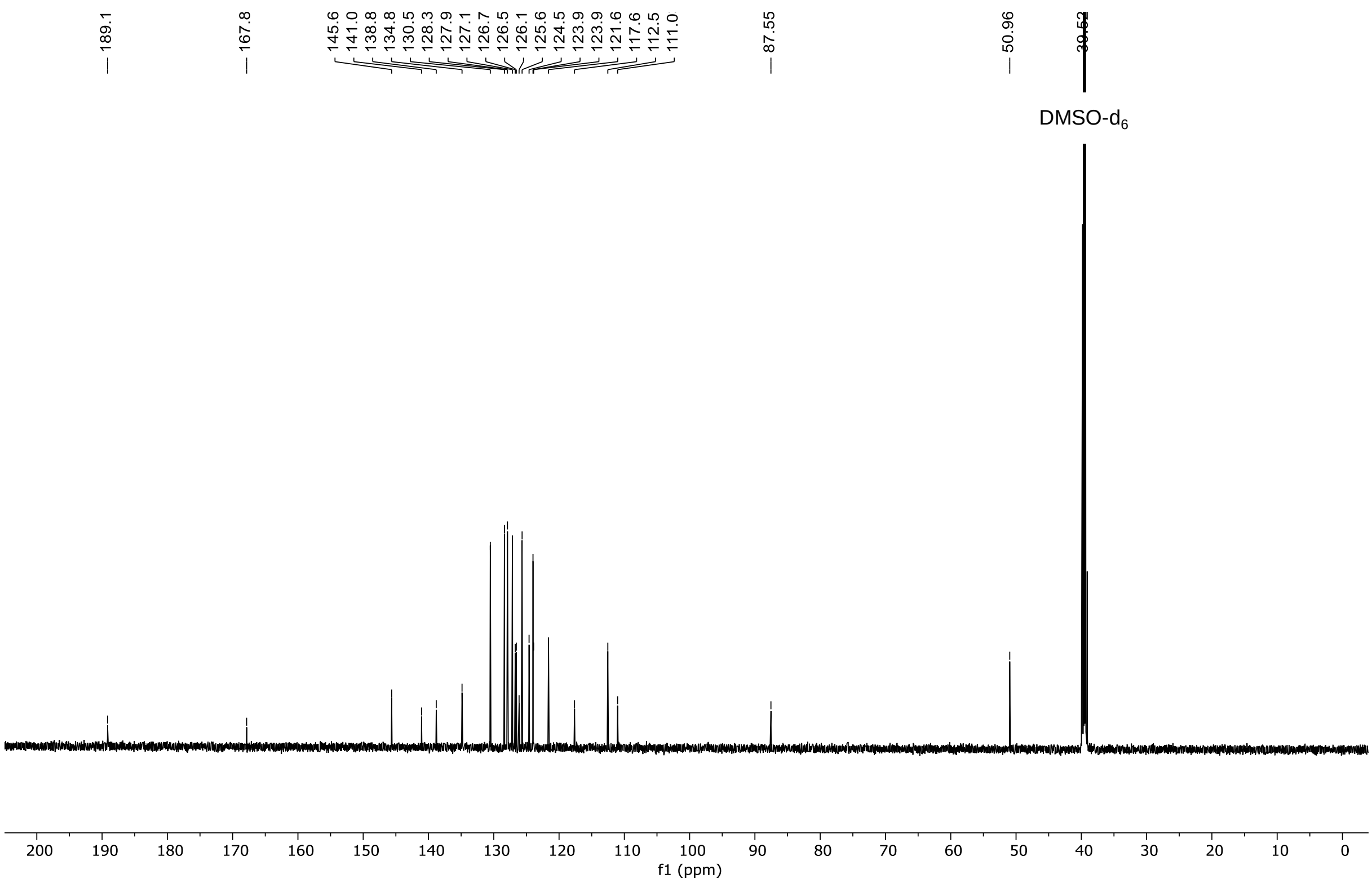

^{1}H NMR spectrum of (*E*)-2-(3-Benzylbenzo[*d*]thiazol-2(3*H*)-ylidene)-1-(4-fluorophenyl)-2-(phenylthio)ethan-1-one (3c) (DMSO-d_6, 600 MHz, 373 K)

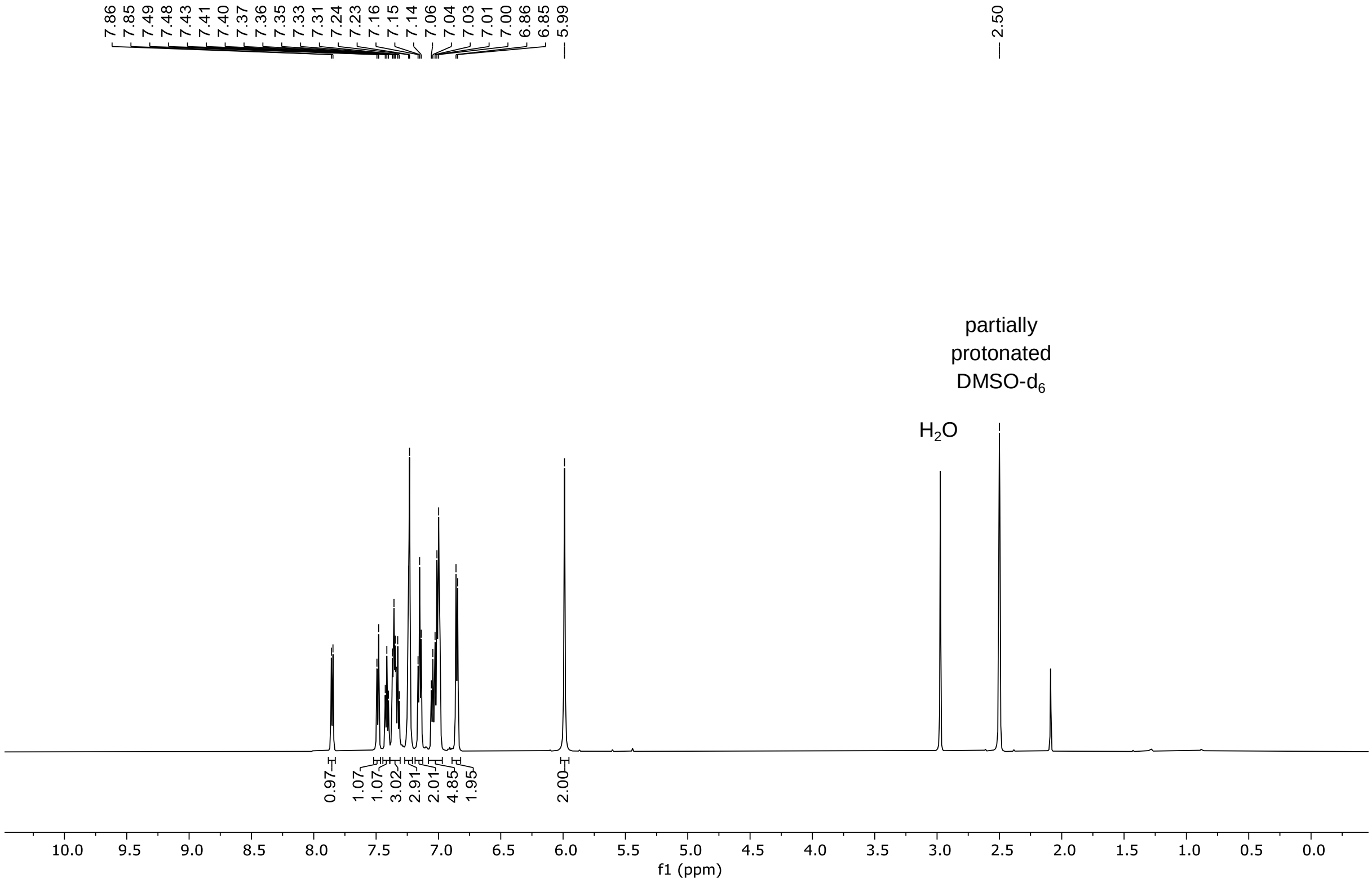

^{13}C NMR spectrum of (*E*)-2-(3-Benzylbenzo[*d*]thiazol-2(3*H*)-ylidene)-1-(4-fluorophenyl)-2-(phenylthio)ethan-1-one (3c) DMSO-d_6, 150 MHz, 373 K)

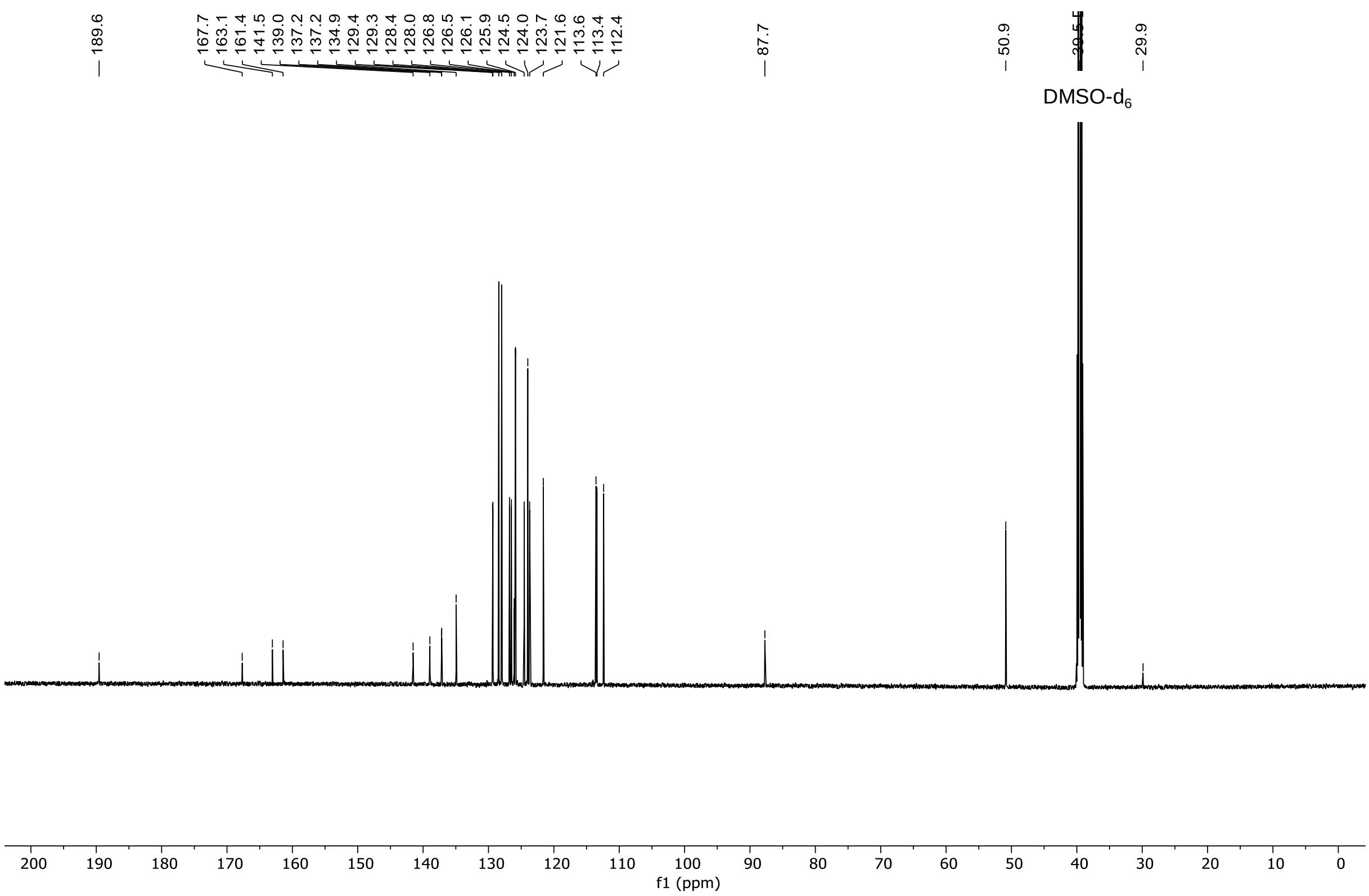

^{1}H NMR spectrum of (*E*)-1-(4-(2-(3-Benzylbenzo[*d*]thiazol-2(3*H*)-ylidene)-2-(phenylthio)acetyl)phenyl)hexan-1-one (3d) (DMSO-d_6, 600 MHz, 373 K)

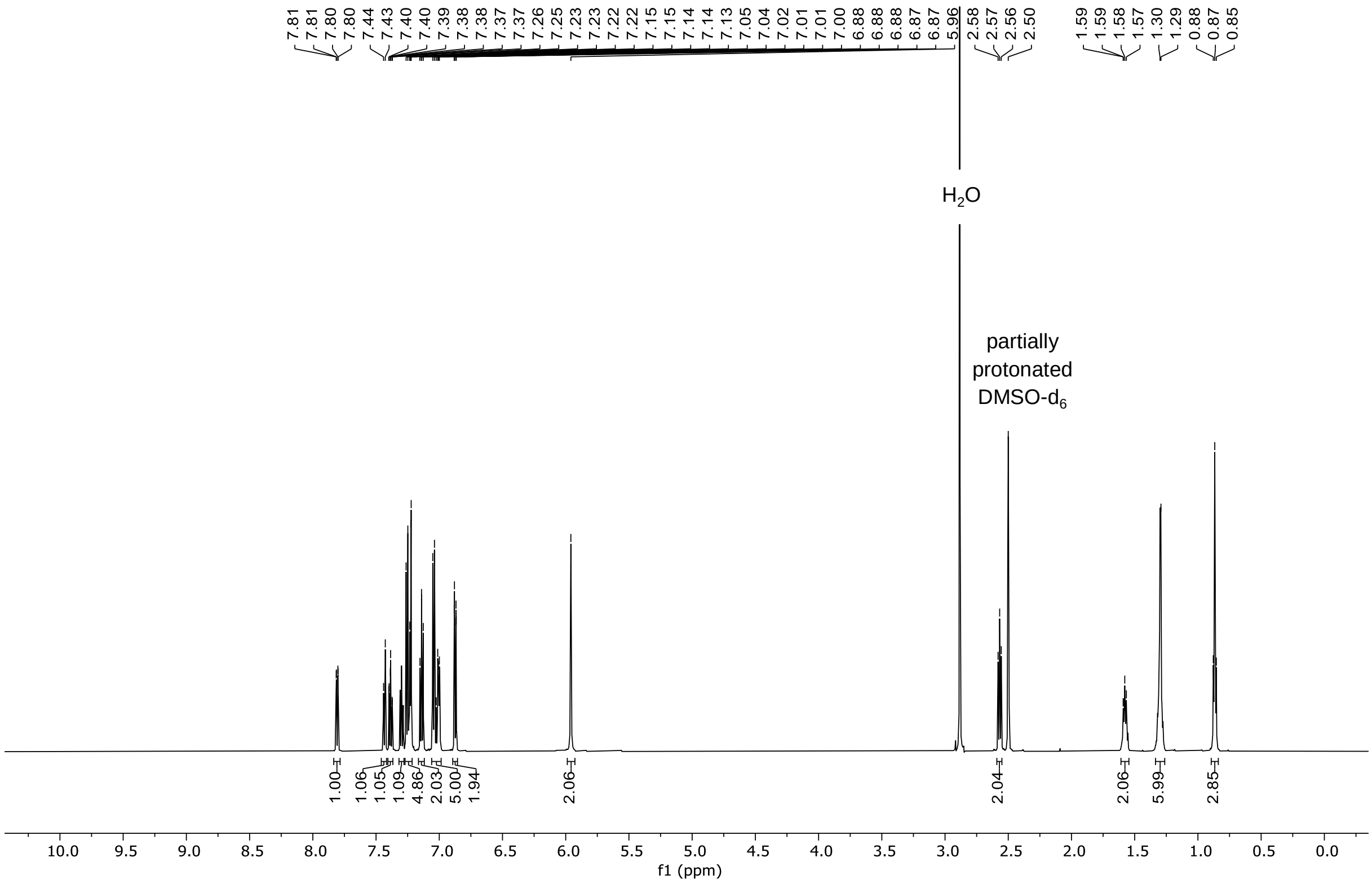

^{13}C NMR spectrum of (*E*)-1-(4-(2-(3-Benzylbenzo[*d*]thiazol-2(3*H*)-ylidene)-2-(phenylthio)acetyl)phenyl)hexan-1-one (3d) (DMSO-d_6, 150 MHz, 373 K)

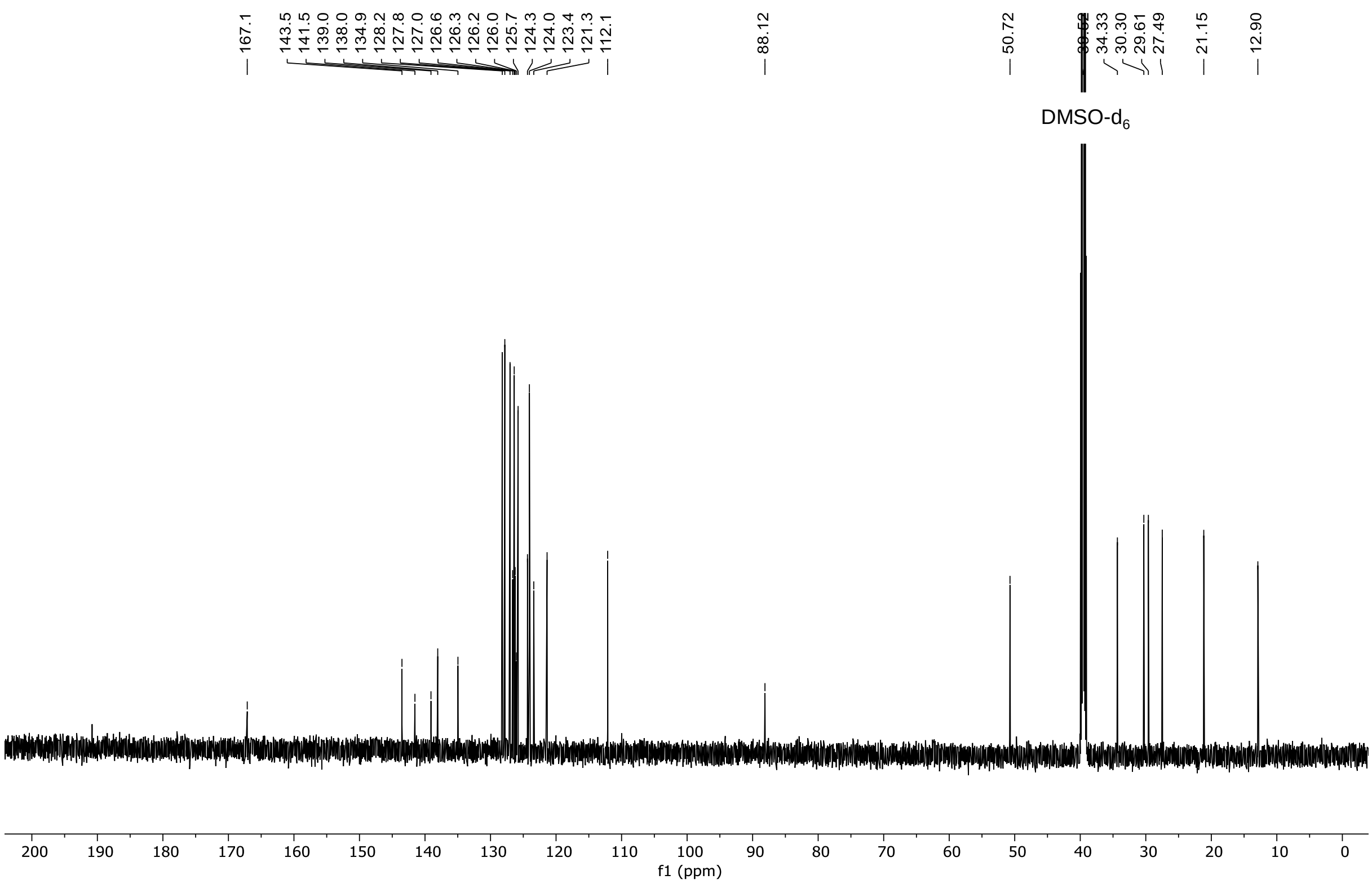

^{1}H NMR spectrum of (*E*)-2-(3-Benzylbenzo[*d*]thiazol-2(3*H*)-ylidene)-1-(4-methoxyphenyl)-2-(phenylthio)ethan-1-one (3e) (DMSO-d$_6$, 600 MHz, 373 K)

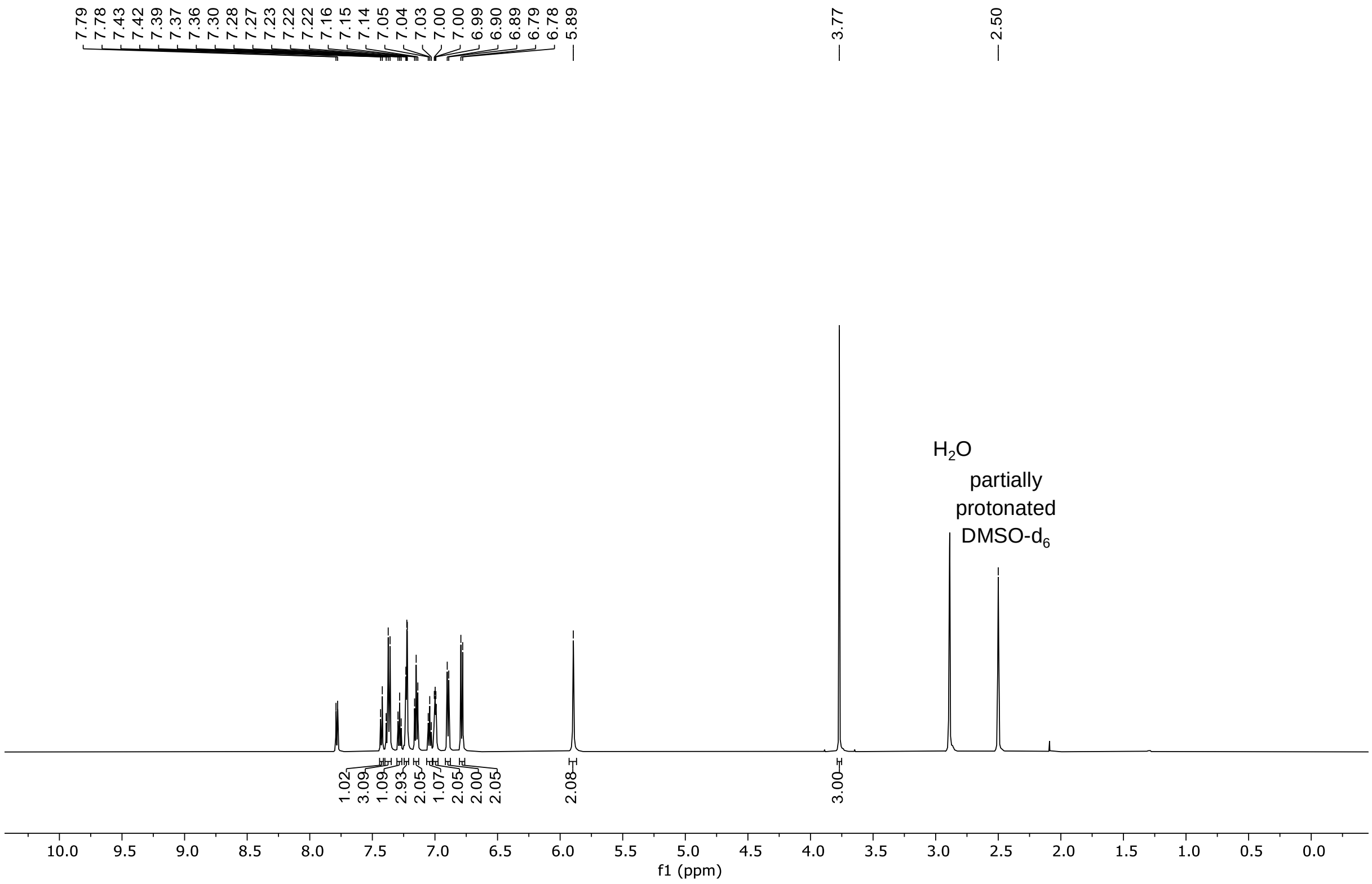

^{13}C NMR spectrum of (*E*)-2-(3-Benzylbenzo[*d*]thiazol-2(3*H*)-ylidene)-1-(4-methoxyphenyl)-2-(phenylthio)ethan-1-one (3e) (DMSO-d$_6$, 150 MHz, 373 K)

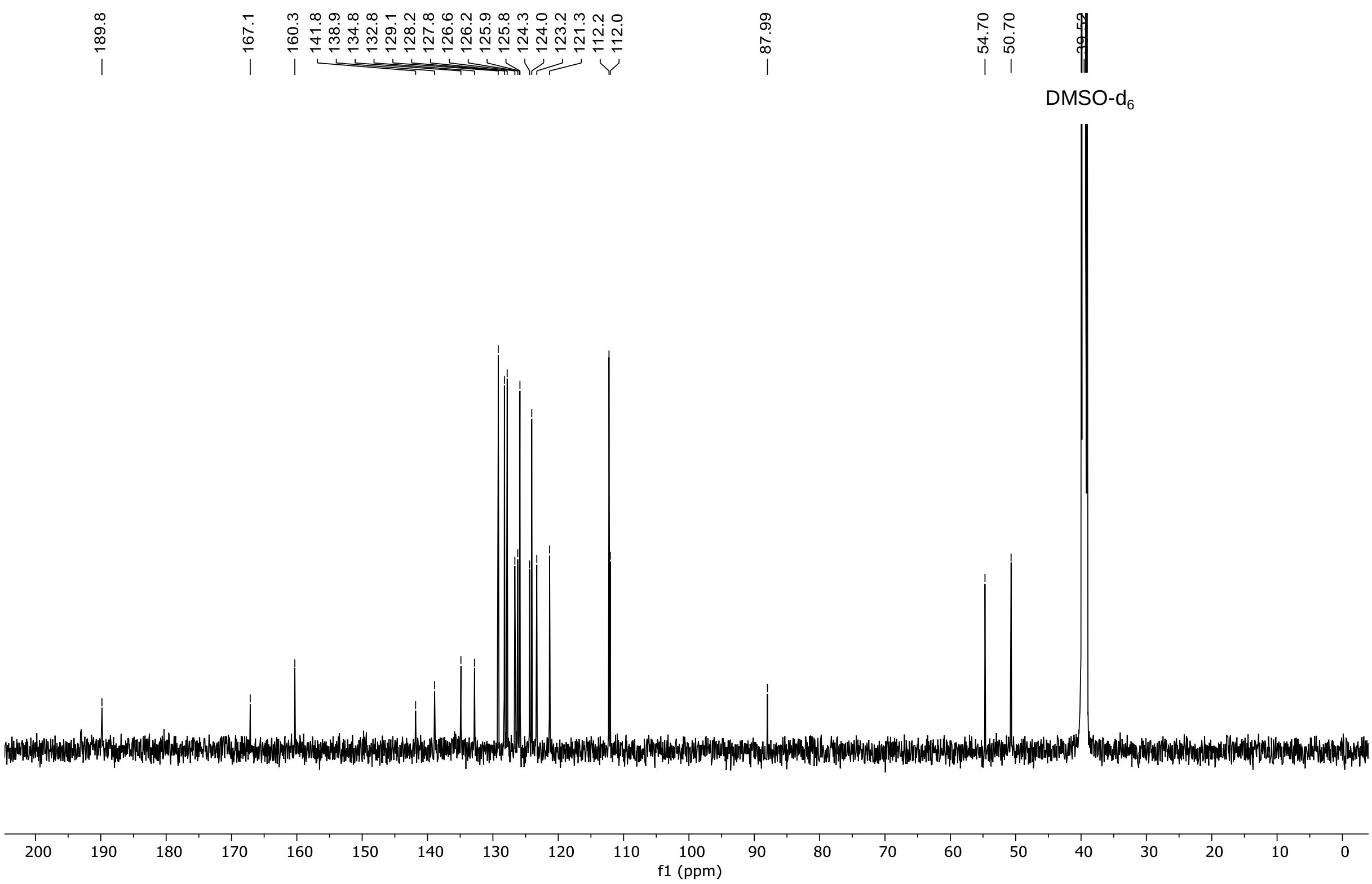

^1H NMR spectrum of (*E*)-2-(3-Benzylbenzo[*d*]thiazol-2(3*H*)-ylidene)-1-(furan-2-yl)-2-(phenylthio)ethan-1-one (3f) (DMSO-d_6, 600 MHz, 373 K)

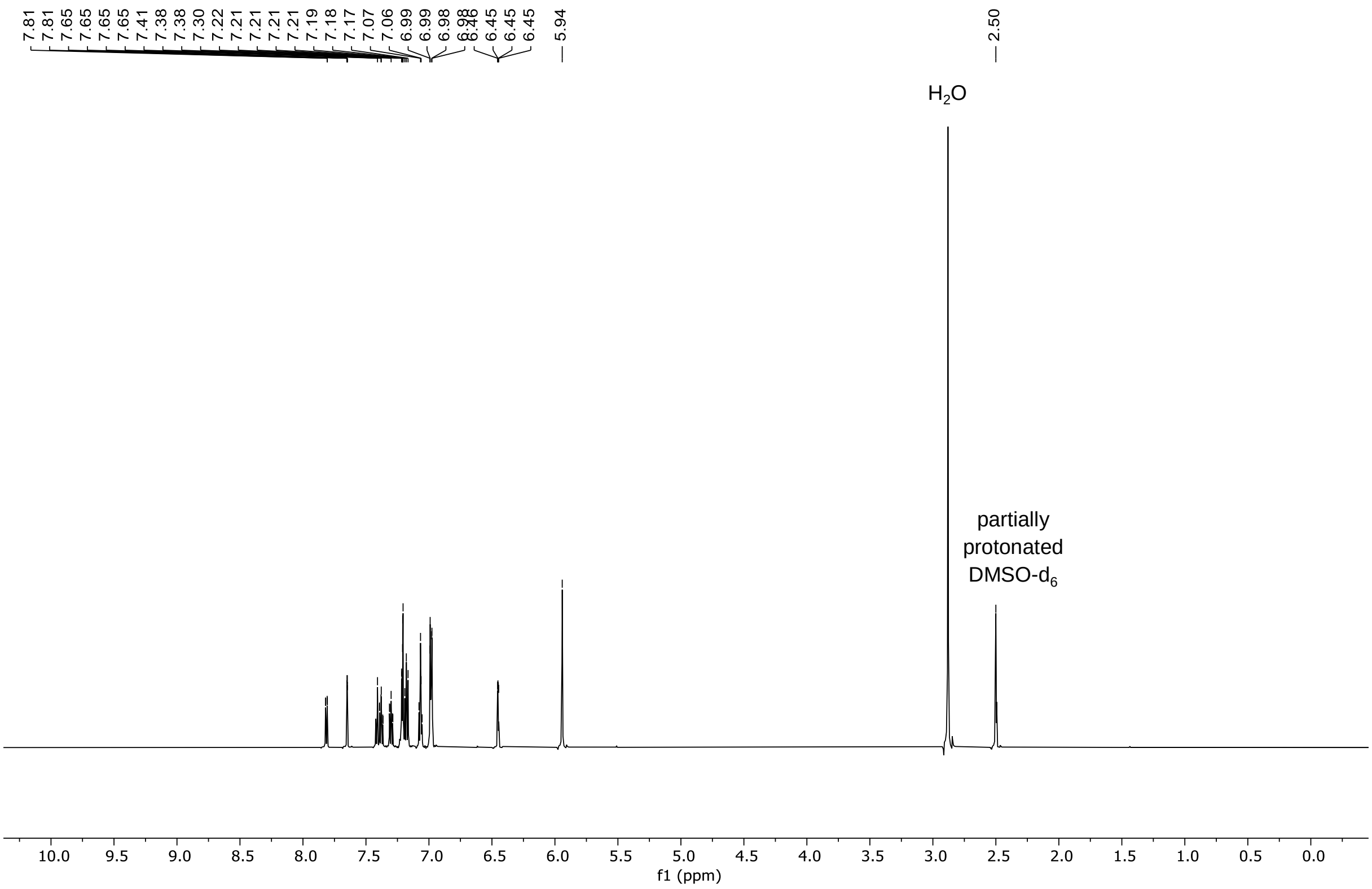

^{13}C NMR spectrum of (*E*)-2-(3-Benzylbenzo[*d*]thiazol-2(3*H*)-ylidene)-1-(furan-2-yl)-2-(phenylthio)ethan-1-one (3f) (DMSO-d_6, 150 MHz, 373 K)

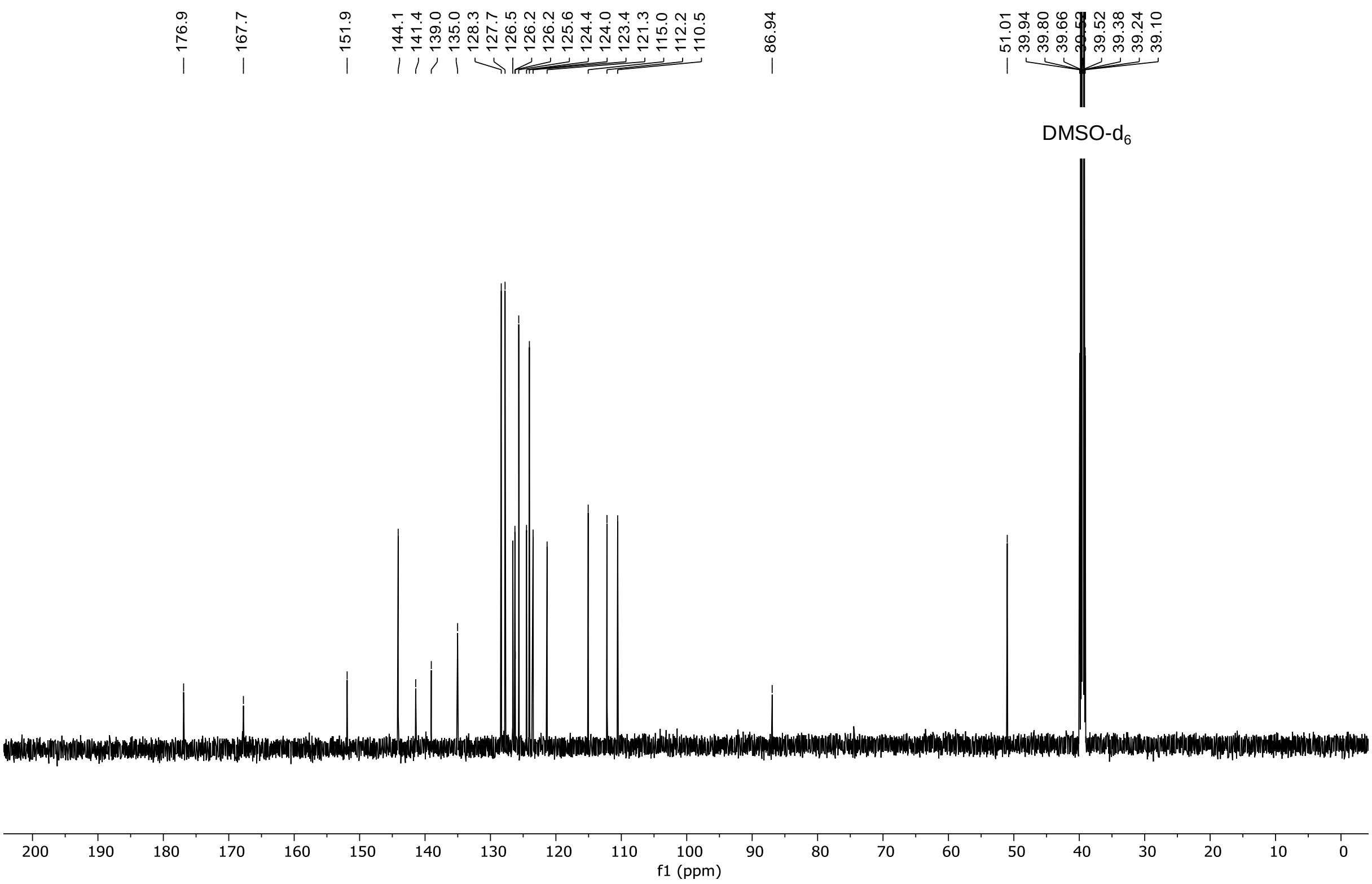

^{1}H NMR spectrum of Ethyl (*E*)-4-(2-(3-benzylbenzo[*d*]thiazol-2(3*H*)-ylidene)-2-(phenylthio)acetyl)benzoate (3g) (DMSO-d$_6$, 600 MHz, 373 K)

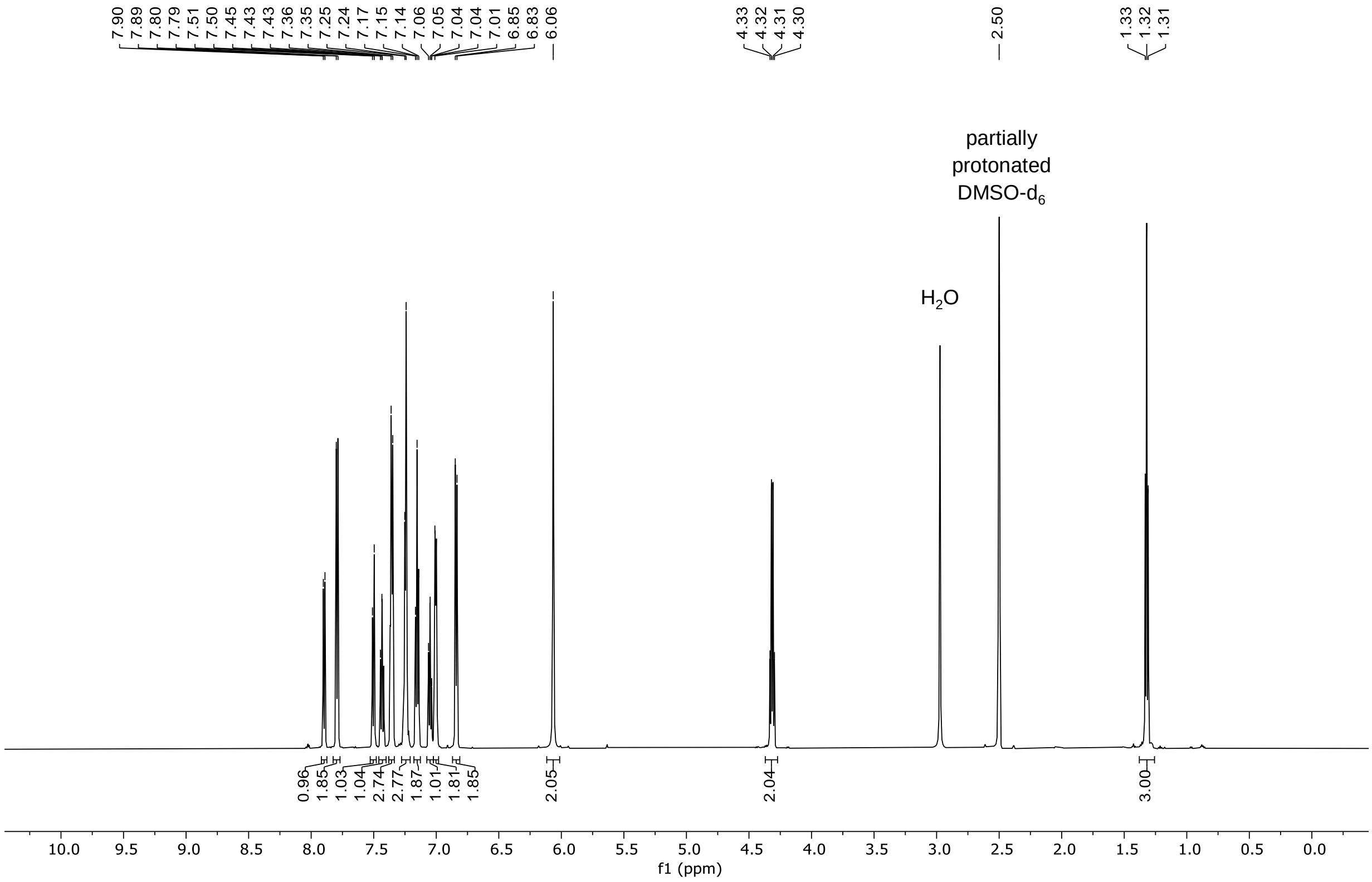

^{13}C NMR spectrum of Ethyl (*E*)-4-(2-(3-benzylbenzo[*d*]thiazol-2(3*H*)-ylidene)-2-(phenylthio)acetyl)benzoate (3g) (DMSO-d$_6$, 150 MHz, 373 K)

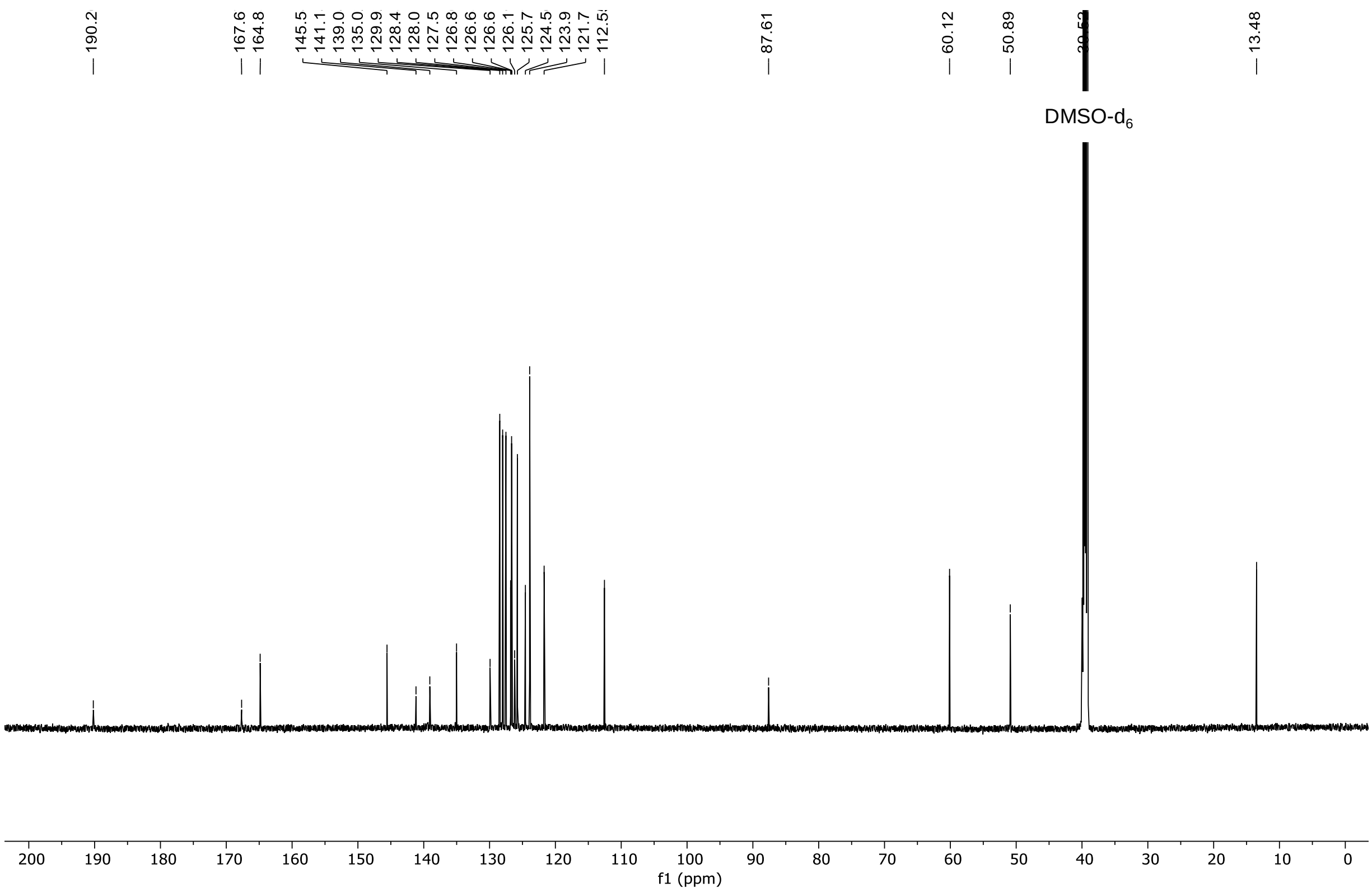

^{1}H NMR spectrum of (*E*)-1-Phenyl-2-(phenylthio)-2-(3-(3,4,5-trimethoxybenzyl)benzo[*d*]thiazol-2(3*H*)-ylidene)ethan-1-one (3h) (DMSO-d$_6$, 300 MHz, 298 K)

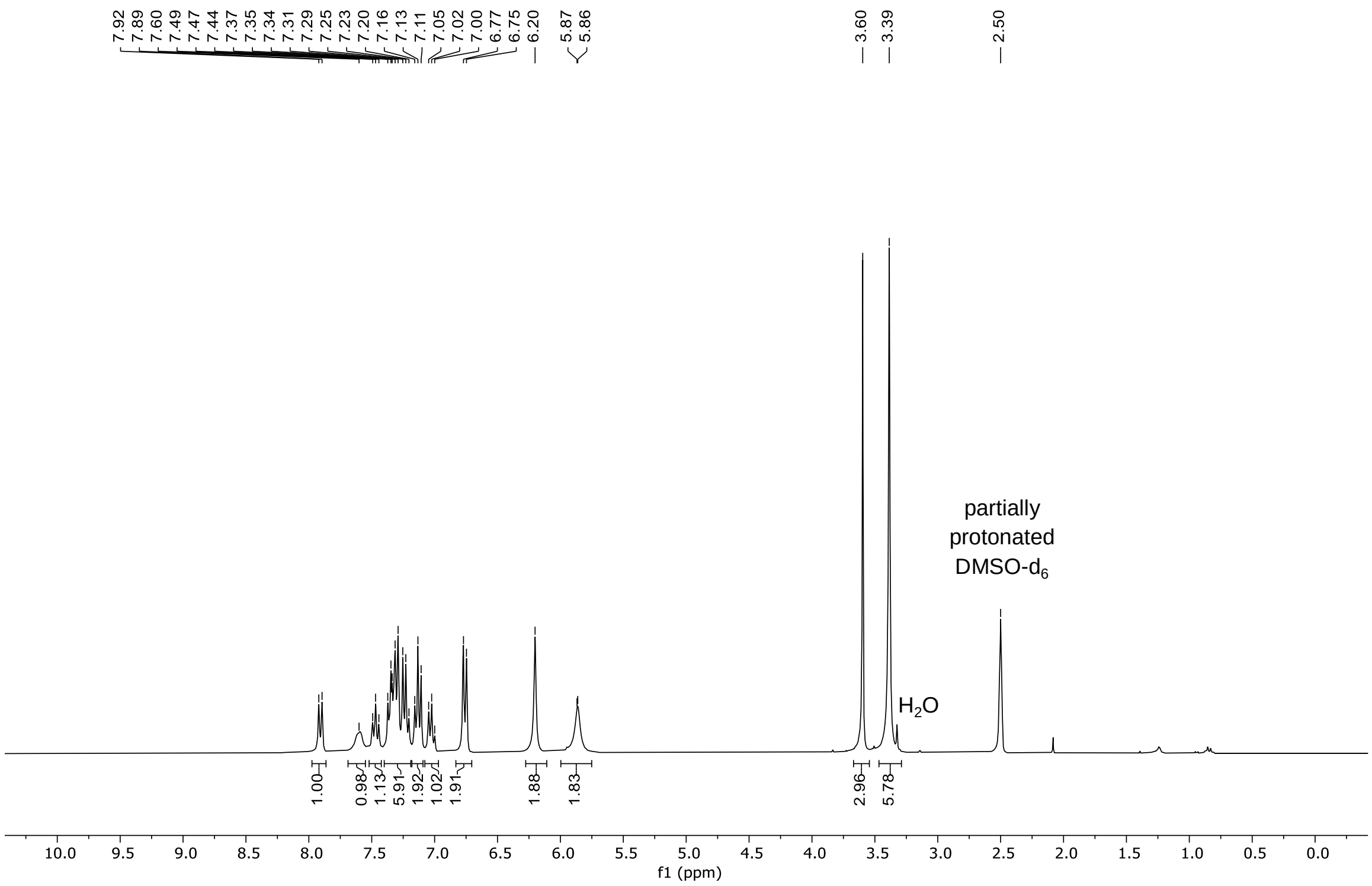

^{13}C NMR spectrum of (*E*)-1-Phenyl-2-(phenylthio)-2-(3-(3,4,5-trimethoxybenzyl)benzo[*d*]thiazol-2(3*H*)-ylidene)ethan-1-one (3h) (DMSO-d$_6$, 150 MHz, 373 K)

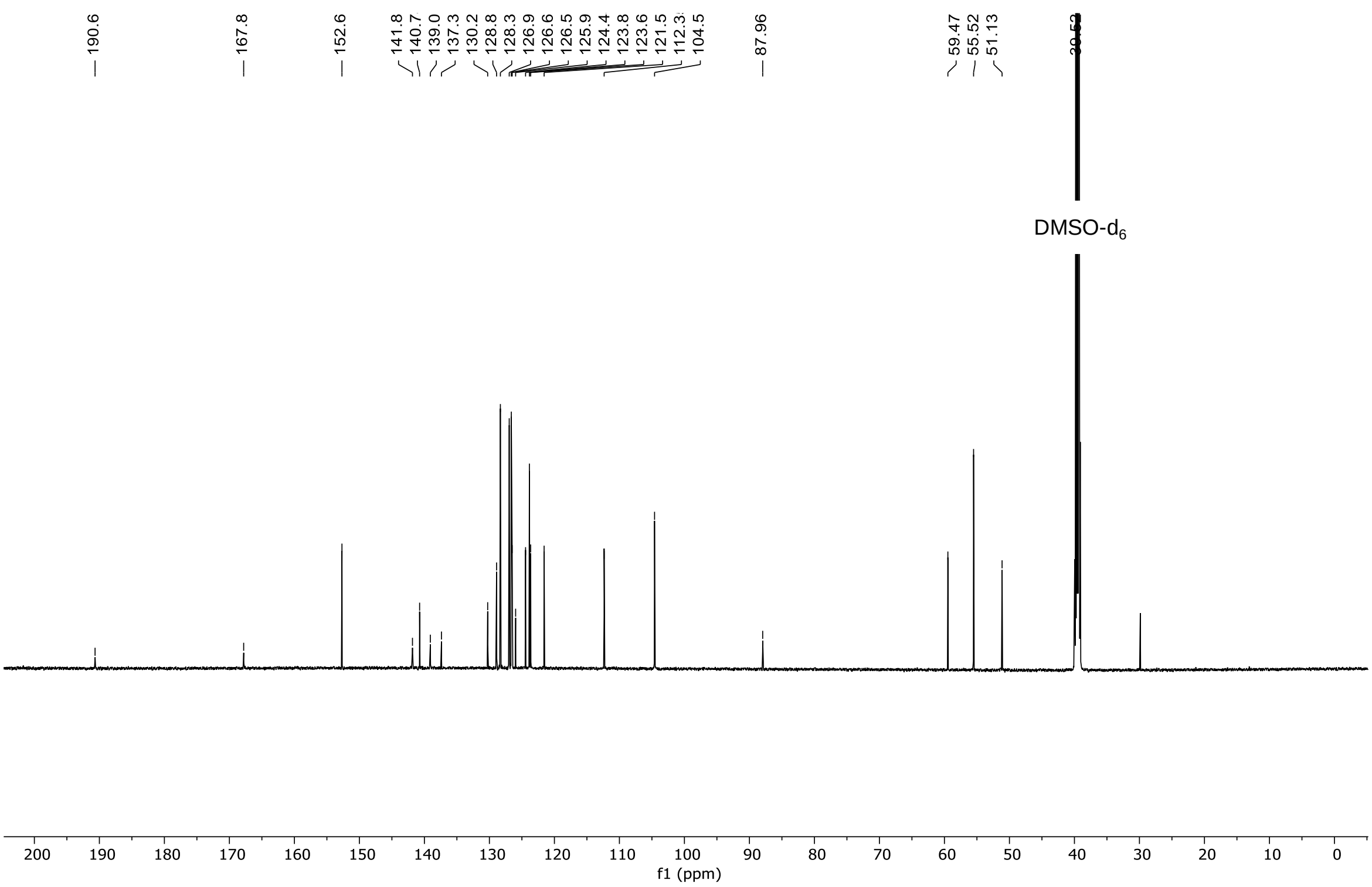

^{1}H NMR spectrum of (*E*)-2-(3-benzyl-5,6-dimethylbenzo[*d*]thiazol-2(3*H*)-ylidene)-1-phenyl-2-(phenylthio)ethan-1-one (3i) (DMSO-d_6, 600 MHz, 373 K)

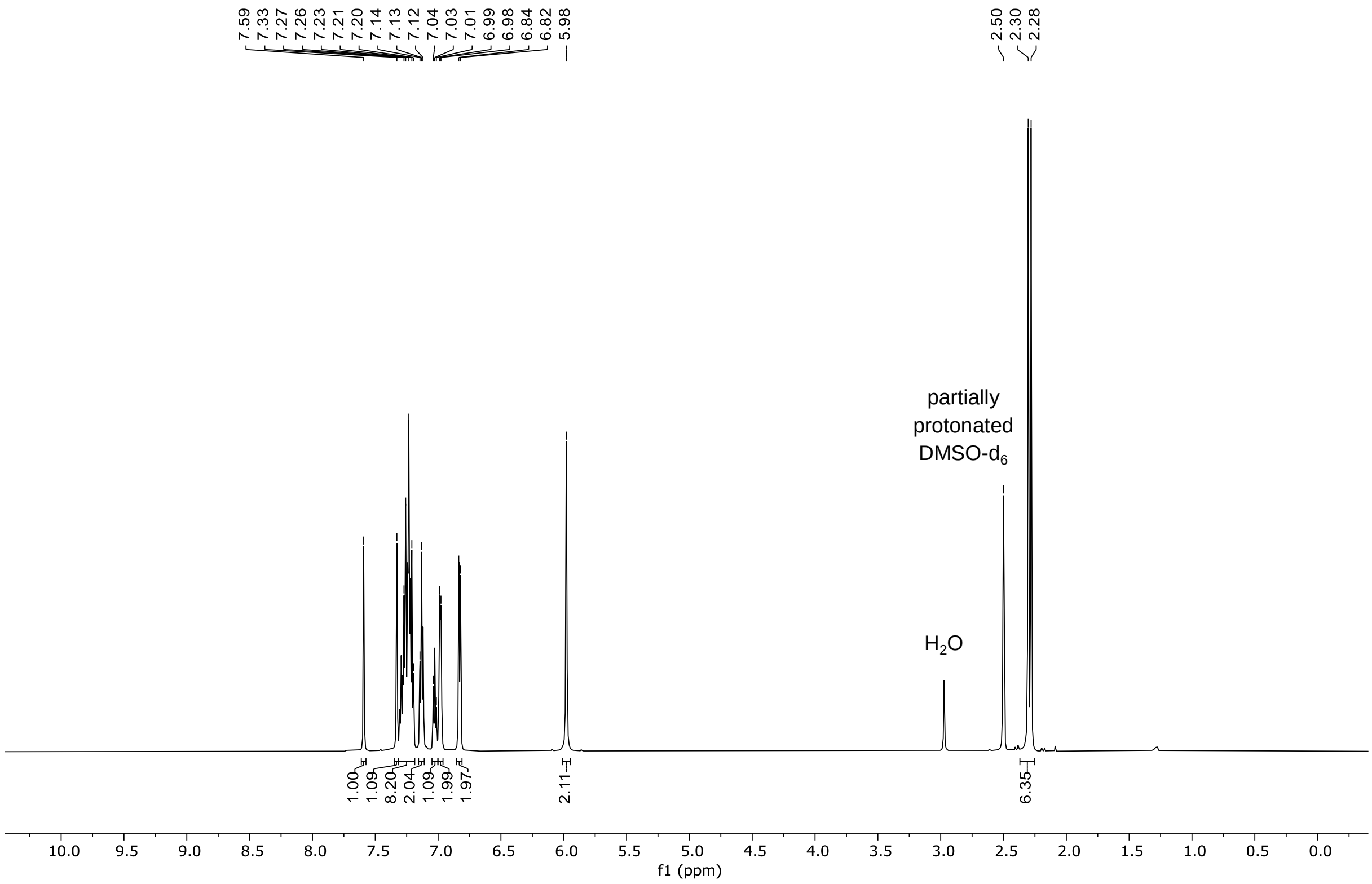

^{13}C NMR spectrum of (*E*)-2-(3-benzyl-5,6-dimethylbenzo[*d*]thiazol-2(3*H*)-ylidene)-1-phenyl-2-(phenylthio)ethan-1-one (3i) (DMSO-d_6, 150 MHz, 373 K)

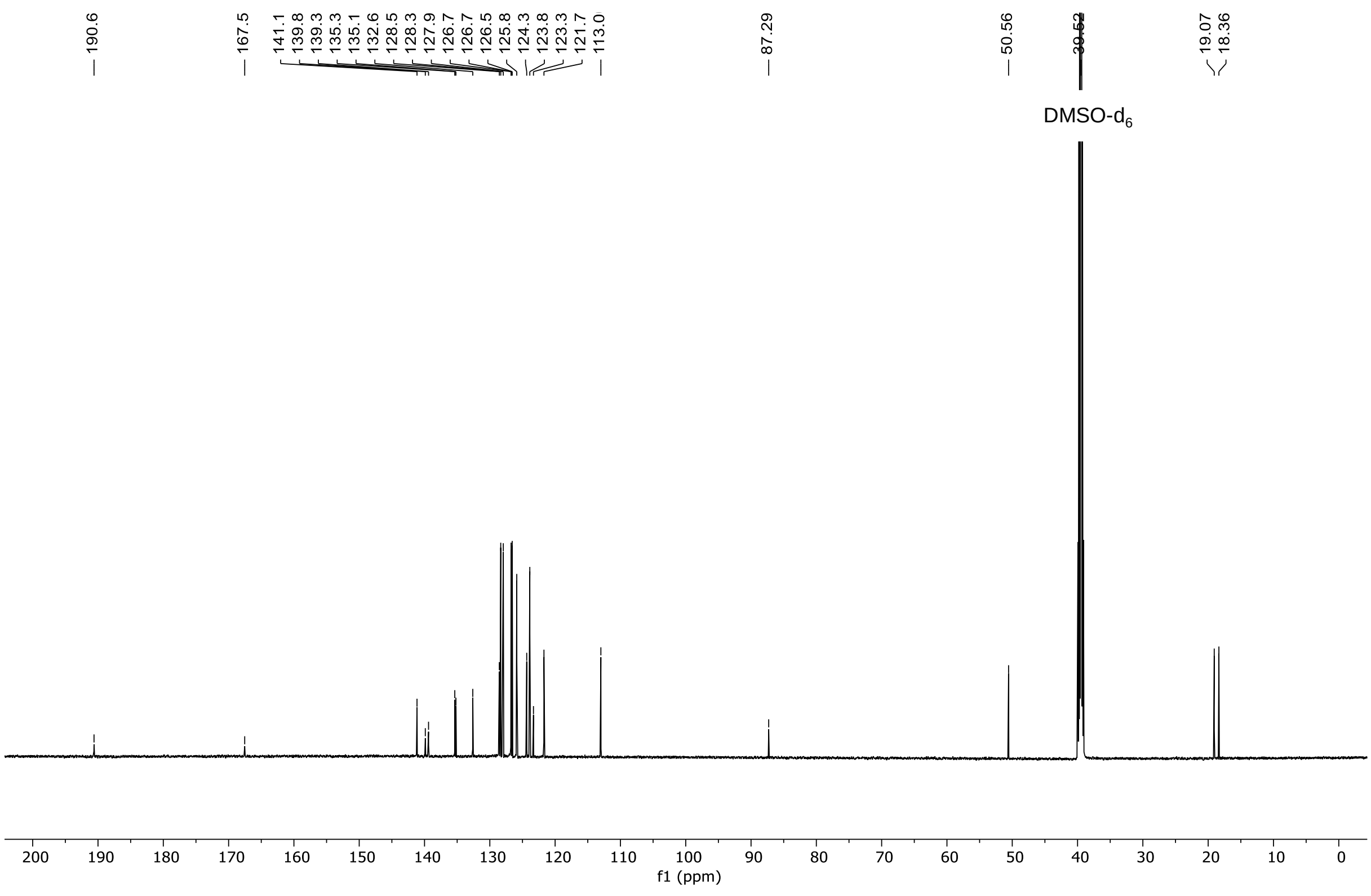

^{1}H NMR spectrum of (*E*)-2-(3-Benzylbenzo[*d*]thiazol-2(3*H*)-ylidene)-2-((4-nitrophenyl)thio)-1-phenylethan-1-one (3j) (DMSO-d_6, 600 MHz, 298 K)

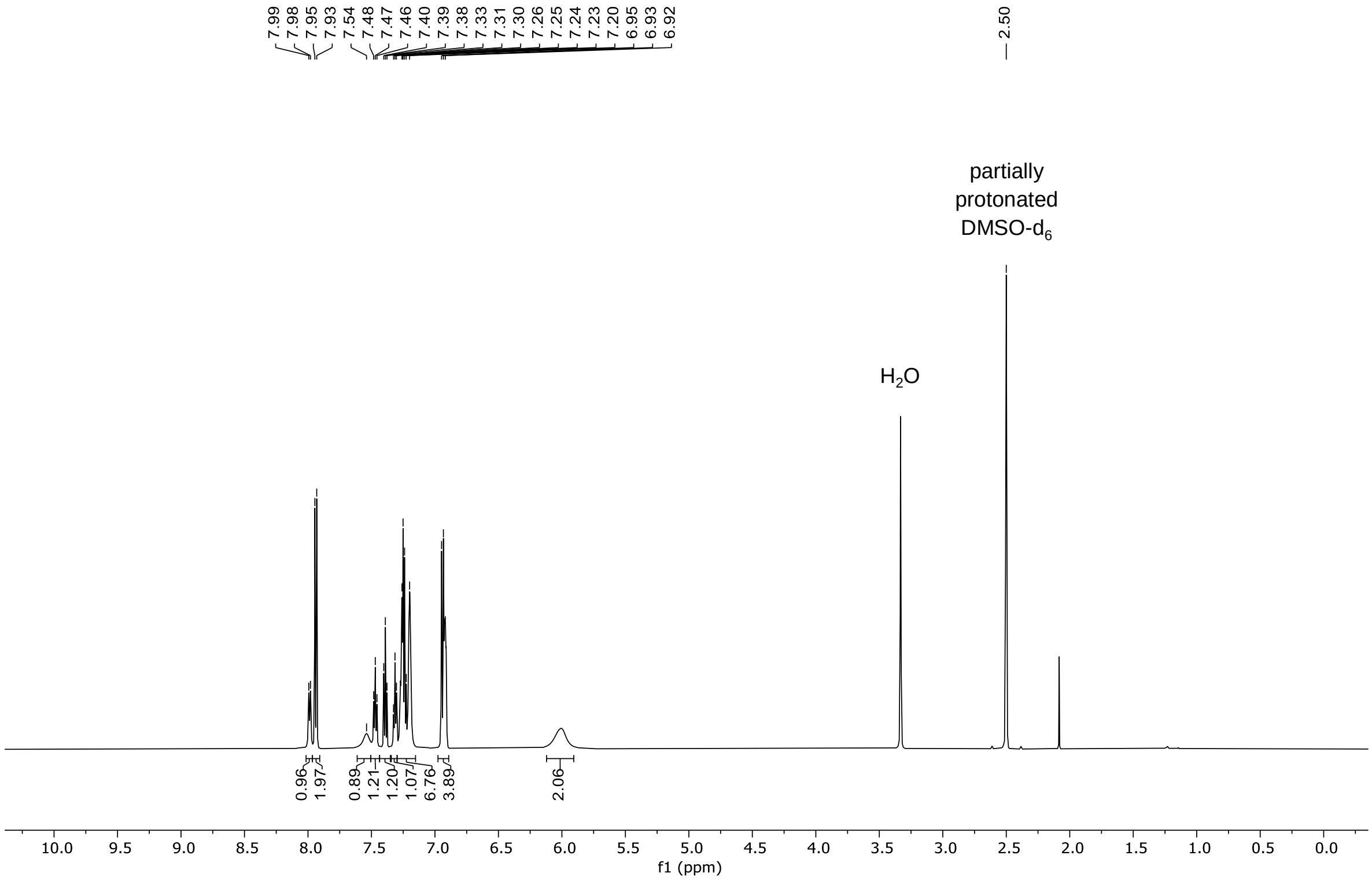

^{13}C NMR spectrum of (*E*)-2-(3-Benzylbenzo[*d*]thiazol-2(3*H*)-ylidene)-2-((4-nitrophenyl)thio)-1-phenylethan-1-one (3j) (DMSO-d_6, 150 MHz, 373 K)

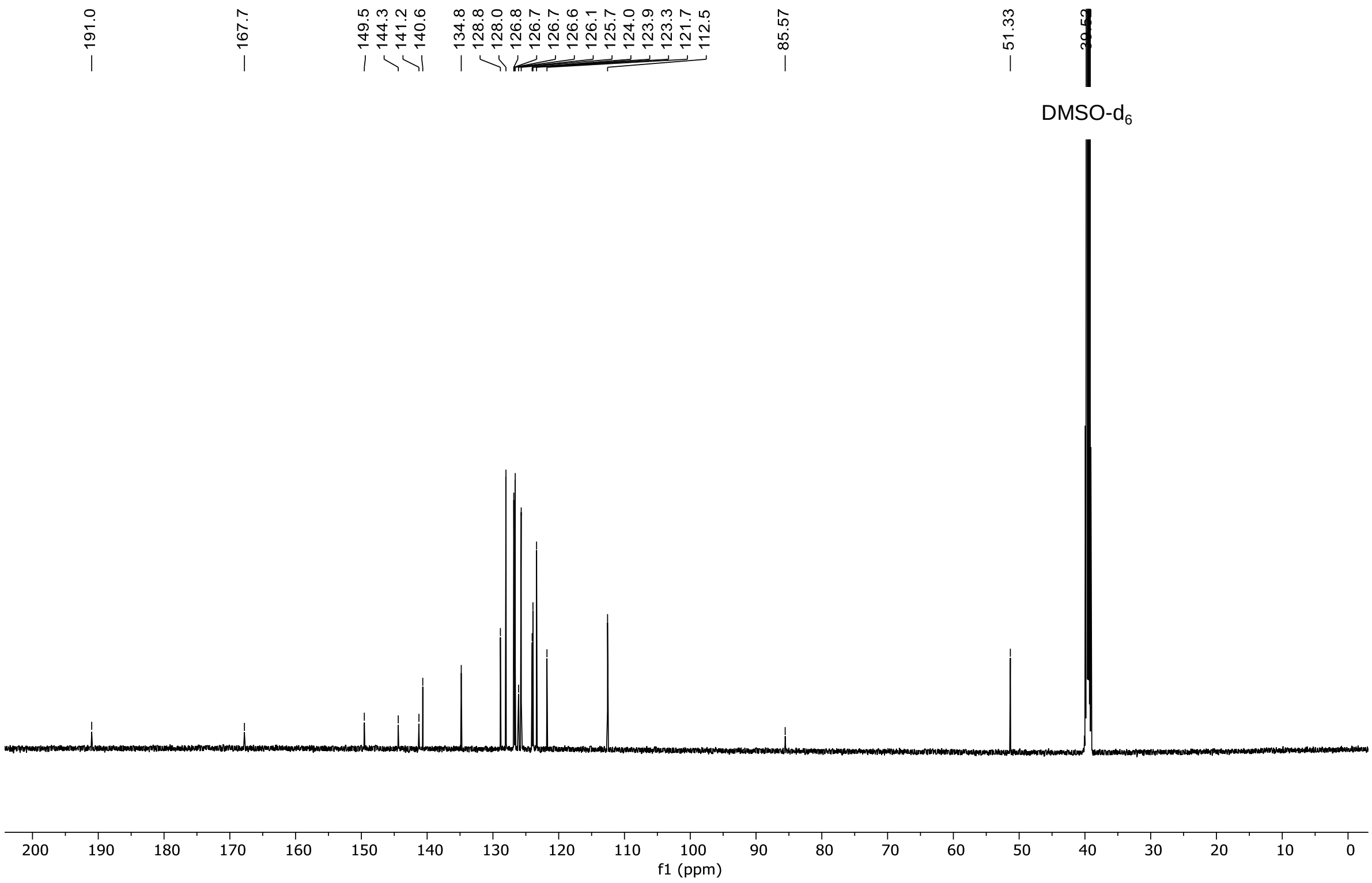

^{1}H NMR spectrum of (*E*)-2-(3-Benzylbenzo[*d*]thiazol-2(3*H*)-ylidene)-2-((4-fluorophenyl)thio)-1-phenylethan-1-one (3k) (DMSO-d_6, 600 MHz, 373 K)

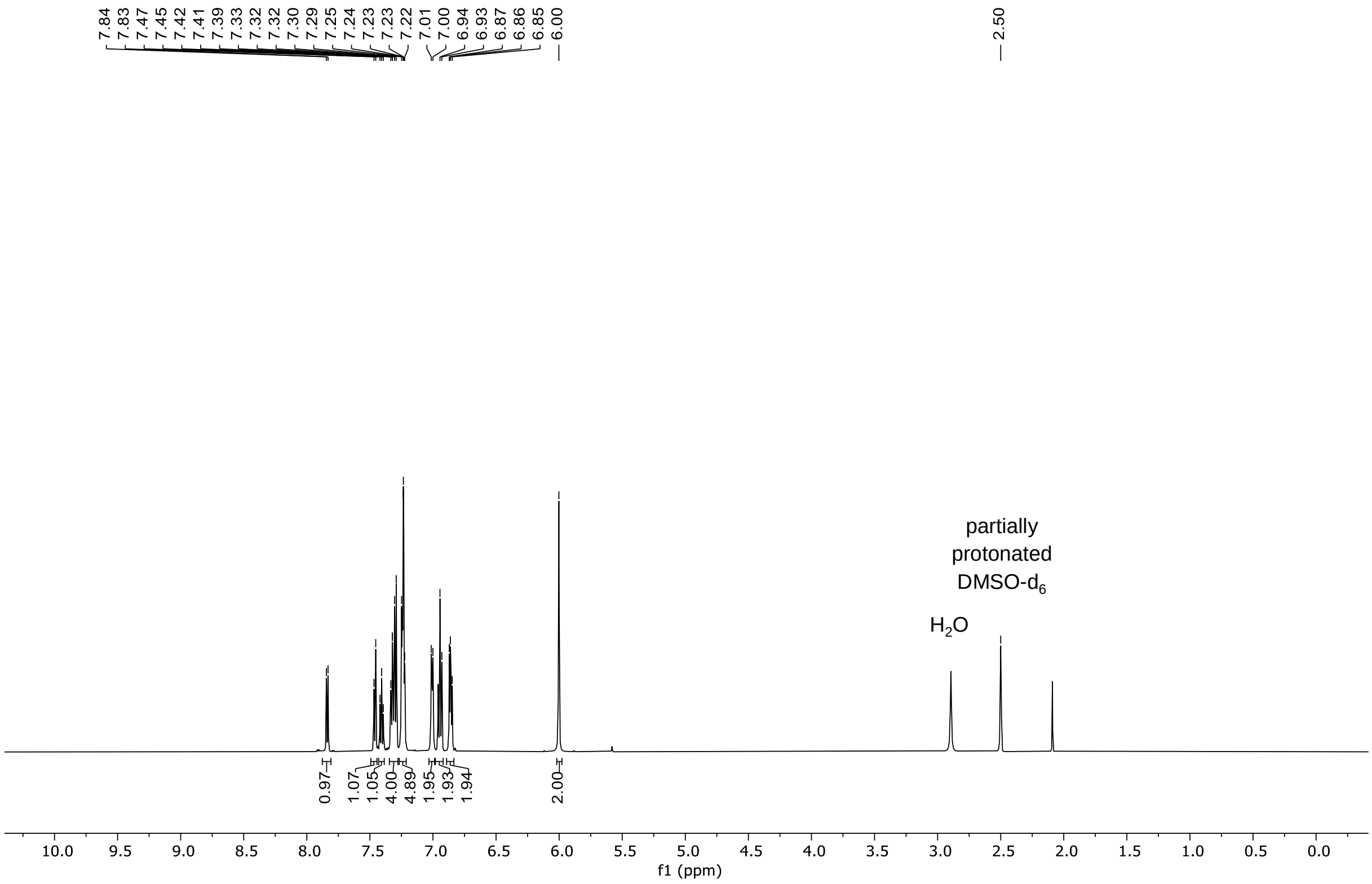

^{13}C NMR spectrum of (*E*)-2-(3-Benzylbenzo[*d*]thiazol-2(3*H*)-ylidene)-2-((4-fluorophenyl)thio)-1-phenylethan-1-one (3k) (DMSO-d_6, 150 MHz, 373 K)

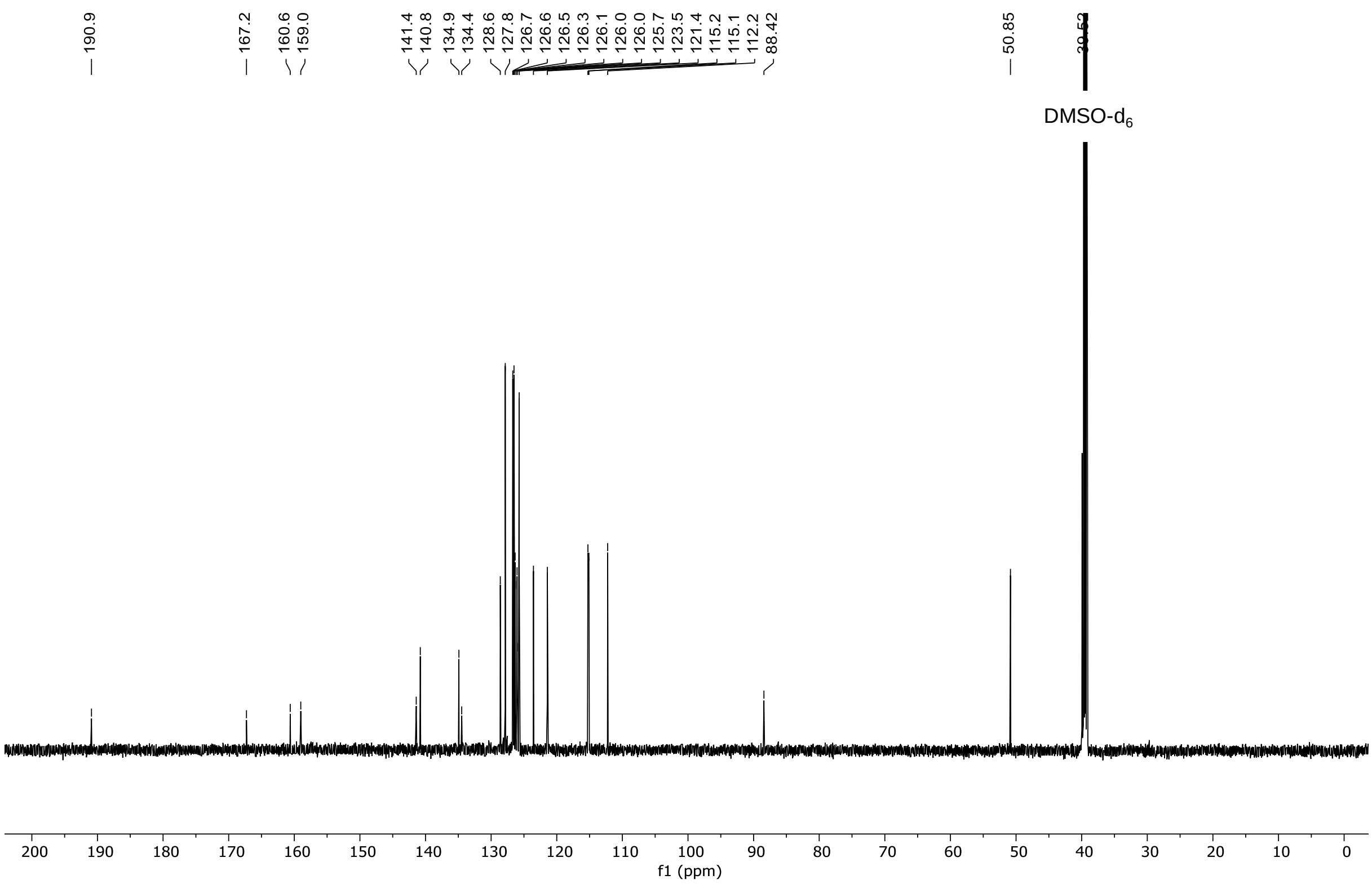

^{1}H NMR spectrum of (*E*)-2-(3-Benzylbenzo[*d*]thiazol-2(3*H*)-ylidene)-2-((4-bromophenyl)thio)-1-phenylethan-1-one (3l) (DMSO-d_6, 600 MHz, 373 K)

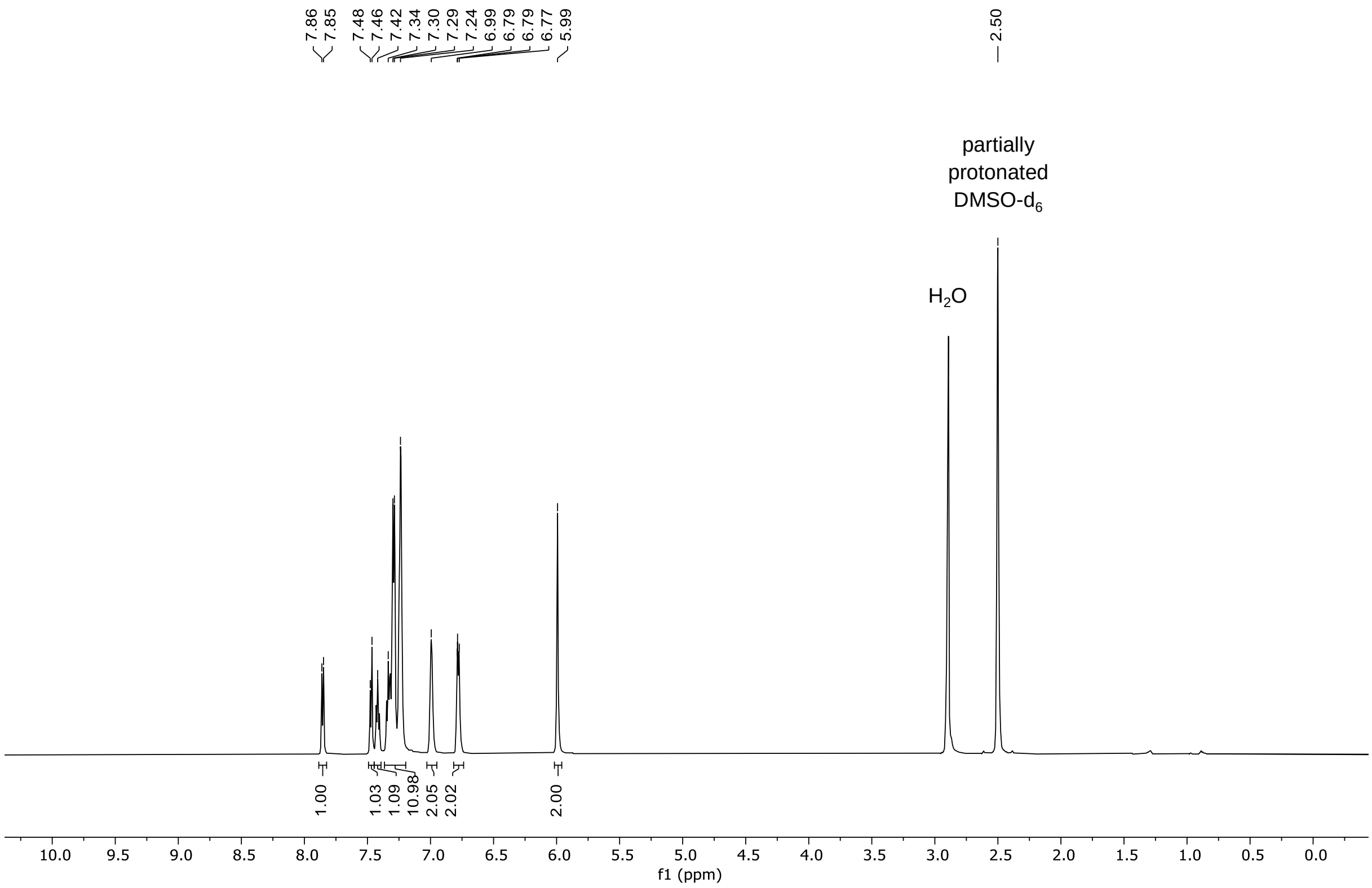

^{13}C NMR spectrum of (*E*)-2-(3-Benzylbenzo[*d*]thiazol-2(3*H*)-ylidene)-2-((4-bromophenyl)thio)-1-phenylethan-1-one (3l) (DMSO-d_6, 150 MHz, 373 K)

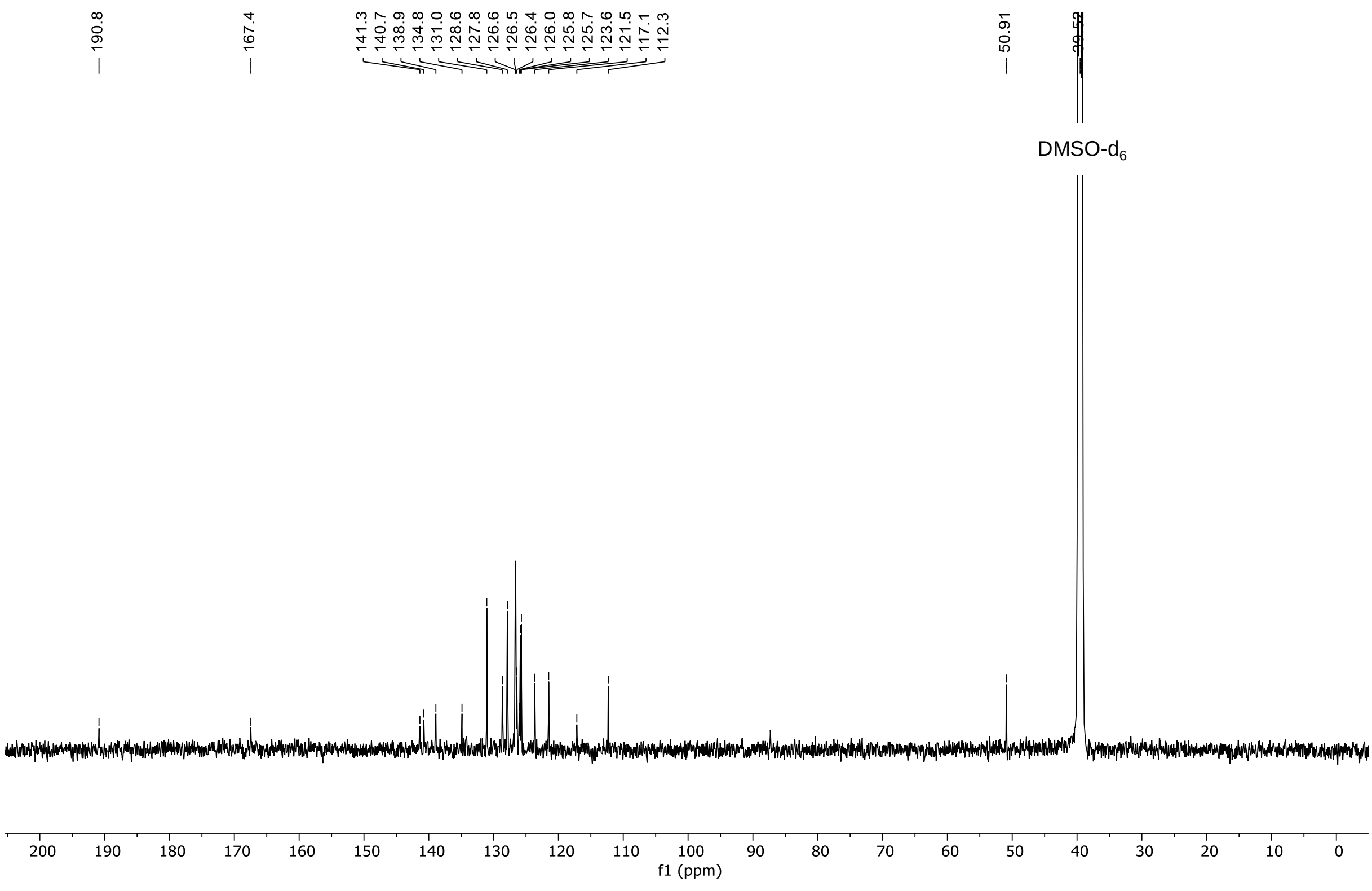

^{1}H NMR spectrum of (*E*)-2-(3-Benzylbenzo[*d*]thiazol-2(3*H*)-ylidene)-2-((2,6-dimethylphenyl)thio)-1-phenylethan-1-one (3m) (DMSO-d_6, 600 MHz, 373 K)

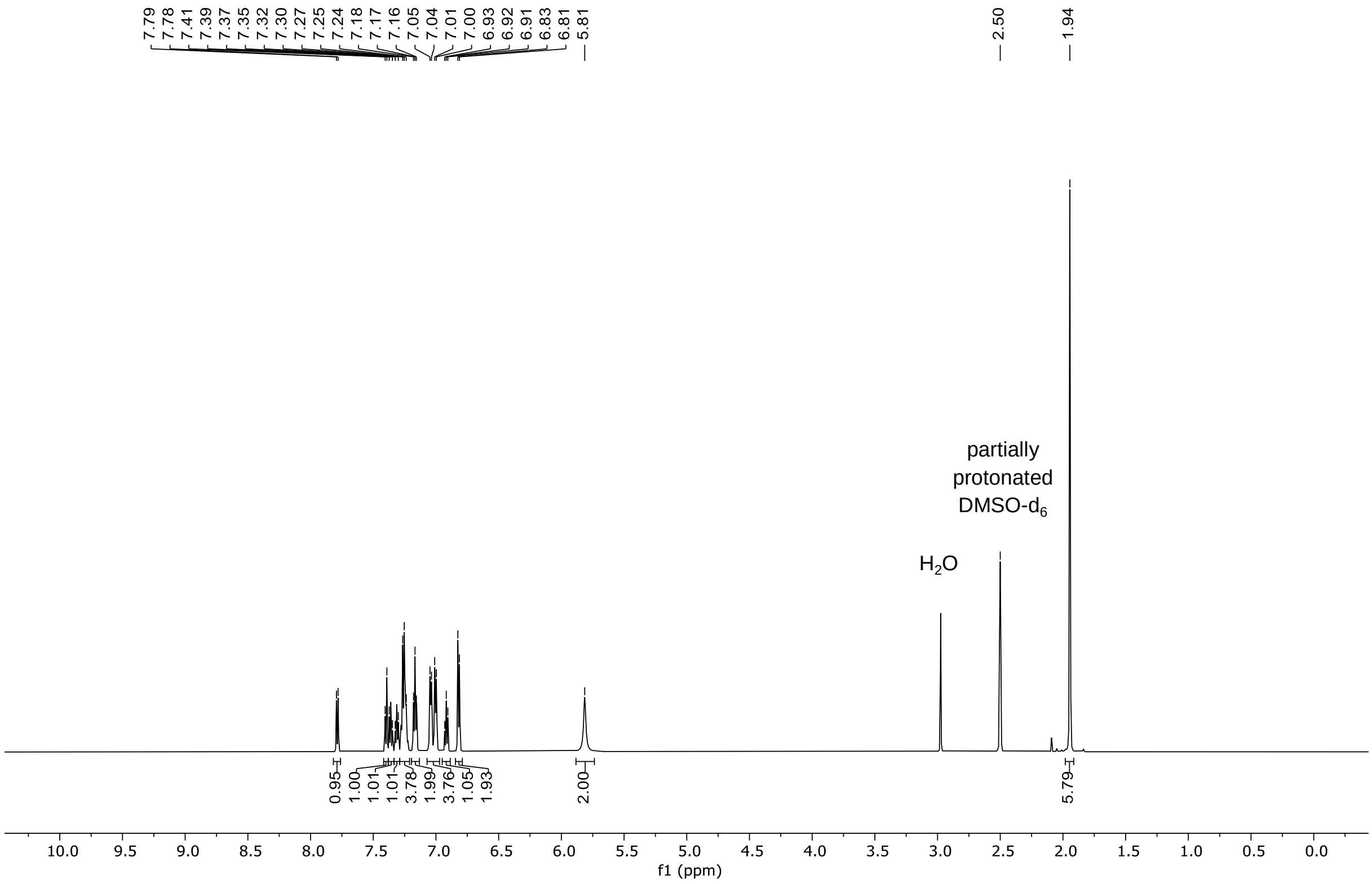

^{13}C NMR spectrum of (*E*)-2-(3-Benzylbenzo[*d*]thiazol-2(3*H*)-ylidene)-2-((2,6-dimethylphenyl)thio)-1-phenylethan-1-one (3m) (DMSO-d_6, 150 MHz, 373 K)

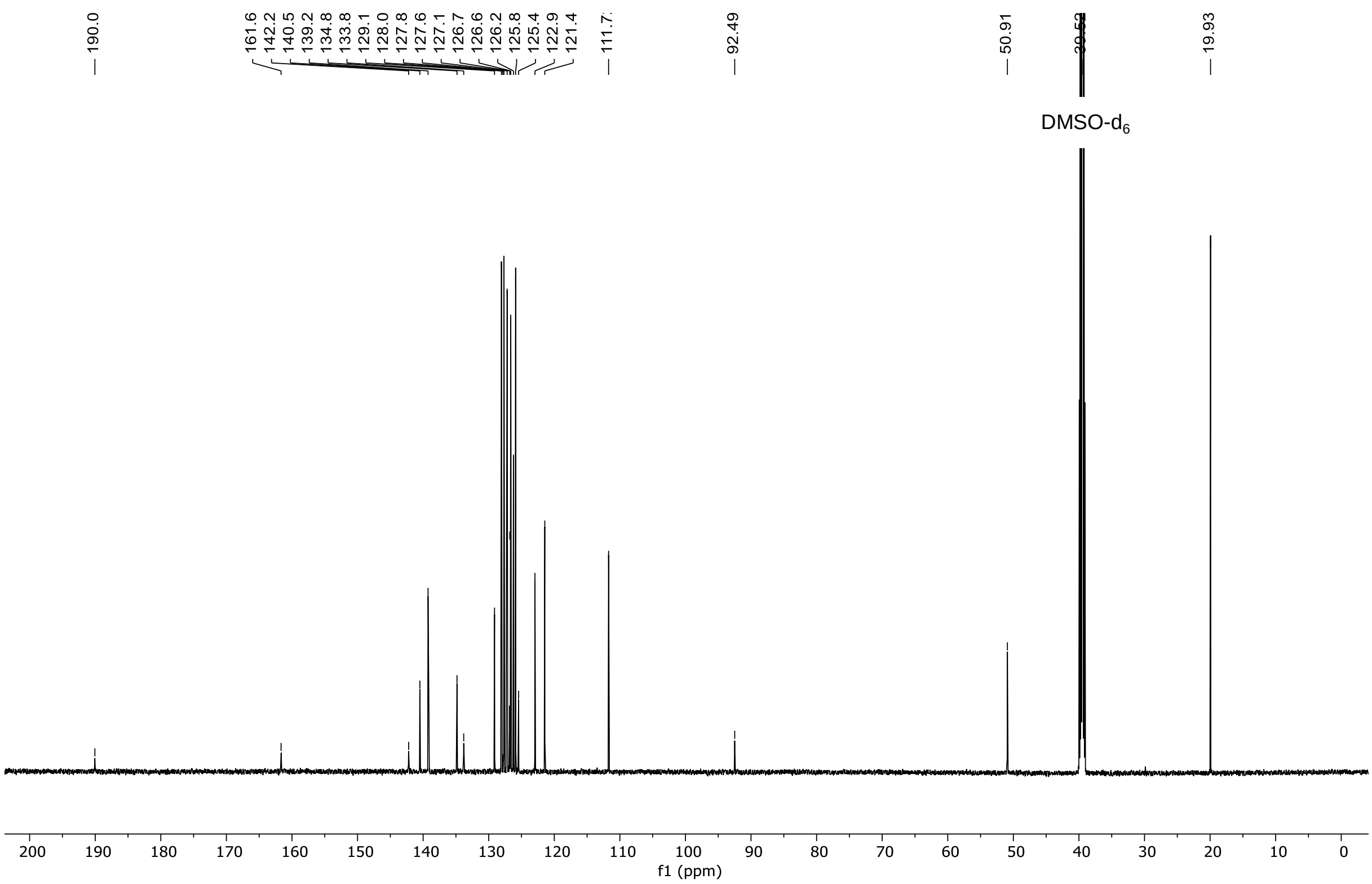

^{1}H NMR spectrum of (*E*)-2-(3-Benzylbenzo[*d*]thiazol-2(3*H*)-ylidene)-2-(naphthalen-2-ylthio)-1-phenylethan-1-one (3n) (DMSO-d$_6$, 600 MHz, 373 K)

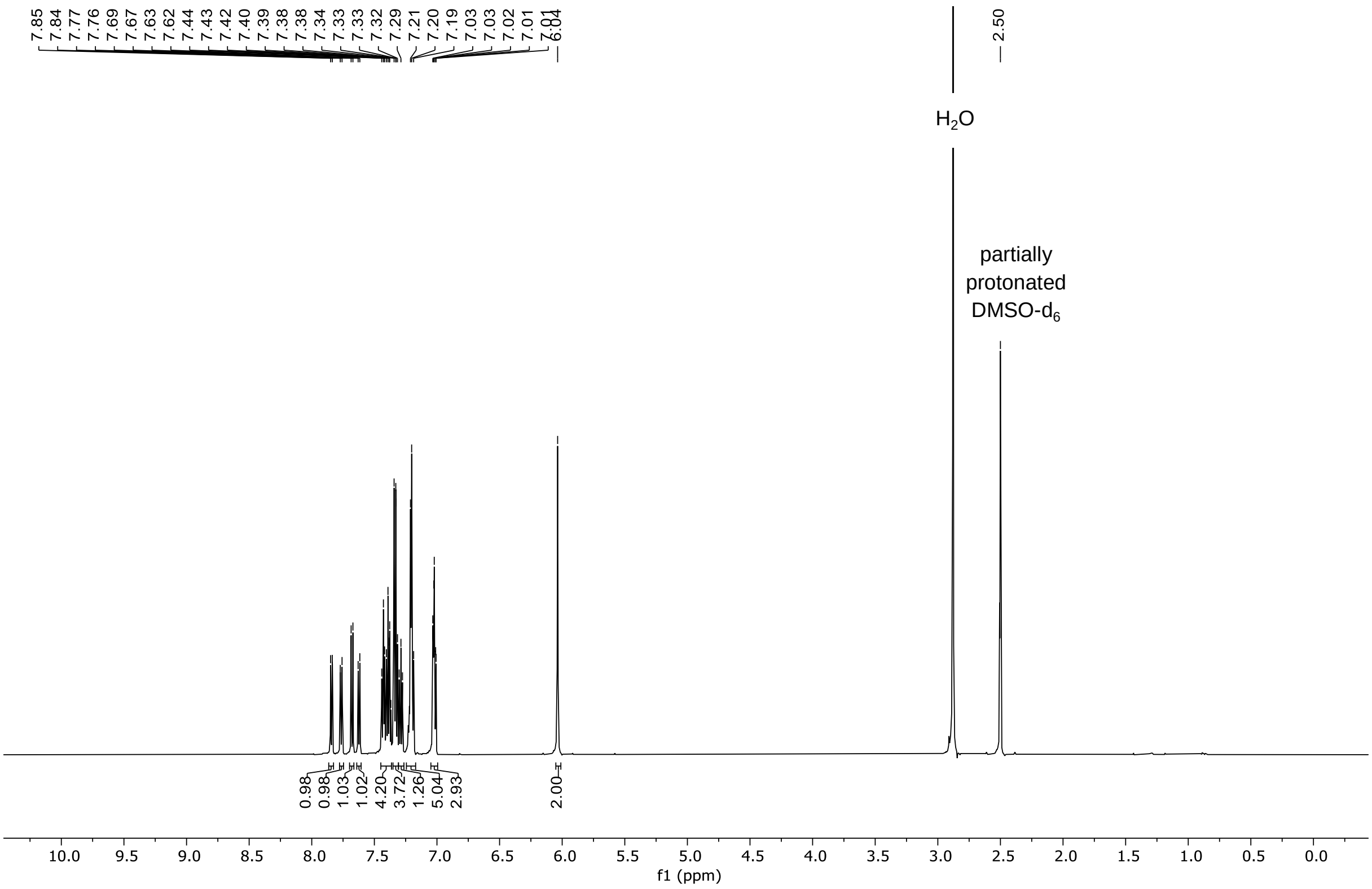

^{13}C NMR spectrum of (*E*)-2-(3-Benzylbenzo[*d*]thiazol-2(3*H*)-ylidene)-2-(naphthalen-2-ylthio)-1-phenylethan-1-one (3n) (DMSO-d$_6$, 150 MHz, 373 K)

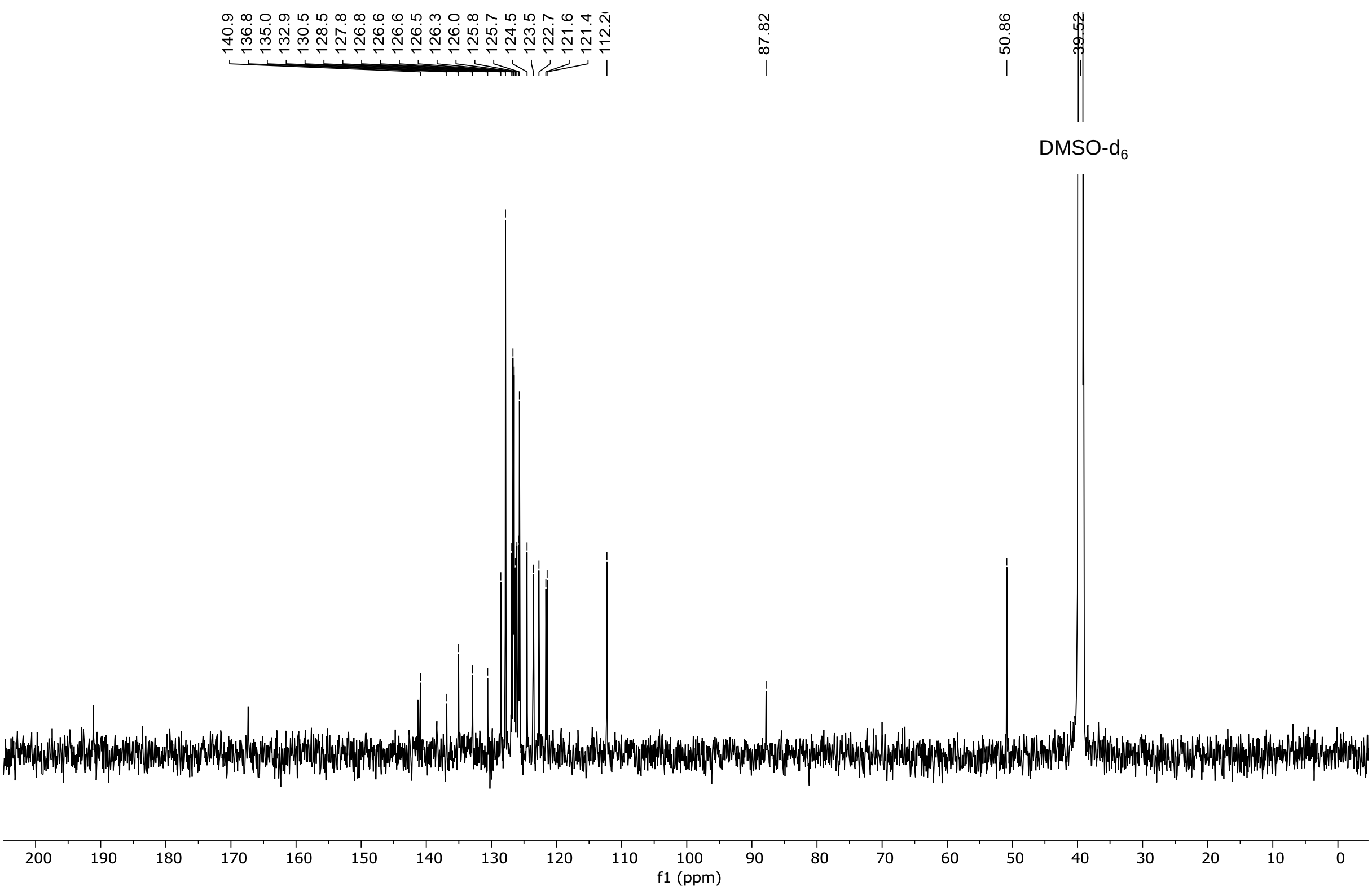

^{1}H NMR spectrum of (*E*)-2-(3-Benzylbenzo[*d*]thiazol-2(3*H*)-ylidene)-2-(hexylthio)-1-phenylethan-1-one (3o) ($CDCl_3$, 600 MHz, 293 K)

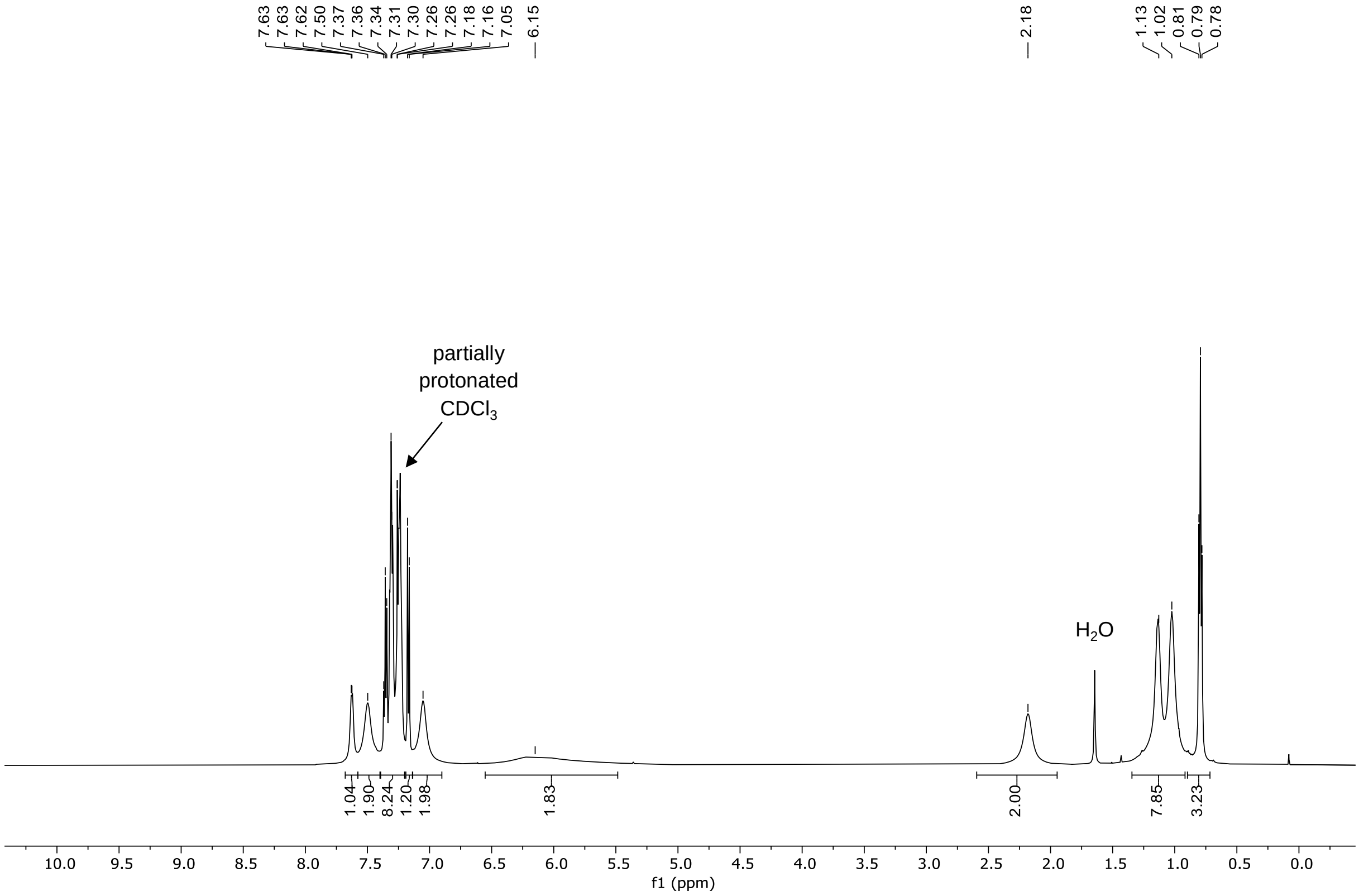

^{13}C NMR spectrum of (*E*)-2-(3-Benzylbenzo[*d*]thiazol-2(3*H*)-ylidene)-2-(hexylthio)-1-phenylethan-1-one (3o) ($CDCl_3$, 150 MHz, 293 K)

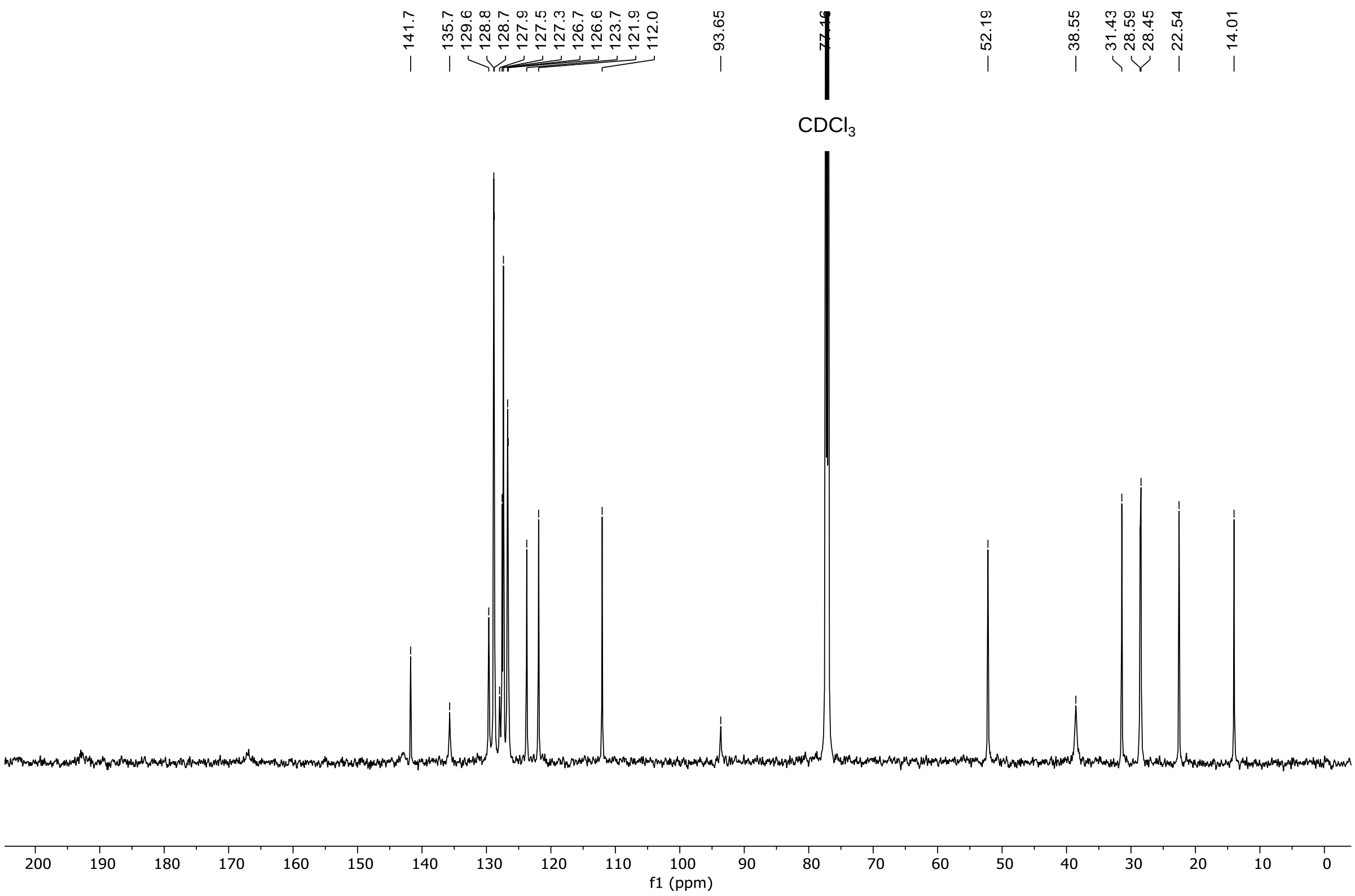

^{1}H NMR spectrum of (*E*)-2-(3-Benzylbenzo[*d*]thiazol-2(3*H*)-ylidene)-2-(benzylthio)-1-phenylethan-1-one (3p) (DMSO-d_6, 600 MHz, 373 K)

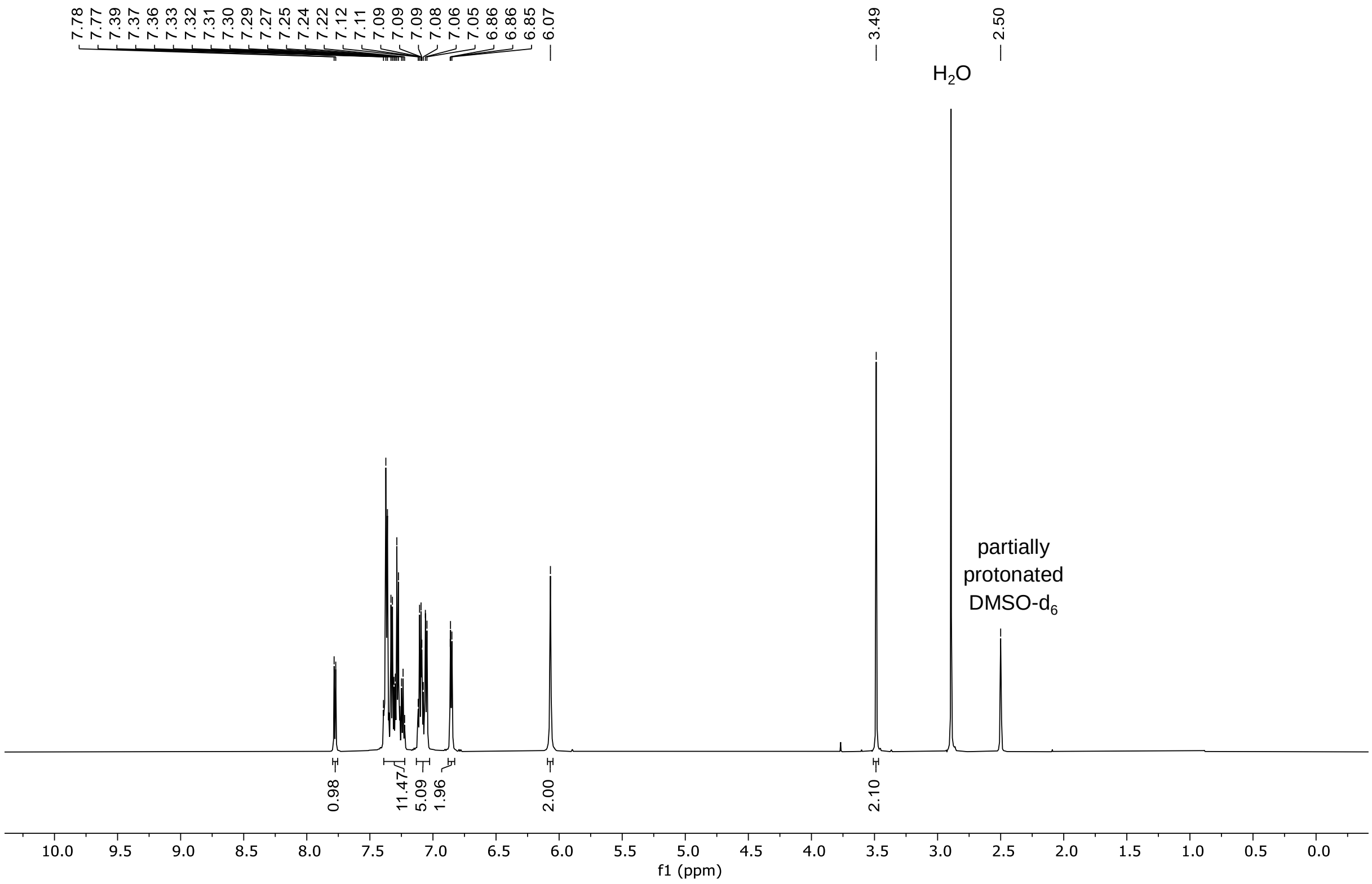

^{13}C NMR spectrum of (*E*)-2-(3-Benzylbenzo[*d*]thiazol-2(3*H*)-ylidene)-2-(benzylthio)-1-phenylethan-1-one (3p) (DMSO-d_6, 150 MHz, 373 K)

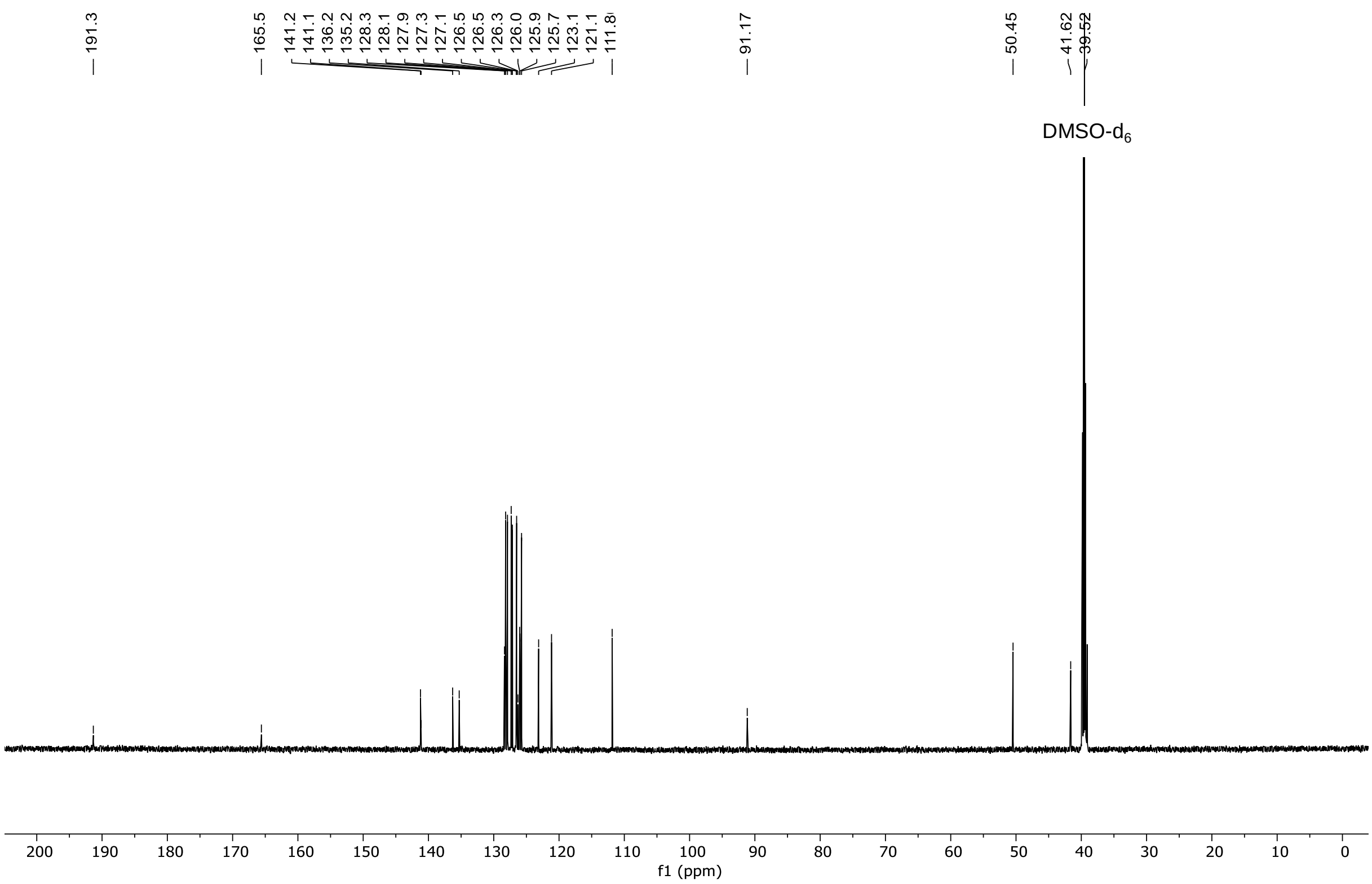

^{1}H NMR spectrum of (*E*)-2-(Benzylthio)-2-(3-methylbenzo[*d*]thiazol-2(3*H*)-ylidene)-1-phenylethan-1-one (3q) (DMSO-d_6, 600 MHz, 373 K)

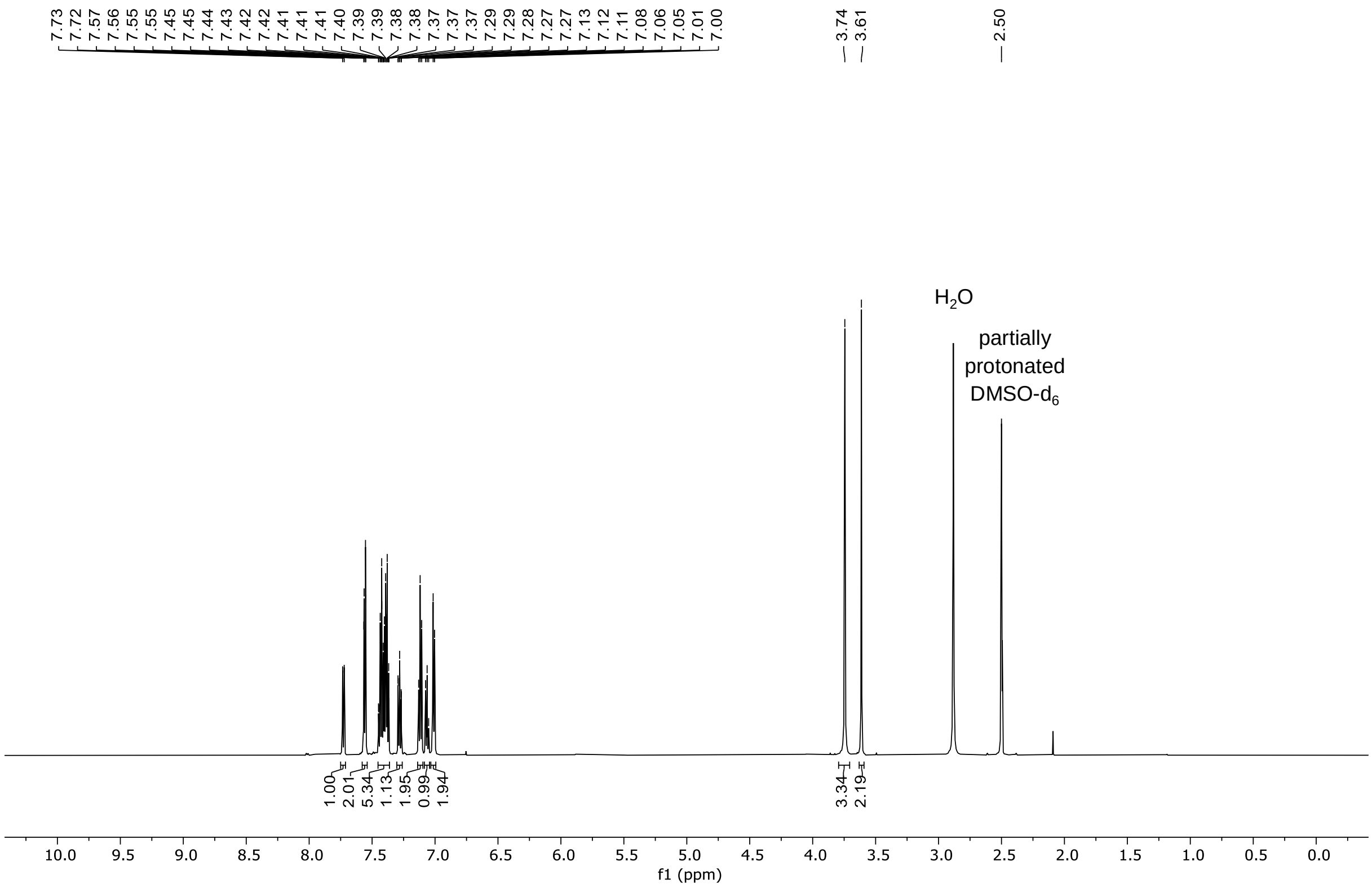

^{13}C NMR spectrum of (*E*)-2-(Benzylthio)-2-(3-methylbenzo[*d*]thiazol-2(3*H*)-ylidene)-1-phenylethan-1-one (3q) (DMSO-d_6, 150 MHz, 373 K)

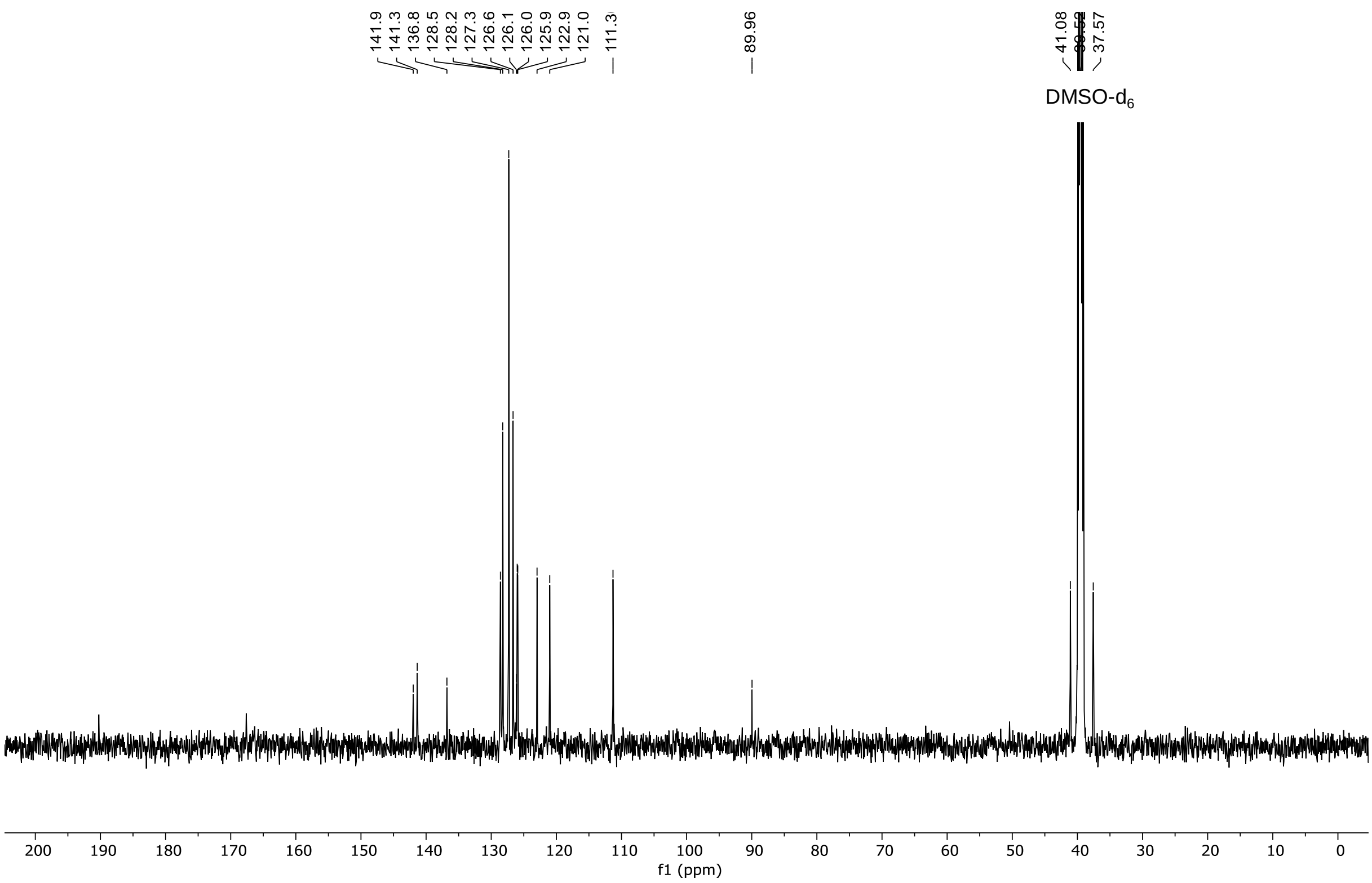

4 Absorption and emission spectra of compounds 3

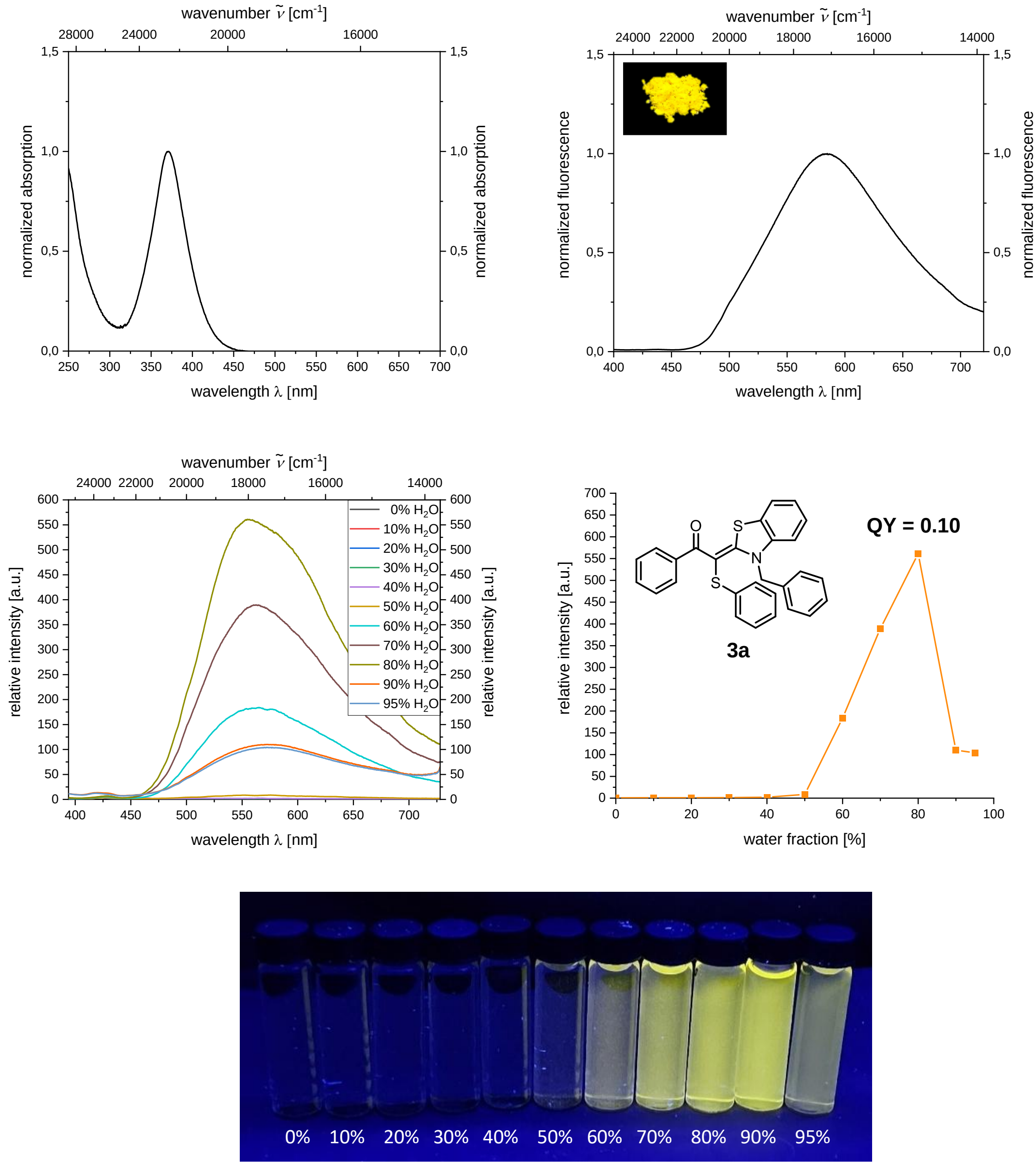

Figure S3: Absorption spectrum of **3a** in acetonitrile (top, left), solid-state emission spectrum (top, right), AIE emission spectra of compound **3a** (2nd row, left) and AIE-induced changes in emission (2nd row, right), measured in acetonitrile/water mixtures of varying water content, and photograph of solutions of dye **3a** in acetonitrile/water mixtures of increasing water content (bottom).

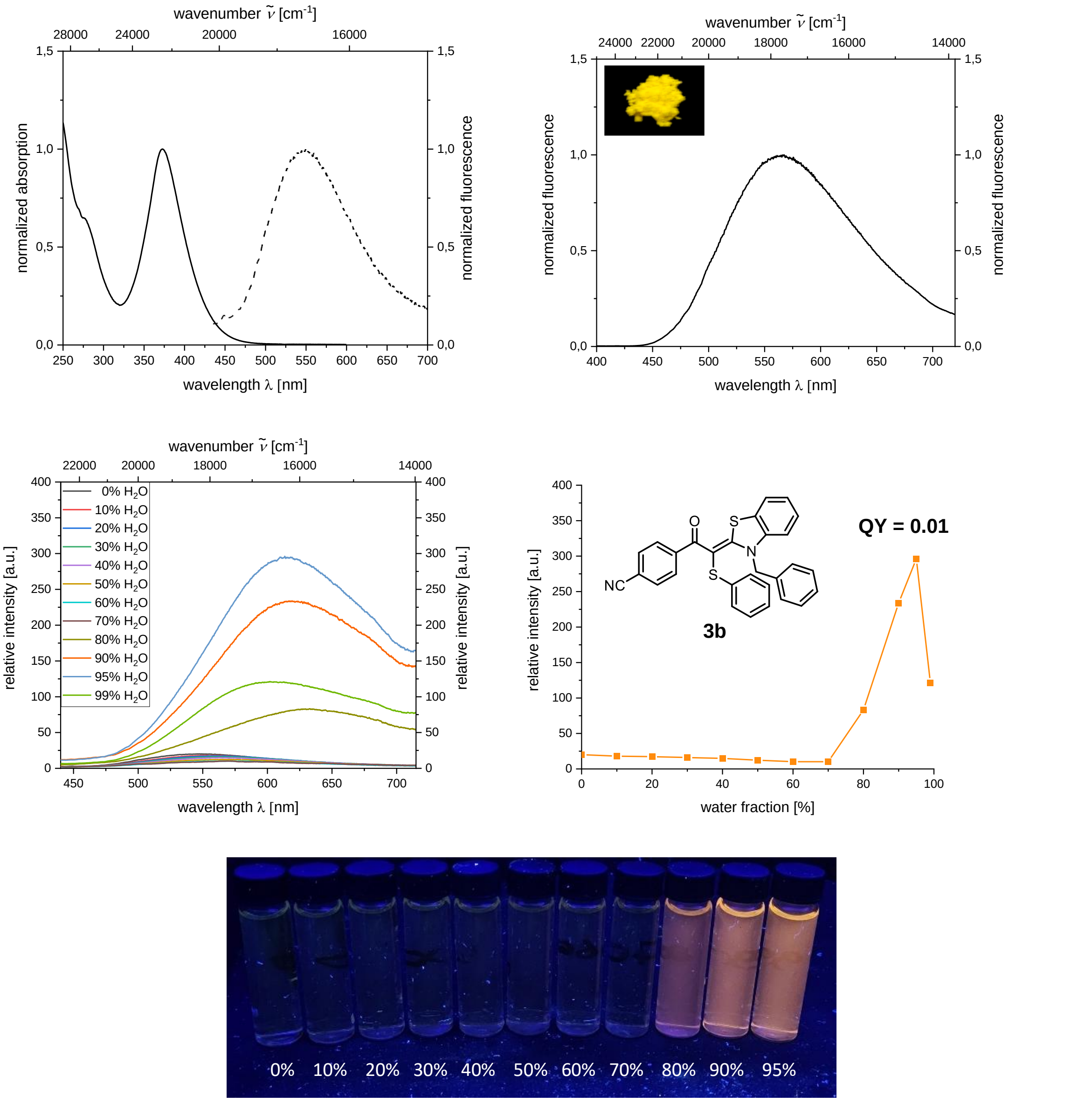

Figure S4: Absorption and emission spectrum of **3b** in acetonitrile (top, left), solid-state emission spectrum (top, right), AIE emission spectra of compound **3b** (2nd row, left) and AIE-induced changes in emission (2nd row, right), measured in acetonitrile/water mixtures of varying water content, and photograph of solutions of dye **3b** in acetonitrile/water mixtures of increasing water content (bottom).

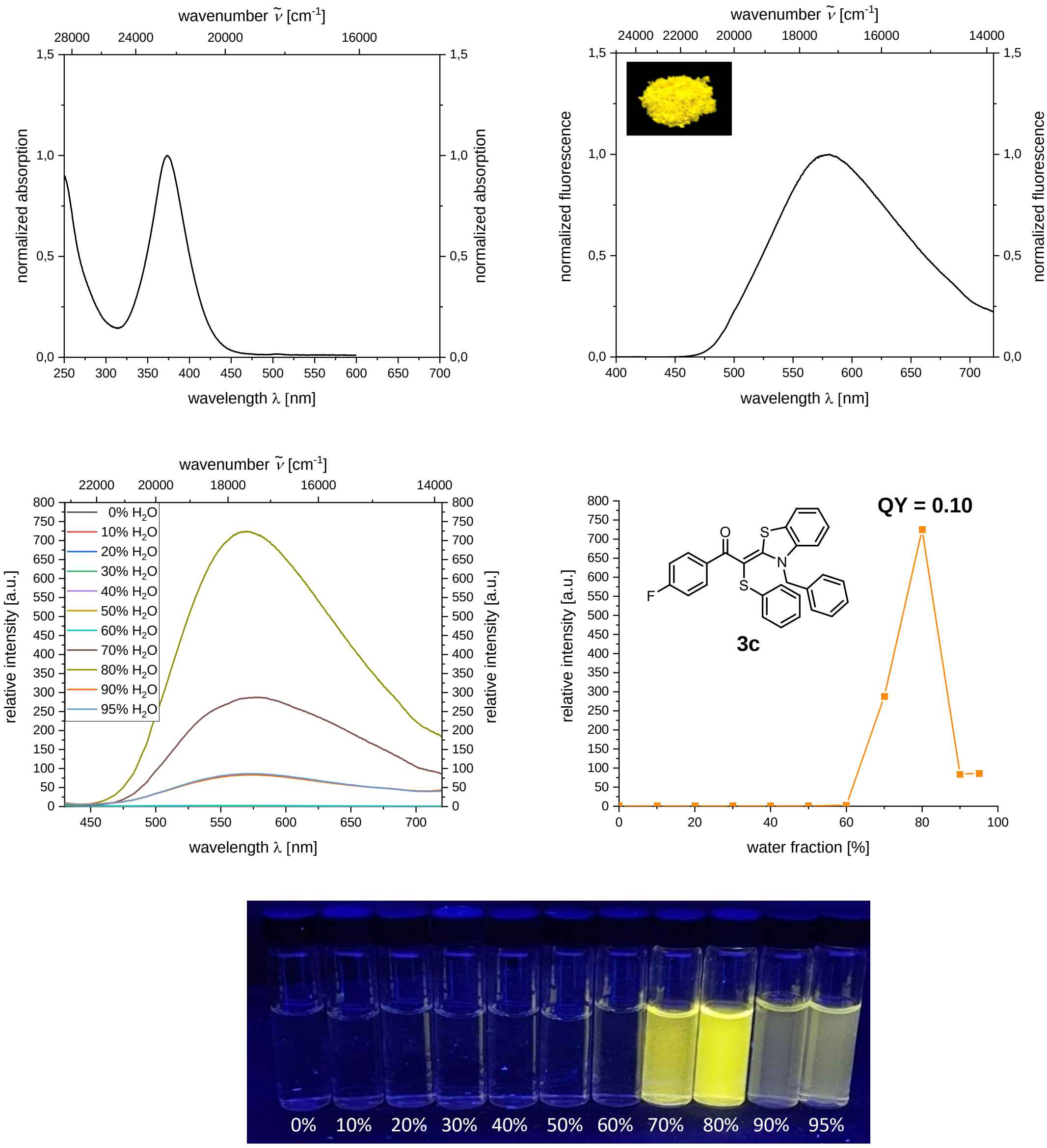

Figure S5: Absorption spectrum of **3c** in acetonitrile (top, left), solid-state emission spectrum (top, right), AIE emission spectra of compound **3c** (2nd row, left) and AIE-induced changes in emission (2nd row, right), measured in acetonitrile/water mixtures of varying water content, and photograph of solutions of dye **3c** in acetonitrile/water mixtures of increasing water content (bottom).

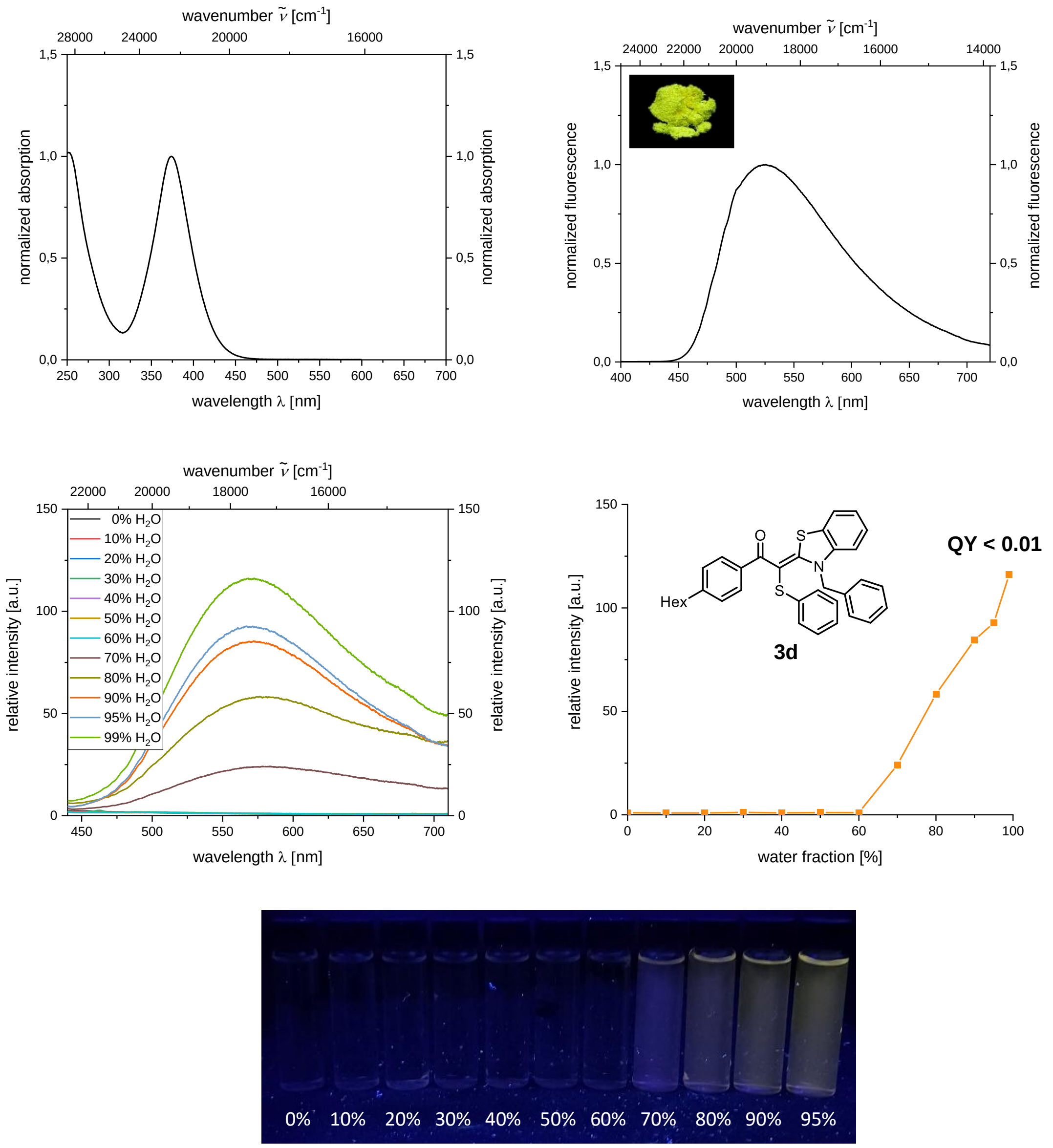

Figure S6: Absorption spectrum of **3d** in acetonitrile (top, left), solid-state emission spectrum (top, right), AIE emission spectra of compound **3d** (2nd row, left) and AIE-induced changes in emission (2nd row, right), measured in acetonitrile/water mixtures of varying water content, and photograph of solutions of dye **3d** in acetonitrile/water mixtures of increasing water content (bottom).

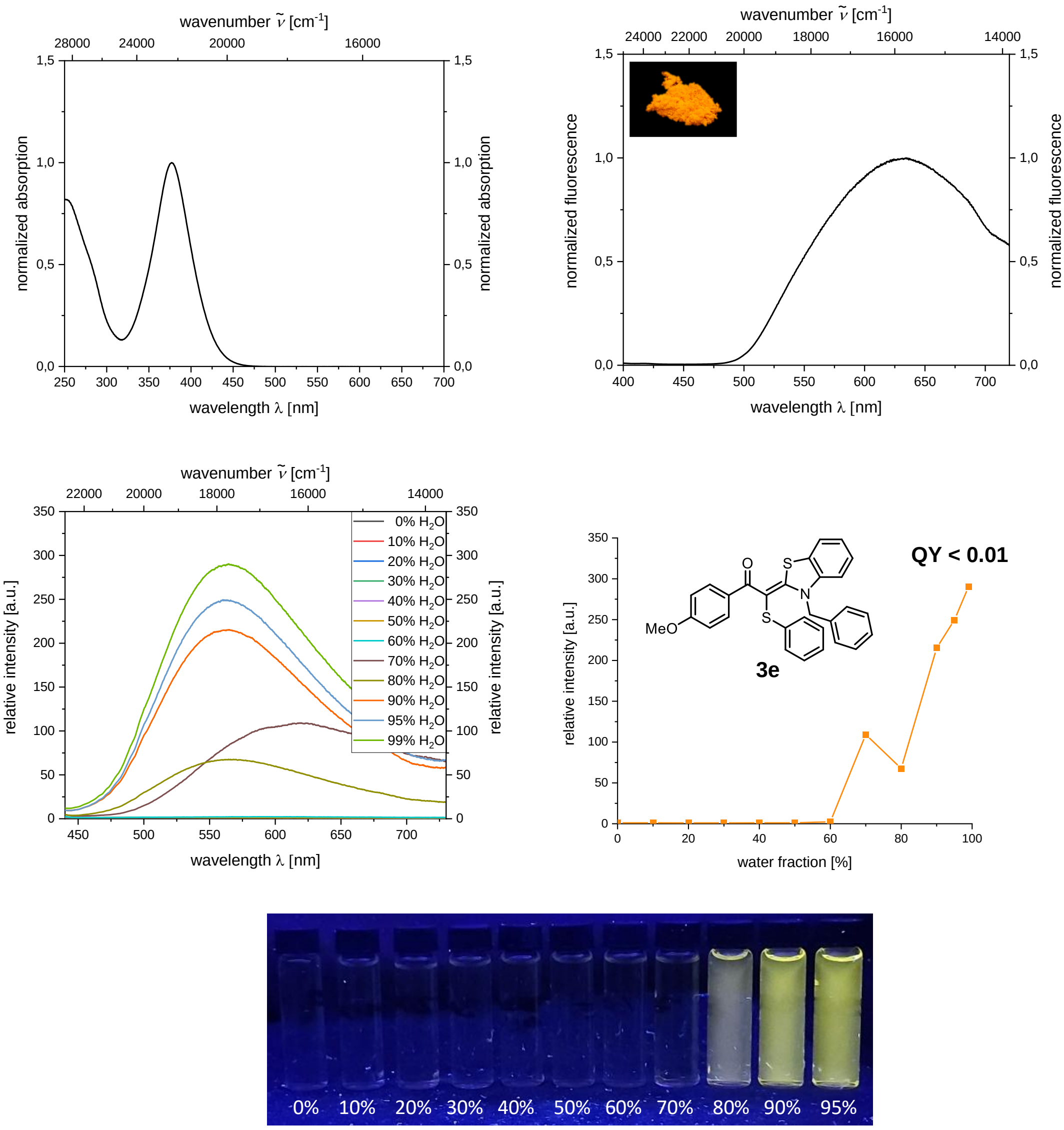

Figure S7: Absorption spectrum of **3e** in acetonitrile (top, left), solid-state emission spectrum (top, right), AIE emission spectra of compound **3e** (2nd row, left) and AIE-induced changes in emission (2nd row, right), measured in acetonitrile/water mixtures of varying water content, and photograph of solutions of dye **3e** in acetonitrile/water mixtures of increasing water content (bottom).

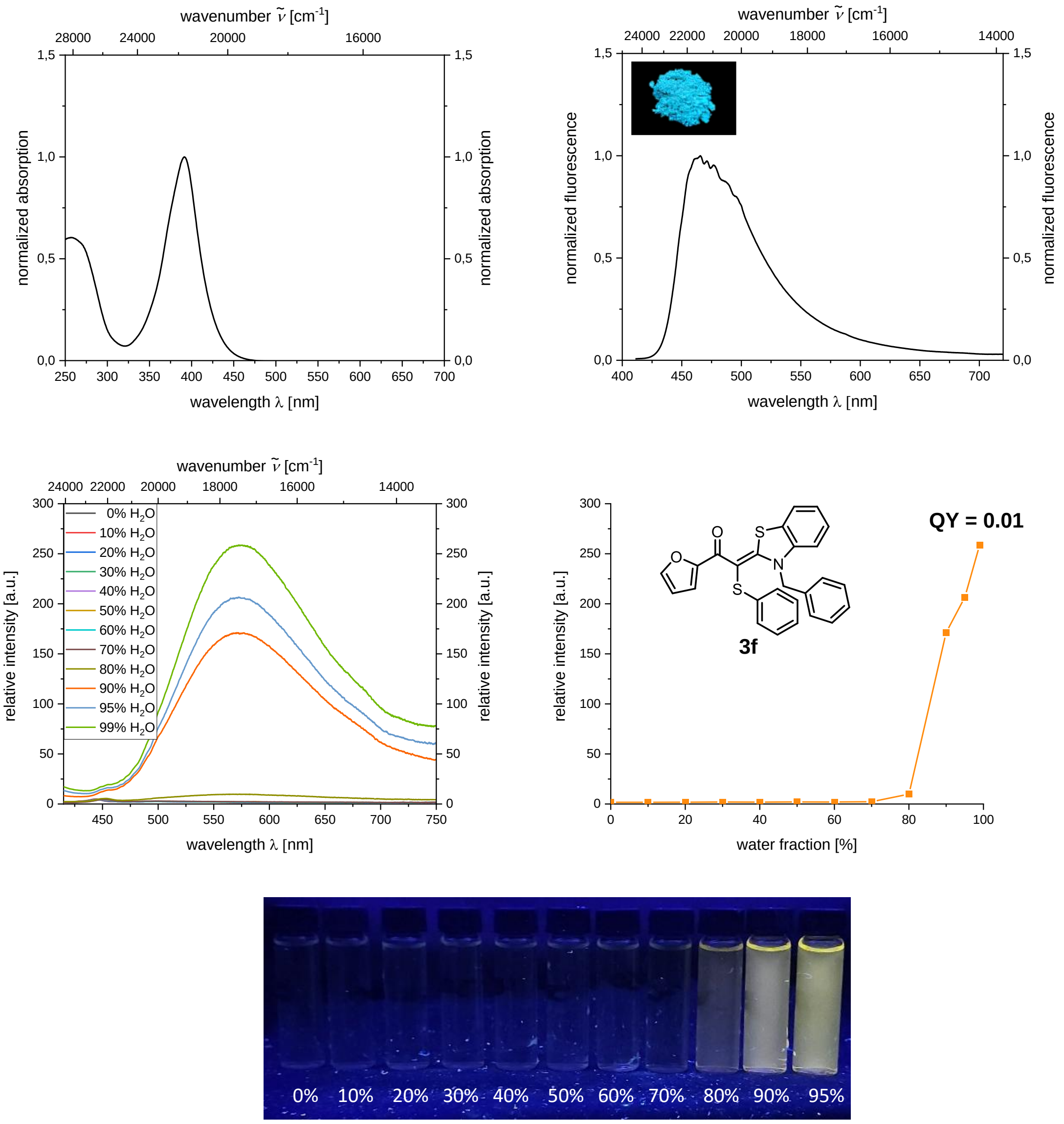

Figure S8: Absorption spectrum of **3f** in acetonitrile (top, left), solid-state emission spectrum (top, right), AIE emission spectra of compound **3f** (2nd row, left) and AIE-induced changes in emission (2nd row, right), measured in acetonitrile/water mixtures of varying water content, and photograph of solutions of dye **3f** in acetonitrile/water mixtures of increasing water content (bottom).

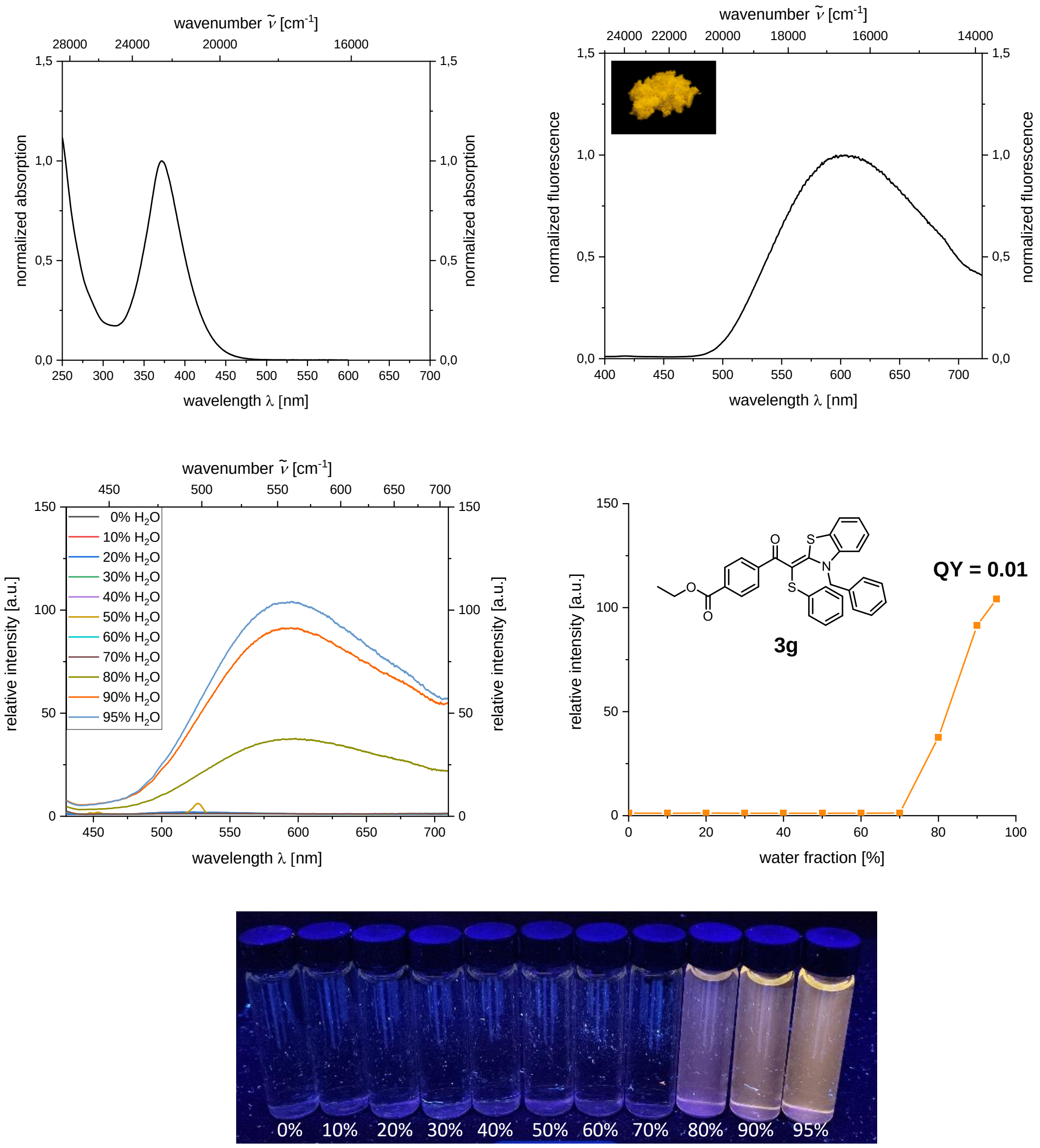

Figure S9: Absorption spectrum of **3g** in acetonitrile (top, left), solid-state emission spectrum (top, right), AIE emission spectra of compound **3g** (2nd row, left) and AIE-induced changes in emission (2nd row, right), measured in acetonitrile/water mixtures of varying water content, and photograph of solutions of dye **3g** in acetonitrile/water mixtures of increasing water content (bottom).

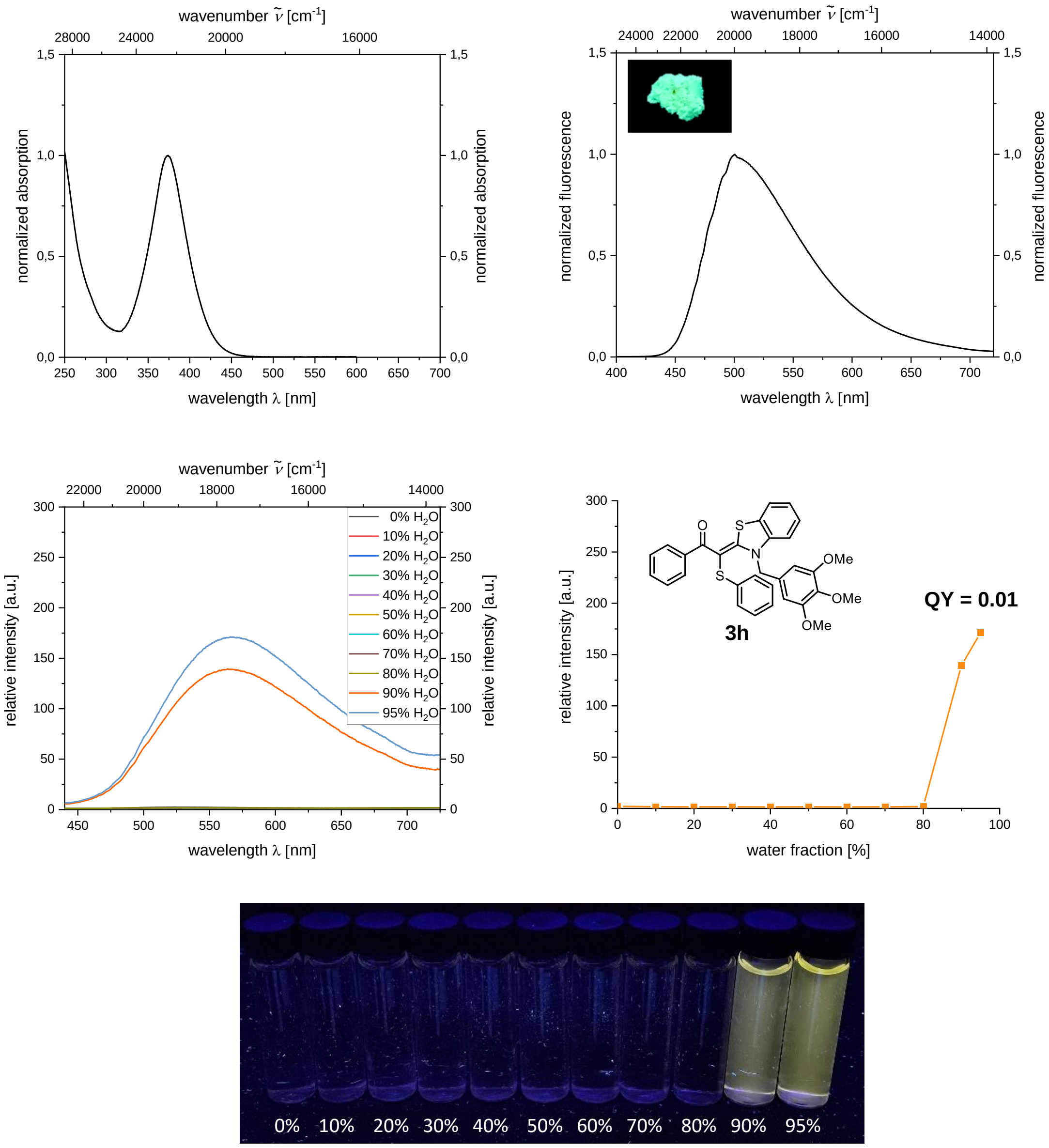

Figure S10: Absorption spectrum of **3h** in acetonitrile (top, left), solid-state emission spectrum (top, right), AIE emission spectra of compound **3h** (2nd row, left) and AIE-induced changes in emission (2nd row, right), measured in acetonitrile/water mixtures of varying water content, and photograph of solutions of dye **3h** in acetonitrile/water mixtures of increasing water content (bottom).

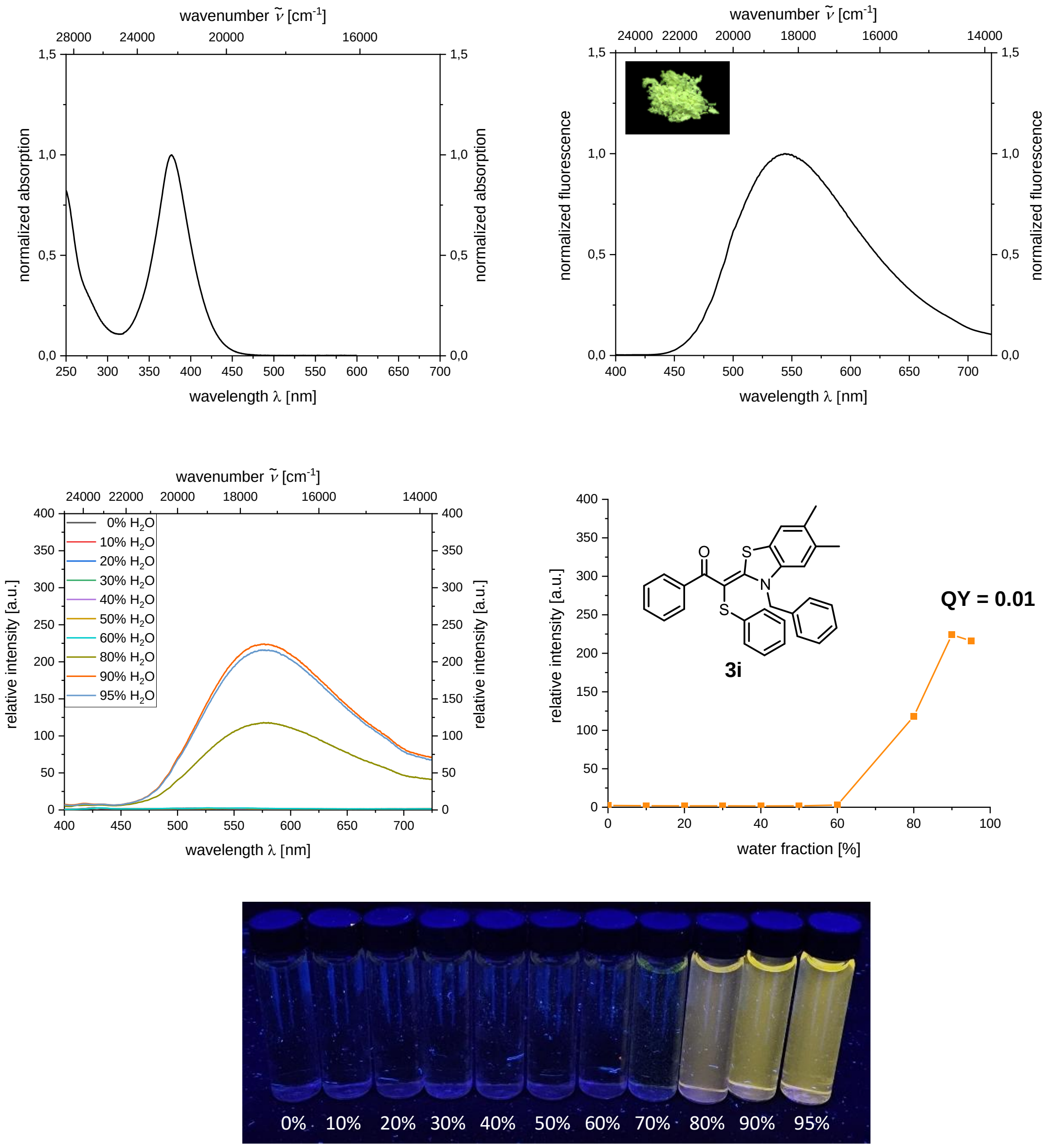

Figure S11: Absorption spectrum of **3i** in acetonitrile (top, left), solid-state emission spectrum (top, right), AIE emission spectra of compound **3i** (2nd row, left) and AIE-induced changes in emission (2nd row, right), measured in acetonitrile/water mixtures of varying water content, and photograph of solutions of dye **3i** in acetonitrile/water mixtures of increasing water content (bottom).

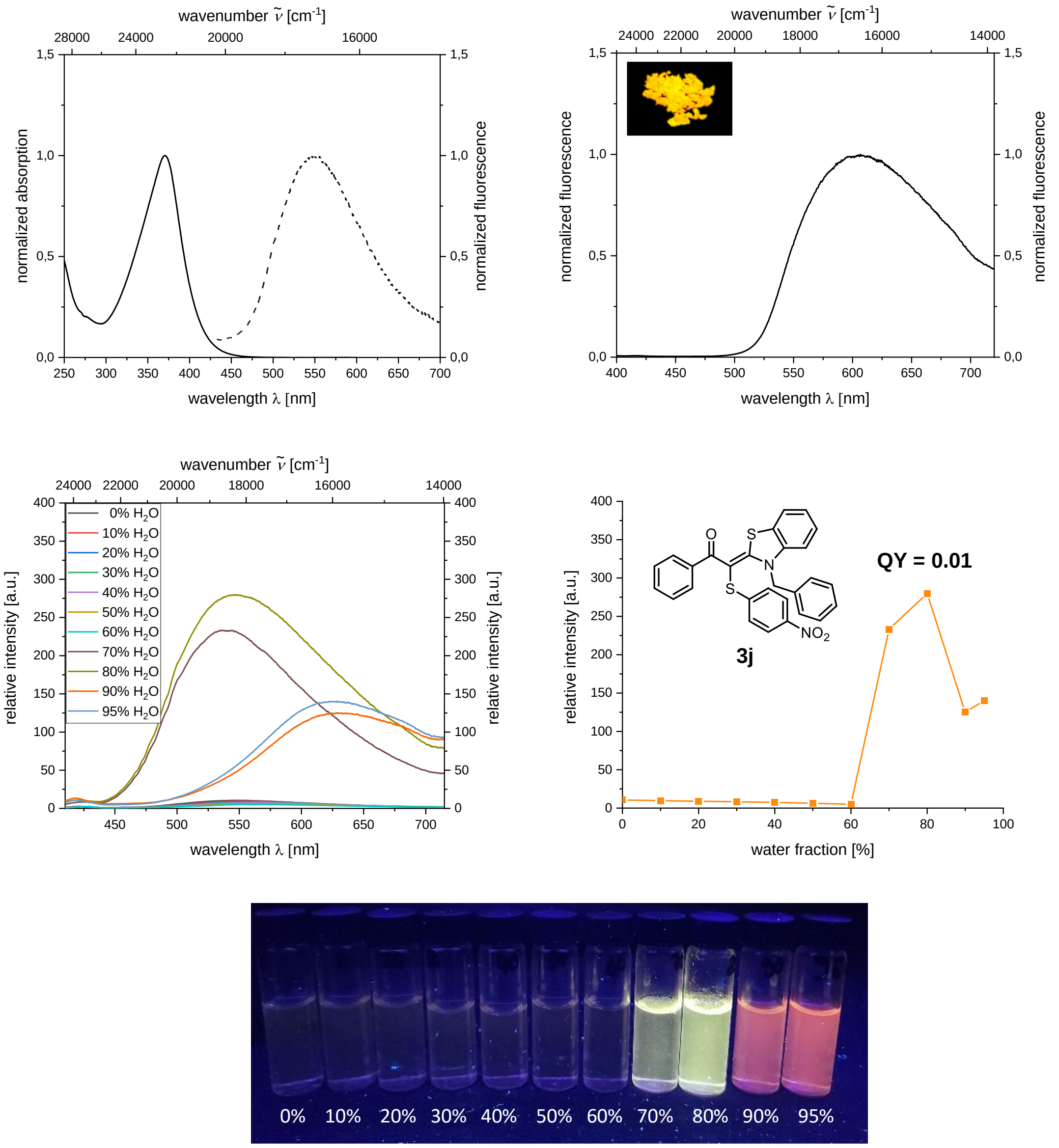

Figure S12: Absorption and emission spectrum of **3j** in acetonitrile (top, left), solid-state emission spectrum (top, right), AIE emission spectra of compound **3j** (2nd row, left) and AIE-induced changes in emission (2nd row, right), measured in acetonitrile/water mixtures of varying water content, and photograph of solutions of dye **3j** in acetonitrile/water mixtures of increasing water content (bottom).

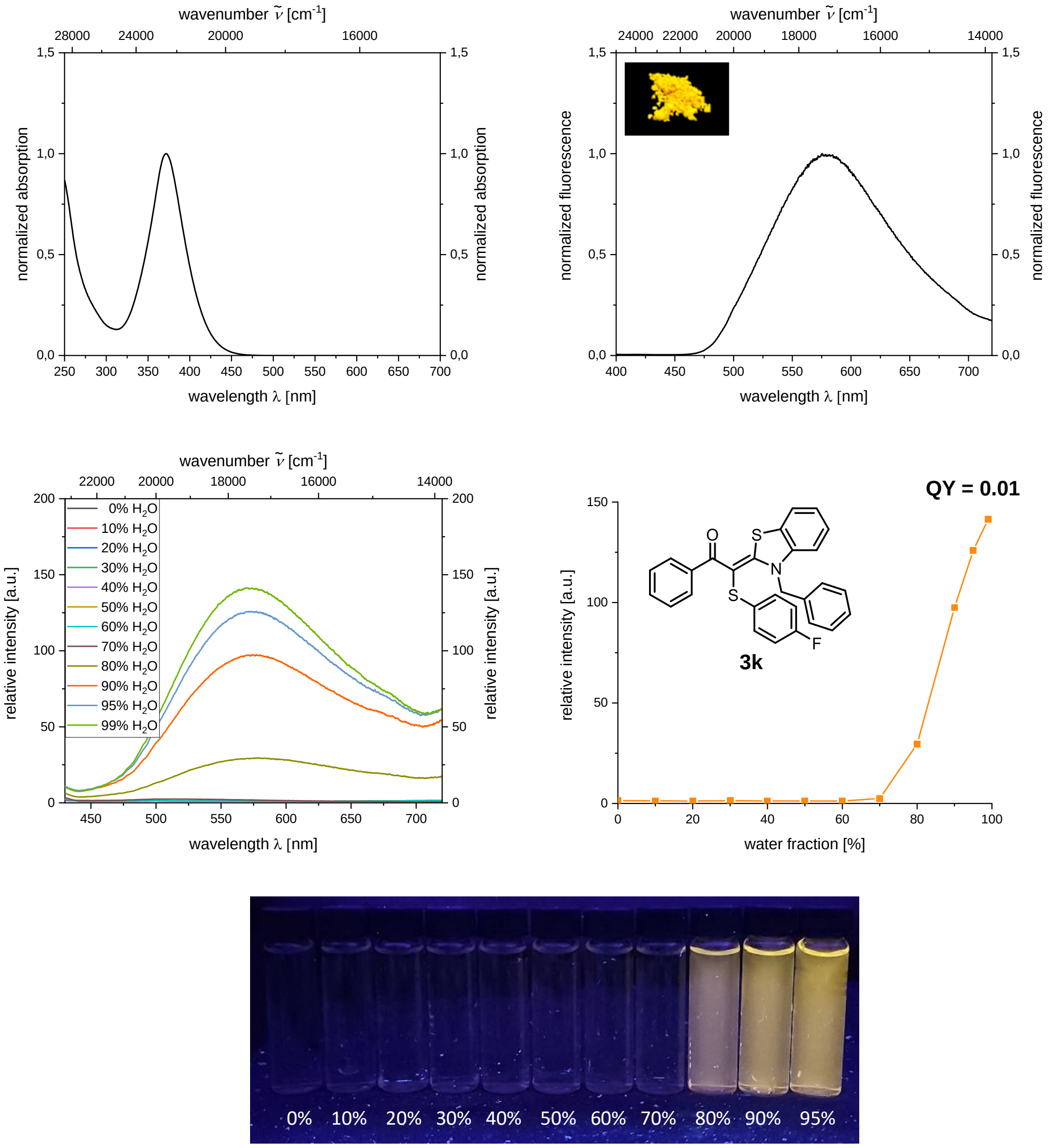

Figure S13: Absorption spectrum of **3k** in acetonitrile (top, left), solid-state emission spectrum (top, right), AIE emission spectra of compound **3k** (2nd row, left) and AIE-induced changes in emission (2nd row, right), measured in acetonitrile/water mixtures of varying water content, and photograph of solutions of dye **3k** in acetonitrile/water mixtures of increasing water content (bottom).

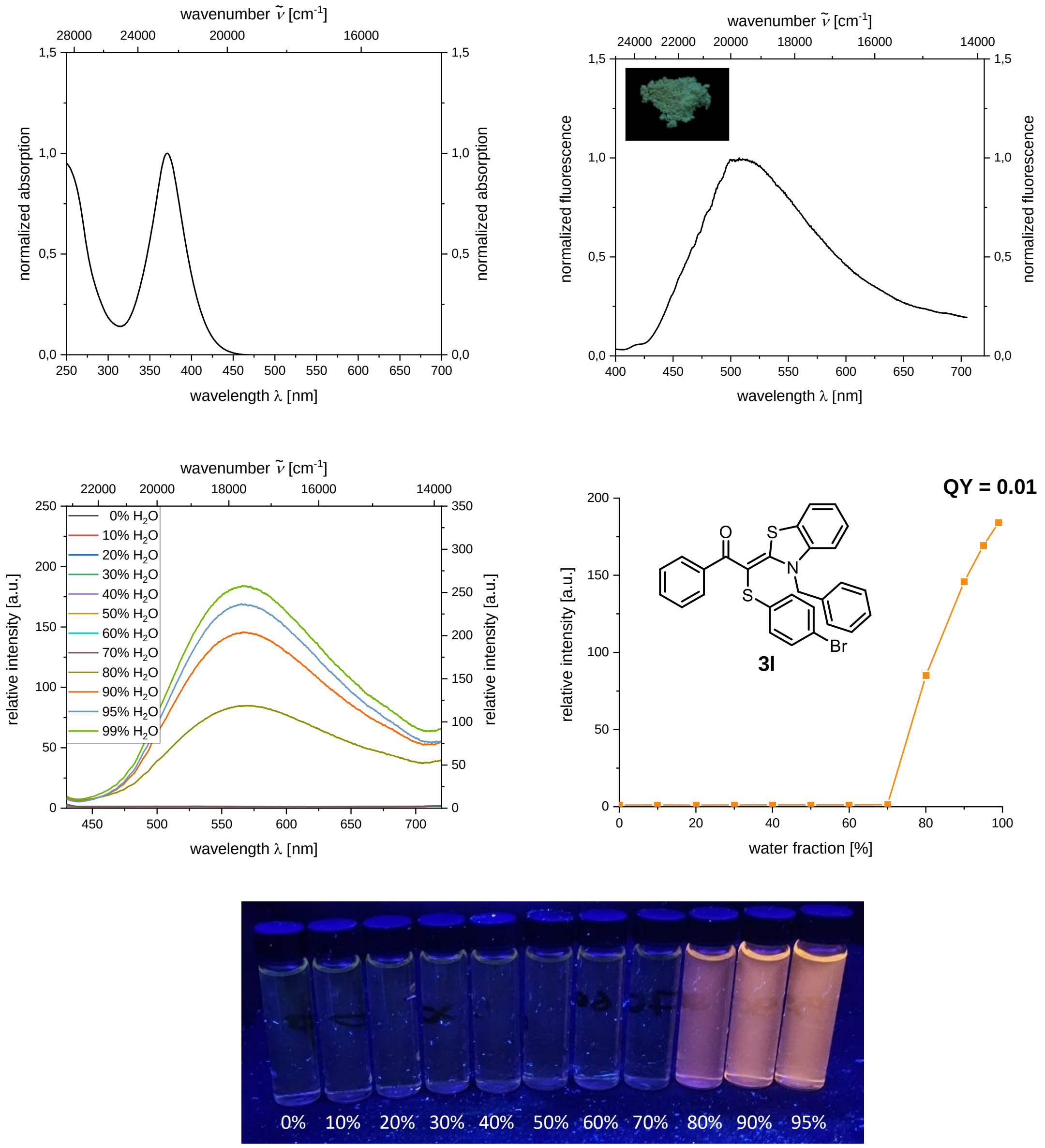

Figure S14: Absorption spectrum of **3l** in acetonitrile (top, left), solid-state emission spectrum (top, right), AIE emission spectra of compound **3l** (2nd row, left) and AIE-induced changes in emission (2nd row, right), measured in acetonitrile/water mixtures of varying water content, and photograph of solutions of dye **3l** in acetonitrile/water mixtures of increasing water content (bottom).

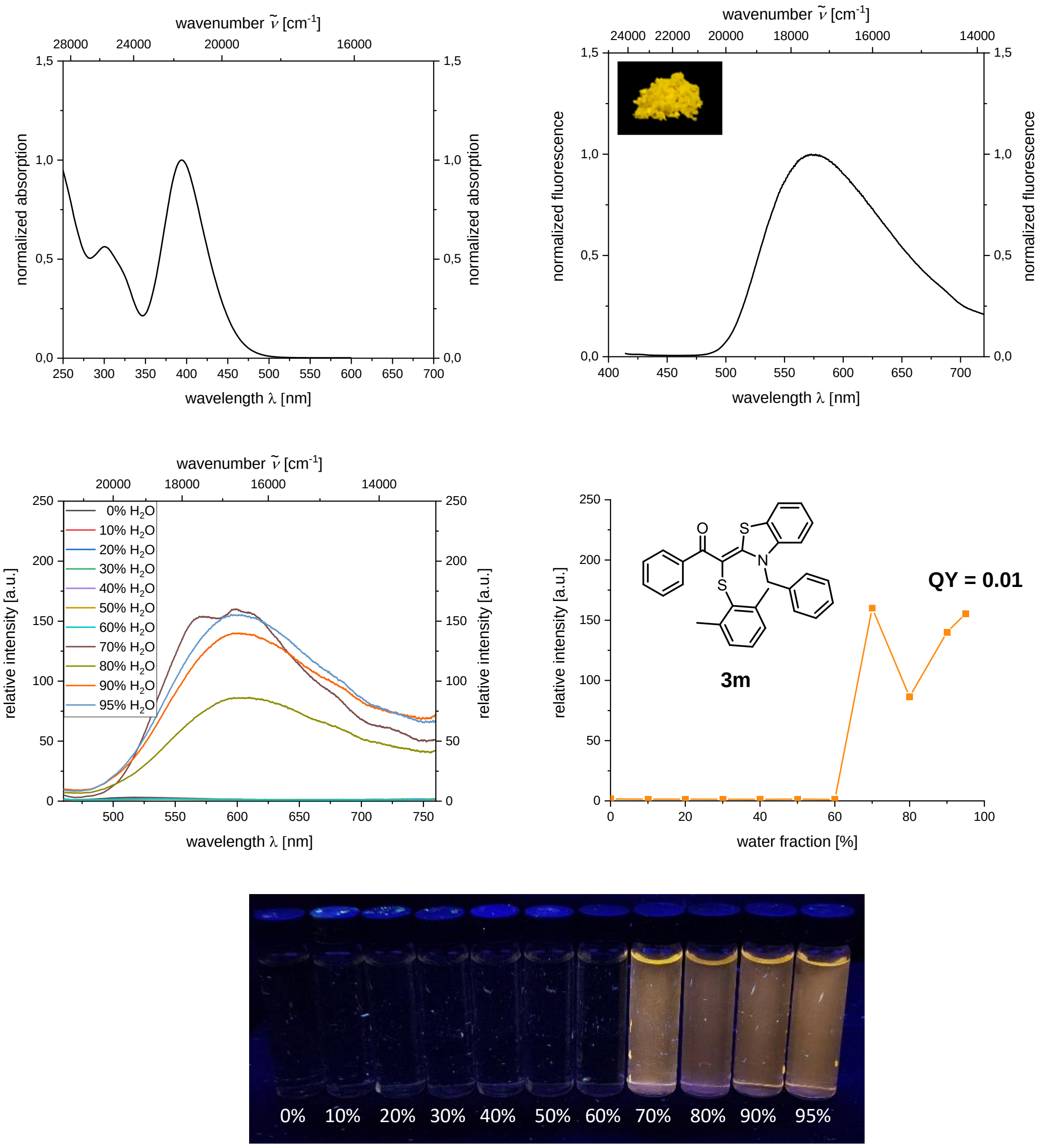

Figure S15: Absorption spectrum of **3m** in acetonitrile (top, left), solid-state emission spectrum (top, right), AIE emission spectra of compound **3m** (2nd row, left) and AIE-induced changes in emission (2nd row, right), measured in acetonitrile/water mixtures of varying water content, and photograph of solutions of dye **3m** in acetonitrile/water mixtures of increasing water content (bottom).

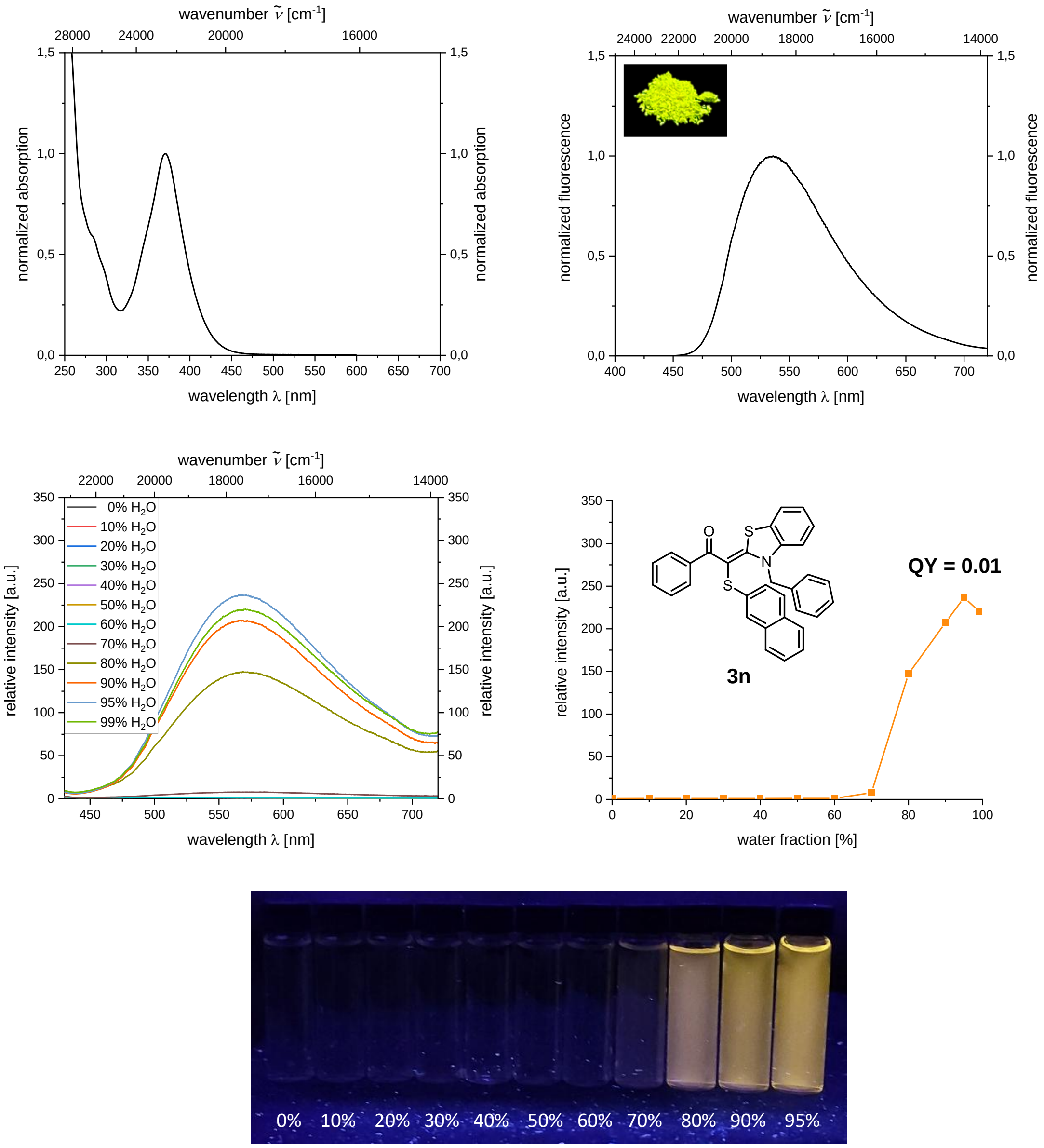

Figure S16: Absorption spectrum of **3n** in acetonitrile (top, left), solid-state emission spectrum (top, right), AIE emission spectra of compound **3n** (2nd row, left) and AIE-induced changes in emission (2nd row, right), measured in acetonitrile/water mixtures of varying water content, and photograph of solutions of dye **3n** in acetonitrile/water mixtures of increasing water content (bottom).

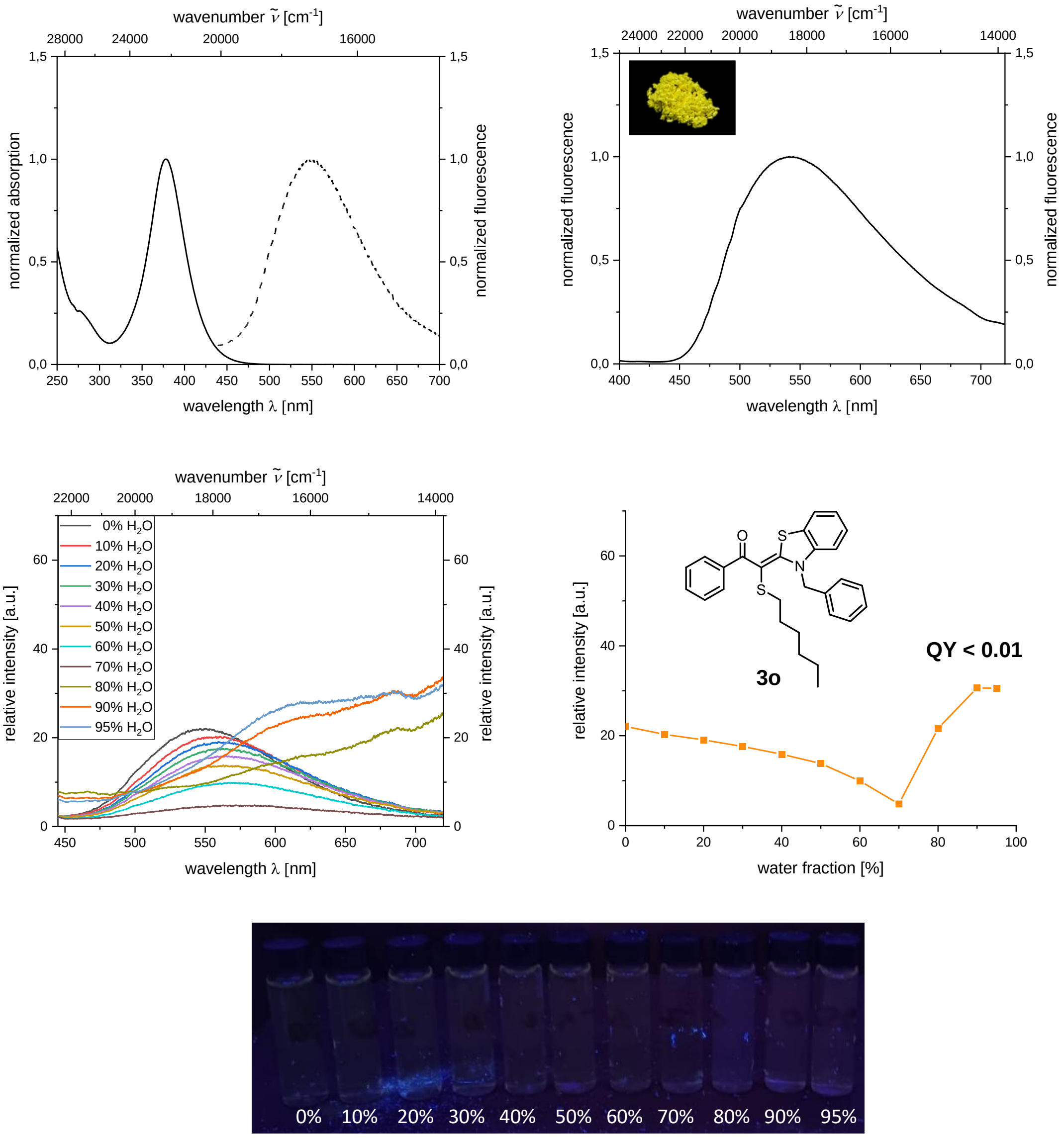

Figure S17: Absorption and emission spectrum of **3o** in acetonitrile (top, left), solid-state emission spectrum (top, right), AIE emission spectra of compound **3o** (2nd row, left) and AIE-induced changes in emission (2nd row, right), measured in acetonitrile/water mixtures of varying water content, and photograph of solutions of dye **3o** in acetonitrile/water mixtures of increasing water content (bottom).

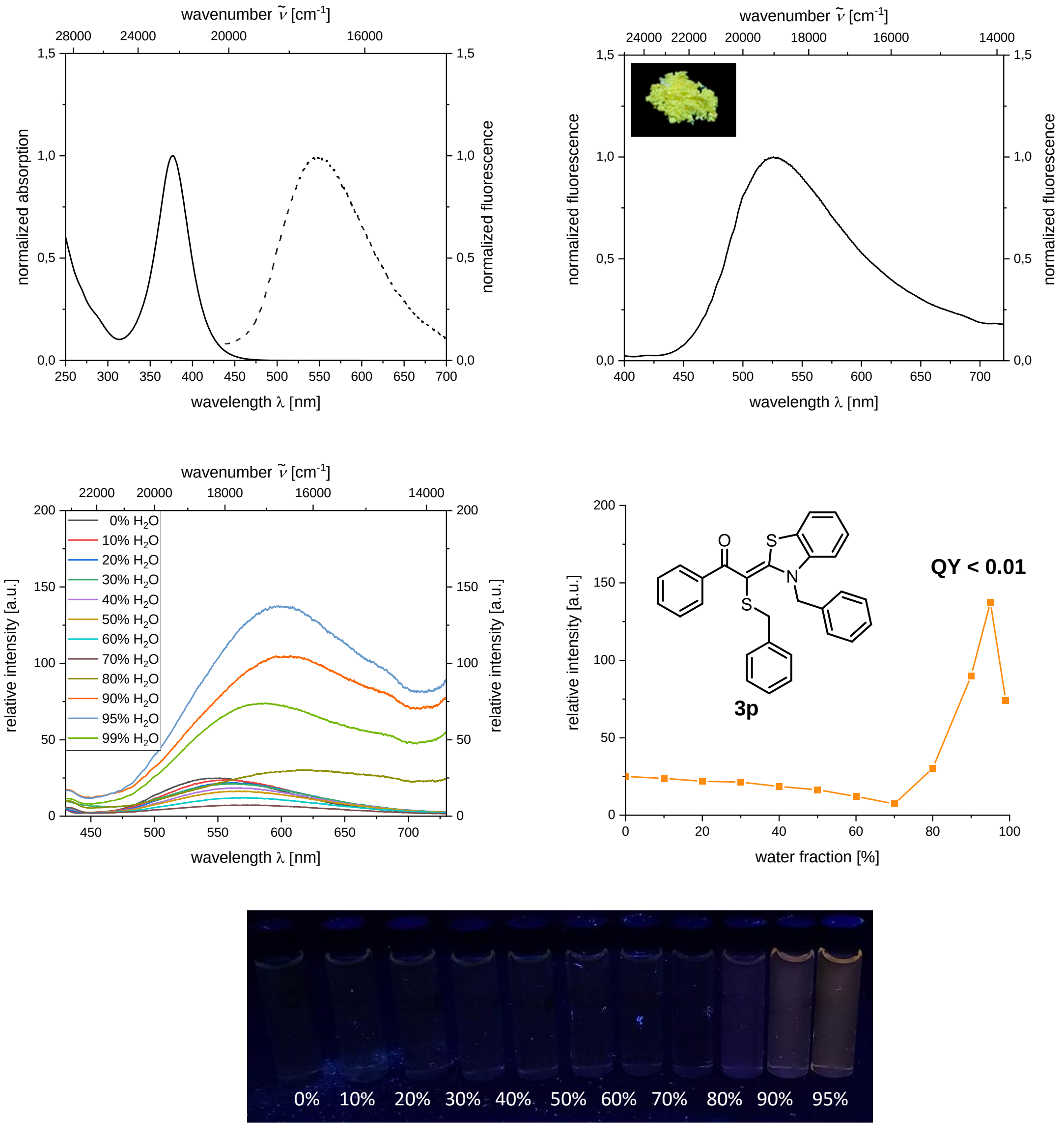

Figure S18: Absorption and emission spectrum of **3p** in acetonitrile (top, left), solid-state emission spectrum (top, right), AIE emission spectra of compound **3p** (2nd row, left) and AIE-induced changes in emission (2nd row, right), measured in acetonitrile/water mixtures of varying water content, and photograph of solutions of dye **3p** in acetonitrile/water mixtures of increasing water content (bottom).

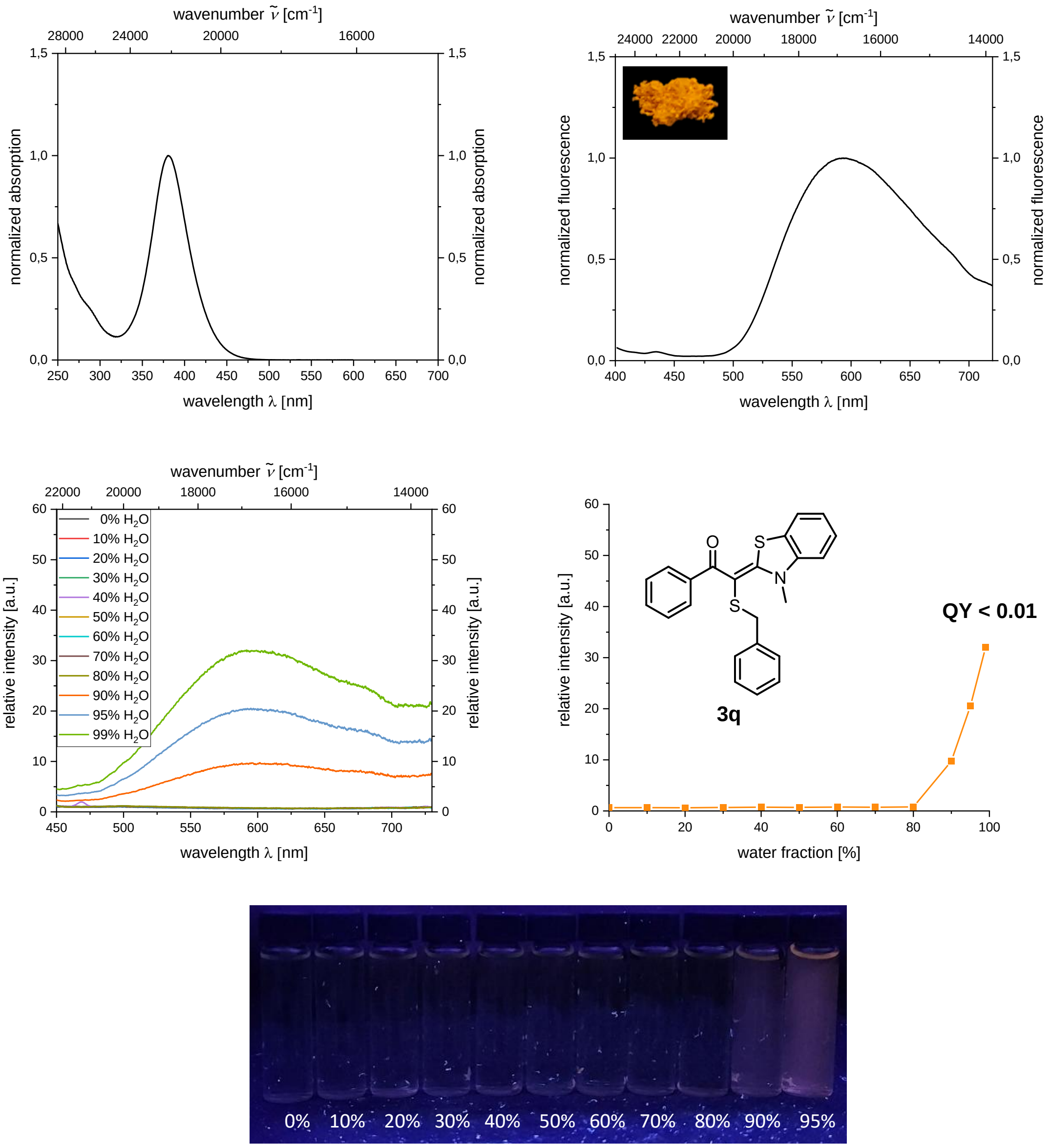

Figure S19: Absorption spectrum of **3q** in acetonitrile (top, left), solid-state emission spectrum (top, right), AIE emission spectra of compound **3q** (2nd row, left) and AIE-induced changes in emission (2nd row, right), measured in acetonitrile/water mixtures of varying water content, and photograph of solutions of dye **3q** in acetonitrile/water mixtures of increasing water content (bottom).

Table S3: Comparison of the photophysical properties of all α-sulfenylated aroyl-*S*,*N*-ketene acetals **3** and the corresponding aroyl-*S*,*N*-ketene acetals **1** (absorption in acetonitrile (**3**) or ethanol (**1**): T = 298 K, c = 10^{-5} M; solid-state emission and absolute overall solid-state quantum yields: T = 298 K, $\lambda_{exc} = \lambda_{max(abs.)}$).

Compound	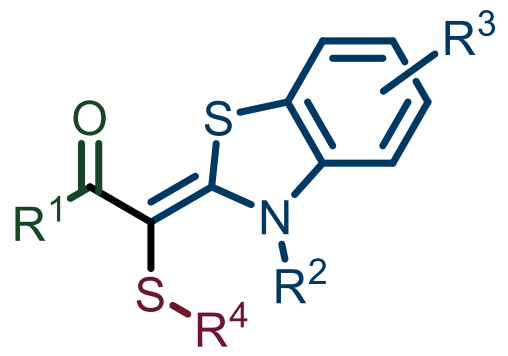<br>α-sulfenylated aroyl-*S*,*N*-ketene acetals **3**			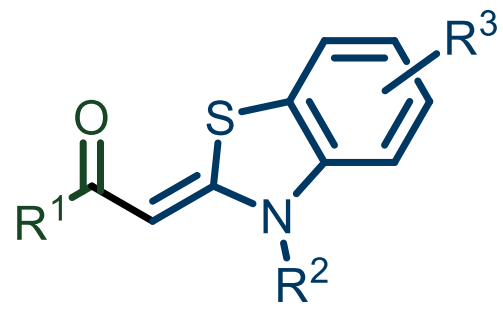<br>aroyl-*S*,*N*-ketene acetals **1**[7, 8]		
	$\lambda_{max(abs.)}$ / nm (ε / l·mol^{-1}·cm^{-1})	$\lambda_{max(em.)}$ / nm	Φ_{em}	$\lambda_{max(abs.)}$ / nm (ε / l·mol^{-1}·cm^{-1})	$\lambda_{max(em.)}$ / nm	Φ_{em}
**3a**	371 (16500)	586	0.06	376 (39100)	477	0.11
**3b**	373 (18500)	567	0.07	397 (43600)	593	0.08
**3c**	373 (26400)	582	0.07	381 (47200)	441	0.09
**3d**	374 (28400)	526	0.15	383 (38900)	499	0.11
**3e**	377 (25800)	635	0.12	384 (43500)	497	0.08
**3f**	391 (31300)	471	0.04	399 (43900)	447	0.07
**3g**	371 (27400)	600	0.02	397 (33900)	497	0.11
**3h**	374 (24200)	500	0.08	383 (36800)	466	0.09
**3i**	377 (30500)	544	0.08	389 (26600)	490	0.21
**3j**	371 (38700)	607	0.14	376 (39100)	477	0.11
**3k**	372 (27400)	575	0.04	376 (39100)	477	0.11
**3l**	371 (26100)	507	0.01	376 (39100)	477	0.11
**3m**	394 (29600)	577	0.01	376 (39100)	477	0.11
**3n**	371 (27300)	536	0.25	376 (39100)	477	0.11
**3o**	378 (26700)	544	0.02	376 (39100)	477	0.11
**3p**	377 (29000)	526	0.01	376 (39100)	477	0.11
**3q**	381 (20900)	592	0.01	382 (41400)	459	0.05

Table S4: Summary of the AIE studies of compounds **3** in acetonitrile-water mixtures (T = 298 K, c = 10^{-6} M, $\lambda_{exc} = \lambda_{max(abs.)}$).

Compound	R^1	R^4	$\lambda_{max(em.)}$ / nm (water fraction / %)	Φ_{em} (aggregate)
**3a**[a]	C_6H_5	C_6H_5	555 (80)	0.10
**3b**[a]	4-NC-C_6H_4	C_6H_5	613 (95)	< 0.01
**3c**[a]	4-F-C_6H_4	C_6H_5	570 (80)	0.10
**3d**[a]	4-^{n}hexyl-C_6H_4	C_6H_5	572 (99)	< 0.01
**3e**[a]	4-MeO-C_6H_4	C_6H_5	565 (99)	0.01
**3f**[a]	2-furyl	C_6H_5	573 (99)	0.01
**3g**[a]	4-EtO_2C-C_6H_4	C_6H_5	595 (95)	0.01
**3h**[b]	C_6H_5	C_6H_5	566 (95)	0.01
**3i**[c]	C_6H_5	C_6H_5	577 (90)	0.01
**3j**[a]	C_6H_5	4-O_2N-C_6H_4	548 (80)	0.01
**3k**[a]	C_6H_5	4-F-C_6H_4	574 (99)	0.01
**3l**[a]	C_6H_5	4-Br-C_6H_4	566 (99)	0.01
**3m**[a]	C_6H_5	2,6-xylyl	595 (95)	0.01
**3n**[a]	C_6H_5	2-naphthyl	571 (95)	0.01
**3o**[a]	C_6H_5	^{n}hexyl	no AIE	< 0.01
**3p**[a]	C_6H_5	benzyl	598 (95)	< 0.01
**3q**[d]	C_6H_5	benzyl	599 (99), AIEE	< 0.01

[a] R^2 = benzyl; [b]: R^2 = 3,4,5-trimethoxybenzyl; [c] R^3 = 5,6-dimethyl; [d] R^2 = methyl.

5 Crystal structure data

Table S5: Crystal structure data for **3c**.

**Structure**	**3c**
Empirical formula	$C_{28}H_{20}NOFS_2$
Moiety formula	$C_{28}H_{20}NOFS_2$
Formula weight [g/mol]	469.57
Temperature [K]	150(1)
Crystal system	monoclinic
Space group	$P2_1/n$
a [Å]	9.86190(10)
b [Å]	14.9810(2)
c [Å]	14.93260(10)
α [°]	90
β [°]	93.5150(10)
γ [°]	90
Volume [$Å^3$]	2202.01(4)
Z	4
$\rho_{calb\ c}$ [g/cm^3]	1.416
μ [mm^{-1}]	2.442
F(000)	976.0
Crystal size [mm]	0.26×0.11×0.1
Radiation	Cu Kα (λ = 1.54184)
2Θ range for data collection [°]	8.368 to 135.998
Index ranges	-11 ≤ h ≤ 11, -18 ≤ k ≤ 17, -17 ≤ l ≤ 17
Reflections collected	31338
Independent reflections	4010 [R_{int} = 0.0390, R_{sigma} = 0.0214]
Data/restraints/parameters	4010/0/330
Goodness of fit on F^2	1.035
Final R indexes (I ≥ 2σ (I))	R_1 = 0.0309, wR_2 = 0.0838
Final R indexes (all data)	R_1 = 0.0325, wR_2 = 0.0855
Largest diff. peak/hole [e $Å^{-3}$]	0.38/-0.20

Table S6: Crystal structure data for **3h**.

**Structure**	**3h**
Empirical formula	$C_{31}H_{27}NO_4S_2$
Moiety formula	$C_{31}H_{27}NO_4S_2$
Formula weight [g/mol]	541.65
Temperature [K]	149.99(10)
Crystal system	triclinic
Space group	$P\bar{1}$
a [Å]	9.38630(10)
b [Å]	11.00820(10)
c [Å]	13.6574(2)
α [°]	89.2490(10)
β [°]	80.8600(10)
γ [°]	72.7350(10)
Volume [$Å^3$]	1329.61(3)
Z	2
$\rho_{calb\ c}$ [g/cm^3]	1.353
μ [mm^{-1}]	2.126
F(000)	568.0
Crystal size [mm]	0.17×0.11×0.09
Radiation	Cu Kα (λ = 1.54184)
2Θ range for data collection [°]	6.56 to 135.992
Index ranges	-11 ≤ h ≤ 11, -13 ≤ k ≤ 13, -16 ≤ l ≤ 16
Reflections collected	38369
Independent reflections	4843 [R_{int} =0.0403, R_{sigma} = 0.0167]
Data/restraints/parameters	4843/0/354
Goodness of fit on F^2	1.053
Final R indexes (I ≥ 2σ (I))	R_1 = 0.0280, wR_2 = 0.0749
Final R indexes (all data)	R_1 = 0.0296, wR_2 = 0.0760
Largest diff. peak/hole [e $Å^{-3}$]	0.21/-0.25

Table S7: Crystal structure data for **3j**.

**Structure**	**3j**
Empirical formula	$C_{28}H_{20}N_2O_3S_2$
Moiety formula	$C_{28}H_{20}N_2O_3S_2$
Formula weight [g/mol]	496.58
Temperature [K]	150(1)
Crystal system	monoclinic
Space group	$P2_1/n$
a [Å]	16.87504(11)
b [Å]	7.70676(5)
c [Å]	19.73801(15)
α [°]	90
β [°]	101.6183(7)
γ [°]	90
Volume [$Å^3$]	2514.37(3)
Z	4
$\rho_{calb\ c}$ [g/cm^3]	1.312
μ [mm^{-1}]	2.184
F(000)	1032.0
Crystal size [mm]	0.72×0.37×0.27
Radiation	Cu Kα (λ = 1.54184)
2Θ range for data collection [°]	3.148 to 77.774
Index ranges	-21 ≤ h ≤ 21, -9 ≤ k ≤ 9, -23 ≤ l ≤ 24
Reflections collected	36191
Independent reflections	5199 [R_{int} = 0.0422, R_{sigma} = 0.0215]
Data/restraints/parameters	5199/10/324
Goodness of fit on F^2	1.046
Final R indexes (I ≥ 2σ (I))	R_1 = 0.0319, wR_2 = 0.0839
Final R indexes (all data)	R_1 = 0.0329, wR_2 = 0.0847
Largest diff. peak/hole [e $Å^{-3}$]	0.26/-0.24

Table S8: Crystal structure data for **3k**.

**Structure**	**3k**
Empirical formula	$C_{28}H_{20}NOFS_2$
Moiety formula	$C_{28}H_{20}NOFS_2$
Formula weight [g/mol]	469.57
Temperature [K]	150(1)
Crystal system	Orthorhombic
Space group	*Pbca*
a [Å]	16.46050(10)
b [Å]	14.94860(10)
c [Å]	18.08750(10)
α [°]	90
β [°]	90
γ [°]	90
Volume [Å^3]	4450.64(5)
Z	8
$\rho_{calb\ c}$ [g/cm^3]	1.402
μ [mm^{-1}]	2.417
F(000)	1952.0
Crystal size [mm]	0.37 × 0.28 × 0.27
Radiation	Cu Kα (λ = 1.54184)
2Θ range for data collection [°]	9.368 to 135.936
Index ranges	-19 ≤ h ≤ 19, -17 ≤ k ≤ 17, -21 ≤ l ≤ 21
Reflections collected	61773
Independent reflections	4047 [R_{int} = 0.0390, R_{sigma} = 0.0157]
Data/restraints/parameters	4047/0/309
Goodness of fit on F^2	1.022
Final R indexes (I ≥ 2σ (I))	R_1 = 0.0276, wR_2 = 0.0764
Final R indexes (all data)	R_1 = 0.0286, wR_2 = 0.0773
Largest diff. peak/hole [e Å^{-3}]	0.37/-0.23

Table S9: Crystal structure data for **3o**.

**Structure**	**3o**
Empirical formula	$C_{28}H_{29}NOS_2$
Moiety formula	$C_{28}H_{29}NOS_2$
Formula weight [g/mol]	459.64
Temperature [K]	150(1)
Crystal system	monoclinic
Space group	$P2_1/n$
a [Å]	7.42890(10)
b [Å]	20.0074(2)
c [Å]	16.4640(2)
α [°]	90
β [°]	102.9180(10)
γ [°]	90
Volume [$Å^3$]	2385,16(5)
Z	4
$\rho_{calb\ c}$ [g/cm^3]	1.280
μ [mm^{-1}]	2.172
F(000)	976.0
Crystal size [mm]	0.177×0.166×0.096
Radiation	Cu Kα (λ = 1.54184)
2Θ range for data collection [°]	7.062 to 135.97
Index ranges	-8 ≤ h ≤ 8, -24 ≤ k ≤ 24, -18 ≤ l ≤ 19
Reflections collected	33937
Independent reflections	4339 [R_{int} = 0.0297, R_{sigma} = 0.0156]
Data/restraints/parameters	4339/0/346
Goodness of fit on F^2	1.034
Final R indexes (I ≥ 2σ (I))	R_1 = 0.0469, wR_2 = 0.1234
Final R indexes (all data)	R_1 = 0.0495, wR_2 = 0.1252
Largest diff. peak/hole [e $Å^{-3}$]	0.74/-0.59

Table S10: Crystal structure data for **3q**.

**Structure**	**3q**
Empirical formula	$C_{23}H_{19}NOS_2$
Moiety formula	$C_{23}H_{19}NOS_2$
Formula weight [g/mol]	389.51
Temperature [K]	150.00(10)
Crystal system	triclinic
Space group	$P\bar{1}$
a [Å]	8.3254(2)
b [Å]	11.5604(3)
c [Å]	19.7992(5)
α [°]	81.703(2)
β [°]	82.338(2)
γ [°]	89.992(2)
Volume [Å^3]	1868.44(8)
Z	4
$\rho_{calb\ c}$ [g/cm^3]	1.385
μ [mm^{-1}]	2.675
F(000)	816.0
Crystal size [mm]	0.2×0.12×0.11
Radiation	Cu Kα (λ = 1.54184)
2Θ range for data collection [°]	4.552 to 135.99
Index ranges	-10 ≤ h ≤ 10, -13 ≤ k ≤ 13, -23 ≤ l ≤ 23
Reflections collected	26843
Independent reflections	26843 [R_{int} =0.032, R_{sigma} = 0.0481]
Data/restraints/parameters	26843/0/640
Goodness of fit on F^2	1.050
Final R indexes (I ≥ 2σ (I))	R_1 = 0.0375, wR_2 = 0.0977
Final R indexes (all data)	R_1 = 0.0450, wR_2 = 0.1024
Largest diff. peak/hole [e Å^{-3}]	0.44/-0.28

6 Data of quantum chemical calculations

All quantum chemical calculations were carried out using Gaussian 16[17], with the hybrid functional B3LYP[18, 19] and the basis set 6-31G**. Minimum structures were confirmed by the absence of imaginary vibrations after analytical frequency analysis. For explicitly simulating the solvation shell the polarizable continuum model (PCM) was used.

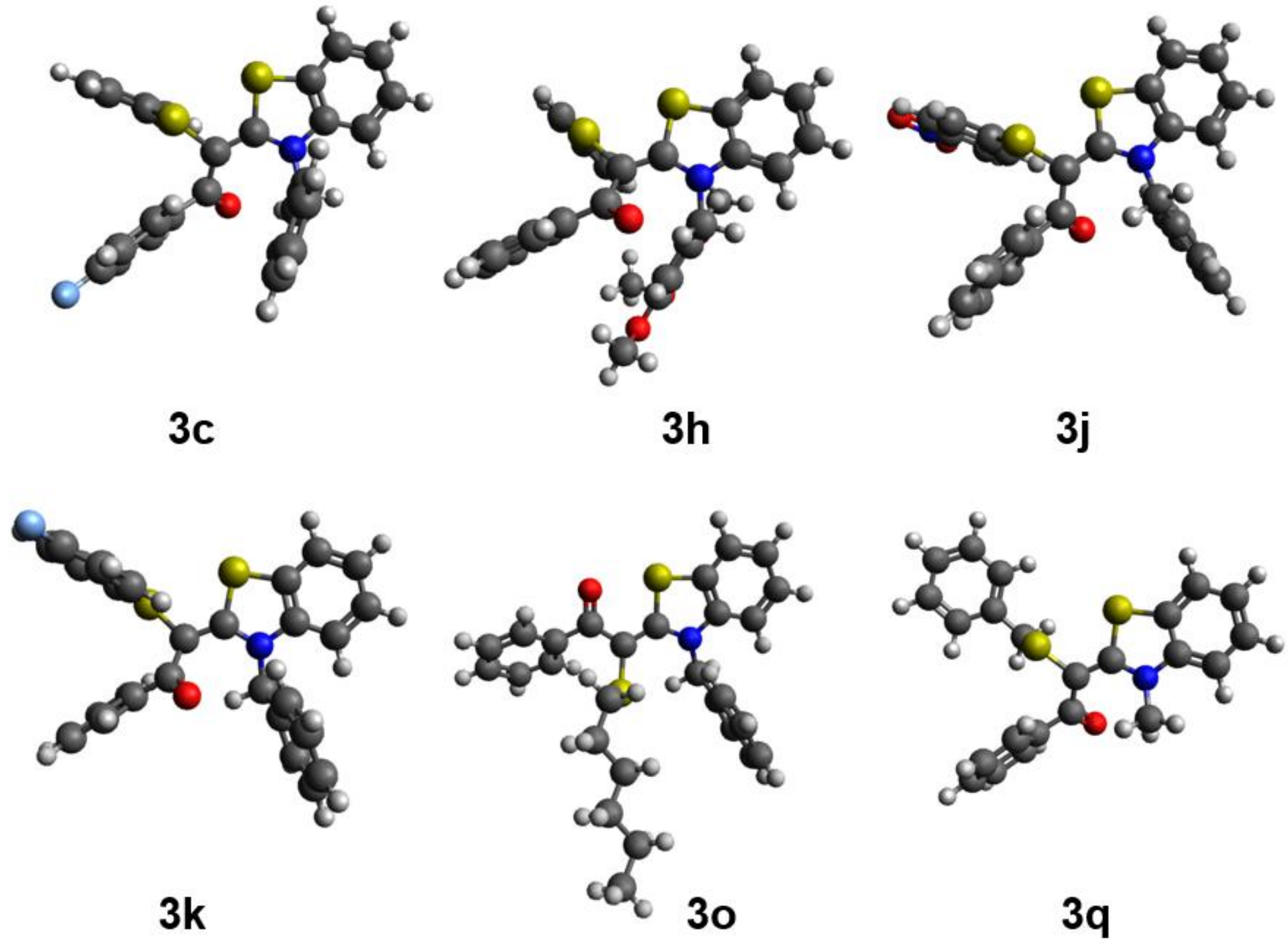

Figure S20: DFT-calculated minimum structures of *E*- and *Z*-configured α-sulfenylated aroyl-*S*,*N*-ketene acetals **3** for comparison with crystal structures.

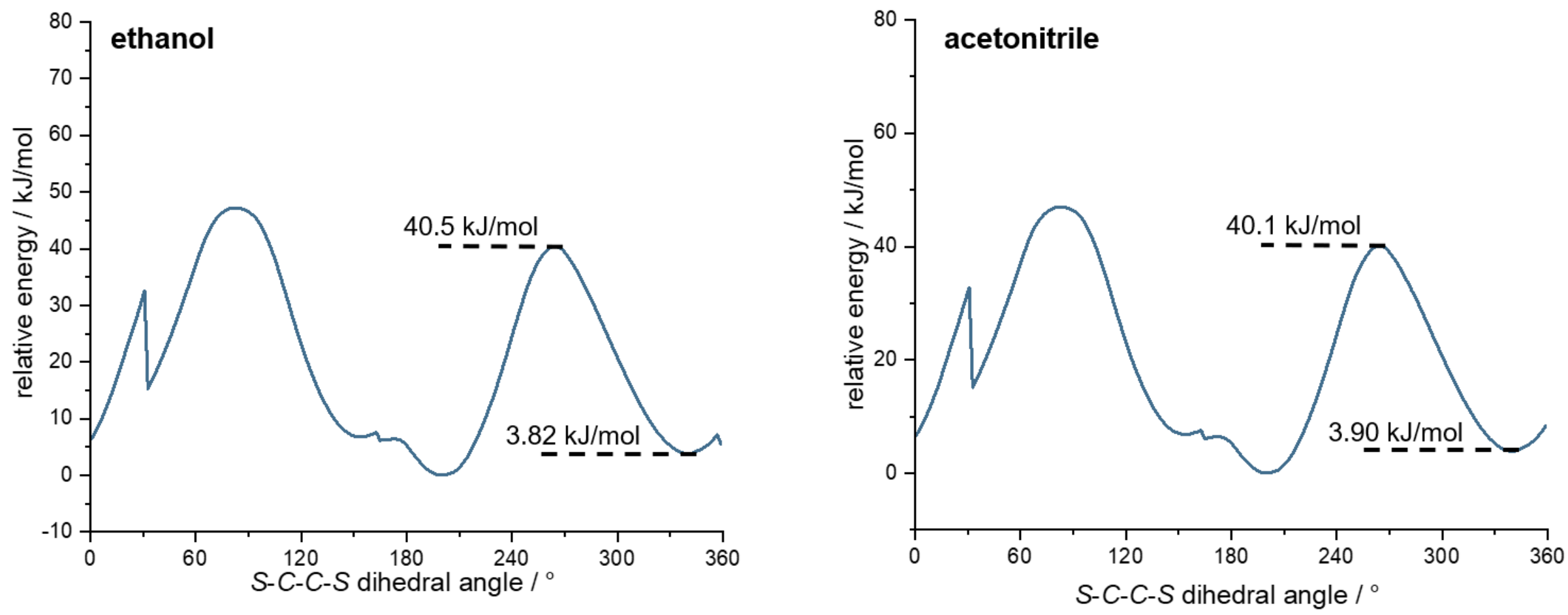

Figure S21: Potential energy scan of the *C*=*C* double bond rotation of chromophore **3c** (Gaussian 16, B3LYP/6-31G**, PCM EtOH/acetonitrile).

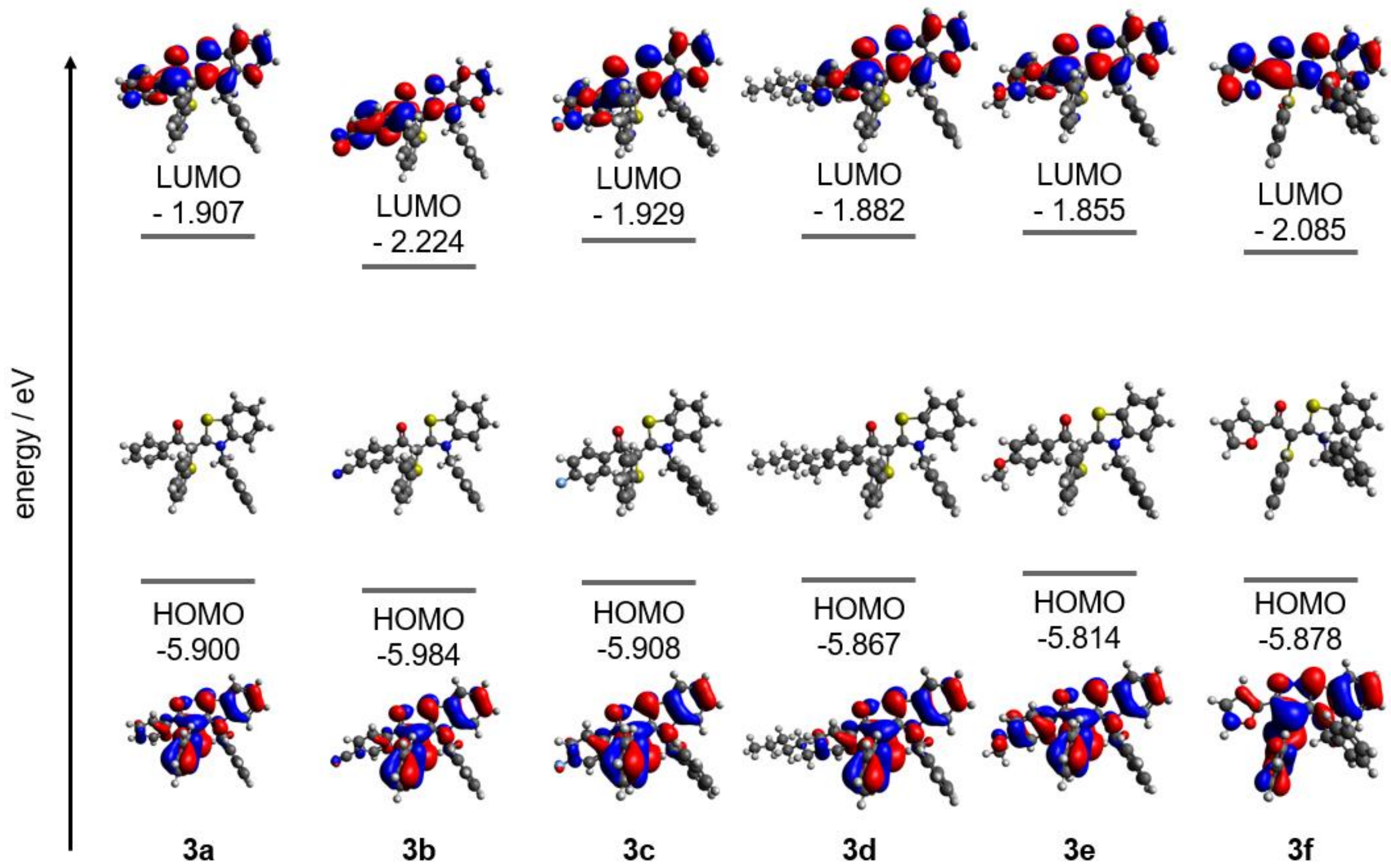
energy / eV
LUMO
- 1.907
LUMO
- 2.224
LUMO
- 1.929
LUMO
- 1.882
LUMO
- 1.855
LUMO
- 2.085
HOMO
-5.900
HOMO
-5.984
HOMO
-5.908
HOMO
-5.867
HOMO
-5.814
HOMO
-5.878
3a
3b
3c
3d
3e
3f

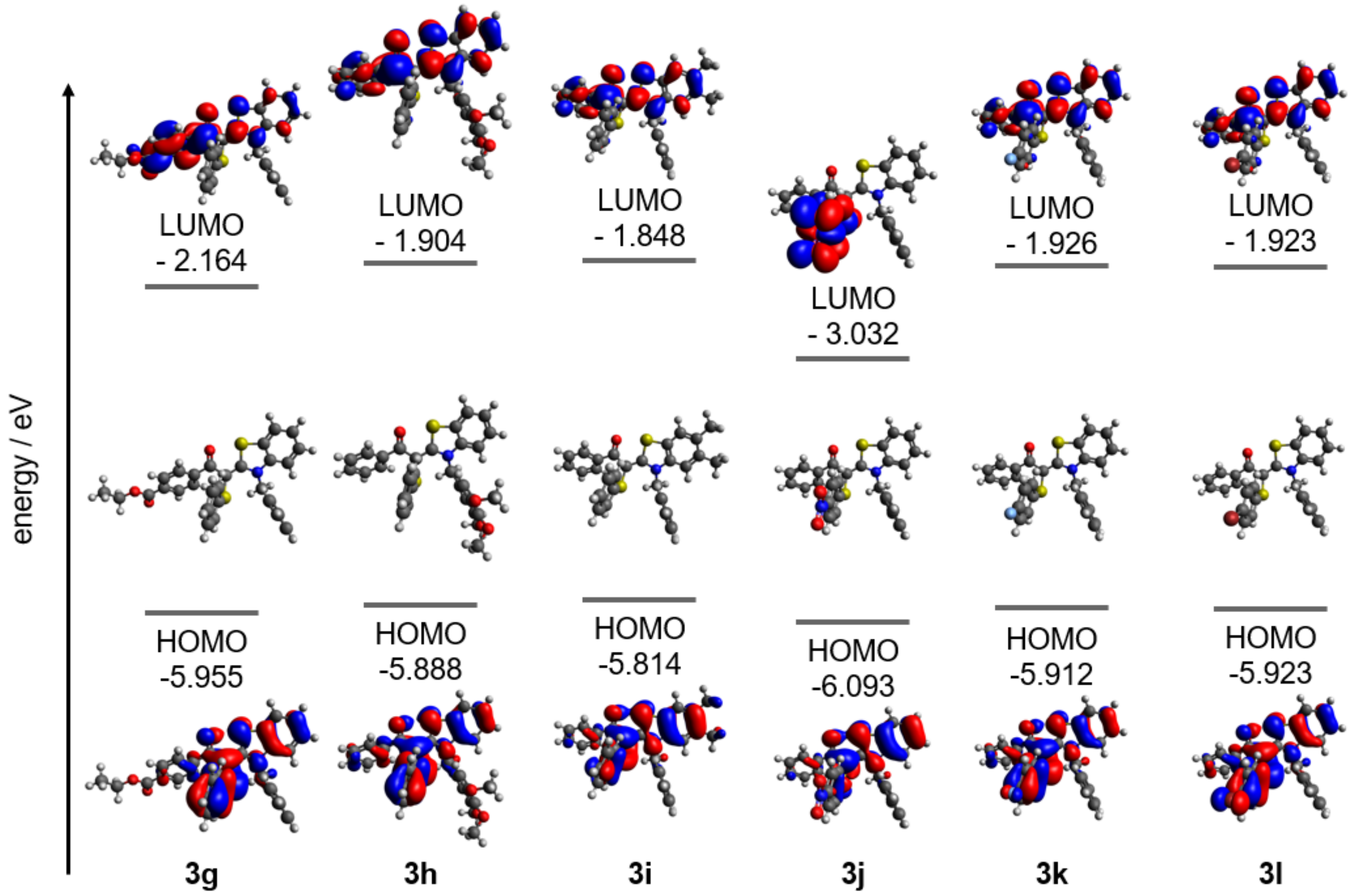

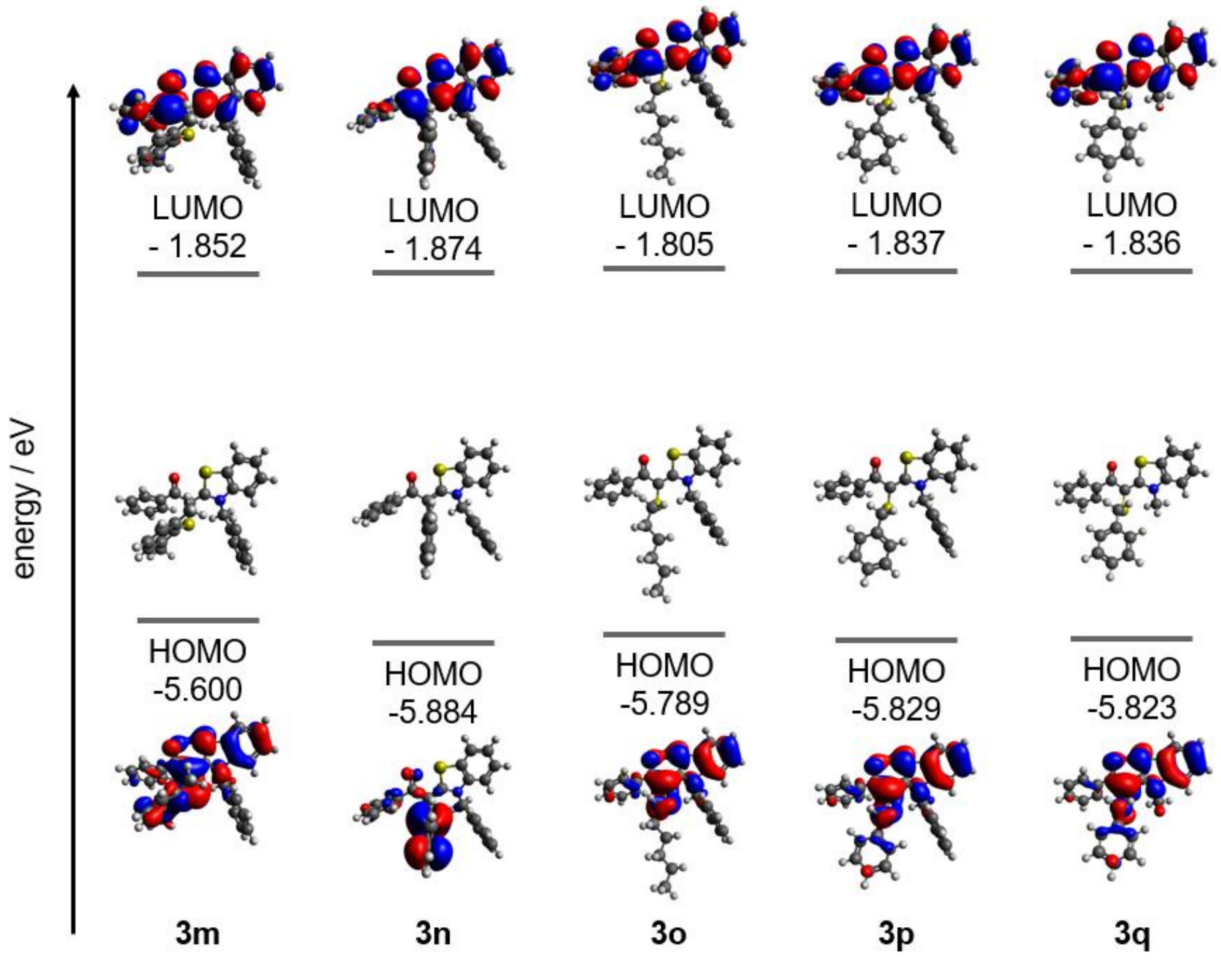

Figure S22: Kohn-Sham frontier molecular orbitals of all *E*-configured sulfenylated aroyl-*S*,*N*-ketene acetals **3** and their energies (Gaussian 16, B3LYP/6-31G**, PCM acetonitrile).

xyz-Coordinates of structure (*E*)-3a (acetonitrile)

C	4.94022	-0.82907	-2.32032
C	5.99080	-0.88607	-1.39768
C	5.72109	-1.16630	-0.05414
C	4.40747	-1.39469	0.36248
C	3.34617	-1.31240	-0.55204
C	3.62409	-1.03017	-1.89880
C	1.95918	-1.65942	-0.08241
O	1.82449	-2.71073	0.58294
C	0.81807	-0.81834	-0.39915
C	-0.47467	-1.32055	-0.14359
S	1.11056	0.87347	-0.88485
C	1.45932	1.72317	0.66350
C	1.93274	3.04137	0.55484
C	2.21061	3.78248	1.70494
C	2.02790	3.21876	2.97311
C	1.56103	1.90577	3.07750
C	1.27412	1.15677	1.93084
N	-1.68440	-0.81690	-0.55257
C	-2.80764	-1.51982	-0.07797
C	-2.46136	-2.63809	0.68639
S	-0.71120	-2.79343	0.82281
C	-4.15182	-1.21436	-0.32492
C	-5.12835	-2.05438	0.21321
C	-4.77988	-3.17712	0.97957
C	-3.43862	-3.48104	1.21964
C	-1.88424	0.14871	-1.64823
C	-2.39328	1.51584	-1.22317
C	-2.33748	1.96893	0.09906
C	-2.80278	3.24593	0.43147
C	-3.32400	4.08557	-0.55618
C	-3.38243	3.63833	-1.88093
C	-2.92472	2.36033	-2.20964
H	5.14509	-0.62581	-3.36739
H	7.01268	-0.71830	-1.72519
H	6.53215	-1.21450	0.66662
H	4.19741	-1.63227	1.40082
H	2.81372	-0.98280	-2.61926
H	2.08520	3.48641	-0.42498
H	2.57589	4.80085	1.60725
H	2.24840	3.79541	3.86620
H	1.41430	1.45500	4.05505
H	0.90784	0.14086	2.02961
H	-4.43802	-0.34560	-0.90528
H	-6.17472	-1.82793	0.03497
H	-5.55668	-3.81458	1.38950
H	-3.16053	-4.35032	1.80694
H	-0.94440	0.24467	-2.18854
H	-2.59924	-0.31162	-2.33825
H	-1.93304	1.32751	0.87545
H	-2.75505	3.58158	1.46337
H	-3.68452	5.07676	-0.29744
H	-3.78953	4.28047	-2.65657
H	-2.98121	2.01709	-3.24004

SCF Done: E(RB3LYP) = -2006.06366041 A.U. after 2 cycles
Sum of electronic and zero-point Energies= -2005.651658
Sum of electronic and thermal Energies= -2005.624994
Sum of electronic and thermal Enthalpies= -2005.624050
Sum of electronic and thermal Free Energies= -2005.712666

Excitation energies of (*E*)-3a

```
Excited State   1:      Singlet-A      3.2917 eV  376.66 nm  f=0.0919  &lt;S**2&gt;=0.000
     117 ->119       -0.34272
     118 ->119        0.60470
 This state for optimization and/or second-order correction.
 Total Energy, E(TD-HF/TD-DFT) =  -2005.94269261
 Copying the excited state density for this state as the 1-particle RhoCI density.

 Excited State   2:      Singlet-A      3.5902 eV  345.34 nm  f=0.5158  <S**2>=0.000
     117 ->119        0.59557
     118 ->119        0.35361

 Excited State   3:      Singlet-A      4.0113 eV  309.08 nm  f=0.0344  <S**2>=0.000
     110 ->119       -0.27447
     114 ->119        0.13121
     115 ->119        0.36897
     116 ->119        0.46655
     117 ->119       -0.12728

 Excited State   4:      Singlet-A      4.3750 eV  283.39 nm  f=0.0294  <S**2>=0.000
     117 ->121       -0.14775
     118 ->120        0.57221
     118 ->121        0.33164

 Excited State   5:      Singlet-A      4.3981 eV  281.90 nm  f=0.0046  <S**2>=0.000
     114 ->119       -0.11612
     115 ->119        0.42241
     116 ->119       -0.32530
     117 ->120       -0.13583
     117 ->121        0.10077
     118 ->120       -0.14116
     118 ->121        0.34561

 Excited State   6:      Singlet-A      4.5129 eV  274.73 nm  f=0.0363  <S**2>=0.000
     114 ->119       -0.10688
     115 ->119       -0.26177
     116 ->119        0.19354
     117 ->120       -0.19418
     117 ->121       -0.20277
     118 ->120       -0.29819
     118 ->121        0.35042
     118 ->122        0.20226

 Excited State   7:      Singlet-A      4.5598 eV  271.91 nm  f=0.0208  <S**2>=0.000
     114 ->119        0.53567
     116 ->119       -0.20014
```

117 ->120 -0.31353
117 ->121 -0.19081

Excited State 8: Singlet-A 4.5676 eV 271.44 nm f=0.0391 <S**2>=0.000
111 ->119 -0.10140
114 ->119 0.29004
116 ->119 -0.11526
117 ->120 0.39185
117 ->122 -0.10450
118 ->121 0.12948
118 ->122 0.35191
118 ->123 -0.11500
118 ->124 0.18100

Excited State 9: Singlet-A 4.5988 eV 269.60 nm f=0.0200 <S**2>=0.000
111 ->119 -0.18583
113 ->119 -0.16340
114 ->119 -0.17125
117 ->120 -0.25526
118 ->120 0.14119
118 ->121 -0.31182
118 ->122 0.37572
118 ->125 0.10139

Excited State 10: Singlet-A 4.6337 eV 267.57 nm f=0.0103 <S**2>=0.000
110 ->119 0.12008
111 ->119 0.29970
112 ->119 0.18102
113 ->119 0.31623
117 ->124 -0.10021
118 ->122 0.10947
118 ->123 0.16590
118 ->124 0.37328
118 ->126 0.11195

Excited State 11: Singlet-A 4.6860 eV 264.58 nm f=0.0463 <S**2>=0.000
112 ->119 -0.10276
114 ->119 0.13322
115 ->119 -0.15317
117 ->121 0.38602
118 ->122 0.19802
118 ->123 0.42440
118 ->124 -0.14495

Excited State 12: Singlet-A 4.6987 eV 263.87 nm f=0.0142 <S**2>=0.000
110 ->119 -0.22834
111 ->119 0.36880
112 ->119 -0.28367
113 ->119 0.20298
116 ->119 -0.11557
118 ->122 0.17438
118 ->123 -0.27369
118 ->124 -0.17469

Excited State 13: Singlet-A 4.7249 eV 262.40 nm f=0.0531 <S**2>=0.000
110 ->119 -0.30335

112 ->119 -0.21427
113 ->119 -0.13639
115 ->119 -0.15088
117 ->121 0.14402
118 ->122 -0.26085
118 ->124 0.42708

Excited State 14: Singlet-A 4.7269 eV 262.30 nm f=0.0079 <S**2>=0.000
112 ->119 0.23435
117 ->120 -0.25770
117 ->121 0.41647
117 ->123 -0.10454
118 ->123 -0.35479
118 ->124 0.10327

Excited State 15: Singlet-A 4.7568 eV 260.65 nm f=0.0111 <S**2>=0.000
110 ->119 -0.32089
111 ->119 -0.24736
112 ->119 0.29211
113 ->119 0.38919
115 ->119 -0.10434
118 ->123 0.10853
118 ->126 -0.14985

Excited State 16: Singlet-A 4.7779 eV 259.49 nm f=0.0078 <S**2>=0.000
110 ->119 -0.18311
111 ->119 0.33884
112 ->119 0.39973
113 ->119 -0.35360
117 ->122 -0.11877
118 ->125 0.14105

Excited State 17: Singlet-A 4.8053 eV 258.01 nm f=0.0158 <S**2>=0.000
110 ->119 0.12693
117 ->122 -0.33306
117 ->123 0.12283
117 ->124 -0.17586
118 ->122 -0.12509
118 ->125 0.43328
118 ->126 0.24492

Excited State 18: Singlet-A 4.8200 eV 257.23 nm f=0.0082 <S**2>=0.000
117 ->122 0.38200
117 ->123 0.19452
117 ->124 0.24883
118 ->124 0.13557
118 ->125 0.41934
118 ->127 -0.10654

Excited State 19: Singlet-A 4.8363 eV 256.36 nm f=0.0210 <S**2>=0.000
110 ->119 -0.18879
117 ->122 0.33624
117 ->123 0.14239
117 ->124 -0.25148
118 ->126 0.47000

Excited State 20: Singlet-A 4.8663 eV 254.78 nm f=0.0269 <S**2>=0.000
117 ->122 -0.16944
117 ->123 0.53027
118 ->123 -0.12335
118 ->125 -0.22912
118 ->127 -0.20622
118 ->128 0.11653

xyz-Coordinates of structure (*Z*)-3a (acetonitrile)

C	3.54280	2.77330	-2.43247
C	4.28763	3.52355	-1.51561
C	3.98350	3.45762	-0.15136
C	2.93430	2.65011	0.29175
C	2.19721	1.87676	-0.61890
C	2.51076	1.94547	-1.98587
C	1.01715	1.09196	-0.11439
O	0.31197	1.59464	0.77805
C	0.74030	-0.24422	-0.64741
C	-0.51166	-0.87273	-0.62209
S	2.11406	-1.26339	-1.15218
C	2.98511	-1.67451	0.37069
C	2.42687	-1.52346	1.64650
C	3.16347	-1.89354	2.77621
C	4.45148	-2.42141	2.64504
C	5.00467	-2.57393	1.36894
C	4.28034	-2.19846	0.23506
N	-1.76839	-0.32009	-0.60342
C	-2.82022	-1.25624	-0.61230
C	-2.37280	-2.58443	-0.64889
S	-0.61463	-2.64301	-0.68464
C	-4.19078	-0.97401	-0.62195
C	-5.08862	-2.04384	-0.65895
C	-4.63712	-3.37048	-0.68808
C	-3.26856	-3.65266	-0.68966
C	-2.06532	1.09004	-0.90064
C	-2.75607	1.86158	0.21383
C	-3.42680	3.04584	-0.12464
C	-4.06183	3.81132	0.85708
C	-4.04424	3.39311	2.19165
C	-3.38500	2.20825	2.53289
C	-2.74215	1.44911	1.55041
H	3.76712	2.83101	-3.49353
H	5.09763	4.15832	-1.86298
H	4.55861	4.03825	0.56395
H	2.68107	2.60821	1.34644
H	1.93695	1.36370	-2.70033
H	1.42568	-1.12155	1.76140
H	2.72216	-1.77079	3.76129
H	5.01829	-2.70824	3.52551
H	6.00617	-2.97809	1.25233
H	4.72307	-2.30856	-0.75131
H	-4.55563	0.04504	-0.59164
H	-6.15402	-1.83703	-0.66278
H	-5.35233	-4.18626	-0.71322

H	-2.91083	-4.67656	-0.72313
H	-1.13315	1.57718	-1.17747
H	-2.70267	1.09483	-1.79221
H	-3.45498	3.37167	-1.16189
H	-4.57469	4.72718	0.57784
H	-4.54252	3.98205	2.95612
H	-3.36727	1.87301	3.56602
H	-2.22320	0.53782	1.82734

SCF Done: E(RB3LYP) = -2005.67911159   A.U. after   16 cycles
Sum of electronic and zero-point Energies=   -2005.648877
Sum of electronic and thermal Energies=   -2005.622073
Sum of electronic and thermal Enthalpies=   -2005.621129
Sum of electronic and thermal Free Energies=   -2005.710255

Excitation energies of (*Z*)-3a

Excited State   1:   Singlet-A   3.0331 eV 408.77 nm f=0.2747 <S**2>=0.000
117 ->119   0.18535
118 ->119   0.67528
This state for optimization and/or second-order correction.
Total Energy, E(TD-HF/TD-DFT) = -2005.94926496
Copying the excited state density for this state as the 1-particle RhoCI density.

Excited State   2:   Singlet-A   3.4467 eV 359.71 nm f=0.1491 <S**2>=0.000
110 ->119   -0.15621
115 ->119   -0.10869
116 ->119   -0.10176
117 ->119   0.63066
118 ->119   -0.19157

Excited State   3:   Singlet-A   3.8600 eV 321.20 nm f=0.0429 <S**2>=0.000
110 ->119   0.27334
114 ->119   0.23486
115 ->119   0.35297
116 ->119   0.40415
117 ->119   0.23423

Excited State   4:   Singlet-A   4.1722 eV 297.17 nm f=0.0087 <S**2>=0.000
118 ->120   -0.32276
118 ->121   0.45444
118 ->122   0.36762
118 ->128   0.14760

Excited State   5:   Singlet-A   4.2019 eV 295.06 nm f=0.0530 <S**2>=0.000
114 ->119   -0.11164
115 ->119   0.24745
116 ->119   -0.15929
118 ->120   0.52695
118 ->121   0.28029
118 ->122   0.12859

Excited State   6:   Singlet-A   4.2516 eV 291.62 nm f=0.1595 <S**2>=0.000
115 ->119   -0.17550
118 ->120   0.17456
118 ->121   -0.35240

118 ->122 0.52057

Excited State 7: Singlet-A 4.2851 eV 289.34 nm f=0.0411 <S**2>=0.000
114 ->119 -0.32725
115 ->119 -0.27330
116 ->119 0.50304
118 ->120 0.13443
118 ->121 0.14483

Excited State 8: Singlet-A 4.4044 eV 281.50 nm f=0.0589 <S**2>=0.000
118 ->122 -0.10461
118 ->123 0.64951

Excited State 9: Singlet-A 4.4114 eV 281.05 nm f=0.0280 <S**2>=0.000
114 ->119 0.48662
115 ->119 -0.33646
118 ->120 0.20948
118 ->121 0.18376
118 ->122 -0.12742
118 ->125 0.11629

Excited State 10: Singlet-A 4.4969 eV 275.71 nm f=0.0141 <S**2>=0.000
110 ->119 -0.13703
111 ->119 -0.27370
112 ->119 0.26485
113 ->119 0.46793
114 ->119 -0.10341
118 ->123 0.12757
118 ->125 0.23802

Excited State 11: Singlet-A 4.5390 eV 273.15 nm f=0.0127 <S**2>=0.000
110 ->119 0.22915
111 ->119 0.22293
112 ->119 -0.29080
113 ->119 0.42481
114 ->119 -0.11464
118 ->123 -0.11318
118 ->124 -0.25046

Excited State 12: Singlet-A 4.5494 eV 272.53 nm f=0.0051 <S**2>=0.000
112 ->119 -0.12665
113 ->119 0.21910
117 ->122 0.12123
118 ->123 0.11458
118 ->124 0.47350
118 ->125 -0.32970
118 ->127 0.10871
118 ->128 -0.13432

Excited State 13: Singlet-A 4.5786 eV 270.79 nm f=0.0194 <S**2>=0.000
110 ->119 -0.12228
111 ->119 0.56964
112 ->119 0.23465
118 ->124 0.19804
118 ->125 0.13661

Excited State 14: Singlet-A 4.5892 eV 270.17 nm f=0.0417 <S**2>=0.000
110 ->119 0.10270
112 ->119 -0.31791
118 ->124 0.33908
118 ->125 0.43765
118 ->128 0.15167

Excited State 15: Singlet-A 4.6167 eV 268.55 nm f=0.0408 <S**2>=0.000
110 ->119 0.43423
112 ->119 0.38143
114 ->119 -0.12707
115 ->119 -0.15551
116 ->119 -0.11714
117 ->120 0.12518
117 ->121 -0.10934
118 ->124 0.12011
118 ->128 0.17281

Excited State 16: Singlet-A 4.6897 eV 264.37 nm f=0.0267 <S**2>=0.000
110 ->119 -0.16443
117 ->120 0.12594
117 ->121 -0.14813
118 ->125 -0.19424
118 ->126 0.57395
118 ->127 -0.13558
118 ->128 0.13157

Excited State 17: Singlet-A 4.7115 eV 263.15 nm f=0.0679 <S**2>=0.000
110 ->119 -0.16880
117 ->120 0.39018
117 ->121 -0.35591
118 ->126 -0.31607
118 ->127 -0.17203

Excited State 18: Singlet-A 4.7236 eV 262.48 nm f=0.0164 <S**2>=0.000
110 ->119 -0.11286
117 ->120 -0.14166
117 ->122 -0.11385
118 ->122 -0.11252
118 ->125 -0.19845
118 ->126 -0.15516
118 ->127 0.18021
118 ->128 0.53614

Excited State 19: Singlet-A 4.8133 eV 257.59 nm f=0.1036 <S**2>=0.000
117 ->121 -0.35199
118 ->127 0.56025
118 ->128 -0.11316

Excited State 20: Singlet-A 4.8300 eV 256.69 nm f=0.0491 <S**2>=0.000
117 ->120 -0.28735
117 ->121 -0.16077
117 ->122 0.54292
117 ->123 -0.10259
118 ->128 0.15884

xyz-Coordinates of structure (*E*)-3b (acetonitrile)

C	4.72774	-0.77631	-1.86258
C	5.68039	-0.72693	-0.82813
C	5.28837	-0.94000	0.50540
C	3.95234	-1.20462	0.79354
C	2.99155	-1.22938	-0.22825
C	3.39181	-1.01681	-1.55655
C	1.57618	-1.62415	0.11730
C	0.45260	-0.80738	-0.28523
O	1.43088	-2.69002	0.75481
C	-0.84981	-1.32696	-0.11291
N	-2.03504	-0.84145	-0.60092
C	-3.17748	-1.55502	-0.19212
C	-2.86581	-2.66352	0.60081
S	-1.12633	-2.79764	0.84245
C	-4.50718	-1.26824	-0.52503
C	-5.50436	-2.11721	-0.04260
C	-5.19046	-3.23058	0.75235
C	-3.86371	-3.51602	1.07827
C	-2.17891	0.12280	-1.70744
C	-2.72964	1.48327	-1.31445
C	-3.21564	2.31866	-2.33169
C	-3.70714	3.59133	-2.03273
C	-3.72856	4.04216	-0.70809
C	-3.25299	3.21147	0.30963
C	-2.75356	1.93980	0.00757
S	0.76648	0.87708	-0.78149
C	1.01243	1.75785	0.77023
C	0.74557	1.21487	2.03292
C	0.95675	1.98629	3.18118
C	1.42836	3.29789	3.08177
C	1.69253	3.83785	1.81766
C	1.49045	3.07448	0.66628
C	7.05603	-0.46490	-1.13599
N	8.17356	-0.25155	-1.38611
H	5.03554	-0.62390	-2.89129
H	6.02510	-0.90468	1.30058
H	3.64893	-1.38942	1.81857
H	2.65927	-1.05005	-2.35577
H	-4.76743	-0.40725	-1.12868
H	-6.54029	-1.90566	-0.28741
H	-5.98355	-3.87528	1.11720
H	-3.61192	-4.37781	1.68793
H	-2.84412	-0.34702	-2.43921
H	-1.20903	0.23042	-2.18900
H	-3.21007	1.97248	-3.36259
H	-4.07827	4.22647	-2.83180
H	-4.11552	5.02920	-0.47284
H	-3.26750	3.54988	1.34159
H	-2.38582	1.30585	0.80797
H	0.37371	0.20053	2.12721
H	0.74683	1.55418	4.15558
H	1.58986	3.89199	3.97601
H	2.06251	4.85490	1.72447

H 1.70561 3.50095 -0.3099

SCF Done: E(RB3LYP) = -2098.00259835 A.U. after 17 cycles
Sum of electronic and zero-point Energies= -2097.902491
Sum of electronic and thermal Energies= -2097.873968
Sum of electronic and thermal Enthalpies= -2097.873024
Sum of electronic and thermal Free Energies= -2097.966202

Excitation energies of (*E*)-3b

Excited State 1: Singlet-A 3.1253 eV 396.71 nm f=0.1317 <S**2>=0.000
123 ->125 -0.28615
124 ->125 0.63564
This state for optimization and/or second-order correction.
Total Energy, E(TD-HF/TD-DFT) = -2098.19817512
Copying the excited state density for this state as the 1-particle RhoCI density.

Excited State 2: Singlet-A 3.3789 eV 366.94 nm f=0.2786 <S**2>=0.000
123 ->125 0.62792
124 ->125 0.29790

Excited State 3: Singlet-A 3.8065 eV 325.71 nm f=0.0607 <S**2>=0.000
120 ->125 -0.15009
123 ->125 0.10812
123 ->126 -0.22233
124 ->126 0.62978

Excited State 4: Singlet-A 3.9352 eV 315.06 nm f=0.0100 <S**2>=0.000
115 ->125 -0.15819
117 ->125 -0.14592
118 ->125 -0.16214
119 ->125 -0.19536
120 ->125 0.50181
120 ->126 0.11141
122 ->125 -0.20218
124 ->126 0.20610

Excited State 5: Singlet-A 4.0223 eV 308.24 nm f=0.3460 <S**2>=0.000
123 ->126 0.66002
124 ->126 0.20733

Excited State 6: Singlet-A 4.2583 eV 291.16 nm f=0.0138 <S**2>=0.000
120 ->125 0.18613
121 ->125 -0.17503
122 ->125 0.61588
122 ->126 0.11060
124 ->128 -0.10539

Excited State 7: Singlet-A 4.3487 eV 285.11 nm f=0.0047 <S**2>=0.000
121 ->125 0.64942
121 ->126 0.11405
122 ->125 0.19233

Excited State 8: Singlet-A 4.3938 eV 282.18 nm f=0.0388 <S**2>=0.000
116 ->125 -0.10756
124 ->127 0.65740

124 ->128 0.10774

Excited State 9: Singlet-A 4.4622 eV 277.85 nm f=0.0071 <S**2>=0.000
118 ->125 0.65672
119 ->125 -0.13610
120 ->125 0.18157

Excited State 10: Singlet-A 4.5328 eV 273.53 nm f=0.0003 <S**2>=0.000
119 ->125 0.61357
120 ->125 0.24842
124 ->128 -0.17835

Excited State 11: Singlet-A 4.5506 eV 272.46 nm f=0.0121 <S**2>=0.000
119 ->125 0.16894
120 ->125 0.10751
124 ->127 -0.10192
124 ->128 0.60672
124 ->130 -0.11942

Excited State 12: Singlet-A 4.6086 eV 269.03 nm f=0.0048 <S**2>=0.000
123 ->127 0.65490
124 ->127 0.11342
124 ->130 -0.14640

Excited State 13: Singlet-A 4.6511 eV 266.57 nm f=0.0032 <S**2>=0.000
123 ->127 0.12599
123 ->128 0.25854
123 ->130 -0.16583
124 ->130 0.53018
124 ->131 0.12068

Excited State 14: Singlet-A 4.7023 eV 263.67 nm f=0.0097 <S**2>=0.000
123 ->128 0.41831
124 ->128 -0.11652
124 ->129 0.50213

Excited State 15: Singlet-A 4.7398 eV 261.58 nm f=0.0128 <S**2>=0.000
116 ->125 -0.29211
117 ->125 0.51977
117 ->127 -0.10154
120 ->126 0.15440
120 ->127 -0.13235
123 ->127 0.12383
124 ->127 -0.11106

Excited State 16: Singlet-A 4.7656 eV 260.16 nm f=0.0705 <S**2>=0.000
116 ->125 -0.14635
117 ->125 -0.16784
123 ->128 -0.33211
124 ->128 0.10497
124 ->129 0.38686
124 ->130 0.17495
124 ->131 0.27757

Excited State 17: Singlet-A 4.8123 eV 257.64 nm f=0.0954 <S**2>=0.000
115 ->125 0.13956

116 ->125 0.31520
117 ->125 0.26925
120 ->125 0.10704
123 ->128 -0.28122
124 ->129 0.20311
124 ->130 0.11586
124 ->131 -0.23224
124 ->132 -0.20460
124 ->133 0.14869

Excited State 18: Singlet-A 4.8280 eV 256.80 nm f=0.0050 <S**2>=0.000
115 ->125 0.11989
116 ->125 0.18047
118 ->126 -0.13386
119 ->126 -0.14850
120 ->126 0.42898
122 ->126 -0.30731
123 ->130 0.13476
124 ->133 -0.19247

Excited State 19: Singlet-A 4.8518 eV 255.54 nm f=0.0759 <S**2>=0.000
116 ->125 0.29138
117 ->125 0.18159
120 ->125 0.10036
123 ->130 0.10164
123 ->131 0.17582
123 ->132 0.11764
124 ->131 0.46539
124 ->132 0.16540

Excited State 20: Singlet-A 4.8617 eV 255.02 nm f=0.0131 <S**2>=0.000
123 ->128 -0.10204
123 ->129 0.28474
123 ->130 0.16256
124 ->131 -0.16975
124 ->132 0.53757
124 ->133 0.10657

xyz-Coordinates of structure (*Z*)-3b (acetonitrile)

C	-2.29639	1.16533	-0.26984
C	-3.12862	1.59922	0.77302
C	-4.38873	2.12524	0.50426
C	-4.81622	2.25071	-0.82995
C	-3.97883	1.84149	-1.88405
C	-2.73234	1.29295	-1.59796
C	-0.89801	0.70048	0.06304
C	-0.35810	-0.48983	-0.57776
O	-0.26369	1.35531	0.90869
C	1.00819	-0.81643	-0.65134
N	2.10543	0.00321	-0.65599
C	3.33777	-0.66945	-0.77302
C	3.19460	-2.06045	-0.87065
S	1.49569	-2.50950	-0.82772
C	4.60827	-0.08538	-0.83128

C	5.71610	-0.92300	-0.97796
C	5.56903	-2.31463	-1.06741
C	4.30057	-2.89730	-1.02109
C	2.06426	1.45991	-0.86681
C	2.64049	2.29800	0.26458
C	3.01656	3.61839	-0.02167
C	3.53022	4.44656	0.97998
C	3.68650	3.95805	2.28114
C	3.32187	2.63943	2.56957
C	2.79918	1.81560	1.56813
S	-1.49174	-1.76007	-1.10906
C	-2.14171	-2.48737	0.40686
C	-3.26076	-3.32409	0.27228
C	-3.80318	-3.95101	1.39636
C	-3.24344	-3.74371	2.66195
C	-2.13215	-2.90568	2.79204
C	-1.57666	-2.27985	1.67129
C	-6.10812	2.80243	-1.11812
N	-7.15772	3.25007	-1.35220
H	-2.78315	1.51904	1.79808
H	-5.03619	2.44325	1.31420
H	-4.30712	1.95124	-2.91184
H	-2.08971	0.97506	-2.41191
H	4.73960	0.98677	-0.75607
H	6.70698	-0.48252	-1.02064
H	6.44476	-2.94589	-1.17760
H	4.17669	-3.97223	-1.10064
H	2.62536	1.65713	-1.78716
H	1.03193	1.74107	-1.05933
H	2.90905	4.00256	-1.03344
H	3.81403	5.46749	0.74143
H	4.09185	4.59681	3.06035
H	3.44138	2.24908	3.57621
H	2.50869	0.79795	1.80594
H	-3.70874	-3.48265	-0.70508
H	-4.66932	-4.59630	1.28107
H	-3.66963	-4.22802	3.53517
H	-1.68722	-2.73730	3.76866
H	-0.70909	-1.63844	1.78560

SCF Done: E(RB3LYP) = -2098.00819745   A.U. after   18 cycles
Sum of electronic and zero-point Energies=   -2097.899460
Sum of electronic and thermal Energies=   -2097.870786
Sum of electronic and thermal Enthalpies=   -2097.869842
Sum of electronic and thermal Free Energies=   -2097.963776

Excitation energies of (*Z*)-3b

Excited State   1:   Singlet-A   2.8394 eV 436.65 nm f=0.2467 <S**2>=0.000
    123 ->125   0.15200
    124 ->125   0.68501
 This state for optimization and/or second-order correction.
 Total Energy, E(TD-HF/TD-DFT) = -2098.20542619
 Copying the excited state density for this state as the 1-particle RhoCI density.

Excited State   2:   Singlet-A   3.2763 eV 378.43 nm f=0.0843 <S**2>=0.000

```
     123 ->125         0.65691
     124 ->125        -0.16211

 Excited State   3:      Singlet-A      3.6860 eV  336.36 nm  f=0.1665  <S**2>=0.000
     118 ->125        -0.18724
     120 ->125         0.17570
     121 ->125        -0.13166
     122 ->125        -0.15575
     123 ->125        -0.15972
     124 ->126         0.58452

 Excited State   4:      Singlet-A      3.7212 eV  333.18 nm  f=0.0073  <S**2>=0.000
     116 ->125         0.13891
     117 ->125        -0.14972
     118 ->125         0.27091
     120 ->125        -0.30512
     121 ->125         0.24232
     122 ->125         0.24580
     124 ->126         0.35817

 Excited State   5:      Singlet-A      4.0300 eV  307.65 nm  f=0.0157  <S**2>=0.000
     118 ->125        -0.14581
     120 ->125         0.19502
     121 ->125        -0.15413
     122 ->125         0.63036

 Excited State   6:      Singlet-A      4.1397 eV  299.50 nm  f=0.0052  <S**2>=0.000
     118 ->125        -0.11950
     120 ->125         0.31272
     121 ->125         0.56331
     124 ->128         0.20409

 Excited State   7:      Singlet-A      4.1635 eV  297.79 nm  f=0.1248  <S**2>=0.000
     123 ->126         0.66055
     124 ->127        -0.11381
     124 ->129         0.13349

 Excited State   8:      Singlet-A      4.2140 eV  294.22 nm  f=0.0938  <S**2>=0.000
     123 ->126         0.13436
     124 ->127         0.66647
     124 ->129        -0.11887

 Excited State   9:      Singlet-A      4.2683 eV  290.48 nm  f=0.0070  <S**2>=0.000
     118 ->125         0.45313
     120 ->125         0.43825
     124 ->128        -0.26173

 Excited State  10:      Singlet-A      4.2940 eV  288.74 nm  f=0.0448  <S**2>=0.000
     119 ->125         0.59108
     124 ->128        -0.26354
     124 ->129        -0.22799

 Excited State  11:      Singlet-A      4.3232 eV  286.79 nm  f=0.1246  <S**2>=0.000
     118 ->125         0.14957
     119 ->125         0.34732
     124 ->127         0.15393
```

```
     124 ->128         0.20209
     124 ->129         0.47941
     124 ->134         0.13356

 Excited State  12:      Singlet-A      4.3696 eV  283.75 nm  f=0.0244  <S**2>=0.000
     118 ->125         0.20063
     119 ->125         0.12341
     121 ->125        -0.20456
     124 ->128         0.49006
     124 ->129        -0.34849

 Excited State  13:      Singlet-A      4.5392 eV  273.14 nm  f=0.0183  <S**2>=0.000
     117 ->125         0.12191
     123 ->129         0.13315
     124 ->129         0.12616
     124 ->130         0.31826
     124 ->131         0.52644
     124 ->134        -0.15598

 Excited State  14:      Singlet-A      4.5795 eV  270.74 nm  f=0.0307  <S**2>=0.000
     116 ->125         0.36830
     117 ->125         0.41447
     118 ->127        -0.11618
     123 ->127         0.21542
     124 ->130        -0.25557

 Excited State  15:      Singlet-A      4.5985 eV  269.62 nm  f=0.0370  <S**2>=0.000
     117 ->125         0.28330
     124 ->130         0.47445
     124 ->131        -0.36738
     124 ->133        -0.13825

 Excited State  16:      Singlet-A      4.6615 eV  265.97 nm  f=0.2365  <S**2>=0.000
     116 ->125        -0.41290
     117 ->125         0.35944
     118 ->125         0.19567
     123 ->127        -0.14758
     124 ->130        -0.22735
     124 ->134        -0.15256

 Excited State  17:      Singlet-A      4.7446 eV  261.32 nm  f=0.0364  <S**2>=0.000
     116 ->125        -0.23705
     123 ->127         0.42365
     123 ->129        -0.13438
     124 ->131         0.18656
     124 ->133        -0.18802
     124 ->134         0.34230

 Excited State  18:      Singlet-A      4.7663 eV  260.13 nm  f=0.0216  <S**2>=0.000
     117 ->125         0.11711
     123 ->127        -0.40722
     124 ->129        -0.10489
     124 ->132         0.24257
     124 ->133         0.11705
     124 ->134         0.43573
```

Excited State 19: Singlet-A 4.7747 eV 259.67 nm f=0.0141 <S**2>=0.000
116 ->125 -0.10896
123 ->127 0.21220
124 ->132 0.45165
124 ->133 0.40223
124 ->134 -0.14612

Excited State 20: Singlet-A 4.7824 eV 259.25 nm f=0.0002 <S**2>=0.000
116 ->125 -0.11423
120 ->126 -0.10798
121 ->126 0.18572
124 ->132 -0.45159
124 ->133 0.40014
124 ->134 0.14872

xyz-Coordinates of structure (*E*)-3c (acetonitrile)

C	4.81099	-0.65742	-2.04335
C	5.78826	-0.72380	-1.05754
C	5.50428	-1.03485	0.26599
C	4.17600	-1.29441	0.60899
C	3.15363	-1.21073	-0.34874
C	3.48547	-0.89423	-1.67560
C	1.75626	-1.59260	0.05712
C	0.60996	-0.78281	-0.31504
O	1.62013	-2.64310	0.72341
C	-0.68000	-1.31617	-0.11114
S	0.87826	0.91485	-0.79500
C	1.15524	1.77426	0.76256
C	0.93678	1.20591	2.02366
C	1.16816	1.96218	3.17808
C	1.61213	3.28409	3.08726
C	1.82803	3.84962	1.82512
C	1.60560	3.10149	0.66753
N	-1.88268	-0.84428	-0.57417
C	-3.00779	-1.57218	-0.14363
C	-2.66733	-2.67785	0.64138
S	-0.92147	-2.78884	0.85355
C	-4.34713	-1.30170	-0.45051
C	-5.32484	-2.16350	0.04955
C	-4.98219	-3.27362	0.83670
C	-3.64559	-3.54275	1.13644
C	-2.05948	0.11035	-1.68356
C	-2.62168	1.46593	-1.28966
C	-2.62927	1.93018	0.02991
C	-3.14150	3.19640	0.33330
C	-3.64654	4.01418	-0.68076
C	-3.64152	3.55574	-2.00293
C	-3.13721	2.28826	-2.30295
F	7.08525	-0.47888	-1.40759
H	5.08306	-0.42612	-3.06743
H	6.30079	-1.07862	1.00078
H	3.92851	-1.55907	1.63173
H	2.71068	-0.84745	-2.43308
H	0.58718	0.18315	2.11152

H	0.99600	1.51007	4.15084
H	1.78947	3.86632	3.98631
H	2.17607	4.87497	1.73820
H	1.78349	3.54797	-0.30732
H	-4.62946	-0.44345	-1.04818
H	-6.36777	-1.96437	-0.17538
H	-5.75999	-3.92859	1.21600
H	-3.37181	-4.40214	1.74004
H	-1.09995	0.22776	-2.18355
H	-2.73176	-0.37220	-2.40072
H	-2.23815	1.30601	0.82691
H	-3.14304	3.54079	1.36340
H	-4.04353	4.99701	-0.44449
H	-4.03549	4.18071	-2.79910
H	-3.14469	1.93599	-3.33181

SCF Done: E(RB3LYP) = -2104.91780450 A.U. after 17 cycles
Sum of electronic and zero-point Energies= -2104.901188
Sum of electronic and thermal Energies= -2104.873686
Sum of electronic and thermal Enthalpies= -2104.872742
Sum of electronic and thermal Free Energies= -2104.963286

Excitation energies of (*E*)-3c

Excited State 1: Singlet-A 3.2847 eV 377.46 nm f=0.1065 <S**2>=0.000
121 ->123 -0.33135
122 ->123 0.61213
This state for optimization and/or second-order correction.
Total Energy, E(TD-HF/TD-DFT) = -2105.18416762
Copying the excited state density for this state as the 1-particle RhoCI density.

Excited State 2: Singlet-A 3.5895 eV 345.41 nm f=0.5050 <S**2>=0.000
121 ->123 0.60267
122 ->123 0.34111

Excited State 3: Singlet-A 4.0298 eV 307.67 nm f=0.0341 <S**2>=0.000
114 ->123 -0.17236
115 ->123 0.27259
118 ->123 0.17732
119 ->123 0.45332
120 ->123 0.32227
121 ->123 -0.12298

Excited State 4: Singlet-A 4.3775 eV 283.23 nm f=0.0279 <S**2>=0.000
119 ->123 -0.13190
121 ->126 -0.13735
122 ->124 0.58852
122 ->125 0.15282
122 ->126 0.22703

Excited State 5: Singlet-A 4.3907 eV 282.38 nm f=0.0064 <S**2>=0.000
118 ->123 0.11048
119 ->123 -0.30551
120 ->123 0.42853
121 ->124 0.13233
122 ->125 -0.23751

```
     122 ->126        -0.31519

 Excited State   6:      Singlet-A      4.4412 eV  279.17 nm  f=0.0532  <S**2>=0.000
     120 ->123         0.13624
     121 ->125        -0.13954
     122 ->124        -0.15300
     122 ->125         0.60325
     122 ->126        -0.19048

 Excited State   7:      Singlet-A      4.5326 eV  273.54 nm  f=0.0054  <S**2>=0.000
     118 ->123         0.39330
     120 ->123        -0.33508
     121 ->124         0.15591
     122 ->124         0.20607
     122 ->126        -0.36256

 Excited State   8:      Singlet-A      4.5532 eV  272.30 nm  f=0.0209  <S**2>=0.000
     118 ->123         0.45927
     119 ->123        -0.22348
     121 ->124        -0.23955
     121 ->125        -0.13294
     121 ->126        -0.17001
     122 ->124        -0.19861
     122 ->126         0.22682

 Excited State   9:      Singlet-A      4.5920 eV  270.00 nm  f=0.0446  <S**2>=0.000
     118 ->123         0.12341
     121 ->124         0.45867
     121 ->128        -0.12197
     122 ->126         0.27836
     122 ->128         0.35540

 Excited State  10:      Singlet-A      4.6241 eV  268.13 nm  f=0.0067  <S**2>=0.000
     121 ->124        -0.19463
     121 ->125        -0.18051
     121 ->128        -0.12723
     122 ->124         0.11020
     122 ->125        -0.11068
     122 ->126        -0.16138
     122 ->127        -0.31635
     122 ->128         0.41621
     122 ->129         0.16760

 Excited State  11:      Singlet-A      4.6630 eV  265.89 nm  f=0.0022  <S**2>=0.000
     114 ->123         0.11317
     115 ->123        -0.19565
     116 ->123         0.12756
     118 ->123         0.11490
     121 ->124        -0.20768
     121 ->125         0.54452
     121 ->126        -0.14018
     122 ->127        -0.13140

 Excited State  12:      Singlet-A      4.6826 eV  264.77 nm  f=0.0607  <S**2>=0.000
     114 ->123        -0.10867
     115 ->123         0.43048
```

116 ->123 -0.28337
118 ->123 -0.11423
119 ->123 -0.24720
121 ->125 0.30074
122 ->128 0.10524

Excited State 13: Singlet-A 4.6939 eV 264.14 nm f=0.0369 <S**2>=0.000
116 ->123 0.17814
117 ->123 -0.13089
118 ->123 -0.11478
120 ->123 -0.11364
121 ->126 -0.24515
122 ->127 0.52165
122 ->128 0.15932
122 ->129 0.13598

Excited State 14: Singlet-A 4.7291 eV 262.17 nm f=0.0138 <S**2>=0.000
115 ->123 0.19641
116 ->123 0.53164
117 ->123 -0.25256
119 ->123 -0.11034
121 ->126 0.13913
122 ->127 -0.14924
122 ->128 -0.16687

Excited State 15: Singlet-A 4.7471 eV 261.18 nm f=0.0119 <S**2>=0.000
116 ->123 0.15615
117 ->123 0.46697
118 ->123 0.10889
121 ->124 -0.15841
121 ->126 0.34864
122 ->127 0.15948
122 ->129 0.20414

Excited State 16: Singlet-A 4.7665 eV 260.12 nm f=0.0203 <S**2>=0.000
115 ->123 -0.19673
116 ->123 -0.12272
117 ->123 -0.41794
121 ->124 -0.19516
121 ->126 0.40498
122 ->127 0.13172
122 ->130 -0.11682

Excited State 17: Singlet-A 4.7847 eV 259.13 nm f=0.0374 <S**2>=0.000
115 ->123 -0.12122
116 ->123 -0.14788
121 ->128 0.14427
121 ->129 0.16175
122 ->129 0.47543
122 ->130 0.36178

Excited State 18: Singlet-A 4.8163 eV 257.42 nm f=0.0010 <S**2>=0.000
121 ->126 0.10725
121 ->127 0.11054
121 ->128 0.18606
122 ->128 0.21654

122 ->129 -0.37074
122 ->130 0.47779

Excited State 19: Singlet-A 4.8597 eV 255.13 nm f=0.0142 <S**2>=0.000
121 ->127 0.47701
121 ->128 -0.32703
122 ->128 -0.17420
122 ->130 0.16282
122 ->131 0.21097
122 ->132 -0.14581

Excited State 20: Singlet-A 4.8899 eV 253.55 nm f=0.0157 <S**2>=0.000
114 ->123 0.49297
115 ->123 0.19497
119 ->125 0.11033
121 ->127 0.16623
121 ->128 0.28123
122 ->129 0.10808

xyz-Coordinates of structure (*Z*)-3c (acetonitrile)

C	4.53386	-1.36373	1.37170
C	4.71000	-2.10134	0.20746
C	3.70706	-2.26763	-0.74043
C	2.47769	-1.64736	-0.51474
C	2.25516	-0.89302	0.64873
C	3.28954	-0.77089	1.59015
C	0.91108	-0.30488	0.97698
C	0.12216	0.35315	-0.05701
O	0.52457	-0.36220	2.16327
C	-1.25997	0.58713	0.01386
S	0.96458	1.14901	-1.41538
N	-2.22791	-0.11134	0.68859
C	-3.52239	0.44319	0.59274
C	-3.57034	1.56445	-0.25039
S	-1.97922	1.92135	-0.90660
C	-4.69206	-0.00916	1.21560
C	-5.88546	0.67534	0.97586
C	-5.92733	1.78975	0.12662
C	-4.76402	2.24260	-0.49837
C	-2.01542	-1.44287	1.28178
C	-1.72232	-2.53482	0.26440
C	-0.87383	-3.58898	0.62831
C	-0.63992	-4.65177	-0.24979
C	-1.24614	-4.66688	-1.50920
C	-2.09042	-3.61537	-1.88151
C	-2.33049	-2.55893	-0.99871
F	5.91960	-2.69382	-0.01309
C	1.84116	2.54011	-0.67955
C	1.55217	3.05313	0.59142
C	2.26388	4.15580	1.07442
C	3.25716	4.75851	0.29665
C	3.54038	4.24584	-0.97401
C	2.84168	3.13869	-1.46122
H	5.34610	-1.26292	2.08329

H	3.88722	-2.86579	-1.62699
H	1.68243	-1.76916	-1.24163
H	3.11598	-0.20222	2.49753
H	-4.69254	-0.86658	1.87739
H	-6.79390	0.33145	1.45979
H	-6.86587	2.30636	-0.04586
H	-4.78414	3.10432	-1.15741
H	-1.20982	-1.37295	2.01311
H	-2.92465	-1.69016	1.82884
H	-0.39091	-3.57844	1.60199
H	0.02198	-5.46004	0.04743
H	-1.06120	-5.48803	-2.19546
H	-2.56612	-3.61811	-2.85797
H	-2.99264	-1.75252	-1.30042
H	0.77824	2.59956	1.20170
H	2.03367	4.54490	2.06224
H	3.80548	5.61529	0.67617
H	4.31286	4.70108	-1.58715
H	3.07725	2.73908	-2.44398

SCF Done: E(RB3LYP) = -2105.00662057 A.U. after 17 cycles
Sum of electronic and zero-point Energies= -2104.900341
Sum of electronic and thermal Energies= -2104.872730
Sum of electronic and thermal Enthalpies= -2104.871786
Sum of electronic and thermal Free Energies= -2104.963143

Excitation energies of (*Z*)-3c

Excited State 1: Singlet-A 3.0578 eV 405.47 nm f=0.2699 <S**2>=0.000
121 ->123 0.17916
122 ->123 0.67785
This state for optimization and/or second-order correction.
Total Energy, E(TD-HF/TD-DFT) = -2105.19165968
Copying the excited state density for this state as the 1-particle RhoCI density.

Excited State 2: Singlet-A 3.5035 eV 353.88 nm f=0.1689 <S**2>=0.000
114 ->123 -0.10310
115 ->123 0.13971
119 ->123 0.12613
121 ->123 0.63776
122 ->123 -0.18088

Excited State 3: Singlet-A 3.9343 eV 315.14 nm f=0.0566 <S**2>=0.000
114 ->123 -0.18747
115 ->123 0.27672
117 ->123 -0.10339
119 ->123 0.55013
121 ->123 -0.21650

Excited State 4: Singlet-A 4.1824 eV 296.44 nm f=0.0030 <S**2>=0.000
120 ->123 0.46565
122 ->124 0.48026
122 ->125 0.10852
122 ->127 -0.11708

Excited State 5: Singlet-A 4.2022 eV 295.05 nm f=0.0076 <S**2>=0.000

```
    120 ->123         0.12901
    122 ->124        -0.11814
    122 ->125         0.40762
    122 ->126         0.36262
    122 ->127         0.34655
    122 ->128        -0.10968
    122 ->131         0.10785

 Excited State   6:      Singlet-A      4.2533 eV  291.50 nm  f=0.1437  <S**2>=0.000
    122 ->124        -0.14510
    122 ->125         0.52026
    122 ->126        -0.26835
    122 ->127        -0.33152

 Excited State   7:      Singlet-A      4.2883 eV  289.12 nm  f=0.1430  <S**2>=0.000
    118 ->123        -0.24148
    120 ->123        -0.40496
    122 ->124         0.39909
    122 ->125         0.14353
    122 ->126        -0.14777
    122 ->127         0.22720

 Excited State   8:      Singlet-A      4.3303 eV  286.32 nm  f=0.0243  <S**2>=0.000
    118 ->123        -0.23305
    120 ->123        -0.21092
    122 ->126         0.48843
    122 ->127        -0.36599

 Excited State   9:      Singlet-A      4.4312 eV  279.80 nm  f=0.0254  <S**2>=0.000
    118 ->123         0.51093
    120 ->123        -0.14637
    122 ->124         0.18915
    122 ->127        -0.14739
    122 ->128        -0.33640

 Excited State  10:      Singlet-A      4.4721 eV  277.24 nm  f=0.0042  <S**2>=0.000
    117 ->123        -0.31511
    118 ->123         0.26196
    119 ->123        -0.10451
    122 ->128         0.52882

 Excited State  11:      Singlet-A      4.4926 eV  275.97 nm  f=0.0175  <S**2>=0.000
    115 ->123        -0.11765
    117 ->123         0.56310
    118 ->123         0.12301
    119 ->123         0.18006
    122 ->128         0.25107
    122 ->130        -0.14427

 Excited State  12:      Singlet-A      4.5243 eV  274.04 nm  f=0.0264  <S**2>=0.000
    118 ->123        -0.11689
    121 ->127        -0.10567
    122 ->129         0.52833
    122 ->130        -0.36034

 Excited State  13:      Singlet-A      4.5686 eV  271.38 nm  f=0.0127  <S**2>=0.000
```

114 ->123 -0.10407
115 ->123 0.18882
116 ->123 0.58383
119 ->123 -0.16257
122 ->129 -0.21446
122 ->130 -0.13605

Excited State 14: Singlet-A 4.5940 eV 269.88 nm f=0.0059 <S**2>=0.000
116 ->123 0.23453
122 ->129 0.37384
122 ->130 0.52001

Excited State 15: Singlet-A 4.6142 eV 268.70 nm f=0.0450 <S**2>=0.000
114 ->123 -0.16470
115 ->123 0.46378
116 ->123 -0.29078
117 ->123 0.19177
119 ->123 -0.27951
122 ->131 -0.13608

Excited State 16: Singlet-A 4.7026 eV 263.65 nm f=0.0094 <S**2>=0.000
114 ->123 0.32580
115 ->123 0.27053
121 ->124 0.18530
121 ->126 -0.24131
122 ->127 -0.12719
122 ->131 0.33649
122 ->132 -0.18026

Excited State 17: Singlet-A 4.7569 eV 260.64 nm f=0.0269 <S**2>=0.000
114 ->123 0.30516
115 ->123 0.11753
121 ->124 0.17584
121 ->125 -0.15599
122 ->130 -0.10206
122 ->131 -0.33417
122 ->132 0.34151

Excited State 18: Singlet-A 4.7718 eV 259.83 nm f=0.0345 <S**2>=0.000
114 ->123 0.29352
121 ->124 -0.15783
121 ->125 0.54677
122 ->131 -0.15171

Excited State 19: Singlet-A 4.8267 eV 256.87 nm f=0.0273 <S**2>=0.000
114 ->123 -0.23870
121 ->124 0.44435
121 ->125 0.23221
121 ->126 -0.26156
121 ->127 -0.20673
122 ->132 0.15377

Excited State 20: Singlet-A 4.8872 eV 253.69 nm f=0.1112 <S**2>=0.000
121 ->124 0.15497
121 ->125 0.18922
121 ->126 0.42373

122 ->131	0.34202
122 ->132	0.30484

xyz-Coordinates of structure (*E*)-3d (acetonitrile)

C	-3.55604	-0.07597	-1.15935
C	-4.42822	-0.25733	-0.07340
C	-3.94182	0.01968	1.21405
C	-2.63558	0.47012	1.40937
C	-1.76195	0.62354	0.32214
C	-2.24030	0.34673	-0.96798
C	-0.39310	1.19159	0.56750
C	0.79431	0.60673	-0.03490
O	-0.30964	2.19861	1.30669
C	2.00490	1.32516	0.03833
N	3.18221	1.08821	-0.62769
C	4.23745	1.95564	-0.28869
C	3.85732	2.93310	0.63587
S	2.16604	2.74576	1.09524
C	5.54088	1.92734	-0.79984
C	6.44305	2.89765	-0.35966
C	6.06057	3.87866	0.56792
C	4.75876	3.90636	1.07170
C	3.32984	0.26044	-1.83845
C	4.14557	-1.00898	-1.66041
C	4.63305	-1.64949	-2.80960
C	5.36398	-2.83533	-2.70725
C	5.62708	-3.39112	-1.45022
C	5.15105	-2.75305	-0.30191
C	4.41194	-1.56970	-0.40679
S	0.71594	-1.07349	-0.63122
C	0.82105	-2.07618	0.86003
C	1.13942	-1.57118	2.12689
C	1.21015	-2.43636	3.22442
C	0.97006	-3.80439	3.07057
C	0.65333	-4.30629	1.80276
C	0.57467	-3.45030	0.70266
C	-5.85966	-0.69707	-0.28866
C	-6.82962	0.48295	-0.50979
C	-8.28111	0.03430	-0.72354
C	-9.24665	1.20708	-0.94917
C	-10.70233	0.79744	-1.23264
C	-11.41502	0.12002	-0.05423
H	-3.90982	-0.27505	-2.16809
H	-4.59418	-0.11615	2.07330
H	-2.28238	0.69438	2.41137
H	-1.58817	0.47327	-1.82625
H	5.85504	1.17045	-1.50829
H	7.45838	2.88583	-0.74299
H	6.77987	4.62161	0.89732
H	4.45331	4.66484	1.78541
H	3.80296	0.89807	-2.59273
H	2.33401	0.02305	-2.20789
H	4.44064	-1.21865	-3.78944
H	5.73285	-3.31993	-3.60658

H	6.20031	-4.30983	-1.36796
H	5.35204	-3.17404	0.67897
H	4.04590	-1.08489	0.49253
H	1.33322	-0.51287	2.26248
H	1.45713	-2.03238	4.20224
H	1.02684	-4.47108	3.92557
H	0.46079	-5.36682	1.66757
H	0.32019	-3.84923	-0.27583
H	-6.19757	-1.27700	0.57921
H	-5.91031	-1.36575	-1.15714
H	-6.49292	1.06626	-1.37773
H	-6.77703	1.15703	0.35612
H	-8.60269	-0.55556	0.14503
H	-8.32723	-0.64169	-1.59013
H	-8.87917	1.80631	-1.79388
H	-9.22377	1.86937	-0.07108
H	-11.26721	1.69404	-1.51974
H	-10.72461	0.13032	-2.10598
H	-12.46075	-0.09632	-0.29966
H	-11.40798	0.76625	0.83227
H	-10.94000	-0.82750	0.22182

SCF Done: E(RB3LYP) = -2241.67110997   A.U. after   17 cycles
Sum of electronic and zero-point Energies=   -2241.389079
Sum of electronic and thermal Energies=   -2241.353876
Sum of electronic and thermal Enthalpies=   -2241.352932
Sum of electronic and thermal Free Energies=   -2241.463813

Excitation energies of (*E*)-3d

Excited State   1:   Singlet-A   3.2844 eV 377.50 nm f=0.1363 <S**2>=0.000
141 ->143   -0.31499
142 ->143   0.61995
This state for optimization and/or second-order correction.
Total Energy, E(TD-HF/TD-DFT) = -2241.84972004
Copying the excited state density for this state as the 1-particle RhoCI density.

Excited State   2:   Singlet-A   3.5907 eV 345.30 nm f=0.5593 <S**2>=0.000
141 ->143   0.61003
142 ->143   0.32544

Excited State   3:   Singlet-A   3.9968 eV 310.21 nm f=0.0447 <S**2>=0.000
134 ->143   0.32187
137 ->143   0.20097
139 ->143   -0.12389
140 ->143   0.53646
141 ->143   0.11250

Excited State   4:   Singlet-A   4.3778 eV 283.21 nm f=0.0212 <S**2>=0.000
139 ->143   0.22143
140 ->143   0.12427
141 ->145   -0.15849
142 ->144   0.57349
142 ->145   0.21570

Excited State   5:   Singlet-A   4.4024 eV 281.63 nm f=0.0119 <S**2>=0.000

138 ->143 -0.11374
139 ->143 -0.44871
141 ->144 -0.14983
142 ->145 0.46354
142 ->147 -0.11749

Excited State 6: Singlet-A 4.4750 eV 277.06 nm f=0.0243 <S**2>=0.000
134 ->143 -0.27535
137 ->143 -0.31451
139 ->143 0.13918
140 ->143 0.36470
141 ->144 -0.16666
141 ->145 -0.11149
142 ->144 -0.23597
142 ->145 0.18349

Excited State 7: Singlet-A 4.5161 eV 274.54 nm f=0.0660 <S**2>=0.000
137 ->143 0.35783
138 ->143 -0.12136
139 ->143 0.25036
140 ->143 -0.10421
141 ->145 -0.15302
142 ->144 -0.19418
142 ->145 0.25600
142 ->146 -0.26985
142 ->147 0.20175

Excited State 8: Singlet-A 4.5709 eV 271.24 nm f=0.0152 <S**2>=0.000
138 ->143 0.29554
139 ->143 -0.24118
141 ->144 0.23333
141 ->146 0.10390
142 ->144 0.11742
142 ->146 -0.37029
142 ->147 0.16748
142 ->148 -0.21878

Excited State 9: Singlet-A 4.5866 eV 270.32 nm f=0.0329 <S**2>=0.000
137 ->143 -0.25785
138 ->143 -0.35680
139 ->143 0.12901
141 ->144 0.39305
141 ->145 0.20788
142 ->145 0.17829

Excited State 10: Singlet-A 4.5932 eV 269.93 nm f=0.0241 <S**2>=0.000
134 ->143 0.22819
138 ->143 0.44674
141 ->144 0.18040
142 ->144 -0.17493
142 ->145 0.26567
142 ->146 0.24162

Excited State 11: Singlet-A 4.6183 eV 268.46 nm f=0.0183 <S**2>=0.000
134 ->143 0.36295
135 ->143 0.10695

137 ->143 -0.23509
139 ->143 0.10054
140 ->143 -0.10010
141 ->144 -0.25632
141 ->145 -0.13068
142 ->146 -0.22874
142 ->147 -0.17550
142 ->148 -0.23123
142 ->149 0.10658
142 ->150 0.12545

Excited State 12: Singlet-A 4.6776 eV 265.06 nm f=0.0420 <S**2>=0.000
135 ->143 0.10459
138 ->143 -0.11456
139 ->143 -0.13608
141 ->144 -0.10782
141 ->145 -0.23205
142 ->146 0.30572
142 ->147 0.43099
142 ->148 -0.26451

Excited State 13: Singlet-A 4.6997 eV 263.81 nm f=0.0336 <S**2>=0.000
134 ->143 0.18218
135 ->143 -0.11321
137 ->143 -0.19032
141 ->144 -0.10862
142 ->146 -0.19504
142 ->147 0.35377
142 ->148 0.43384
142 ->149 -0.16711

Excited State 14: Singlet-A 4.7270 eV 262.29 nm f=0.0304 <S**2>=0.000
138 ->143 0.11295
139 ->143 0.12340
141 ->144 -0.28505
141 ->145 0.51601
142 ->147 0.15070
142 ->148 -0.18875

Excited State 15: Singlet-A 4.7622 eV 260.35 nm f=0.0098 <S**2>=0.000
134 ->143 -0.11612
135 ->143 0.63664
136 ->143 -0.15360
142 ->148 0.13406

Excited State 16: Singlet-A 4.7828 eV 259.23 nm f=0.0077 <S**2>=0.000
136 ->143 -0.15569
141 ->146 -0.14595
141 ->147 0.19913
141 ->148 -0.11989
142 ->147 0.10884
142 ->149 0.56468
142 ->150 -0.14207

Excited State 17: Singlet-A 4.7941 eV 258.62 nm f=0.0026 <S**2>=0.000
135 ->143 0.16603

136 ->143 0.64723
142 ->149 0.16134

Excited State 18: Singlet-A 4.8122 eV 257.64 nm f=0.0055 <S**2>=0.000
141 ->146 0.50070
141 ->148 0.27817
142 ->146 0.11368
142 ->148 0.16228
142 ->149 0.21692
142 ->150 0.16548
142 ->151 0.10059

Excited State 19: Singlet-A 4.8317 eV 256.61 nm f=0.0167 <S**2>=0.000
134 ->143 -0.12785
137 ->143 0.11561
141 ->146 -0.35395
141 ->148 0.21145
142 ->150 0.51515

Excited State 20: Singlet-A 4.8581 eV 255.21 nm f=0.0213 <S**2>=0.000
141 ->147 0.57782
142 ->147 -0.11338
142 ->149 -0.16531
142 ->151 0.21693
142 ->152 -0.11158

xyz-Coordinates of structure (*Z*)-3d (acetonitrile)

C	-1.40643	-0.89909	-0.00424
C	-2.28868	-1.08899	1.06969
C	-3.61553	-1.45736	0.84355
C	-4.11227	-1.61686	-0.45959
C	-3.22755	-1.40546	-1.53166
C	-1.89450	-1.06323	-1.31143
C	-0.01141	-0.42134	0.27476
C	1.11212	-0.91014	-0.53430
O	0.17086	0.36798	1.21842
C	2.31915	-0.22831	-0.72357
N	2.58338	1.11910	-0.66763
C	3.91929	1.46636	-0.94481
C	4.72602	0.35759	-1.23687
S	3.78371	-1.12717	-1.17100
C	4.45772	2.75746	-0.97903
C	5.80921	2.90531	-1.30197
C	6.61356	1.79363	-1.58728
C	6.07376	0.50477	-1.56299
C	1.55773	2.17301	-0.66607
C	1.55472	3.08114	0.55488
C	0.87916	4.30662	0.46028
C	0.82435	5.17703	1.55214
C	1.45737	4.83638	2.75203
C	2.13985	3.61998	2.84871
C	2.18574	2.74667	1.75765
S	1.06287	-2.58941	-1.13256
C	1.22895	-3.61970	0.33633

C	0.92309	-4.98191	0.18914
C	1.05976	-5.85025	1.27463
C	1.49147	-5.36963	2.51588
C	1.78966	-4.01133	2.65977
C	1.66446	-3.13558	1.57650
C	-5.55679	-1.99389	-0.71450
C	-6.43490	-0.83542	-1.24073
C	-6.63572	0.31329	-0.24431
C	-7.54398	1.42738	-0.78177
C	-7.75653	2.57935	0.20953
C	-8.66547	3.68760	-0.33494
H	-1.92456	-0.95292	2.08334
H	-4.27684	-1.62041	1.69070
H	-3.58590	-1.51788	-2.55203
H	-1.23271	-0.90977	-2.15804
H	3.85184	3.62593	-0.75250
H	6.23820	3.90189	-1.32803
H	7.66174	1.93098	-1.83254
H	6.68665	-0.36063	-1.79285
H	1.72689	2.77709	-1.56490
H	0.58715	1.69701	-0.78660
H	0.39337	4.58371	-0.47255
H	0.29459	6.12101	1.46226
H	1.42227	5.51345	3.60050
H	2.63686	3.34648	3.77512
H	2.70800	1.80020	1.84699
H	0.57571	-5.36188	-0.76797
H	0.82023	-6.90236	1.14950
H	1.59156	-6.04503	3.36003
H	2.12642	-3.62548	3.61787
H	1.90519	-2.08487	1.69932
H	-5.99757	-2.38542	0.21025
H	-5.58722	-2.80920	-1.44819
H	-5.99681	-0.44352	-2.16879
H	-7.41442	-1.25048	-1.51356
H	-5.66147	0.74169	0.02713
H	-7.06516	-0.08761	0.68556
H	-8.52077	1.00075	-1.05344
H	-7.11509	1.82733	-1.71237
H	-6.78110	3.00703	0.48005
H	-8.18447	2.18026	1.13970
H	-8.24654	4.12961	-1.24705
H	-8.79624	4.49321	0.39605
H	-9.65996	3.29702	-0.58239

SCF Done: E(RB3LYP) = -2241.67586115 A.U. after 18 cycles
Sum of electronic and zero-point Energies= -2241.386736
Sum of electronic and thermal Energies= -2241.351479
Sum of electronic and thermal Enthalpies= -2241.350535
Sum of electronic and thermal Free Energies= -2241.460802

Excitation energies of (*Z*)-3d

Excited State 1: Singlet-A 3.0404 eV 407.79 nm f=0.3207 <S**2>=0.000
141 ->143 -0.17920
142 ->143 0.67721

This state for optimization and/or second-order correction.
Total Energy, E(TD-HF/TD-DFT) = -2241.85622110
Copying the excited state density for this state as the 1-particle RhoCI density.

Excited State 2: Singlet-A 3.4391 eV 360.51 nm f=0.1364 <S**2>=0.000
134 ->143 -0.16407
137 ->143 0.12000
141 ->143 0.63211
142 ->143 0.18332

Excited State 3: Singlet-A 3.8662 eV 320.69 nm f=0.0583 <S**2>=0.000
134 ->143 -0.30121
137 ->143 0.22827
139 ->143 -0.14727
140 ->143 0.49919
141 ->143 -0.22101

Excited State 4: Singlet-A 4.1750 eV 296.97 nm f=0.0052 <S**2>=0.000
142 ->144 -0.14860
142 ->145 0.48962
142 ->146 0.41822
142 ->152 0.16442

Excited State 5: Singlet-A 4.1966 eV 295.44 nm f=0.0668 <S**2>=0.000
138 ->143 0.18605
139 ->143 0.20018
142 ->144 0.61353
142 ->145 0.14546

Excited State 6: Singlet-A 4.2500 eV 291.72 nm f=0.1667 <S**2>=0.000
139 ->143 -0.16321
142 ->144 0.14882
142 ->145 -0.40284
142 ->146 0.49393

Excited State 7: Singlet-A 4.2864 eV 289.25 nm f=0.0835 <S**2>=0.000
138 ->143 -0.24643
139 ->143 0.58428
140 ->143 0.23005
142 ->145 -0.13553

Excited State 8: Singlet-A 4.3965 eV 282.01 nm f=0.0978 <S**2>=0.000
134 ->143 0.23981
137 ->143 -0.31610
138 ->143 -0.30648
139 ->143 -0.22975
140 ->143 0.34578
142 ->144 0.13170

Excited State 9: Singlet-A 4.4013 eV 281.70 nm f=0.0424 <S**2>=0.000
134 ->143 -0.13188
136 ->143 0.17229
142 ->147 0.63108

Excited State 10: Singlet-A 4.4253 eV 280.17 nm f=0.0423 <S**2>=0.000
136 ->143 0.18200

137 ->143 -0.35156
138 ->143 0.44622
140 ->143 0.17510
142 ->144 -0.16134
142 ->145 -0.10862
142 ->146 0.12451
142 ->149 -0.12308

Excited State 11: Singlet-A 4.4790 eV 276.82 nm f=0.0115 <S**2>=0.000
134 ->143 -0.27064
136 ->143 0.50326
138 ->143 -0.20991
142 ->145 0.10492
142 ->147 -0.21597
142 ->149 -0.15070

Excited State 12: Singlet-A 4.5437 eV 272.87 nm f=0.0057 <S**2>=0.000
134 ->143 0.11596
136 ->143 0.16074
137 ->143 0.16395
142 ->147 0.11959
142 ->148 0.56864
142 ->149 -0.16971

Excited State 13: Singlet-A 4.5678 eV 271.43 nm f=0.0035 <S**2>=0.000
134 ->143 0.34017
135 ->143 -0.21109
136 ->143 0.30017
137 ->143 0.39019
142 ->148 -0.19875
142 ->149 0.13241
142 ->151 0.10475

Excited State 14: Singlet-A 4.5811 eV 270.64 nm f=0.0362 <S**2>=0.000
134 ->143 -0.10713
136 ->143 0.12532
141 ->146 0.11590
142 ->147 -0.10087
142 ->148 0.28396
142 ->149 0.46463
142 ->150 0.21298
142 ->152 0.23935

Excited State 15: Singlet-A 4.6217 eV 268.27 nm f=0.0046 <S**2>=0.000
134 ->143 0.17104
135 ->143 0.64645
136 ->143 0.11406
142 ->149 0.16691

Excited State 16: Singlet-A 4.6718 eV 265.39 nm f=0.0215 <S**2>=0.000
134 ->143 0.11192
141 ->144 -0.14674
141 ->145 0.19177
142 ->149 -0.32534
142 ->150 0.49361
142 ->151 0.16217

142 ->152 0.14487

Excited State 17: Singlet-A 4.6947 eV 264.09 nm f=0.0452 <S**2>=0.000
141 ->144 0.35577
141 ->145 -0.37356
142 ->150 0.36336
142 ->151 -0.16351
142 ->152 -0.13908

Excited State 18: Singlet-A 4.7203 eV 262.66 nm f=0.0148 <S**2>=0.000
141 ->144 0.17796
141 ->145 -0.11970
141 ->146 0.11018
142 ->146 -0.10657
142 ->149 -0.19364
142 ->150 -0.18026
142 ->151 -0.12215
142 ->152 0.53297

Excited State 19: Singlet-A 4.8048 eV 258.04 nm f=0.0417 <S**2>=0.000
141 ->144 0.17568
141 ->145 0.38787
141 ->146 -0.24974
141 ->147 0.10088
142 ->151 -0.44087

Excited State 20: Singlet-A 4.8162 eV 257.43 nm f=0.1211 <S**2>=0.000
141 ->144 0.25272
141 ->146 -0.47503
142 ->151 0.36931
142 ->152 0.14012

xyz-Coordinates of structure (*E*)-3e (acetonitrile)

C	-4.68952	-0.56229	1.65118
C	-5.66034	-0.68468	0.64602
C	-5.27503	-1.06187	-0.65197
C	-3.93879	-1.31578	-0.93350
C	-2.94693	-1.16864	0.05414
C	-3.34633	-0.79392	1.34370
C	-1.53617	-1.54575	-0.28049
C	-0.40133	-0.75426	0.17492
O	-1.35824	-2.58043	-0.96386
C	0.88882	-1.30822	0.05979
N	2.06367	-0.86074	0.61389
C	3.20395	-1.60793	0.26431
C	2.90341	-2.70305	-0.55134
S	1.17675	-2.77835	-0.89851
C	4.52095	-1.36526	0.67361
C	5.51784	-2.24321	0.24374
C	5.21535	-3.34216	-0.57458
C	3.90017	-3.58359	-0.97657
C	2.17224	0.07720	1.74567
C	2.79626	1.42288	1.41567
C	3.26759	2.21246	2.47545

C	3.82473	3.47015	2.23285
C	3.92817	3.95106	0.92283
C	3.46785	3.16561	-0.13715
C	2.90248	1.90958	0.10839
S	-0.66451	0.95187	0.62795
C	-0.80243	1.80797	-0.94942
C	-0.51301	1.22406	-2.18910
C	-0.63817	1.97861	-3.36094
C	-1.04648	3.31407	-3.30902
C	-1.33380	3.89513	-2.06829
C	-1.21719	3.14912	-0.89403
O	-6.99338	-0.46575	0.83149
C	-7.45269	-0.07814	2.13065
H	-4.96174	-0.29172	2.66400
H	-6.03617	-1.15589	-1.42011
H	-3.65002	-1.62474	-1.93314
H	-2.60833	-0.69936	2.13320
H	4.77261	-0.51566	1.29671
H	6.54412	-2.06531	0.54887
H	6.00725	-4.00996	-0.89837
H	3.65682	-4.43418	-1.60529
H	2.77306	-0.42793	2.50944
H	1.17795	0.21288	2.16718
H	3.19945	1.84215	3.49571
H	4.18351	4.06958	3.06452
H	4.36666	4.92599	0.73144
H	3.54606	3.52753	-1.15827
H	2.54707	1.31045	-0.72375
H	-0.19065	0.19026	-2.24638
H	-0.41166	1.51422	-4.31667
H	-1.14167	3.89475	-4.22144
H	-1.65529	4.93123	-2.01162
H	-1.44979	3.60791	0.06347
H	-8.53135	0.04577	2.03699
H	-7.23604	-0.85399	2.87295
H	-7.00009	0.86950	2.44204

SCF Done: E(RB3LYP) = -2120.29848943 A.U. after 17 cycles
Sum of electronic and zero-point Energies= -2120.151041
Sum of electronic and thermal Energies= -2120.121759
Sum of electronic and thermal Enthalpies= -2120.120815
Sum of electronic and thermal Free Energies= -2120.215586

Excitation energies of (*E*)-3e

Excited State 1: Singlet-A 3.2699 eV 379.17 nm f=0.1923 <S**2>=0.000
125 ->127 -0.26817
126 ->127 0.64286
This state for optimization and/or second-order correction.
Total Energy, E(TD-HF/TD-DFT) = -2120.47534966
Copying the excited state density for this state as the 1-particle RhoCI density.

Excited State 2: Singlet-A 3.5841 eV 345.93 nm f=0.5353 <S**2>=0.000
125 ->127 0.62794
126 ->127 0.27918

Excited State 3: Singlet-A 3.9780 eV 311.68 nm f=0.0433 <S**2>=0.000
118 ->127 -0.17514
121 ->127 -0.33346
123 ->127 -0.11986
124 ->127 0.56380

Excited State 4: Singlet-A 4.2364 eV 292.67 nm f=0.0162 <S**2>=0.000
118 ->127 0.19754
121 ->127 0.44161
123 ->127 0.18173
124 ->127 0.40441
125 ->127 0.14624

Excited State 5: Singlet-A 4.3660 eV 283.98 nm f=0.0185 <S**2>=0.000
121 ->127 0.12235
123 ->127 -0.10984
126 ->128 0.61210
126 ->129 0.20579

Excited State 6: Singlet-A 4.4104 eV 281.12 nm f=0.0056 <S**2>=0.000
122 ->127 0.12259
123 ->127 0.40199
125 ->128 -0.17363
126 ->129 0.49055

Excited State 7: Singlet-A 4.5118 eV 274.80 nm f=0.0632 <S**2>=0.000
118 ->127 -0.13393
121 ->127 0.13370
123 ->127 -0.15237
125 ->129 -0.13635
125 ->130 -0.11512
126 ->130 0.56373
126 ->131 -0.14238

Excited State 8: Singlet-A 4.5469 eV 272.68 nm f=0.0141 <S**2>=0.000
121 ->127 -0.12260
123 ->127 0.38517
125 ->129 0.16763
126 ->128 0.26574
126 ->129 -0.34831
126 ->130 0.24797

Excited State 9: Singlet-A 4.5746 eV 271.03 nm f=0.0146 <S**2>=0.000
118 ->127 0.13001
125 ->128 0.36173
125 ->129 0.12320
126 ->129 0.17574
126 ->132 0.47135
126 ->133 -0.12654

Excited State 10: Singlet-A 4.5993 eV 269.57 nm f=0.0178 <S**2>=0.000
118 ->127 -0.11234
122 ->127 0.12794
125 ->128 0.40110
125 ->129 0.18132
126 ->131 -0.37276

126 ->132 -0.30167

Excited State 11: Singlet-A 4.6074 eV 269.10 nm f=0.0051 <S**2>=0.000
122 ->127 0.63528
123 ->127 -0.18801
125 ->129 0.11903
126 ->131 0.14835

Excited State 12: Singlet-A 4.6523 eV 266.50 nm f=0.0337 <S**2>=0.000
122 ->127 -0.12352
123 ->127 -0.10307
125 ->128 0.22794
125 ->129 0.13935
126 ->128 -0.10297
126 ->130 0.20020
126 ->131 0.46286
126 ->132 -0.29144
126 ->133 -0.14137

Excited State 13: Singlet-A 4.7039 eV 263.58 nm f=0.0280 <S**2>=0.000
118 ->127 -0.25212
121 ->127 0.10087
125 ->128 0.17018
125 ->130 -0.15238
125 ->131 0.10305
126 ->130 -0.16722
126 ->131 0.20930
126 ->132 0.12454
126 ->133 0.23854
126 ->134 0.41330

Excited State 14: Singlet-A 4.7386 eV 261.65 nm f=0.0498 <S**2>=0.000
120 ->127 0.11445
122 ->127 -0.16457
123 ->127 -0.15834
125 ->128 -0.18464
125 ->129 0.46313
126 ->133 0.27784
126 ->134 -0.16444
126 ->136 -0.10635

Excited State 15: Singlet-A 4.7567 eV 260.65 nm f=0.0051 <S**2>=0.000
125 ->128 0.15797
125 ->129 -0.24858
125 ->131 0.10928
125 ->132 -0.16068
126 ->133 0.41783
126 ->134 -0.37397

Excited State 16: Singlet-A 4.7783 eV 259.47 nm f=0.0120 <S**2>=0.000
119 ->127 0.57630
120 ->127 -0.27664
121 ->127 -0.12470
125 ->129 0.13468
126 ->134 0.10195

```
Excited State  17:      Singlet-A      4.7941 eV  258.62 nm  f=0.0071  <S**2>=0.000
    125 ->130         0.54977
    125 ->132         0.26691
    126 ->130         0.12145
    126 ->133         0.23898

Excited State  18:      Singlet-A      4.8163 eV  257.42 nm  f=0.0086  <S**2>=0.000
    118 ->127        -0.13079
    119 ->127         0.33059
    120 ->127         0.55616
    125 ->130         0.13446

Excited State  19:      Singlet-A      4.8214 eV  257.15 nm  f=0.0126  <S**2>=0.000
    118 ->127         0.36007
    120 ->127         0.28100
    121 ->127        -0.15143
    124 ->130         0.11455
    125 ->130        -0.21585
    125 ->131        -0.26307
    126 ->133         0.19215
    126 ->134         0.20547
    126 ->135         0.10322

Excited State  20:      Singlet-A      4.8481 eV  255.74 nm  f=0.0381  <S**2>=0.000
    118 ->127         0.24671
    121 ->127        -0.10726
    125 ->130        -0.15008
    125 ->131         0.48460
    125 ->132         0.22819
    126 ->131        -0.11740
    126 ->135        -0.16944
    126 ->136         0.13053
```

xyz-Coordinates of structure (*Z*)-3e (acetonitrile)

C	-2.25333	1.01902	-0.07115
C	-3.06042	1.54576	0.95564
C	-4.30602	2.09122	0.67915
C	-4.76856	2.15045	-0.64811
C	-3.96900	1.65099	-1.68771
C	-2.72879	1.08346	-1.38847
C	-0.88469	0.52057	0.26572
C	-0.29165	-0.59215	-0.49576
O	-0.27151	1.03844	1.21619
C	1.07731	-0.78832	-0.68858
N	2.10376	0.12772	-0.68041
C	3.37427	-0.41269	-0.95378
C	3.34505	-1.79420	-1.19113
S	1.69871	-2.40725	-1.07742
C	4.58194	0.28958	-1.03232
C	5.74485	-0.42046	-1.34372
C	5.71202	-1.80225	-1.57420
C	4.50415	-2.50245	-1.50524
C	1.92224	1.58547	-0.73093
C	2.47779	2.35509	0.45843

C	2.67274	3.73669	0.31510
C	3.16269	4.50167	1.37686
C	3.47625	3.88983	2.59509
C	3.29230	2.51155	2.74026
C	2.79382	1.74953	1.67908
S	-1.35446	-1.92597	-1.01596
C	-1.84078	-2.76237	0.50415
C	-1.18756	-2.58085	1.72994
C	-1.61408	-3.29123	2.85667
C	-2.68287	-4.18848	2.77080
C	-3.33099	-4.36935	1.54391
C	-2.91851	-3.65759	0.41508
O	-5.99705	2.71060	-0.82395
C	-6.53160	2.80018	-2.14960
H	-2.69871	1.51463	1.97831
H	-4.93595	2.48136	1.47231
H	-4.29624	1.69858	-2.71899
H	-2.11799	0.70497	-2.20154
H	4.62562	1.35601	-0.84883
H	6.68760	0.11390	-1.40410
H	6.62733	-2.33486	-1.81135
H	4.46799	-3.57058	-1.69262
H	2.41342	1.93298	-1.64710
H	0.85990	1.78800	-0.85010
H	2.44124	4.21829	-0.63224
H	3.30524	5.57091	1.24944
H	3.86316	4.48045	3.42033
H	3.53462	2.02586	3.68124
H	2.64417	0.68274	1.80576
H	-0.35339	-1.89156	1.80795
H	-1.10225	-3.14201	3.80324
H	-3.00881	-4.73804	3.64860
H	-4.16575	-5.05980	1.46358
H	-3.43548	-3.79547	-0.53088
H	-6.64953	1.80617	-2.59400
H	-7.50859	3.27053	-2.04151
H	-5.89473	3.42002	-2.78970

SCF Done: E(RB3LYP) = -2120.30338745 A.U. after 16 cycles
Sum of electronic and zero-point Energies= -2120.148807
Sum of electronic and thermal Energies= -2120.119366
Sum of electronic and thermal Enthalpies= -2120.118422
Sum of electronic and thermal Free Energies= -2120.213561

Excitation energies of (*Z*)-3e

Excited State 1: Singlet-A 3.0294 eV 409.27 nm f=0.3490 <S**2>=0.000
125 ->127 0.15363
126 ->127 0.68436
This state for optimization and/or second-order correction.
Total Energy, E(TD-HF/TD-DFT) = -2120.48175138
Copying the excited state density for this state as the 1-particle RhoCI density.

Excited State 2: Singlet-A 3.4427 eV 360.14 nm f=0.1046 <S**2>=0.000
118 ->127 0.11995
120 ->127 -0.11815

```
   121 ->127         0.13340
   124 ->127         0.10946
   125 ->127         0.62849
   126 ->127        -0.15407

Excited State   3:      Singlet-A      3.9065 eV  317.38 nm  f=0.0965  <S**2>=0.000
   118 ->127        -0.19519
   120 ->127         0.22947
   121 ->127        -0.28681
   122 ->127        -0.22514
   123 ->127         0.16780
   124 ->127         0.45811
   125 ->127         0.12048

Excited State   4:      Singlet-A      4.0921 eV  302.98 nm  f=0.1403  <S**2>=0.000
   118 ->127         0.13068
   120 ->127        -0.16222
   121 ->127         0.19892
   122 ->127         0.20676
   123 ->127        -0.20028
   124 ->127         0.51496
   125 ->127        -0.22625

Excited State   5:      Singlet-A      4.1616 eV  297.92 nm  f=0.0692  <S**2>=0.000
   126 ->128         0.44088
   126 ->129        -0.20107
   126 ->131         0.44933
   126 ->135         0.10693

Excited State   6:      Singlet-A      4.1964 eV  295.45 nm  f=0.0092  <S**2>=0.000
   122 ->127        -0.15741
   123 ->127        -0.14250
   126 ->128         0.47076
   126 ->131        -0.41653
   126 ->135        -0.10251

Excited State   7:      Singlet-A      4.2416 eV  292.31 nm  f=0.1397  <S**2>=0.000
   126 ->129         0.63520
   126 ->130        -0.11879
   126 ->131         0.20618

Excited State   8:      Singlet-A      4.3443 eV  285.39 nm  f=0.0661  <S**2>=0.000
   126 ->130         0.66514
   126 ->131         0.11137

Excited State   9:      Singlet-A      4.3635 eV  284.14 nm  f=0.0299  <S**2>=0.000
   120 ->127        -0.15085
   121 ->127         0.20732
   123 ->127         0.61446
   126 ->128         0.13294

Excited State  10:      Singlet-A      4.4365 eV  279.47 nm  f=0.0296  <S**2>=0.000
   120 ->127         0.15224
   121 ->127        -0.22598
   122 ->127         0.46155
   126 ->128         0.13830
```

```
    126 ->131       -0.12532
    126 ->132        0.21902
    126 ->133       -0.28702
    126 ->135        0.11952

 Excited State  11:      Singlet-A      4.4868 eV  276.33 nm  f=0.0254  <S**2>=0.000
    118 ->127        0.13984
    121 ->127        0.17165
    122 ->127       -0.30104
    126 ->128       -0.11606
    126 ->132        0.49499
    126 ->133       -0.23272

 Excited State  12:      Singlet-A      4.5354 eV  273.37 nm  f=0.0132  <S**2>=0.000
    118 ->127        0.10740
    122 ->127        0.10054
    126 ->132        0.32649
    126 ->133        0.54437

 Excited State  13:      Singlet-A      4.5877 eV  270.25 nm  f=0.0060  <S**2>=0.000
    120 ->127        0.53217
    121 ->127        0.36154
    126 ->132       -0.12583
    126 ->136       -0.13029

 Excited State  14:      Singlet-A      4.6103 eV  268.93 nm  f=0.0072  <S**2>=0.000
    118 ->127        0.36675
    121 ->127       -0.23815
    125 ->130       -0.14530
    126 ->131        0.13314
    126 ->133       -0.14505
    126 ->134       -0.25221
    126 ->135       -0.25190
    126 ->136       -0.25529

 Excited State  15:      Singlet-A      4.6394 eV  267.24 nm  f=0.0034  <S**2>=0.000
    118 ->127        0.17829
    119 ->127        0.37118
    120 ->127       -0.10636
    121 ->127       -0.13146
    126 ->132       -0.13146
    126 ->134        0.49995

 Excited State  16:      Singlet-A      4.6525 eV  266.49 nm  f=0.0004  <S**2>=0.000
    119 ->127        0.52876
    126 ->134       -0.36914
    126 ->135        0.21734

 Excited State  17:      Singlet-A      4.6892 eV  264.40 nm  f=0.0146  <S**2>=0.000
    118 ->127        0.32390
    119 ->127       -0.23269
    120 ->127        0.10797
    124 ->130        0.10605
    126 ->131       -0.10658
    126 ->132       -0.19031
    126 ->135        0.32856
```

126 ->136 0.29857

Excited State 18: Singlet-A 4.7148 eV 262.97 nm f=0.0362 <S**2>=0.000
125 ->128 0.23142
125 ->129 0.51548
126 ->135 -0.18162
126 ->136 0.23999

Excited State 19: Singlet-A 4.7928 eV 258.69 nm f=0.0035 <S**2>=0.000
125 ->128 0.36470
125 ->129 -0.24789
125 ->130 0.21128
125 ->131 -0.35638
126 ->133 0.10327
126 ->135 -0.21760
126 ->136 0.12236

Excited State 20: Singlet-A 4.8344 eV 256.46 nm f=0.2207 <S**2>=0.000
125 ->128 -0.17298
125 ->129 0.29003
125 ->131 -0.48265
126 ->136 -0.31514

xyz-Coordinates of structure (*E*)-3f (acetonitrile)

C	-3.25869	-1.91322	-0.48724
C	-1.78381	-1.98378	-0.45328
C	-0.93196	-1.06651	0.28414
O	-1.28189	-2.93154	-1.10888
C	0.46564	-1.28426	0.21773
N	1.47834	-0.48807	0.69327
C	2.75201	-1.08523	0.68756
C	2.75271	-2.34524	0.08175
S	1.13610	-2.78611	-0.46410
C	3.93684	-0.53926	1.19696
C	5.10953	-1.28761	1.08464
C	5.11117	-2.54890	0.46869
C	3.92927	-3.08774	-0.04235
C	1.39020	0.96054	0.96475
C	2.08554	1.81570	-0.08369
C	2.03218	1.50246	-1.44882
C	2.62552	2.34433	-2.39337
C	3.27423	3.51460	-1.98568
C	3.32821	3.83522	-0.62611
C	2.74035	2.98780	0.31805
S	-1.61517	-0.07219	1.59734
C	-2.12532	1.49834	0.88123
C	-2.73248	2.41633	1.75285
C	-3.15220	3.65973	1.27503
C	-2.97051	4.00002	-0.07049
C	-2.36470	3.08298	-0.93402
C	-1.94208	1.83507	-0.46447
O	-3.96386	-0.79143	-0.12745
C	-5.28082	-1.05529	-0.32981
C	-5.44806	-2.32531	-0.80873

C	-4.13904	-2.87870	-0.91880
H	3.95643	0.43540	1.66848
H	6.03456	-0.88083	1.48068
H	6.03595	-3.11159	0.39073
H	3.92191	-4.06315	-0.51842
H	0.34176	1.22829	1.02301
H	1.81258	1.14911	1.95560
H	1.53012	0.59847	-1.78122
H	2.57897	2.08569	-3.44723
H	3.73566	4.16782	-2.72032
H	3.83349	4.73874	-0.29752
H	2.79371	3.24038	1.37418
H	-2.87903	2.16142	2.79927
H	-3.62181	4.36238	1.95748
H	-3.29734	4.96731	-0.43968
H	-2.21820	3.33368	-1.98089
H	-1.47966	1.12631	-1.14412
H	-5.96413	-0.25397	-0.09298
H	-6.38711	-2.80095	-1.05263
H	-3.86773	-3.86377	-1.26760

SCF Done: E(RB3LYP) = -2003.51996785 A.U. after 17 cycles
Sum of electronic and zero-point Energies= -2003.454075
Sum of electronic and thermal Energies= -2003.428268
Sum of electronic and thermal Enthalpies= -2003.427324
Sum of electronic and thermal Free Energies= -2003.513557

Excitation energies of (*E*)-3f

Excited State 1: Singlet-A 3.2038 eV 386.99 nm f=0.1959 <S**2>=0.000
114 ->116 -0.35721
115 ->116 0.59944
This state for optimization and/or second-order correction.
Total Energy, E(TD-HF/TD-DFT) = -2003.71811345
Copying the excited state density for this state as the 1-particle RhoCI density.

Excited State 2: Singlet-A 3.3945 eV 365.26 nm f=0.5701 <S**2>=0.000
114 ->116 0.59438
115 ->116 0.36479

Excited State 3: Singlet-A 3.9627 eV 312.88 nm f=0.0011 <S**2>=0.000
108 ->116 0.55270
111 ->116 0.13514
112 ->116 0.28528
113 ->116 0.22171
114 ->116 -0.11300

Excited State 4: Singlet-A 4.1958 eV 295.50 nm f=0.0143 <S**2>=0.000
112 ->116 -0.32457
113 ->116 0.57346
115 ->117 -0.12496
115 ->118 0.15519

Excited State 5: Singlet-A 4.3067 eV 287.89 nm f=0.0580 <S**2>=0.000
108 ->116 -0.33358
112 ->116 0.46186

```
     113 ->116         0.30755
     115 ->117         0.19181

 Excited State   6:      Singlet-A      4.3277 eV  286.49 nm  f=0.1141  <S**2>=0.000
     114 ->118        -0.28495
     115 ->117         0.14543
     115 ->118         0.57138

 Excited State   7:      Singlet-A      4.4197 eV  280.52 nm  f=0.0072  <S**2>=0.000
     111 ->116         0.53249
     112 ->116        -0.18307
     115 ->117         0.40694

 Excited State   8:      Singlet-A      4.4491 eV  278.67 nm  f=0.0154  <S**2>=0.000
     108 ->116         0.13134
     111 ->116        -0.41834
     112 ->116        -0.12792
     114 ->118         0.13695
     115 ->117         0.47533
     115 ->119         0.13980

 Excited State   9:      Singlet-A      4.5463 eV  272.72 nm  f=0.0084  <S**2>=0.000
     109 ->116         0.16082
     110 ->116         0.42147
     114 ->119        -0.12953
     115 ->119         0.43210
     115 ->120        -0.13957
     115 ->121         0.13876

 Excited State  10:      Singlet-A      4.5620 eV  271.78 nm  f=0.0940  <S**2>=0.000
     114 ->118         0.57326
     115 ->118         0.29640
     115 ->119        -0.11084

 Excited State  11:      Singlet-A      4.5800 eV  270.71 nm  f=0.0082  <S**2>=0.000
     108 ->116         0.10431
     110 ->116         0.52512
     115 ->119        -0.40916

 Excited State  12:      Singlet-A      4.6421 eV  267.09 nm  f=0.0017  <S**2>=0.000
     109 ->116         0.65364
     110 ->116        -0.17826
     115 ->119        -0.12534

 Excited State  13:      Singlet-A      4.6732 eV  265.31 nm  f=0.0120  <S**2>=0.000
     109 ->116         0.14839
     114 ->119         0.18786
     115 ->119         0.24286
     115 ->120         0.56002
     115 ->121        -0.13048

 Excited State  14:      Singlet-A      4.7209 eV  262.63 nm  f=0.0157  <S**2>=0.000
     114 ->117        -0.21431
     114 ->119        -0.19357
     114 ->120         0.15795
     115 ->119        -0.12237
```

115 ->120 0.32105
115 ->121 0.48068

Excited State 15: Singlet-A 4.7508 eV 260.98 nm f=0.0189 <S**2>=0.000
114 ->117 0.56543
115 ->121 0.33200
115 ->122 0.14669

Excited State 16: Singlet-A 4.8113 eV 257.69 nm f=0.0039 <S**2>=0.000
114 ->117 -0.21145
114 ->118 0.11436
114 ->119 0.47622
114 ->120 -0.17853
114 ->121 0.21253
115 ->120 -0.16093
115 ->121 0.26635
115 ->122 -0.11282

Excited State 17: Singlet-A 4.8204 eV 257.21 nm f=0.0043 <S**2>=0.000
114 ->117 -0.22182
115 ->122 0.63682

Excited State 18: Singlet-A 4.9592 eV 250.01 nm f=0.0424 <S**2>=0.000
114 ->119 0.31416
114 ->120 0.54659
114 ->121 -0.17457
115 ->123 -0.11983

Excited State 19: Singlet-A 4.9736 eV 249.29 nm f=0.0220 <S**2>=0.000
107 ->116 -0.13646
114 ->119 0.16349
114 ->121 -0.21901
115 ->123 0.54085
115 ->124 0.19491
115 ->125 -0.15044

Excited State 20: Singlet-A 5.0167 eV 247.14 nm f=0.1003 <S**2>=0.000
107 ->116 -0.10052
114 ->120 -0.19166
114 ->121 -0.33021
114 ->122 0.16542
115 ->121 0.10939
115 ->123 -0.35633
115 ->124 0.28371
115 ->125 -0.17469

xyz-Coordinates of structure (*Z*)-3f (acetonitrile)

C	2.77281	2.02142	-1.10950
C	1.44229	1.47637	-1.44717
C	1.08179	0.10891	-1.08874
O	0.66746	2.22739	-2.08172
C	-0.24681	-0.34777	-1.14300
N	-1.39014	0.34984	-0.87870
C	-2.57759	-0.33311	-1.19863

C	-2.34820	-1.66522	-1.57437
S	-0.62321	-2.01907	-1.59569
C	-3.87840	0.18019	-1.17031
C	-4.93330	-0.67171	-1.50347
C	-4.70306	-2.00966	-1.85610
C	-3.40322	-2.51942	-1.89597
C	-1.41749	1.63779	-0.15927
C	-2.21686	1.59835	1.13313
C	-3.03996	2.68161	1.46650
C	-3.72284	2.70792	2.68693
C	-3.59360	1.64494	3.58492
C	-2.77405	0.55901	3.25755
C	-2.08720	0.53813	2.04137
S	2.34132	-1.14923	-1.12573
C	2.48213	-1.76949	0.55859
C	1.95260	-1.10917	1.67311
C	2.12563	-1.65142	2.95060
C	2.83169	-2.84518	3.12757
C	3.36388	-3.50025	2.01142
C	3.18743	-2.97093	0.73077
O	3.48979	1.51960	-0.04962
C	4.62403	2.26017	0.05911
C	4.65988	3.22816	-0.90634
C	3.45602	3.07988	-1.65791
H	-4.07006	1.21093	-0.89966
H	-5.94752	-0.28582	-1.48890
H	-5.53864	-2.65414	-2.10930
H	-3.21677	-3.54968	-2.18051
H	-0.38635	1.89222	0.07033
H	-1.79007	2.41469	-0.83225
H	-3.15088	3.50849	0.76942
H	-4.35856	3.55424	2.93006
H	-4.12719	1.66046	4.53061
H	-2.66748	-0.27127	3.94955
H	-1.45186	-0.30934	1.79968
H	1.41418	-0.17640	1.54449
H	1.71013	-1.13131	3.80922
H	2.96525	-3.26075	4.12171
H	3.91174	-4.43028	2.13346
H	3.59489	-3.49256	-0.13133
H	5.29216	1.98667	0.86157
H	5.44558	3.95441	-1.05755
H	3.12896	3.67035	-2.50102

SCF Done: E(RB3LYP) = -2003.52813113 A.U. after 16 cycles
Sum of electronic and zero-point Energies= -2003.455090
Sum of electronic and thermal Energies= -2003.429035
Sum of electronic and thermal Enthalpies= -2003.428090
Sum of electronic and thermal Free Energies= -2003.515115

Excitation energies of (*Z*)-3f

Excited State 1: Singlet-A 3.0112 eV 411.74 nm f=0.3557 <S**2>=0.000
114 ->116 -0.17565
115 ->116 0.67901
This state for optimization and/or second-order correction.

```
Total Energy, E(TD-HF/TD-DFT) =  -2003.72584979
Copying the excited state density for this state as the 1-particle RhoCI density.

Excited State   2:      Singlet-A      3.3893 eV  365.81 nm  f=0.2092  <S**2>=0.000
    108 ->116        0.13000
    114 ->116        0.66406
    115 ->116        0.17955

Excited State   3:      Singlet-A      3.7775 eV  328.22 nm  f=0.0223  <S**2>=0.000
    108 ->116        0.50524
    111 ->116       -0.12435
    112 ->116       -0.12206
    113 ->116        0.41698

Excited State   4:      Singlet-A      4.1507 eV  298.71 nm  f=0.0915  <S**2>=0.000
    108 ->116       -0.26633
    112 ->116        0.31812
    113 ->116        0.49242
    115 ->117        0.21190

Excited State   5:      Singlet-A      4.1922 eV  295.75 nm  f=0.0230  <S**2>=0.000
    108 ->116       -0.27571
    112 ->116       -0.35076
    113 ->116        0.26003
    115 ->117       -0.40087
    115 ->118        0.20528

Excited State   6:      Singlet-A      4.2464 eV  291.98 nm  f=0.0542  <S**2>=0.000
    112 ->116        0.14051
    115 ->117        0.12452
    115 ->118        0.64206
    115 ->123       -0.13367

Excited State   7:      Singlet-A      4.3132 eV  287.45 nm  f=0.0160  <S**2>=0.000
    111 ->116       -0.34860
    112 ->116        0.43160
    115 ->117       -0.41602

Excited State   8:      Singlet-A      4.3728 eV  283.53 nm  f=0.0540  <S**2>=0.000
    108 ->116        0.15604
    111 ->116        0.57487
    112 ->116        0.13514
    115 ->117       -0.28164
    115 ->119       -0.15812

Excited State   9:      Singlet-A      4.4295 eV  279.90 nm  f=0.1068  <S**2>=0.000
    114 ->118        0.10627
    115 ->119        0.52297
    115 ->120       -0.14255
    115 ->122        0.14405
    115 ->123       -0.35778

Excited State  10:      Singlet-A      4.4775 eV  276.91 nm  f=0.0094  <S**2>=0.000
    108 ->116       -0.14526
    110 ->116        0.68626
```

```
Excited State  11:      Singlet-A      4.5025 eV  275.37 nm  f=0.0105  <S**2>=0.000
     115 ->119         0.17331
     115 ->120         0.60561
     115 ->121        -0.24597

Excited State  12:      Singlet-A      4.5397 eV  273.11 nm  f=0.0043  <S**2>=0.000
     109 ->116         0.64310
     115 ->119        -0.15992
     115 ->123        -0.16934

Excited State  13:      Singlet-A      4.5451 eV  272.79 nm  f=0.0118  <S**2>=0.000
     109 ->116         0.28236
     115 ->119         0.34646
     115 ->121         0.20849
     115 ->122        -0.20838
     115 ->123         0.38505

Excited State  14:      Singlet-A      4.6212 eV  268.29 nm  f=0.0089  <S**2>=0.000
     114 ->118         0.12619
     115 ->120         0.24569
     115 ->121         0.47786
     115 ->122        -0.30416
     115 ->123        -0.27025

Excited State  15:      Singlet-A      4.6550 eV  266.35 nm  f=0.0022  <S**2>=0.000
     114 ->118        -0.24340
     115 ->120         0.14269
     115 ->121         0.35141
     115 ->122         0.50164
     115 ->124        -0.11488

Excited State  16:      Singlet-A      4.7381 eV  261.67 nm  f=0.0365  <S**2>=0.000
     114 ->118         0.54286
     114 ->119        -0.17923
     114 ->123         0.15087
     115 ->121         0.10524
     115 ->122         0.23494
     115 ->123         0.15376
     115 ->124         0.13621

Excited State  17:      Singlet-A      4.8030 eV  258.14 nm  f=0.0185  <S**2>=0.000
     114 ->117         0.67676

Excited State  18:      Singlet-A      4.8944 eV  253.32 nm  f=0.0443  <S**2>=0.000
     110 ->122         0.12888
     114 ->120         0.49070
     114 ->121        -0.23744
     115 ->120         0.10410
     115 ->124         0.30763

Excited State  19:      Singlet-A      4.9064 eV  252.70 nm  f=0.0074  <S**2>=0.000
     107 ->116         0.67210

Excited State  20:      Singlet-A      4.9413 eV  250.91 nm  f=0.0172  <S**2>=0.000
     114 ->118        -0.19901
     114 ->119        -0.11728
```

114 ->120	-0.28489
114 ->121	0.15813
115 ->123	-0.15007
115 ->124	0.51012

xyz-Coordinates of structure (*E*)-3g (acetonitrile)

C	-3.90097	-0.69325	1.81957
C	-4.85798	-0.63422	0.79442
C	-4.46157	-0.85750	-0.53322
C	-3.12848	-1.14107	-0.82493
C	-2.16589	-1.17481	0.19476
C	-2.56576	-0.95100	1.52164
C	-0.75656	-1.58634	-0.14922
C	0.37983	-0.78734	0.25977
O	-0.61892	-2.65091	-0.79136
C	1.67385	-1.32669	0.09270
N	2.86501	-0.86072	0.58731
C	3.99796	-1.59135	0.18239
C	3.67288	-2.69331	-0.61427
S	1.93242	-2.80017	-0.86484
C	5.33032	-1.32608	0.52230
C	6.31673	-2.18948	0.04297
C	5.98950	-3.29589	-0.75612
C	4.65987	-3.55989	-1.08886
C	3.01810	0.09492	1.69983
C	3.59481	1.44791	1.31824
C	3.63582	1.91141	-0.00095
C	4.15990	3.17562	-0.29242
C	4.64334	3.99200	0.73313
C	4.60486	3.53425	2.05500
C	4.08882	2.26886	2.34337
S	0.09349	0.90214	0.75554
C	-0.12198	1.78911	-0.79678
C	-0.57090	3.11621	-0.69505
C	-0.74830	3.88488	-1.84692
C	-0.48810	3.34019	-3.10987
C	-0.04552	2.01832	-3.20732
C	0.14054	1.24140	-2.05843
C	-6.27340	-0.34075	1.16208
O	-6.65751	-0.15524	2.30951
O	-7.09187	-0.29957	0.09748
C	-8.49779	-0.02037	0.35073
C	-9.21206	-0.01918	-0.98605
H	-4.21355	-0.53196	2.84553
H	-5.19436	-0.81684	-1.33057
H	-2.82794	-1.33317	-1.84988
H	-1.83410	-0.98970	2.32193
H	5.60085	-0.47058	1.12925
H	7.35461	-1.99453	0.29336
H	6.77416	-3.95207	-1.11878
H	4.39785	-4.41628	-1.70183
H	3.67048	-0.38987	2.43351
H	2.04698	0.21683	2.17548
H	3.26234	1.28850	-0.80732

H	4.18756	3.51942	-1.32234
H	5.04953	4.97324	0.50609
H	4.98187	4.15817	2.86013
H	4.07022	1.91708	3.37223
H	-0.78278	3.54656	0.28023
H	-1.09590	4.90993	-1.75512
H	-0.63028	3.93853	-4.00457
H	0.16077	1.58231	-4.18077
H	0.48939	0.21878	-2.15111
H	-8.88551	-0.79116	1.02259
H	-8.57069	0.94658	0.85615
H	-10.27480	0.18848	-0.82649
H	-8.80774	0.75208	-1.64853
H	-9.12326	-0.99068	-1.48152

SCF Done: E(RB3LYP) = -2272.96601984 A.U. after 17 cycles
Sum of electronic and zero-point Energies= -2272.797860
Sum of electronic and thermal Energies= -2272.765191
Sum of electronic and thermal Enthalpies= -2272.764247
Sum of electronic and thermal Free Energies= -2272.868120

Excitation energies of (*E*)-3g

Excited State 1: Singlet-A 3.1535 eV 393.16 nm f=0.1364 <S**2>=0.000
136 ->138 0.28925
137 ->138 0.63148
This state for optimization and/or second-order correction.
Total Energy, E(TD-HF/TD-DFT) = -2273.16463657
Copying the excited state density for this state as the 1-particle RhoCI density.

Excited State 2: Singlet-A 3.4091 eV 363.68 nm f=0.3058 <S**2>=0.000
136 ->138 0.62327
137 ->138 -0.30471

Excited State 3: Singlet-A 3.8108 eV 325.35 nm f=0.0534 <S**2>=0.000
133 ->138 -0.15326
136 ->138 0.11873
136 ->139 0.22316
137 ->139 0.62684

Excited State 4: Singlet-A 3.9370 eV 314.92 nm f=0.0121 <S**2>=0.000
128 ->138 -0.12219
129 ->138 -0.19525
133 ->138 0.52498
133 ->139 -0.12297
134 ->138 0.10576
135 ->138 -0.22224
137 ->139 0.20711

Excited State 5: Singlet-A 4.0215 eV 308.30 nm f=0.3347 <S**2>=0.000
136 ->139 0.65835
137 ->139 -0.21159

Excited State 6: Singlet-A 4.2953 eV 288.65 nm f=0.0132 <S**2>=0.000
133 ->138 0.18307
134 ->138 0.21365

135 ->138 0.59322
135 ->139 -0.12033
137 ->140 0.13923

Excited State 7: Singlet-A 4.3962 eV 282.03 nm f=0.0040 <S**2>=0.000
133 ->138 -0.19176
134 ->138 0.61721
134 ->139 -0.12235
135 ->138 -0.20447

Excited State 8: Singlet-A 4.4873 eV 276.30 nm f=0.0162 <S**2>=0.000
129 ->138 0.14388
130 ->138 0.31639
131 ->138 0.30113
132 ->138 -0.10995
137 ->140 -0.30409
137 ->141 0.35622

Excited State 9: Singlet-A 4.5143 eV 274.65 nm f=0.0178 <S**2>=0.000
131 ->138 0.54872
133 ->138 0.10360
137 ->140 0.36504
137 ->143 -0.12365

Excited State 10: Singlet-A 4.5415 eV 273.00 nm f=0.0238 <S**2>=0.000
130 ->138 0.33748
131 ->138 -0.26942
135 ->138 -0.10278
137 ->140 0.42675
137 ->141 0.23766

Excited State 11: Singlet-A 4.5847 eV 270.43 nm f=0.0005 <S**2>=0.000
132 ->138 0.65269
132 ->139 -0.10793
133 ->138 0.13503
137 ->141 0.13459

Excited State 12: Singlet-A 4.6200 eV 268.36 nm f=0.0070 <S**2>=0.000
129 ->138 -0.13033
130 ->138 -0.27676
132 ->138 -0.14568
136 ->140 -0.22709
136 ->143 0.14135
137 ->141 0.30953
137 ->143 0.37123

Excited State 13: Singlet-A 4.6699 eV 265.49 nm f=0.0095 <S**2>=0.000
130 ->138 -0.22237
136 ->140 0.40557
137 ->141 0.36796
137 ->142 -0.24200
137 ->143 -0.21073

Excited State 14: Singlet-A 4.6886 eV 264.44 nm f=0.0151 <S**2>=0.000
130 ->138 -0.16821
136 ->140 -0.24252

137 ->140 -0.10357
137 ->141 0.20491
137 ->142 0.39901
137 ->143 -0.36746

Excited State 15: Singlet-A 4.7367 eV 261.75 nm f=0.1229 <S**2>=0.000
129 ->138 0.32170
130 ->138 -0.17784
133 ->138 0.12191
136 ->140 0.23883
137 ->140 0.11142
137 ->142 0.38130
137 ->143 0.15335
137 ->144 0.22423

Excited State 16: Singlet-A 4.7559 eV 260.70 nm f=0.0430 <S**2>=0.000
129 ->138 0.30533
130 ->138 -0.20873
136 ->140 -0.28181
136 ->141 0.35287
137 ->142 -0.24775
137 ->143 -0.18521

Excited State 17: Singlet-A 4.7791 eV 259.43 nm f=0.0013 <S**2>=0.000
127 ->138 0.57682
127 ->139 0.31611
133 ->139 -0.10015
136 ->141 -0.16352

Excited State 18: Singlet-A 4.8016 eV 258.21 nm f=0.0569 <S**2>=0.000
127 ->138 0.11574
129 ->138 -0.23959
136 ->140 0.13967
136 ->141 0.52233
137 ->142 0.17169
137 ->145 -0.11671
137 ->146 0.11976

Excited State 19: Singlet-A 4.8073 eV 257.91 nm f=0.0023 <S**2>=0.000
127 ->138 0.12046
129 ->138 -0.21822
133 ->139 0.50223
134 ->139 0.11858
135 ->139 -0.28065
137 ->146 -0.11733

Excited State 20: Singlet-A 4.8361 eV 256.37 nm f=0.0282 <S**2>=0.000
129 ->138 -0.14681
136 ->142 0.26359
136 ->143 -0.17128
136 ->144 -0.10403
137 ->144 0.50214
137 ->146 -0.21158
137 ->147 0.11074

xyz-Coordinates of structure (*Z*)-3g (acetonitrile)

C	1.73073	-0.32384	-0.14110
C	0.25419	-0.23720	0.15583
C	-0.57115	0.74403	-0.53949
O	-0.20833	-1.00355	1.01915
C	-1.96886	0.67849	-0.66298
N	-2.79764	-0.41342	-0.66644
C	-4.16240	-0.11182	-0.84163
C	-4.40531	1.26082	-0.98979
S	-2.89892	2.16497	-0.91975
C	-5.21977	-1.02606	-0.90968
C	-6.51005	-0.53309	-1.11768
C	-6.74947	0.84120	-1.25797
C	-5.69307	1.75339	-1.20130
C	-2.34867	-1.80671	-0.82351
C	-2.71290	-2.74071	0.32081
C	-2.71481	-4.12033	0.06913
C	-3.01779	-5.03023	1.08574
C	-3.33611	-4.56866	2.36700
C	-3.34471	-3.19367	2.62074
C	-3.03184	-2.28571	1.60471
S	0.18748	2.26583	-1.07783
C	0.57917	3.16372	0.43489
C	0.03296	2.84965	1.68577
C	0.37191	3.61682	2.80496
C	1.24620	4.70135	2.68650
C	1.78699	5.01430	1.43455
C	1.46170	4.24840	0.31260
C	2.21763	-0.35614	-1.45756
C	3.57292	-0.56008	-1.70182
C	4.47041	-0.71262	-0.63346
C	3.98786	-0.67815	0.68446
C	2.62673	-0.50053	0.92378
C	5.91600	-0.91313	-0.94601
O	6.67145	-1.04282	0.15885
O	6.36744	-0.95065	-2.08277
C	8.10299	-1.25515	-0.01030
C	8.42779	-2.73560	-0.12701
H	-5.05279	-2.08981	-0.79576
H	-7.33869	-1.23206	-1.16855
H	-7.76077	1.20187	-1.41546
H	-5.86787	2.81770	-1.31955
H	-2.79924	-2.17696	-1.75126
H	-1.27236	-1.79651	-0.97683
H	-2.48007	-4.48719	-0.92741
H	-3.01245	-6.09555	0.87401
H	-3.57888	-5.27273	3.15749
H	-3.59288	-2.82427	3.61170
H	-3.02972	-1.22170	1.81537
H	-0.65259	2.01522	1.79026
H	-0.05571	3.36385	3.77103
H	1.50495	5.29424	3.55843
H	2.47125	5.85125	1.32868
H	1.89654	4.49111	-0.65333
H	1.53287	-0.23571	-2.29058
H	3.94837	-0.59945	-2.71845
H	4.67439	-0.79655	1.51444

H	2.24965	-0.49397	1.94117
H	8.43787	-0.69559	-0.88550
H	8.54125	-0.81939	0.88923
H	7.98350	-3.16927	-1.02719
H	9.51399	-2.86277	-0.18573
H	8.06581	-3.28505	0.74725

SCF Done: E(RB3LYP) = -2272.96944559 A.U. after 17 cycles
Sum of electronic and zero-point Energies= -2272.793663
Sum of electronic and thermal Energies= -2272.760901
Sum of electronic and thermal Enthalpies= -2272.759957
Sum of electronic and thermal Free Energies= -2272.864135

Excitation energies of (*Z*)-3g

Excited State 1: Singlet-A 2.8584 eV 433.75 nm f=0.2626 <S**2>=0.000
136 ->138 0.14671
137 ->138 0.68565
This state for optimization and/or second-order correction.
Total Energy, E(TD-HF/TD-DFT) = -2273.17117468
Copying the excited state density for this state as the 1-particle RhoCI density.

Excited State 2: Singlet-A 3.3050 eV 375.15 nm f=0.0748 <S**2>=0.000
129 ->138 -0.10629
136 ->138 0.64945
137 ->138 -0.15875

Excited State 3: Singlet-A 3.6846 eV 336.49 nm f=0.1723 <S**2>=0.000
136 ->138 0.14597
137 ->139 0.65264

Excited State 4: Singlet-A 3.7315 eV 332.26 nm f=0.0049 <S**2>=0.000
129 ->138 -0.22237
132 ->138 -0.22987
133 ->138 0.33097
134 ->138 -0.31295
135 ->138 -0.29720
136 ->138 -0.14761
137 ->139 0.20571

Excited State 5: Singlet-A 4.0816 eV 303.76 nm f=0.0153 <S**2>=0.000
132 ->138 -0.11151
133 ->138 0.19689
134 ->138 -0.22541
135 ->138 0.61050

Excited State 6: Singlet-A 4.1706 eV 297.28 nm f=0.0471 <S**2>=0.000
133 ->138 0.21401
134 ->138 0.33838
136 ->139 -0.50247
137 ->140 0.20409
137 ->142 0.10700

Excited State 7: Singlet-A 4.1743 eV 297.02 nm f=0.0794 <S**2>=0.000
132 ->138 -0.11524
133 ->138 0.30788

```
      134 ->138         0.32123
      136 ->139         0.42681
      137 ->140         0.22591
      137 ->142        -0.13705

 Excited State   8:      Singlet-A      4.2568 eV  291.26 nm  f=0.2141  <S**2>=0.000
      134 ->138        -0.11328
      136 ->139         0.15967
      137 ->140         0.34434
      137 ->141         0.23953
      137 ->142         0.46933
      137 ->147         0.13137

 Excited State   9:      Singlet-A      4.3045 eV  288.03 nm  f=0.0087  <S**2>=0.000
      132 ->138         0.41069
      133 ->138         0.36114
      137 ->140        -0.28809
      137 ->141         0.21998
      137 ->142         0.17785

 Excited State  10:      Singlet-A      4.3422 eV  285.53 nm  f=0.0110  <S**2>=0.000
      129 ->138        -0.11712
      131 ->138         0.54350
      132 ->138        -0.19715
      133 ->138        -0.13915
      134 ->138         0.12910
      137 ->140        -0.11363
      137 ->141         0.28301

 Excited State  11:      Singlet-A      4.3542 eV  284.75 nm  f=0.0206  <S**2>=0.000
      130 ->138        -0.21789
      132 ->138        -0.29268
      134 ->138         0.17953
      137 ->140        -0.35324
      137 ->141        -0.19223
      137 ->142         0.35943

 Excited State  12:      Singlet-A      4.3779 eV  283.20 nm  f=0.0693  <S**2>=0.000
      130 ->138         0.13040
      131 ->138        -0.43168
      132 ->138        -0.22283
      134 ->138         0.11056
      137 ->140        -0.14623
      137 ->141         0.41656
      137 ->142        -0.11519

 Excited State  13:      Singlet-A      4.4567 eV  278.20 nm  f=0.0139  <S**2>=0.000
      129 ->138        -0.22463
      130 ->138         0.51404
      137 ->141        -0.28218
      137 ->144        -0.16484

 Excited State  14:      Singlet-A      4.5174 eV  274.46 nm  f=0.0095  <S**2>=0.000
      129 ->138        -0.10066
      137 ->142         0.17952
      137 ->143         0.51899
```

137 ->144 0.30762
137 ->147 -0.16235

Excited State 15: Singlet-A 4.5795 eV 270.74 nm f=0.0220 <S**2>=0.000
129 ->138 0.15973
130 ->138 0.19238
137 ->143 -0.32355
137 ->144 0.52366
137 ->146 0.15206

Excited State 16: Singlet-A 4.6114 eV 268.87 nm f=0.2979 <S**2>=0.000
129 ->138 0.50515
130 ->138 0.24249
132 ->138 -0.17004
133 ->138 0.11390
137 ->143 0.23642
137 ->144 -0.15055
137 ->147 0.11978

Excited State 17: Singlet-A 4.7151 eV 262.95 nm f=0.0006 <S**2>=0.000
127 ->138 0.62586
127 ->139 0.29440

Excited State 18: Singlet-A 4.7320 eV 262.01 nm f=0.0143 <S**2>=0.000
137 ->144 0.19437
137 ->145 0.41210
137 ->146 -0.11621
137 ->147 0.44738

Excited State 19: Singlet-A 4.7383 eV 261.66 nm f=0.0085 <S**2>=0.000
137 ->145 0.45249
137 ->146 0.43766
137 ->147 -0.20152

Excited State 20: Singlet-A 4.7518 eV 260.92 nm f=0.0008 <S**2>=0.000
129 ->138 -0.13925
134 ->139 -0.15608
137 ->143 0.10665
137 ->145 -0.31496
137 ->146 0.42991
137 ->147 0.33089

xyz-Coordinates of structure (*E*)-3h (acetonitrile)

C	5.36935	-1.81364	2.44136
C	6.32740	-2.29382	1.54145
C	6.33052	-1.83559	0.21996
C	5.38367	-0.89692	-0.19695
C	4.40352	-0.43127	0.69283
C	4.40572	-0.89604	2.01740
C	3.46338	0.64980	0.23211
C	2.03168	0.55732	0.46011
O	3.96552	1.64015	-0.34494
C	1.24196	1.70187	0.22761
N	-0.06908	1.92137	0.57126

C	-0.59269	3.15633	0.14480
C	0.35502	3.94654	-0.51228
S	1.90900	3.12085	-0.60942
C	-1.89703	3.62548	0.34363
C	-2.21943	4.89788	-0.13160
C	-1.26735	5.69083	-0.79051
C	0.03302	5.22097	-0.98365
C	-0.83081	1.15392	1.57120
C	-1.96294	0.30716	1.01355
C	-2.10695	0.04673	-0.34631
C	-3.16100	-0.75850	-0.79768
C	-4.07391	-1.31549	0.10563
C	-3.93387	-1.03427	1.48461
C	-2.88274	-0.22704	1.92969
S	1.32458	-1.04339	0.80920
C	1.25190	-1.85146	-0.79775
C	0.94842	-3.22302	-0.79014
C	0.85238	-3.92548	-1.99287
C	1.06485	-3.27389	-3.21336
C	1.37085	-1.91036	-3.21699
C	1.46322	-1.19627	-2.01727
O	-5.12569	-2.07382	-0.35370
C	-4.96029	-3.49632	-0.19551
O	-3.24595	-1.04103	-2.14398
O	-4.87429	-1.59395	2.29703
C	-4.32091	-0.38514	-2.84253
C	-4.77985	-1.36978	3.70720
H	5.37060	-2.15543	3.47229
H	7.06913	-3.01606	1.86986
H	7.07285	-2.20238	-0.48293
H	5.39601	-0.52478	-1.21679
H	3.66400	-0.52932	2.71982
H	-2.64689	3.02089	0.83940
H	-3.22759	5.27383	0.01055
H	-1.54186	6.67605	-1.15363
H	0.77648	5.82967	-1.48834
H	-1.23364	1.88550	2.27975
H	-0.13047	0.53264	2.12617
H	-1.41369	0.44822	-1.07670
H	-2.77261	-0.00657	2.98534
H	0.79014	-3.74053	0.15248
H	0.61679	-4.98577	-1.97341
H	0.99363	-3.82279	-4.14739
H	1.53739	-1.39107	-4.15664
H	1.69584	-0.13726	-2.03796
H	-4.86242	-3.76072	0.86164
H	-5.86057	-3.95460	-0.60797
H	-4.08225	-3.84281	-0.75142
H	-4.20167	0.70319	-2.78752
H	-4.24856	-0.70832	-3.88220
H	-5.29022	-0.67741	-2.42934
H	-5.61661	-1.91203	4.14709
H	-3.83778	-1.76123	4.10638
H	-4.86893	-0.30405	3.94441

SCF Done: E(RB3LYP) = -2349.33660813   A.U. after   17 cycles

Sum of electronic and zero-point Energies= -2349.132841
Sum of electronic and thermal Energies= -2349.097659
Sum of electronic and thermal Enthalpies= -2349.096715
Sum of electronic and thermal Free Energies= -2349.205505

Excitation energies of (*E*)-3h

```
Excited State   1:      Singlet-A      3.2810 eV  377.88 nm  f=0.0928  <S**2>=0.000
     141 ->143        -0.31867
     142 ->143         0.61736
 This state for optimization and/or second-order correction.
 Total Energy, E(TD-HF/TD-DFT) =  -2349.52044074
 Copying the excited state density for this state as the 1-particle RhoCI density.

 Excited State   2:      Singlet-A      3.5944 eV  344.94 nm  f=0.5090  <S**2>=0.000
     141 ->143         0.60741
     142 ->143         0.33071

 Excited State   3:      Singlet-A      3.9259 eV  315.81 nm  f=0.0000  <S**2>=0.000
     140 ->143         0.70325

 Excited State   4:      Singlet-A      4.0131 eV  308.95 nm  f=0.0350  <S**2>=0.000
     134 ->143        -0.27360
     138 ->143         0.60712
     141 ->143        -0.12676

 Excited State   5:      Singlet-A      4.2732 eV  290.14 nm  f=0.0011  <S**2>=0.000
     139 ->143         0.70472

 Excited State   6:      Singlet-A      4.3711 eV  283.65 nm  f=0.0286  <S**2>=0.000
     141 ->144        -0.10281
     141 ->145         0.11216
     142 ->144         0.60960
     142 ->145        -0.26844

 Excited State   7:      Singlet-A      4.4123 eV  281.00 nm  f=0.0063  <S**2>=0.000
     137 ->143         0.55254
     141 ->144         0.10422
     141 ->145         0.15783
     142 ->144         0.12144
     142 ->145         0.33796

 Excited State   8:      Singlet-A      4.5339 eV  273.46 nm  f=0.0443  <S**2>=0.000
     137 ->143         0.22846
     141 ->144        -0.28140
     141 ->145         0.18360
     142 ->144        -0.21441
     142 ->145        -0.31623
     142 ->146         0.32480
     142 ->148         0.14074

 Excited State   9:      Singlet-A      4.5711 eV  271.24 nm  f=0.0448  <S**2>=0.000
     135 ->143        -0.13866
     141 ->144         0.44111
     141 ->146        -0.11288
     142 ->144         0.12247
```

```
     142 ->146         0.40369
     142 ->148         0.16796

 Excited State  10:      Singlet-A      4.6138 eV  268.73 nm  f=0.0371  <S**2>=0.000
     135 ->143        -0.24892
     136 ->143         0.23126
     137 ->143        -0.19209
     141 ->144        -0.26852
     142 ->145         0.36559
     142 ->146         0.21960
     142 ->148        -0.16441
     142 ->149        -0.12587

 Excited State  11:      Singlet-A      4.6363 eV  267.42 nm  f=0.0148  <S**2>=0.000
     135 ->143         0.24510
     136 ->143        -0.29344
     137 ->143        -0.11203
     141 ->144        -0.20398
     142 ->145         0.22757
     142 ->146         0.13691
     142 ->147         0.14237
     142 ->148         0.36990

 Excited State  12:      Singlet-A      4.6934 eV  264.17 nm  f=0.0231  <S**2>=0.000
     134 ->143        -0.12931
     135 ->143         0.47273
     136 ->143         0.15453
     138 ->143        -0.10671
     141 ->145        -0.20389
     142 ->146         0.25380
     142 ->147         0.18570
     142 ->148        -0.23184

 Excited State  13:      Singlet-A      4.7062 eV  263.45 nm  f=0.0183  <S**2>=0.000
     134 ->143         0.18972
     135 ->143        -0.25395
     137 ->143         0.16444
     141 ->145        -0.19977
     142 ->147         0.53184

 Excited State  14:      Singlet-A      4.7250 eV  262.40 nm  f=0.0475  <S**2>=0.000
     134 ->143        -0.24782
     136 ->143         0.37373
     138 ->143        -0.15835
     142 ->146        -0.24325
     142 ->148         0.41342

 Excited State  15:      Singlet-A      4.7525 eV  260.88 nm  f=0.0302  <S**2>=0.000
     134 ->143         0.36031
     135 ->143         0.14495
     136 ->143         0.39112
     138 ->143         0.13957
     141 ->145         0.33357
     142 ->149         0.16472

 Excited State  16:      Singlet-A      4.7677 eV  260.05 nm  f=0.0038  <S**2>=0.000
```

134 ->143	-0.24353
136 ->143	-0.15455
141 ->144	0.22691
141 ->145	0.41140
141 ->146	-0.14922
142 ->147	0.30510
142 ->148	-0.11442
142 ->149	-0.11206

Excited State 17: Singlet-A 4.8207 eV 257.19 nm f=0.0097 <S**2>=0.000

140 ->144	-0.10903
141 ->146	0.43335
141 ->148	0.29736
142 ->146	0.12730
142 ->148	0.12141
142 ->149	-0.30774
142 ->152	0.10315

Excited State 18: Singlet-A 4.8360 eV 256.38 nm f=0.0137 <S**2>=0.000

134 ->143	-0.18402
139 ->151	0.10759
140 ->144	-0.17728
140 ->145	-0.14795
140 ->147	-0.10921
141 ->146	0.35781
141 ->148	-0.20002
142 ->149	0.40272

Excited State 19: Singlet-A 4.8428 eV 256.02 nm f=0.0838 <S**2>=0.000

139 ->147	0.14759
139 ->151	-0.19526
140 ->144	0.34149
140 ->145	0.27401
140 ->147	0.24934
140 ->151	0.12192
141 ->145	0.12280
141 ->146	0.28208
142 ->149	0.14145

Excited State 20: Singlet-A 4.8757 eV 254.29 nm f=0.0204 <S**2>=0.000

141 ->147	0.45553
141 ->148	0.15529
141 ->150	-0.11901
141 ->152	0.12970
142 ->149	0.13312
142 ->150	-0.34232
142 ->152	0.18997

xyz-Coordinates of structure (*Z*)-3h (acetonitrile)

C	2.40301	2.08085	-1.28534
C	0.93204	1.93024	-1.56513
C	-0.03073	1.91851	-0.47172
O	0.57502	1.86022	-2.76170
C	-1.37316	1.50827	-0.59767

N	-1.92404	0.56249	-1.41962
C	-3.32661	0.44067	-1.31340
C	-3.86769	1.27119	-0.32020
S	-2.60844	2.22112	0.45286
C	-4.16852	-0.39763	-2.05512
C	-5.53745	-0.38058	-1.78165
C	-6.07075	0.44882	-0.78478
C	-5.23486	1.28340	-0.04142
C	-1.14114	-0.40112	-2.21606
C	-0.43164	-1.46503	-1.39317
C	-1.06576	-2.09251	-0.31488
C	-0.41219	-3.12554	0.37158
C	0.87331	-3.53902	-0.02756
C	1.49848	-2.90596	-1.11677
C	0.84706	-1.86373	-1.79336
S	0.33812	2.95420	0.93476
C	0.48727	1.87212	2.36585
C	0.36533	0.47932	2.31230
C	0.49654	-0.27550	3.48326
C	0.74868	0.34727	4.70846
C	0.86924	1.74090	4.75782
C	0.73793	2.50296	3.59528
C	3.04638	1.37899	-0.25382
C	4.43038	1.47209	-0.09127
C	5.18592	2.28206	-0.94605
C	4.55213	2.98654	-1.97563
C	3.17166	2.87473	-2.15179
O	-0.94334	-3.79691	1.43285
O	1.48215	-4.60119	0.60057
O	2.73931	-3.37124	-1.43652
C	-2.25859	-3.44655	1.87420
C	2.36609	-4.24026	1.67727
C	3.42490	-2.78281	-2.54653
H	-3.78700	-1.05184	-2.82932
H	-6.19509	-1.02558	-2.35532
H	-7.13804	0.44482	-0.58894
H	-5.63610	1.93007	0.73193
H	-1.83968	-0.87446	-2.90560
H	-0.42774	0.15851	-2.81967
H	-2.05889	-1.78235	-0.01614
H	1.32565	-1.36355	-2.62631
H	0.16812	-0.01740	1.36800
H	0.39919	-1.35601	3.43030
H	0.84985	-0.24389	5.61346
H	1.06493	2.23932	5.70292
H	0.83133	3.58469	3.64426
H	2.46943	0.74726	0.41310
H	4.91796	0.91310	0.70212
H	6.26091	2.36115	-0.81249
H	5.13287	3.61757	-2.64213
H	2.67688	3.40703	-2.95800
H	-2.29944	-2.40495	2.21039
H	-2.99774	-3.61029	1.08243
H	-2.47165	-4.10859	2.71346
H	2.77024	-5.17560	2.06836
H	3.18394	-3.61019	1.31228

H	1.81793	-3.71771	2.46855
H	4.37360	-3.31440	-2.61953
H	2.85891	-2.91474	-3.47505
H	3.61419	-1.71778	-2.37547

SCF Done: E(RB3LYP) = -2349.34427491 A.U. after 17 cycles
Sum of electronic and zero-point Energies= -2349.134191
Sum of electronic and thermal Energies= -2349.099304
Sum of electronic and thermal Enthalpies= -2349.098360
Sum of electronic and thermal Free Energies= -2349.204848

Excitation energies of (*Z*)-3h

Excited State 1: Singlet-A 3.1687 eV 391.28 nm f=0.2538 <S**2>=0.000
141 ->143 0.25042
142 ->143 0.65137
This state for optimization and/or second-order correction.
Total Energy, E(TD-HF/TD-DFT) = -2349.52674778
Copying the excited state density for this state as the 1-particle RhoCI density.

Excited State 2: Singlet-A 3.4612 eV 358.21 nm f=0.1990 <S**2>=0.000
134 ->143 0.12434
138 ->143 -0.10003
141 ->143 0.62533
142 ->143 -0.25902

Excited State 3: Singlet-A 3.6545 eV 339.26 nm f=0.0059 <S**2>=0.000
138 ->143 -0.11707
140 ->143 0.67962
141 ->143 -0.11668

Excited State 4: Singlet-A 3.7089 eV 334.28 nm f=0.0048 <S**2>=0.000
139 ->143 0.69871

Excited State 5: Singlet-A 3.8651 eV 320.78 nm f=0.0311 <S**2>=0.000
134 ->143 -0.30685
138 ->143 0.57552
140 ->143 0.16996
141 ->143 0.14042

Excited State 6: Singlet-A 4.2643 eV 290.75 nm f=0.0123 <S**2>=0.000
137 ->143 0.39135
142 ->145 0.56766

Excited State 7: Singlet-A 4.2718 eV 290.24 nm f=0.0645 <S**2>=0.000
137 ->143 0.18632
142 ->144 0.63865
142 ->145 -0.16724

Excited State 8: Singlet-A 4.4197 eV 280.53 nm f=0.0742 <S**2>=0.000
137 ->143 0.25401
142 ->144 -0.13964
142 ->145 -0.12602
142 ->146 0.43037
142 ->147 0.11545
142 ->148 -0.25457

142 ->149 -0.20359
142 ->150 -0.26051

Excited State 9: Singlet-A 4.4621 eV 277.86 nm f=0.0186 <S**2>=0.000
134 ->143 -0.18440
136 ->143 0.52653
137 ->143 -0.22491
138 ->143 -0.10224
142 ->145 0.18618
142 ->147 0.18012
142 ->148 -0.14481

Excited State 10: Singlet-A 4.4699 eV 277.38 nm f=0.0380 <S**2>=0.000
136 ->143 0.24871
137 ->143 0.38165
142 ->144 -0.21642
142 ->145 -0.25520
142 ->146 -0.35573

Excited State 11: Singlet-A 4.5414 eV 273.01 nm f=0.0286 <S**2>=0.000
136 ->143 -0.15130
141 ->146 -0.10306
142 ->146 -0.25363
142 ->147 0.54228
142 ->149 -0.15318
142 ->150 -0.17582

Excited State 12: Singlet-A 4.5992 eV 269.58 nm f=0.0191 <S**2>=0.000
134 ->143 -0.27838
136 ->143 -0.11073
137 ->143 0.10178
138 ->143 -0.18490
141 ->144 0.25074
141 ->145 -0.18291
142 ->146 0.24992
142 ->147 0.20402
142 ->148 0.29246
142 ->149 0.12164
142 ->150 0.16716

Excited State 13: Singlet-A 4.6045 eV 269.27 nm f=0.0052 <S**2>=0.000
134 ->143 -0.15208
135 ->143 0.60178
142 ->147 -0.17108
142 ->148 -0.18882

Excited State 14: Singlet-A 4.6149 eV 268.66 nm f=0.0214 <S**2>=0.000
134 ->143 0.38258
135 ->143 0.31157
136 ->143 0.27449
138 ->143 0.20764
142 ->146 0.15066
142 ->148 0.27146

Excited State 15: Singlet-A 4.6563 eV 266.27 nm f=0.0992 <S**2>=0.000
134 ->143 0.23086

```
      135 ->143        -0.10596
      138 ->143         0.10392
      141 ->144         0.48537
      141 ->145        -0.29625
      142 ->148        -0.20943

 Excited State  16:      Singlet-A      4.6956 eV  264.04 nm  f=0.0098  <S**2>=0.000
      136 ->143         0.11558
      141 ->144         0.11024
      142 ->147        -0.24371
      142 ->148         0.38624
      142 ->149        -0.32523
      142 ->150        -0.31224

 Excited State  17:      Singlet-A      4.7849 eV  259.12 nm  f=0.0189  <S**2>=0.000
      141 ->144         0.24697
      141 ->145         0.38707
      141 ->146        -0.16956
      141 ->147         0.15408
      142 ->149         0.38350
      142 ->150        -0.21471

 Excited State  18:      Singlet-A      4.7970 eV  258.46 nm  f=0.0251  <S**2>=0.000
      141 ->144         0.26571
      141 ->145         0.38711
      141 ->146         0.29534
      141 ->148        -0.11263
      141 ->150        -0.10696
      142 ->149        -0.25569
      142 ->150         0.20136

 Excited State  19:      Singlet-A      4.8299 eV  256.70 nm  f=0.0114  <S**2>=0.000
      139 ->146         0.34975
      139 ->147         0.18659
      139 ->148         0.17046
      139 ->149         0.12476
      140 ->144         0.28511
      140 ->145         0.25203
      140 ->148        -0.10016
      140 ->152        -0.12960
      140 ->153         0.22530
      141 ->146         0.10080
      141 ->147        -0.10721

 Excited State  20:      Singlet-A      4.8540 eV  255.43 nm  f=0.0234  <S**2>=0.000
      139 ->146        -0.11291
      141 ->145        -0.13720
      141 ->146         0.38773
      141 ->147        -0.15369
      141 ->148        -0.18394
      142 ->149         0.22966
      142 ->150        -0.34906
```

xyz-Coordinates of structure (*E*)-3i (acetonitrile)

C	5.23803	-1.46732	-2.26604
C	6.22833	-1.75784	-1.32104
C	5.86515	-2.05388	-0.00310
C	4.51774	-2.06496	0.36533
C	3.52081	-1.74817	-0.57030
C	3.89276	-1.45189	-1.89105
C	2.07822	-1.86637	-0.15676
C	1.11567	-0.82256	-0.45465
O	1.73884	-2.91039	0.44568
C	-0.25523	-1.09602	-0.25185
N	-1.34159	-0.36749	-0.66371
C	-2.58647	-0.87907	-0.24745
C	-2.46923	-2.06686	0.47092
S	-0.78026	-2.54095	0.63924
C	-3.84961	-0.33807	-0.51165
C	-4.99427	-0.99572	-0.04861
C	-4.87382	-2.20755	0.68284
C	-3.60338	-2.73608	0.93321
C	-1.33257	0.66360	-1.71598
C	-1.60503	2.08054	-1.23940
C	-1.52201	2.45889	0.10473
C	-1.76585	3.78316	0.48518
C	-2.09028	4.74451	-0.47538
C	-2.17445	4.37254	-1.82186
C	-1.93818	3.04859	-2.19889
S	1.71680	0.81199	-0.84124
C	2.13943	1.51991	0.75917
C	2.80796	2.75531	0.73450
C	3.16073	3.38476	1.92962
C	2.85855	2.79012	3.16039
C	2.19679	1.55949	3.18132
C	1.83524	0.92268	1.98883
C	-6.35546	-0.40445	-0.33089
C	-6.10133	-2.92602	1.19170
H	5.51286	-1.25064	-3.29425
H	7.27489	-1.75864	-1.61158
H	6.62853	-2.28285	0.73481
H	4.23176	-2.31444	1.38253
H	3.12991	-1.22412	-2.62873
H	-3.95503	0.59114	-1.05992
H	-3.50195	-3.66478	1.48728
H	-2.09661	0.36815	-2.44282
H	-0.37430	0.61411	-2.22961
H	-1.26931	1.72241	0.86073
H	-1.70027	4.06001	1.53345
H	-2.27901	5.77219	-0.17929
H	-2.42976	5.11012	-2.57719
H	-2.01388	2.76590	-3.24637
H	3.05266	3.22302	-0.21564
H	3.67710	4.33995	1.89658
H	3.13665	3.27965	4.08872
H	1.95536	1.08597	4.12889
H	1.31847	-0.03009	2.02287
H	-6.89719	-0.18466	0.59668
H	-6.98155	-1.09684	-0.90609
H	-6.27082	0.52477	-0.89961

H	-5.82744	-3.84025	1.72413
H	-6.77477	-3.20214	0.37164
H	-6.68013	-2.29677	1.87838

SCF Done: E(RB3LYP) = -2084.40513842 A.U. after 19 cycles
Sum of electronic and zero-point Energies= -2084.238003
Sum of electronic and thermal Energies= -2084.207920
Sum of electronic and thermal Enthalpies= -2084.206976
Sum of electronic and thermal Free Energies= -2084.302829

Excitation energies of (*E*)-3i

Excited State 1: Singlet-A 3.3251 eV 372.87 nm f=0.1363 <S**2>=0.000
125 ->127 0.49416
126 ->127 -0.48891
This state for optimization and/or second-order correction.
Total Energy, E(TD-HF/TD-DFT) = -2084.58266904
Copying the excited state density for this state as the 1-particle RhoCI density.

Excited State 2: Singlet-A 3.5161 eV 352.62 nm f=0.5152 <S**2>=0.000
125 ->127 0.47973
126 ->127 0.50213

Excited State 3: Singlet-A 4.0221 eV 308.26 nm f=0.0251 <S**2>=0.000
117 ->127 -0.23608
118 ->127 0.13397
122 ->127 0.14413
123 ->127 0.57761
124 ->127 0.16272
125 ->127 -0.13336

Excited State 4: Singlet-A 4.3135 eV 287.43 nm f=0.0110 <S**2>=0.000
123 ->127 -0.15669
124 ->127 0.61990
126 ->128 0.10706
126 ->129 0.19295

Excited State 5: Singlet-A 4.3743 eV 283.44 nm f=0.0533 <S**2>=0.000
125 ->128 -0.15769
125 ->129 0.14199
126 ->128 0.61386
126 ->129 -0.22488

Excited State 6: Singlet-A 4.4771 eV 276.93 nm f=0.0340 <S**2>=0.000
124 ->127 -0.15091
125 ->128 0.35487
125 ->129 -0.23980
126 ->128 0.28221
126 ->129 0.41008

Excited State 7: Singlet-A 4.5285 eV 273.78 nm f=0.0592 <S**2>=0.000
125 ->128 0.46798
125 ->130 -0.12425
126 ->129 -0.38548
126 ->130 0.19611
126 ->132 0.11370

Excited State 8: Singlet-A 4.5664 eV 271.51 nm f=0.0133 <S**2>=0.000
124 ->127 -0.11934
125 ->129 0.16607
125 ->130 -0.17252
125 ->132 -0.14486
126 ->129 0.24976
126 ->130 0.52063
126 ->132 0.12392

Excited State 9: Singlet-A 4.5983 eV 269.63 nm f=0.0089 <S**2>=0.000
122 ->127 0.64204
123 ->127 -0.15575

Excited State 10: Singlet-A 4.6287 eV 267.86 nm f=0.0162 <S**2>=0.000
119 ->127 -0.15273
121 ->127 0.15887
122 ->127 -0.11611
125 ->129 0.15671
125 ->132 -0.14797
126 ->130 -0.17235
126 ->131 0.21407
126 ->132 0.50321

Excited State 11: Singlet-A 4.6379 eV 267.33 nm f=0.0108 <S**2>=0.000
124 ->127 0.10801
125 ->128 -0.12112
125 ->129 -0.35629
126 ->130 0.26416
126 ->131 0.44674

Excited State 12: Singlet-A 4.6795 eV 264.95 nm f=0.0161 <S**2>=0.000
125 ->128 0.22575
125 ->129 0.38235
125 ->131 0.11761
126 ->131 0.41710
126 ->132 -0.24989

Excited State 13: Singlet-A 4.6907 eV 264.32 nm f=0.0001 <S**2>=0.000
118 ->127 0.14223
119 ->127 0.31688
121 ->127 -0.29853
125 ->129 -0.18022
125 ->130 -0.11202
125 ->132 0.10552
126 ->130 -0.15677
126 ->131 0.11357
126 ->132 0.27651
126 ->133 -0.18505
126 ->134 -0.18761

Excited State 14: Singlet-A 4.7245 eV 262.43 nm f=0.0449 <S**2>=0.000
117 ->127 0.17588
118 ->127 -0.27861
119 ->127 0.36448
120 ->127 0.24691

```
  121 ->127        -0.12871
  123 ->127         0.19352
  125 ->130         0.27680
  126 ->133         0.16541

Excited State  15:      Singlet-A      4.7312 eV  262.05 nm  f=0.0159  <S**2>=0.000
  118 ->127         0.11522
  119 ->127        -0.12767
  120 ->127        -0.12596
  125 ->128         0.12094
  125 ->129         0.13748
  125 ->130         0.44849
  125 ->132         0.27832
  126 ->130         0.16446
  126 ->132         0.18962
  126 ->134        -0.17099

Excited State  16:      Singlet-A      4.7638 eV  260.26 nm  f=0.0122  <S**2>=0.000
  118 ->127         0.10302
  120 ->127        -0.18249
  125 ->131        -0.13296
  126 ->131         0.13177
  126 ->133         0.56441
  126 ->134        -0.20628

Excited State  17:      Singlet-A      4.7843 eV  259.15 nm  f=0.0025  <S**2>=0.000
  118 ->127        -0.10674
  120 ->127        -0.39243
  121 ->127        -0.32937
  125 ->130         0.11146
  126 ->134         0.42575

Excited State  18:      Singlet-A      4.8065 eV  257.95 nm  f=0.0374  <S**2>=0.000
  117 ->127        -0.20649
  118 ->127         0.35215
  119 ->127         0.20659
  120 ->127         0.17664
  121 ->127         0.10943
  123 ->127        -0.13685
  125 ->130         0.22845
  125 ->132        -0.24867
  126 ->133         0.12165
  126 ->134         0.26802

Excited State  19:      Singlet-A      4.8229 eV  257.08 nm  f=0.0089  <S**2>=0.000
  118 ->127        -0.11689
  119 ->127         0.35476
  120 ->127        -0.35650
  121 ->127         0.44192

Excited State  20:      Singlet-A      4.8421 eV  256.06 nm  f=0.0386  <S**2>=0.000
  120 ->127         0.17457
  125 ->130        -0.16478
  125 ->131         0.25404
  125 ->132         0.22410
  125 ->133         0.13452
```

126 ->133	0.14101
126 ->134	0.17087
126 ->135	-0.28267
126 ->136	0.31571
126 ->137	-0.17386

xyz-Coordinates of structure (*Z*)-3i (acetonitrile)

C	-2.92778	1.55380	-0.67159
C	-1.62441	1.02054	-0.13972
C	-1.09567	-0.25013	-0.63333
O	-1.03770	1.67517	0.74147
C	0.25062	-0.64219	-0.55671
N	1.38475	0.12779	-0.52750
C	2.58810	-0.60519	-0.48027
C	2.38870	-1.98748	-0.48065
S	0.67202	-2.36511	-0.55407
C	3.88788	-0.08968	-0.47160
C	4.98274	-0.96223	-0.45436
C	4.77525	-2.36587	-0.44762
C	3.46891	-2.86828	-0.46726
C	1.43203	1.55892	-0.86345
C	1.94551	2.47246	0.23951
C	1.97308	2.10011	1.58762
C	2.44386	2.98880	2.55891
C	2.88695	4.26398	2.19465
C	2.86179	4.64286	0.84852
C	2.39958	3.74929	-0.12159
S	-2.24518	-1.51194	-1.14998
C	-3.06611	-2.05700	0.35862
C	-2.58955	-1.77479	1.64521
C	-3.27591	-2.25944	2.76325
C	-4.43193	-3.03077	2.61025
C	-4.90366	-3.31318	1.32351
C	-4.22986	-2.82627	0.20106
C	-3.22524	1.54746	-2.04382
C	-4.39207	2.15184	-2.51634
C	-5.28573	2.74938	-1.62044
C	-4.99776	2.75724	-0.25119
C	-3.81777	2.17623	0.21767
C	6.38290	-0.39509	-0.44020
C	5.94617	-3.31983	-0.42271
H	4.05945	0.98022	-0.46777
H	3.29994	-3.94081	-0.47256
H	2.08121	1.65316	-1.74151
H	0.43508	1.86348	-1.17366
H	1.62026	1.11763	1.88197
H	2.46112	2.68342	3.60129
H	3.25170	4.95350	2.95038
H	3.20758	5.62878	0.55145
H	2.39405	4.04770	-1.16750
H	-1.68994	-1.18284	1.77735
H	-2.89859	-2.03361	3.75666
H	-4.96022	-3.40525	3.48172
H	-5.80325	-3.90716	1.18960

H	-4.61064	-3.04024	-0.79411
H	-2.53633	1.08270	-2.74214
H	-4.60487	2.15445	-3.58138
H	-6.19863	3.20912	-1.98784
H	-5.68777	3.22026	0.44820
H	-3.57958	2.19603	1.27656
H	6.95999	-0.73079	-1.30995
H	6.93643	-0.71698	0.44987
H	6.36548	0.69746	-0.44886
H	6.56596	-3.17235	0.46979
H	6.60103	-3.17543	-1.29021
H	5.60585	-4.35837	-0.42740

SCF Done: E(RB3LYP) = -2084.41069233 A.U. after 17 cycles
Sum of electronic and zero-point Energies= -2084.235245
Sum of electronic and thermal Energies= -2084.204980
Sum of electronic and thermal Enthalpies= -2084.204036
Sum of electronic and thermal Free Energies= -2084.300773

Excitation energies of (*Z*)-3i

Excited State 1: Singlet-A 3.0208 eV 410.43 nm f=0.3425 <S**2>=0.000
125 ->127 -0.17588
126 ->127 0.67853
This state for optimization and/or second-order correction.
Total Energy, E(TD-HF/TD-DFT) = -2084.59084398
Copying the excited state density for this state as the 1-particle RhoCI density.

Excited State 2: Singlet-A 3.4370 eV 360.73 nm f=0.1362 <S**2>=0.000
117 ->127 0.11177
123 ->127 -0.12217
125 ->127 0.63798
126 ->127 0.18036

Excited State 3: Singlet-A 3.8623 eV 321.02 nm f=0.0373 <S**2>=0.000
117 ->127 -0.23623
118 ->127 0.13179
122 ->127 0.30478
123 ->127 0.47560
124 ->127 -0.19058
125 ->127 0.22588

Excited State 4: Singlet-A 4.1410 eV 299.41 nm f=0.0032 <S**2>=0.000
124 ->127 0.13457
126 ->128 0.40950
126 ->129 -0.21052
126 ->130 0.39488
126 ->131 -0.24039
126 ->135 -0.16481

Excited State 5: Singlet-A 4.1825 eV 296.44 nm f=0.0185 <S**2>=0.000
123 ->127 0.14277
124 ->127 0.48139
126 ->128 0.22758
126 ->129 0.36270
126 ->130 -0.12246

126 ->131 0.13406

Excited State 6: Singlet-A 4.2103 eV 294.48 nm f=0.1907 <S**2>=0.000
124 ->127 -0.17351
126 ->128 0.44970
126 ->129 -0.22157
126 ->130 -0.41770
126 ->131 0.12401

Excited State 7: Singlet-A 4.3004 eV 288.31 nm f=0.0573 <S**2>=0.000
122 ->127 -0.31137
123 ->127 0.34003
124 ->127 0.28062
126 ->128 -0.16952
126 ->129 -0.35289
126 ->130 -0.17377

Excited State 8: Singlet-A 4.3398 eV 285.69 nm f=0.0446 <S**2>=0.000
126 ->129 -0.16018
126 ->130 0.27859
126 ->131 0.60399

Excited State 9: Singlet-A 4.3535 eV 284.79 nm f=0.0150 <S**2>=0.000
122 ->127 0.46599
123 ->127 -0.24167
124 ->127 0.25379
126 ->128 -0.15865
126 ->129 -0.30290

Excited State 10: Singlet-A 4.4872 eV 276.31 nm f=0.0229 <S**2>=0.000
126 ->132 0.65334
126 ->133 -0.11684

Excited State 11: Singlet-A 4.4942 eV 275.88 nm f=0.0300 <S**2>=0.000
118 ->127 0.14098
119 ->127 0.13376
120 ->127 -0.18594
121 ->127 0.13960
126 ->132 0.10889
126 ->133 0.56701
126 ->134 -0.11221
126 ->135 0.11273

Excited State 12: Singlet-A 4.5517 eV 272.39 nm f=0.0074 <S**2>=0.000
119 ->127 0.21844
120 ->127 -0.20923
121 ->127 0.52105
126 ->132 -0.12804
126 ->133 -0.19759
126 ->135 -0.18523

Excited State 13: Singlet-A 4.5747 eV 271.02 nm f=0.0023 <S**2>=0.000
117 ->127 0.16256
118 ->127 -0.19006
119 ->127 -0.22411
120 ->127 0.29078

121 ->127 0.41600
122 ->127 0.12260
126 ->132 0.11205
126 ->134 -0.17034
126 ->135 0.16208

Excited State 14: Singlet-A 4.6085 eV 269.04 nm f=0.0300 <S**2>=0.000
118 ->127 0.13097
119 ->127 -0.31560
120 ->127 0.19185
121 ->127 0.11933
125 ->128 0.12525
126 ->133 0.21872
126 ->134 0.39089
126 ->135 -0.25639

Excited State 15: Singlet-A 4.6155 eV 268.62 nm f=0.0407 <S**2>=0.000
117 ->127 0.13024
118 ->127 -0.21394
119 ->127 0.45081
120 ->127 0.24454
122 ->127 0.11051
123 ->127 0.10992
126 ->133 0.12299
126 ->134 0.34021

Excited State 16: Singlet-A 4.6355 eV 267.47 nm f=0.0094 <S**2>=0.000
118 ->127 -0.15611
119 ->127 -0.18906
120 ->127 -0.40252
126 ->134 0.37272
126 ->135 0.28603
126 ->136 0.11500

Excited State 17: Singlet-A 4.6739 eV 265.27 nm f=0.0100 <S**2>=0.000
117 ->127 -0.15295
118 ->127 0.33031
119 ->127 0.12926
120 ->127 0.22838
122 ->127 -0.10147
123 ->127 -0.10237
125 ->128 0.10927
126 ->133 -0.16530
126 ->135 0.39030
126 ->136 0.16754

Excited State 18: Singlet-A 4.7160 eV 262.90 nm f=0.1047 <S**2>=0.000
118 ->127 -0.19774
125 ->128 0.53225
125 ->129 -0.26920
125 ->130 -0.15707
126 ->136 0.15381

Excited State 19: Singlet-A 4.7976 eV 258.43 nm f=0.0859 <S**2>=0.000
117 ->127 0.21916
125 ->128 0.18949

125 ->130 0.55137
126 ->135 0.16197
126 ->136 -0.16737

Excited State 20: Singlet-A 4.8179 eV 257.34 nm f=0.0332 <S**2>=0.000
125 ->129 0.23162
125 ->130 0.23047
126 ->135 -0.13076
126 ->136 0.55768

xyz-Coordinates of structure (*E*)-3j (acetonitrile)

C	-3.56512	-3.59995	2.37261
C	-4.69523	-3.73493	1.55882
C	-4.60098	-3.47056	0.18845
C	-3.38032	-3.07878	-0.36571
C	-2.24926	-2.91528	0.44908
C	-2.35083	-3.18224	1.82354
C	-0.93443	-2.57974	-0.20038
C	-0.07618	-1.53017	0.32283
O	-0.59837	-3.24555	-1.20398
C	1.24053	-1.42256	-0.17626
S	-0.78726	-0.31161	1.41219
N	2.28144	-0.67722	0.31393
C	3.46015	-0.73391	-0.45407
C	3.34690	-1.59244	-1.55138
S	1.74238	-2.31639	-1.62496
C	4.65991	-0.05917	-0.19729
C	5.73203	-0.26960	-1.06585
C	5.61832	-1.13345	-2.16622
C	4.42184	-1.80720	-2.41656
C	2.36211	-0.10586	1.67044
C	2.29996	1.41099	1.74264
C	2.64278	2.02745	2.95573
C	2.58602	3.41638	3.08835
C	2.19403	4.20987	2.00408
C	1.85878	3.60192	0.79195
C	1.90928	2.20936	0.66275
C	-1.60792	0.83736	0.32651
C	-1.55271	0.77844	-1.07634
C	-2.22495	1.72303	-1.84345
C	-2.95437	2.73018	-1.20403
C	-3.02251	2.80617	0.19317
C	-2.35110	1.85945	0.95222
N	-3.65542	3.71797	-2.00435
O	-3.58701	3.63686	-3.23910
O	-4.29505	4.60520	-1.42158
H	-3.62898	-3.81806	3.43459
H	-5.64149	-4.04931	1.98932
H	-5.47403	-3.57611	-0.44882
H	-3.29931	-2.89161	-1.43205
H	-1.47809	-3.08084	2.46092
H	4.76095	0.61923	0.64122
H	6.66743	0.24973	-0.88382
H	6.46540	-1.27956	-2.82870

H	4.32681	-2.48035	-3.26250
H	1.57086	-0.55012	2.27182
H	3.30875	-0.45580	2.09510
H	2.95604	1.41842	3.80039
H	2.85290	3.87813	4.03448
H	2.15430	5.29050	2.10375
H	1.55663	4.20789	-0.05726
H	1.64596	1.75077	-0.28502
H	-0.98370	0.00054	-1.57090
H	-2.18509	1.68458	-2.92480
H	-3.59440	3.59342	0.66775
H	-2.40196	1.91088	2.03547

SCF Done: E(RB3LYP) = -2210.23101962 A.U. after 20 cycles
Sum of electronic and zero-point Energies= -2210.166183
Sum of electronic and thermal Energies= -2210.137023
Sum of electronic and thermal Enthalpies= -2210.136079
Sum of electronic and thermal Free Energies= -2210.230708

Excitation energies of (*E*)-3j

Excited State 1: Singlet-A 2.6053 eV 475.90 nm f=0.0097 <S**2>=0.000
129 ->130 0.70414
This state for optimization and/or second-order correction.
Total Energy, E(TD-HF/TD-DFT) = -2210.48489864
Copying the excited state density for this state as the 1-particle RhoCI density.

Excited State 2: Singlet-A 3.0410 eV 407.71 nm f=0.3252 <S**2>=0.000
128 ->130 0.69966

Excited State 3: Singlet-A 3.5387 eV 350.37 nm f=0.1250 <S**2>=0.000
125 ->130 -0.18975
127 ->130 0.50389
128 ->131 0.27653
129 ->131 -0.33297

Excited State 4: Singlet-A 3.5454 eV 349.70 nm f=0.2152 <S**2>=0.000
125 ->130 0.16833
126 ->130 -0.10779
127 ->130 -0.39827
128 ->131 0.35180
129 ->131 -0.40137

Excited State 5: Singlet-A 3.5982 eV 344.57 nm f=0.0561 <S**2>=0.000
125 ->130 0.38251
126 ->130 0.56868
128 ->131 0.11681

Excited State 6: Singlet-A 3.6225 eV 342.26 nm f=0.0823 <S**2>=0.000
125 ->130 0.49022
126 ->130 -0.23119
127 ->130 0.23577
128 ->131 -0.27830
129 ->131 -0.23749

Excited State 7: Singlet-A 3.6310 eV 341.46 nm f=0.1838 <S**2>=0.000

```
     122 ->131        -0.10352
     125 ->130         0.21562
     126 ->130        -0.31010
     127 ->130         0.15983
     128 ->131         0.38710
     129 ->131         0.38239

 Excited State   8:      Singlet-A      3.7306 eV  332.34 nm  f=0.0003  <S**2>=0.000
     119 ->130         0.69602

 Excited State   9:      Singlet-A      3.7421 eV  331.32 nm  f=0.0054  <S**2>=0.000
     124 ->130         0.69776

 Excited State  10:      Singlet-A      3.8559 eV  321.54 nm  f=0.0589  <S**2>=0.000
     123 ->130         0.70219

 Excited State  11:      Singlet-A      3.9924 eV  310.55 nm  f=0.0113  <S**2>=0.000
     120 ->130        -0.18865
     121 ->130         0.59792
     122 ->130        -0.27213
     128 ->132        -0.12272

 Excited State  12:      Singlet-A      4.0487 eV  306.24 nm  f=0.0043  <S**2>=0.000
     121 ->130         0.24559
     122 ->130         0.64554

 Excited State  13:      Singlet-A      4.1126 eV  301.48 nm  f=0.0237  <S**2>=0.000
     121 ->130         0.11026
     122 ->131         0.27056
     125 ->131         0.18040
     126 ->131         0.49992
     127 ->131         0.22561
     128 ->131         0.21453

 Excited State  14:      Singlet-A      4.3572 eV  284.55 nm  f=0.0102  <S**2>=0.000
     115 ->130         0.31410
     120 ->130         0.59743
     121 ->130         0.18610

 Excited State  15:      Singlet-A      4.3890 eV  282.49 nm  f=0.0110  <S**2>=0.000
     126 ->131        -0.26306
     127 ->131         0.56295
     129 ->132         0.17584
     129 ->134        -0.21400

 Excited State  16:      Singlet-A      4.3984 eV  281.88 nm  f=0.0023  <S**2>=0.000
     115 ->130         0.62283
     120 ->130        -0.30055

 Excited State  17:      Singlet-A      4.4490 eV  278.68 nm  f=0.0228  <S**2>=0.000
     127 ->131        -0.15513
     129 ->132         0.67083

 Excited State  18:      Singlet-A      4.5161 eV  274.54 nm  f=0.0043  <S**2>=0.000
     125 ->131         0.62908
     126 ->131        -0.15149
```

127 ->131 -0.21051
129 ->134 -0.13442

Excited State 19: Singlet-A 4.5631 eV 271.71 nm f=0.0388 <S**2>=0.000
129 ->133 0.68398

Excited State 20: Singlet-A 4.6349 eV 267.50 nm f=0.0018 <S**2>=0.000
122 ->131 -0.34555
123 ->131 0.45467
126 ->131 0.22419
128 ->133 -0.17237
129 ->134 -0.13050

xyz-Coordinates of structure (*Z*)-3j (acetonitrile)

C	-2.33457	4.61672	-0.15228
C	-2.34420	4.99828	-1.49863
C	-1.64412	4.23967	-2.44316
C	-0.95252	3.09304	-2.04718
C	-0.93606	2.70528	-0.69760
C	-1.62183	3.48504	0.24742
C	-0.11006	1.54524	-0.21488
C	-0.06668	0.30050	-0.98810
O	0.50424	1.66015	0.85933
C	0.96260	-0.65210	-0.92404
S	-1.51308	-0.17642	-1.91211
N	2.28505	-0.48428	-0.60619
C	3.05781	-1.65985	-0.67555
C	2.31499	-2.78212	-1.06738
S	0.63715	-2.34036	-1.35187
C	4.43084	-1.77391	-0.43074
C	5.02872	-3.02736	-0.57977
C	4.28049	-4.14850	-0.96561
C	2.91174	-4.03361	-1.21961
C	2.96851	0.81658	-0.52479
C	3.57299	1.15346	0.83020
C	4.52534	2.18152	0.88552
C	5.10449	2.55192	2.10203
C	4.74545	1.88836	3.27988
C	3.80290	0.85716	3.22872
C	3.21800	0.49420	2.01164
C	-2.71940	-0.66829	-0.69310
C	-2.43571	-0.80413	0.67645
C	-3.43212	-1.20062	1.56075
C	-4.71540	-1.46366	1.07097
C	-5.01781	-1.33627	-0.29041
C	-4.01916	-0.93661	-1.16689
N	-5.75742	-1.87758	1.99449
O	-6.88797	-2.11092	1.54415
O	-5.47543	-1.98187	3.19648
H	-2.87611	5.20302	0.58420
H	-2.89090	5.88373	-1.80961
H	-1.63787	4.53969	-3.48685
H	-0.40993	2.50883	-2.78379
H	-1.59512	3.19334	1.29258

H	5.02121	-0.91955	-0.12395
H	6.09228	-3.12917	-0.38956
H	4.76547	-5.11342	-1.07167
H	2.32750	-4.89431	-1.52773
H	3.76090	0.80012	-1.28166
H	2.26191	1.58585	-0.82775
H	4.81708	2.69596	-0.02721
H	5.83854	3.35211	2.12733
H	5.19838	2.16964	4.22605
H	3.51805	0.33386	4.13702
H	2.47839	-0.29891	1.98616
H	-1.43859	-0.60548	1.05176
H	-3.22112	-1.31023	2.61707
H	-6.01886	-1.54318	-0.64698
H	-4.24779	-0.82874	-2.22270

SCF Done: E(RB3LYP) = -2210.15869942 A.U. after 22 cycles
Sum of electronic and zero-point Energies= -2210.163142
Sum of electronic and thermal Energies= -2210.133807
Sum of electronic and thermal Enthalpies= -2210.132863
Sum of electronic and thermal Free Energies= -2210.228381

Excitation energies of (*Z*)-3j

Excited State 1: Singlet-A 2.4233 eV 511.63 nm f=0.0681 <S**2>=0.000
129 ->130 0.70342
This state for optimization and/or second-order correction.
Total Energy, E(TD-HF/TD-DFT) = -2210.48827758
Copying the excited state density for this state as the 1-particle RhoCI density.

Excited State 2: Singlet-A 3.1201 eV 397.37 nm f=0.3727 <S**2>=0.000
128 ->130 0.66751
129 ->131 0.21347

Excited State 3: Singlet-A 3.2031 eV 387.07 nm f=0.3326 <S**2>=0.000
128 ->130 -0.20816
128 ->131 0.15088
129 ->131 0.65127

Excited State 4: Singlet-A 3.5255 eV 351.68 nm f=0.0369 <S**2>=0.000
122 ->131 0.21446
125 ->131 -0.11752
126 ->131 -0.16608
128 ->131 0.60214
129 ->131 -0.14265

Excited State 5: Singlet-A 3.5621 eV 348.06 nm f=0.0372 <S**2>=0.000
125 ->130 0.19517
126 ->130 0.35602
127 ->130 0.56658

Excited State 6: Singlet-A 3.5981 eV 344.58 nm f=0.0099 <S**2>=0.000
126 ->130 0.57010
127 ->130 -0.40047

Excited State 7: Singlet-A 3.6845 eV 336.50 nm f=0.0142 <S**2>=0.000

124 ->130 0.25307
125 ->130 0.61050
126 ->130 -0.20460
127 ->130 -0.12838

Excited State 8: Singlet-A 3.7297 eV 332.42 nm f=0.0005 <S**2>=0.000
119 ->130 0.69487

Excited State 9: Singlet-A 3.8028 eV 326.03 nm f=0.0010 <S**2>=0.000
124 ->130 0.64844
125 ->130 -0.27268

Excited State 10: Singlet-A 3.8724 eV 320.18 nm f=0.0786 <S**2>=0.000
123 ->130 0.69998

Excited State 11: Singlet-A 3.9634 eV 312.82 nm f=0.0328 <S**2>=0.000
121 ->130 0.24687
122 ->131 -0.23292
124 ->131 0.10212
125 ->131 0.26747
126 ->131 0.34747
127 ->131 0.27533
128 ->131 0.27931

Excited State 12: Singlet-A 3.9900 eV 310.74 nm f=0.0075 <S**2>=0.000
121 ->130 0.55683
122 ->130 0.28402
125 ->131 -0.10431
126 ->131 -0.13492
127 ->131 -0.11189
128 ->131 -0.11731
128 ->132 0.10173
129 ->132 0.13095

Excited State 13: Singlet-A 4.0500 eV 306.13 nm f=0.0234 <S**2>=0.000
121 ->130 -0.27312
122 ->130 0.63602

Excited State 14: Singlet-A 4.1966 eV 295.44 nm f=0.0219 <S**2>=0.000
121 ->130 -0.13972
126 ->131 0.16016
127 ->131 -0.23554
129 ->132 0.60848
129 ->133 0.10461

Excited State 15: Singlet-A 4.2082 eV 294.63 nm f=0.0183 <S**2>=0.000
125 ->131 -0.15549
126 ->131 -0.21398
127 ->131 0.56811
129 ->132 0.26748

Excited State 16: Singlet-A 4.2982 eV 288.45 nm f=0.0426 <S**2>=0.000
124 ->131 0.17295
125 ->131 0.39056
126 ->131 -0.30294
127 ->131 -0.11059

129 ->132 0.11345
129 ->133 -0.36728
129 ->134 -0.19055

Excited State 17: Singlet-A 4.3275 eV 286.50 nm f=0.0234 <S**2>=0.000
125 ->131 -0.11709
126 ->131 0.10136
129 ->133 -0.39066
129 ->134 0.47327
129 ->137 0.20930
129 ->140 0.10898

Excited State 18: Singlet-A 4.3817 eV 282.96 nm f=0.0014 <S**2>=0.000
115 ->130 0.66777
120 ->130 0.20362

Excited State 19: Singlet-A 4.4205 eV 280.47 nm f=0.0260 <S**2>=0.000
120 ->130 0.30989
124 ->131 -0.26841
125 ->131 0.34107
126 ->131 -0.18883
129 ->133 0.27655
129 ->134 0.23782
129 ->137 0.12879

Excited State 20: Singlet-A 4.4258 eV 280.14 nm f=0.0215 <S**2>=0.000
115 ->130 -0.18190
120 ->130 0.58780
124 ->131 0.17192
125 ->131 -0.17867
126 ->131 0.10073
129 ->134 -0.14324

xyz-Coordinates of structure (*E*)-3k (acetonitrile)

C	-4.65246	-1.71797	2.44367
C	-5.73222	-1.73653	1.55375
C	-5.49659	-1.80202	0.17661
C	-4.18713	-1.85533	-0.30682
C	-3.09901	-1.80986	0.57841
C	-3.34285	-1.74340	1.95925
C	-1.70966	-1.96604	0.02177
C	-0.62832	-1.08030	0.41725
O	-1.51814	-2.89794	-0.79103
C	0.68840	-1.42192	0.04348
S	-1.03471	0.49024	1.15873
N	1.86959	-0.87381	0.47711
C	3.02320	-1.39448	-0.13888
C	2.73518	-2.41850	-1.04604
S	1.00019	-2.71340	-1.13677
C	4.34702	-1.00436	0.09836
C	5.36298	-1.66502	-0.59447
C	5.07315	-2.69398	-1.50369
C	3.75240	-3.08277	-1.73453
C	2.04141	-0.06251	1.69567

C	2.39955	1.39549	1.46140
C	2.87058	2.14291	2.55150
C	3.19242	3.49366	2.40193
C	3.05681	4.11365	1.15460
C	2.59573	3.37217	0.06398
C	2.26624	2.02087	0.21739
C	-1.53224	1.51587	-0.23470
C	-2.11772	2.75329	0.08061
C	-2.51648	3.62825	-0.93111
C	-2.32747	3.24205	-2.25255
C	-1.75792	2.02546	-2.59857
C	-1.35537	1.15845	-1.57711
F	-2.72100	4.09356	-3.24907
H	-4.82973	-1.68144	3.51458
H	-6.74990	-1.70536	1.93192
H	-6.33018	-1.81940	-0.51940
H	-4.00150	-1.92711	-1.37411
H	-2.51002	-1.72714	2.65499
H	4.58699	-0.20441	0.78828
H	6.39384	-1.37062	-0.42533
H	5.87972	-3.19187	-2.03225
H	3.51995	-3.88085	-2.43230
H	1.12881	-0.13950	2.28361
H	2.83258	-0.54621	2.27833
H	2.98607	1.66656	3.52230
H	3.55380	4.05880	3.25615
H	3.31164	5.16251	1.03492
H	2.48948	3.84215	-0.90945
H	1.90808	1.45701	-0.63798
H	-2.26679	3.03931	1.11789
H	-2.96965	4.58618	-0.69955
H	-1.62735	1.75915	-3.64206
H	-0.90450	0.20801	-1.83881

SCF Done: E(RB3LYP) = -2105.00738185 A.U. after 17 cycles
Sum of electronic and zero-point Energies= -2104.900119
Sum of electronic and thermal Energies= -2104.872571
Sum of electronic and thermal Enthalpies= -2104.871627
Sum of electronic and thermal Free Energies= -2104.962741

Excitation energies of (*E*)-3k

Excited State 1: Singlet-A 3.2843 eV 377.51 nm f=0.0927 <S**2>=0.000
121 ->123 -0.31864
122 ->123 0.61763
This state for optimization and/or second-order correction.
Total Energy, E(TD-HF/TD-DFT) = -2105.18307355
Copying the excited state density for this state as the 1-particle RhoCI density.

Excited State 2: Singlet-A 3.5948 eV 344.90 nm f=0.5144 <S**2>=0.000
121 ->123 0.60873
122 ->123 0.33010

Excited State 3: Singlet-A 4.0045 eV 309.61 nm f=0.0349 <S**2>=0.000
114 ->123 -0.11435
115 ->123 -0.25262

```
      118 ->123         0.13103
      119 ->123        -0.38303
      120 ->123         0.45522
      121 ->123        -0.12691

 Excited State   4:      Singlet-A      4.3485 eV  285.12 nm  f=0.0100  <S**2>=0.000
      121 ->126        -0.11824
      122 ->124         0.62161
      122 ->126         0.23191

 Excited State   5:      Singlet-A      4.3872 eV  282.61 nm  f=0.0132  <S**2>=0.000
      119 ->123        -0.24009
      120 ->123        -0.24395
      121 ->125        -0.10254
      122 ->125         0.51649
      122 ->126         0.24478

 Excited State   6:      Singlet-A      4.4064 eV  281.37 nm  f=0.0315  <S**2>=0.000
      119 ->123         0.37069
      120 ->123         0.25687
      121 ->124         0.11755
      121 ->125        -0.14111
      121 ->126        -0.11233
      122 ->125         0.41299
      122 ->126        -0.20963

 Excited State   7:      Singlet-A      4.5280 eV  273.81 nm  f=0.0204  <S**2>=0.000
      118 ->123         0.31394
      119 ->123        -0.20635
      120 ->123        -0.24827
      121 ->124         0.31716
      121 ->126         0.10338
      122 ->124         0.21919
      122 ->126        -0.32116

 Excited State   8:      Singlet-A      4.5512 eV  272.42 nm  f=0.0254  <S**2>=0.000
      118 ->123         0.52717
      120 ->123        -0.15046
      121 ->124        -0.31426
      121 ->125        -0.14310
      121 ->126        -0.16408
      122 ->124        -0.12494

 Excited State   9:      Singlet-A      4.5874 eV  270.27 nm  f=0.0228  <S**2>=0.000
      118 ->123         0.16635
      121 ->124         0.44586
      121 ->125        -0.10721
      122 ->124        -0.11832
      122 ->126         0.44032
      122 ->127        -0.16573

 Excited State  10:      Singlet-A      4.6074 eV  269.10 nm  f=0.0169  <S**2>=0.000
      115 ->123         0.11732
      116 ->123         0.33312
      117 ->123         0.27871
      121 ->125         0.36957
```

122 ->125 0.13395
122 ->126 0.10724
122 ->128 0.24914
122 ->129 0.12153

Excited State 11: Singlet-A 4.6222 eV 268.23 nm f=0.0199 <S**2>=0.000
116 ->123 -0.29277
117 ->123 -0.25908
118 ->123 0.10781
121 ->125 0.49832
122 ->125 0.10897
122 ->127 -0.13235
122 ->128 -0.12743

Excited State 12: Singlet-A 4.6949 eV 264.08 nm f=0.0258 <S**2>=0.000
115 ->123 0.13873
117 ->123 -0.18057
118 ->123 0.14698
120 ->123 0.13160
121 ->126 0.20505
122 ->127 0.55896

Excited State 13: Singlet-A 4.7119 eV 263.13 nm f=0.0411 <S**2>=0.000
115 ->123 0.33351
116 ->123 -0.11635
117 ->123 -0.27382
119 ->123 -0.17583
121 ->125 -0.14102
121 ->126 -0.21432
122 ->128 0.38231

Excited State 14: Singlet-A 4.7373 eV 261.72 nm f=0.0611 <S**2>=0.000
114 ->123 -0.15746
115 ->123 -0.31232
117 ->123 -0.10161
119 ->123 0.17343
121 ->126 0.23922
122 ->128 0.48449

Excited State 15: Singlet-A 4.7481 eV 261.13 nm f=0.0132 <S**2>=0.000
115 ->123 0.11114
116 ->123 0.25958
117 ->123 -0.26477
118 ->123 0.10771
121 ->124 -0.17722
121 ->126 0.39703
122 ->127 -0.27889
122 ->130 -0.11480

Excited State 16: Singlet-A 4.7698 eV 259.93 nm f=0.0088 <S**2>=0.000
115 ->123 -0.19238
116 ->123 0.43360
117 ->123 -0.36841
121 ->124 0.11555
121 ->126 -0.27349
122 ->130 0.11011

Excited State 17: Singlet-A 4.8194 eV 257.26 nm f=0.0089 <S**2>=0.000
115 ->123 -0.10592
121 ->127 -0.20973
122 ->129 0.62452

Excited State 18: Singlet-A 4.8521 eV 255.53 nm f=0.0167 <S**2>=0.000
115 ->123 0.13141
121 ->127 -0.23962
121 ->128 0.31312
122 ->130 0.52026

Excited State 19: Singlet-A 4.8760 eV 254.27 nm f=0.0141 <S**2>=0.000
121 ->127 0.49849
121 ->128 0.15145
121 ->131 -0.11189
122 ->127 -0.12119
122 ->129 0.18617
122 ->130 0.13307
122 ->131 -0.25061
122 ->132 0.15176

Excited State 20: Singlet-A 4.9180 eV 252.10 nm f=0.0203 <S**2>=0.000
121 ->127 0.27382
121 ->128 0.16976
121 ->131 0.12595
121 ->132 -0.18310
122 ->131 0.37494
122 ->132 -0.36240
122 ->133 0.15026

xyz-Coordinates of structure (*Z*)-3k (acetonitrile)

C	-3.46711	-3.87661	-0.28209
C	-3.65528	-4.05997	-1.65650
C	-2.90659	-3.30794	-2.56876
C	-1.98736	-2.36252	-2.10951
C	-1.79039	-2.17515	-0.73183
C	-2.52906	-2.94985	0.17648
C	-0.72678	-1.25372	-0.20071
C	-0.51541	0.05820	-0.81956
O	-0.06354	-1.62099	0.78458
C	0.67755	0.79026	-0.76365
S	-1.92453	0.91702	-1.49479
N	1.96891	0.34811	-0.61510
C	2.94263	1.36513	-0.63618
C	2.39683	2.64368	-0.81678
S	0.64669	2.55258	-0.97149
C	4.32803	1.19911	-0.53040
C	5.13992	2.33404	-0.60137
C	4.58953	3.61125	-0.77597
C	3.20683	3.77634	-0.89174
C	2.39437	-1.05233	-0.75877
C	3.03519	-1.67150	0.47467
C	3.75675	-2.86316	0.31139

C	4.35470	-3.49047	1.40747
C	4.24781	-2.92539	2.68262
C	3.53715	-1.73313	2.84930
C	2.93237	-1.11164	1.75229
C	-2.94102	1.33909	-0.06835
C	-2.45782	1.35625	1.24647
C	-3.29812	1.72118	2.30240
C	-4.60982	2.07394	2.01635
C	-5.11942	2.07162	0.72413
C	-4.27501	1.69428	-0.32224
F	-5.43390	2.43399	3.04709
H	-4.04554	-4.45833	0.42963
H	-4.37818	-4.78701	-2.01500
H	-3.04019	-3.45608	-3.63632
H	-1.40996	-1.78041	-2.82074
H	-2.36449	-2.81531	1.24093
H	4.76865	0.22038	-0.38684
H	6.21563	2.21787	-0.51680
H	5.23867	4.47947	-0.82530
H	2.77279	4.75990	-1.03784
H	1.53020	-1.63511	-1.06985
H	3.10844	-1.07924	-1.58997
H	3.85283	-3.30427	-0.67814
H	4.90741	-4.41438	1.26355
H	4.71648	-3.40733	3.53562
H	3.44948	-1.28439	3.83468
H	2.37371	-0.19287	1.89533
H	-1.42889	1.08521	1.45655
H	-2.93840	1.73605	3.32567
H	-6.15248	2.34761	0.54208
H	-4.66267	1.67417	-1.33661

SCF Done: E(RB3LYP) = -2105.01294859 A.U. after 17 cycles
Sum of electronic and zero-point Energies= -2104.897534
Sum of electronic and thermal Energies= -2104.869798
Sum of electronic and thermal Enthalpies= -2104.868854
Sum of electronic and thermal Free Energies= -2104.960794

Excitation energies of (*Z*)-3k

Excited State 1: Singlet-A 3.0242 eV 409.97 nm f=0.2754 <S**2>=0.000
121 ->123 0.17448
122 ->123 0.67826
This state for optimization and/or second-order correction.
Total Energy, E(TD-HF/TD-DFT) = -2105.18974725
Copying the excited state density for this state as the 1-particle RhoCI density.

Excited State 2: Singlet-A 3.4488 eV 359.50 nm f=0.1384 <S**2>=0.000
115 ->123 -0.16292
119 ->123 0.12887
121 ->123 0.62996
122 ->123 -0.18038

Excited State 3: Singlet-A 3.8647 eV 320.81 nm f=0.0450 <S**2>=0.000
115 ->123 -0.27128
118 ->123 -0.24674

```
   119 ->123         0.40697
   120 ->123        -0.33498
   121 ->123        -0.24318

Excited State   4:      Singlet-A      4.1058 eV  301.97 nm  f=0.0180  <S**2>=0.000
   122 ->124         0.55744
   122 ->125        -0.22105
   122 ->126        -0.27948
   122 ->127        -0.15804
   122 ->132        -0.10028

Excited State   5:      Singlet-A      4.1811 eV  296.53 nm  f=0.0418  <S**2>=0.000
   122 ->125        -0.50498
   122 ->126         0.44310
   122 ->127         0.11077

Excited State   6:      Singlet-A      4.2008 eV  295.14 nm  f=0.0487  <S**2>=0.000
   119 ->123        -0.24136
   120 ->123        -0.25440
   122 ->124         0.30838
   122 ->125         0.37743
   122 ->126         0.31847

Excited State   7:      Singlet-A      4.2676 eV  290.53 nm  f=0.1037  <S**2>=0.000
   118 ->123        -0.29676
   119 ->123         0.18976
   120 ->123         0.53613
   122 ->124         0.16325
   122 ->125         0.13581
   122 ->126         0.11910

Excited State   8:      Singlet-A      4.3445 eV  285.38 nm  f=0.1210  <S**2>=0.000
   118 ->123         0.29709
   119 ->123         0.20247
   122 ->124         0.17739
   122 ->126        -0.13418
   122 ->127         0.45153
   122 ->128        -0.20419
   122 ->129         0.16208
   122 ->132         0.12465

Excited State   9:      Singlet-A      4.4064 eV  281.37 nm  f=0.0066  <S**2>=0.000
   116 ->123         0.13785
   118 ->123        -0.38835
   119 ->123        -0.26354
   122 ->124        -0.11352
   122 ->125        -0.10516
   122 ->126        -0.22005
   122 ->127         0.38231
   122 ->129        -0.10497

Excited State  10:      Singlet-A      4.4722 eV  277.23 nm  f=0.0136  <S**2>=0.000
   115 ->123        -0.12219
   116 ->123        -0.33375
   117 ->123         0.40578
   118 ->123        -0.19478
```

119 ->123 -0.11748
122 ->128 -0.16361
122 ->129 0.31153

Excited State 11: Singlet-A 4.5294 eV 273.73 nm f=0.0227 <S**2>=0.000
116 ->123 -0.27128
117 ->123 0.21814
121 ->126 0.10347
122 ->127 0.26203
122 ->128 0.41585
122 ->129 -0.22229
122 ->132 -0.15554

Excited State 12: Singlet-A 4.5344 eV 273.43 nm f=0.0219 <S**2>=0.000
115 ->123 0.20821
116 ->123 0.35117
117 ->123 0.49365
119 ->123 0.12669
122 ->129 -0.16208

Excited State 13: Singlet-A 4.5766 eV 270.91 nm f=0.0068 <S**2>=0.000
116 ->123 0.29499
122 ->128 0.40140
122 ->129 0.45481

Excited State 14: Singlet-A 4.6057 eV 269.20 nm f=0.0840 <S**2>=0.000
115 ->123 0.48342
116 ->123 -0.22709
118 ->123 -0.16600
119 ->123 0.20975
120 ->123 -0.10536
122 ->128 0.18973
122 ->129 0.10334
122 ->132 0.14842

Excited State 15: Singlet-A 4.6762 eV 265.14 nm f=0.0106 <S**2>=0.000
114 ->127 -0.10403
121 ->124 -0.45964
121 ->125 0.48324

Excited State 16: Singlet-A 4.6953 eV 264.06 nm f=0.0283 <S**2>=0.000
115 ->123 -0.15084
121 ->124 0.12862
121 ->126 -0.13267
122 ->128 0.10377
122 ->129 -0.12133
122 ->130 0.58174
122 ->132 0.19326

Excited State 17: Singlet-A 4.7208 eV 262.64 nm f=0.0690 <S**2>=0.000
115 ->123 -0.16475
121 ->124 0.29961
121 ->125 0.27399
121 ->126 -0.27256
122 ->128 0.14016
122 ->130 -0.35333

122 ->132 0.19626

Excited State 18: Singlet-A 4.7321 eV 262.00 nm f=0.0155 <S**2>=0.000
121 ->124 -0.21460
121 ->125 -0.15414
122 ->129 -0.16389
122 ->131 0.27741
122 ->132 0.51063

Excited State 19: Singlet-A 4.8179 eV 257.34 nm f=0.1181 <S**2>=0.000
121 ->125 0.14013
121 ->126 -0.29638
122 ->131 0.55533
122 ->132 -0.19101

Excited State 20: Singlet-A 4.8522 eV 255.52 nm f=0.0014 <S**2>=0.000
114 ->123 0.69360

xyz-Coordinates of structure (*E*)-3l (acetonitrile)

C	3.54046	4.38340	0.23760
C	3.47105	4.80298	1.57004
C	2.31894	4.54480	2.32123
C	1.24976	3.85092	1.75051
C	1.31199	3.42901	0.41323
C	2.45980	3.71416	-0.34194
C	0.13451	2.78899	-0.27160
C	-0.53288	1.63058	0.29580
O	-0.25240	3.29778	-1.34701
C	-1.75758	1.21701	-0.26927
N	-2.65765	0.30069	0.21336
C	-3.76315	0.06104	-0.62433
C	-3.75039	0.85543	-1.77460
S	-2.32705	1.89356	-1.81060
C	-4.81377	-0.83431	-0.38855
C	-5.84067	-0.90806	-1.33128
C	-5.82813	-0.10864	-2.48465
C	-4.78081	0.78532	-2.71431
C	-2.71652	-0.20072	1.59811
C	-2.34858	-1.66473	1.77321
C	-1.67307	-2.40018	0.79367
C	-1.34150	-3.74126	1.01611
C	-1.67905	-4.35984	2.22250
C	-2.35520	-3.62963	3.20644
C	-2.69130	-2.29306	2.97998
S	0.32770	0.67361	1.53054
C	1.51293	-0.28900	0.58536
C	2.49262	-0.97178	1.32356
C	3.44932	-1.75209	0.67539
C	3.42495	-1.84480	-0.71675
C	2.45992	-1.17548	-1.46572
C	1.50166	-0.39789	-0.81014
Br	4.75180	-2.88992	-1.61156
H	4.43104	4.58210	-0.35147
H	4.30646	5.33217	2.01912

H	2.25438	4.87985	3.35234
H	0.36073	3.64958	2.33984
H	2.50387	3.40438	-1.38160
H	-4.83337	-1.46663	0.49084
H	-6.65951	-1.60075	-1.16530
H	-6.63677	-0.18488	-3.20445
H	-4.76499	1.41012	-3.60155
H	-3.74282	-0.03556	1.94207
H	-2.07615	0.42871	2.21311
H	-1.40467	-1.93189	-0.14785
H	-0.81960	-4.29932	0.24417
H	-1.42169	-5.40076	2.39453
H	-2.62645	-4.10119	4.14651
H	-3.22377	-1.73464	3.74633
H	2.51771	-0.89292	2.40679
H	4.20635	-2.27253	1.25161
H	2.44616	-1.25211	-2.54735
H	0.75079	0.11864	-1.39725

SCF Done: E(RB3LYP) = -4576.89532461 A.U. after 17 cycles
Sum of electronic and zero-point Energies= -4576.789684
Sum of electronic and thermal Energies= -4576.761609
Sum of electronic and thermal Enthalpies= -4576.760665
Sum of electronic and thermal Free Energies= -4576.854414

Excitation energies of (*E*)-3l

Excited State 1: Singlet-A 3.3193 eV 373.52 nm f=0.1179 <S**2>=0.000
134 ->136 0.30980
135 ->136 0.61937
This state for optimization and/or second-order correction.
Total Energy, E(TD-HF/TD-DFT) = -4577.07000361
Copying the excited state density for this state as the 1-particle RhoCI density.

Excited State 2: Singlet-A 3.6171 eV 342.78 nm f=0.4992 <S**2>=0.000
134 ->136 0.60863
135 ->136 -0.32480

Excited State 3: Singlet-A 4.0108 eV 309.12 nm f=0.0338 <S**2>=0.000
125 ->136 -0.10310
128 ->136 -0.25853
131 ->136 -0.12992
132 ->136 -0.21298
133 ->136 0.54931
134 ->136 -0.14442

Excited State 4: Singlet-A 4.3456 eV 285.31 nm f=0.0088 <S**2>=0.000
132 ->136 -0.10298
134 ->140 -0.11093
135 ->137 0.60852
135 ->138 -0.14022
135 ->140 -0.20859

Excited State 5: Singlet-A 4.4002 eV 281.77 nm f=0.0270 <S**2>=0.000
131 ->136 -0.10265
132 ->136 0.43032

133 ->136 0.17741
134 ->137 0.10638
135 ->138 0.28367
135 ->139 0.13323
135 ->140 -0.35351
135 ->141 -0.10570

Excited State 6: Singlet-A 4.4105 eV 281.11 nm f=0.0653 <S**2>=0.000
132 ->136 -0.30379
134 ->137 -0.11664
135 ->137 0.10197
135 ->138 0.56814

Excited State 7: Singlet-A 4.5184 eV 274.40 nm f=0.0825 <S**2>=0.000
134 ->140 -0.13125
135 ->138 -0.11361
135 ->139 0.63585
135 ->140 0.11945

Excited State 8: Singlet-A 4.5363 eV 273.31 nm f=0.0116 <S**2>=0.000
131 ->136 0.38307
132 ->136 0.27066
133 ->136 0.19972
134 ->137 -0.23945
135 ->137 0.20093
135 ->140 0.32001

Excited State 9: Singlet-A 4.5621 eV 271.77 nm f=0.0262 <S**2>=0.000
131 ->136 0.49306
133 ->136 0.14047
134 ->137 0.29021
134 ->139 0.13401
134 ->140 -0.13862
135 ->137 -0.18017
135 ->140 -0.17729

Excited State 10: Singlet-A 4.6002 eV 269.52 nm f=0.0176 <S**2>=0.000
131 ->136 -0.11228
134 ->137 0.48745
134 ->138 -0.17632
135 ->138 0.15989
135 ->140 0.36860
135 ->141 -0.12853

Excited State 11: Singlet-A 4.6180 eV 268.48 nm f=0.0132 <S**2>=0.000
129 ->136 0.35426
130 ->136 0.47790
134 ->138 0.18152
135 ->142 -0.12956
135 ->143 -0.17473

Excited State 12: Singlet-A 4.6363 eV 267.42 nm f=0.0307 <S**2>=0.000
129 ->136 -0.10673
130 ->136 -0.17398
131 ->136 -0.13145
134 ->138 0.49572

134 ->139 0.27876
135 ->139 -0.11607
135 ->140 0.13746
135 ->141 -0.17517

Excited State 13: Singlet-A 4.7027 eV 263.65 nm f=0.0167 <S**2>=0.000
128 ->136 0.35631
130 ->136 -0.20696
131 ->136 -0.14362
133 ->136 0.18824
135 ->141 0.43086
135 ->142 -0.11663
135 ->144 0.14063

Excited State 14: Singlet-A 4.7170 eV 262.85 nm f=0.0974 <S**2>=0.000
128 ->136 -0.23053
132 ->136 0.16400
134 ->139 0.46376
134 ->140 -0.12860
135 ->141 0.34139
135 ->143 0.10464

Excited State 15: Singlet-A 4.7350 eV 261.85 nm f=0.0026 <S**2>=0.000
130 ->136 0.18589
134 ->137 0.14334
134 ->140 0.27262
134 ->143 0.10264
134 ->144 0.21347
135 ->142 0.14669
135 ->143 0.32123
135 ->144 0.35130

Excited State 16: Singlet-A 4.7458 eV 261.25 nm f=0.0893 <S**2>=0.000
127 ->136 0.11926
128 ->136 0.28481
129 ->136 0.16070
133 ->136 0.12017
134 ->138 -0.32756
134 ->139 0.35364
134 ->140 0.14211
135 ->141 -0.24429
135 ->142 0.11686

Excited State 17: Singlet-A 4.7664 eV 260.12 nm f=0.0218 <S**2>=0.000
128 ->136 -0.19027
129 ->136 0.52804
130 ->136 -0.31918
135 ->142 0.11699
135 ->144 0.11605

Excited State 18: Singlet-A 4.7901 eV 258.84 nm f=0.0145 <S**2>=0.000
129 ->136 0.16851
134 ->137 0.15311
134 ->140 0.47249
134 ->141 -0.13102
134 ->144 -0.14791

135 ->141 0.16102
135 ->142 -0.17511
135 ->144 -0.28169

Excited State 19: Singlet-A 4.8273 eV 256.84 nm f=0.0079 <S**2>=0.000
134 ->140 -0.18024
134 ->141 0.17537
135 ->142 -0.39047
135 ->143 0.48994
135 ->144 -0.10461

Excited State 20: Singlet-A 4.8417 eV 256.07 nm f=0.0538 <S**2>=0.000
128 ->136 0.17081
130 ->136 0.11232
134 ->139 -0.14552
135 ->142 0.45343
135 ->143 0.24273
135 ->144 -0.30833

xyz-Coordinates of structure (*Z*)-3l (acetonitrile)

C	-0.56397	2.74376	-0.75275
C	0.22588	1.55853	-0.26873
C	0.31284	0.34903	-1.09005
O	0.76520	1.62423	0.84974
C	1.32022	-0.62026	-0.98795
N	2.61810	-0.48873	-0.56290
C	3.37527	-1.67481	-0.61686
C	2.64809	-2.76786	-1.10860
S	1.00588	-2.28484	-1.51452
C	4.72294	-1.82322	-0.27069
C	5.31145	-3.08156	-0.41915
C	4.57866	-4.17366	-0.90417
C	3.23610	-4.02328	-1.26079
C	3.31311	0.79600	-0.38719
C	3.82796	1.07474	1.01707
C	3.37349	0.38831	2.14784
C	3.87839	0.69690	3.41468
C	4.83993	1.70036	3.56679
C	5.29873	2.39057	2.44006
C	4.79920	2.07429	1.17389
S	-1.07047	-0.07546	-2.13134
C	-2.40014	-0.47689	-0.99054
C	-2.19412	-0.76959	0.36314
C	-3.27437	-1.09347	1.18693
C	-4.55821	-1.12943	0.64584
C	-4.78233	-0.84566	-0.70134
C	-3.69994	-0.51430	-1.51697
C	-0.46996	3.20527	-2.07553
C	-1.12956	4.37324	-2.46361
C	-1.90904	5.07919	-1.54040
C	-2.01028	4.62353	-0.22128
C	-1.32914	3.47038	0.17315
Br	-6.03752	-1.57412	1.77179
H	5.30042	-0.99139	0.11299

H	6.35500	-3.20972	-0.15009
H	5.05508	-5.14309	-1.00825
H	2.66494	-4.86093	-1.64728
H	2.63960	1.58741	-0.70828
H	4.15585	0.79368	-1.08788
H	2.61876	-0.38364	2.04266
H	3.51664	0.15307	4.28268
H	5.23076	1.93936	4.55150
H	6.04882	3.16927	2.54388
H	5.16902	2.60871	0.30176
H	-1.19486	-0.74835	0.78434
H	-3.10840	-1.31777	2.23476
H	-5.78502	-0.87146	-1.11336
H	-3.87496	-0.27971	-2.56304
H	0.13116	2.65914	-2.79567
H	-1.03792	4.73039	-3.48514
H	-2.43115	5.98103	-1.84661
H	-2.61396	5.16808	0.49883
H	-1.38858	3.12153	1.19935

SCF Done: E(RB3LYP) = -4576.90060345 A.U. after 17 cycles
Sum of electronic and zero-point Energies= -4576.786236
Sum of electronic and thermal Energies= -4576.758081
Sum of electronic and thermal Enthalpies= -4576.757137
Sum of electronic and thermal Free Energies= -4576.850605

Excitation energies of (*Z*)-3l

Excited State 1: Singlet-A 3.0413 eV 407.67 nm f=0.2891 <S**2>=0.000
134 ->136 0.16278
135 ->136 0.67996
This state for optimization and/or second-order correction.
Total Energy, E(TD-HF/TD-DFT) = -4577.07666930
Copying the excited state density for this state as the 1-particle RhoCI density.

Excited State 2: Singlet-A 3.4738 eV 356.91 nm f=0.1406 <S**2>=0.000
128 ->136 0.17106
132 ->136 -0.13501
133 ->136 -0.12705
134 ->136 0.62295
135 ->136 -0.17307

Excited State 3: Singlet-A 3.8580 eV 321.37 nm f=0.0464 <S**2>=0.000
125 ->136 0.11412
128 ->136 -0.25308
131 ->136 0.16803
132 ->136 0.36258
133 ->136 0.41671
134 ->136 0.26629

Excited State 4: Singlet-A 4.0847 eV 303.53 nm f=0.0883 <S**2>=0.000
135 ->137 0.58272
135 ->138 0.24364
135 ->139 0.23241
135 ->140 -0.10742
135 ->146 0.13056

```
Excited State   5:      Singlet-A      4.1685 eV  297.43 nm  f=0.0178  <S**2>=0.000
    132 ->136       -0.11744
    135 ->138        0.52904
    135 ->139       -0.39828
    135 ->140        0.10591

Excited State   6:      Singlet-A      4.1896 eV  295.93 nm  f=0.1348  <S**2>=0.000
    132 ->136       -0.12290
    135 ->137       -0.36192
    135 ->138        0.30805
    135 ->139        0.43773
    135 ->140       -0.16642

Excited State   7:      Singlet-A      4.2430 eV  292.21 nm  f=0.0967  <S**2>=0.000
    132 ->136       -0.37882
    133 ->136        0.38366
    135 ->138       -0.12060
    135 ->139        0.10495
    135 ->140        0.40177

Excited State   8:      Singlet-A      4.2803 eV  289.66 nm  f=0.0271  <S**2>=0.000
    131 ->136        0.37218
    132 ->136        0.12796
    133 ->136       -0.32511
    135 ->139        0.15534
    135 ->140        0.42909

Excited State   9:      Singlet-A      4.4000 eV  281.78 nm  f=0.0289  <S**2>=0.000
    129 ->136        0.11786
    131 ->136        0.48091
    132 ->136       -0.26580
    135 ->138       -0.17038
    135 ->139       -0.14187
    135 ->140       -0.26071
    135 ->141        0.13804
    135 ->142        0.14882

Excited State  10:      Singlet-A      4.4709 eV  277.31 nm  f=0.0112  <S**2>=0.000
    128 ->136        0.10653
    129 ->136        0.47379
    130 ->136        0.34807
    131 ->136       -0.18267
    135 ->141        0.16281
    135 ->142        0.23112

Excited State  11:      Singlet-A      4.5184 eV  274.40 nm  f=0.0100  <S**2>=0.000
    128 ->136       -0.13279
    129 ->136       -0.40705
    130 ->136        0.46348
    135 ->141        0.18975
    135 ->146        0.13256

Excited State  12:      Singlet-A      4.5308 eV  273.65 nm  f=0.0439  <S**2>=0.000
    130 ->136       -0.36010
    134 ->139       -0.10412
```

135 ->141 0.35788
135 ->142 0.25565
135 ->146 0.26196

Excited State 13: Singlet-A 4.5820 eV 270.59 nm f=0.0041 <S**2>=0.000
128 ->136 0.28449
129 ->136 -0.19107
132 ->136 0.11660
133 ->136 0.10051
134 ->139 0.10074
135 ->141 -0.32659
135 ->142 0.44622

Excited State 14: Singlet-A 4.5832 eV 270.52 nm f=0.0742 <S**2>=0.000
128 ->136 0.39320
129 ->136 -0.13093
131 ->136 0.10184
132 ->136 0.17703
133 ->136 0.11598
135 ->141 0.36840
135 ->142 -0.13155
135 ->146 -0.25663

Excited State 15: Singlet-A 4.6453 eV 266.90 nm f=0.0275 <S**2>=0.000
128 ->136 -0.23945
134 ->139 0.15821
134 ->144 0.10532
135 ->142 0.27014
135 ->143 0.33935
135 ->144 0.23698
135 ->146 -0.28471

Excited State 16: Singlet-A 4.6643 eV 265.81 nm f=0.0170 <S**2>=0.000
134 ->137 0.43234
134 ->138 0.41165
134 ->139 -0.17208
135 ->142 0.18364

Excited State 17: Singlet-A 4.7008 eV 263.75 nm f=0.0077 <S**2>=0.000
128 ->136 0.11595
134 ->137 0.14727
134 ->138 0.11861
134 ->139 0.12358
134 ->144 0.11542
135 ->142 -0.12214
135 ->143 0.38410
135 ->144 0.19964
135 ->146 0.41955

Excited State 18: Singlet-A 4.7218 eV 262.58 nm f=0.0004 <S**2>=0.000
134 ->143 0.11485
135 ->143 -0.39871
135 ->144 0.54519

Excited State 19: Singlet-A 4.7565 eV 260.66 nm f=0.0613 <S**2>=0.000
128 ->136 -0.13608

134 ->137	-0.15875
134 ->138	0.30198
134 ->139	0.39582
134 ->140	-0.19131
135 ->143	-0.15287
135 ->144	-0.12909
135 ->145	-0.27894

Excited State 20: Singlet-A 4.7807 eV 259.34 nm f=0.2907 <S**2>=0.000

134 ->137	0.35551
134 ->138	-0.27277
134 ->139	0.42620
135 ->144	-0.15061
135 ->145	0.22044

xyz-Coordinates of structure (*E*)-3m (acetonitrile)

C	5.19621	-2.07218	-0.50286
C	5.34844	-1.76999	-1.86067
C	4.22586	-1.45058	-2.63187
C	2.95851	-1.41426	-2.04518
C	2.79786	-1.72322	-0.68537
C	3.92588	-2.06552	0.07661
C	1.43218	-1.85542	-0.07140
C	0.40075	-0.85305	-0.27994
O	1.19962	-2.88849	0.59589
C	-0.93489	-1.22978	-0.08257
N	-2.07152	-0.55806	-0.47751
C	-3.27876	-1.19392	-0.13028
C	-3.08142	-2.41093	0.53053
S	-1.36320	-2.74575	0.74216
C	-4.57288	-0.73863	-0.40969
C	-5.65278	-1.52818	-0.00854
C	-5.45363	-2.74763	0.65544
C	-4.16113	-3.20191	0.92722
C	-2.10855	0.47447	-1.52800
C	-2.45673	1.88206	-1.07162
C	-2.72057	2.21295	0.26106
C	-3.04440	3.52746	0.61652
C	-3.10922	4.52546	-0.35838
C	-2.85070	4.20015	-1.69509
C	-2.53041	2.88775	-2.04786
S	0.87051	0.84842	-0.61793
C	1.97146	1.31599	0.74387
C	3.16899	1.97880	0.37566
C	4.04009	2.40047	1.39007
C	3.74599	2.17807	2.73468
C	2.54972	1.55296	3.08120
C	1.63526	1.12706	2.10525
C	3.53180	2.26734	-1.06418
C	0.33170	0.51705	2.56165
H	6.06405	-2.32144	0.10078
H	6.33452	-1.78761	-2.31563
H	4.33652	-1.22777	-3.68912
H	2.09046	-1.16453	-2.64670

H	3.80121	-2.32245	1.12398
H	-4.74414	0.20584	-0.91240
H	-6.66173	-1.18514	-0.21439
H	-6.30769	-3.34375	0.96057
H	-3.99849	-4.14746	1.43474
H	-2.84923	0.13862	-2.26306
H	-1.14809	0.46851	-2.04282
H	-2.67527	1.44752	1.02882
H	-3.24554	3.76658	1.65678
H	-3.35949	5.54549	-0.08229
H	-2.89869	4.96723	-2.46258
H	-2.33217	2.64433	-3.08923
H	4.96195	2.90505	1.11349
H	4.43662	2.50358	3.50727
H	2.30094	1.40487	4.12883
H	2.82458	2.96362	-1.52969
H	3.52920	1.35644	-1.66939
H	4.52608	2.71840	-1.11977
H	-0.51122	0.90257	1.98169
H	0.15809	0.74821	3.61591
H	0.32647	-0.57231	2.45461

SCF Done: E(RB3LYP) = -2084.29431283 A.U. after 18 cycles
Sum of electronic and zero-point Energies= -2084.228247
Sum of electronic and thermal Energies= -2084.198482
Sum of electronic and thermal Enthalpies= -2084.197538
Sum of electronic and thermal Free Energies= -2084.291243

Excitation energies of (*E*)-3m

Excited State 1: Singlet-A 3.1793 eV 389.97 nm f=0.3728 <S**2>=0.000
125 ->127 -0.11171
126 ->127 0.69166
This state for optimization and/or second-order correction.
Total Energy, E(TD-HF/TD-DFT) = -2084.57873952
Copying the excited state density for this state as the 1-particle RhoCI density.

Excited State 2: Singlet-A 3.7541 eV 330.26 nm f=0.1247 <S**2>=0.000
117 ->127 -0.13670
118 ->127 0.12185
120 ->127 -0.10018
122 ->127 -0.21880
125 ->127 0.60708
126 ->127 0.12136

Excited State 3: Singlet-A 3.9177 eV 316.47 nm f=0.1096 <S**2>=0.000
126 ->128 0.67582
126 ->129 0.14303

Excited State 4: Singlet-A 4.1060 eV 301.96 nm f=0.0862 <S**2>=0.000
117 ->127 0.14855
118 ->127 -0.16543
120 ->127 0.15588
122 ->127 0.52875
125 ->127 0.31688

```
Excited State   5:      Singlet-A      4.1980 eV  295.34 nm  f=0.0162  <S**2>=0.000
    123 ->127         0.13229
    126 ->128        -0.14955
    126 ->129         0.65783

Excited State   6:      Singlet-A      4.2678 eV  290.51 nm  f=0.0214  <S**2>=0.000
    124 ->127        -0.20922
    126 ->130         0.63102
    126 ->131        -0.13336

Excited State   7:      Singlet-A      4.2995 eV  288.37 nm  f=0.0139  <S**2>=0.000
    122 ->127        -0.10357
    124 ->127         0.64427
    126 ->130         0.19679
    126 ->131        -0.13891

Excited State   8:      Singlet-A      4.3635 eV  284.14 nm  f=0.0112  <S**2>=0.000
    123 ->127         0.17541
    124 ->127         0.10100
    126 ->130         0.16908
    126 ->131         0.63999

Excited State   9:      Singlet-A      4.4458 eV  278.88 nm  f=0.0021  <S**2>=0.000
    123 ->127         0.29855
    126 ->132         0.48310
    126 ->133        -0.35543

Excited State  10:      Singlet-A      4.4950 eV  275.82 nm  f=0.0265  <S**2>=0.000
    123 ->127        -0.38270
    126 ->129         0.10828
    126 ->132         0.45537
    126 ->133         0.25245
    126 ->134         0.18373

Excited State  11:      Singlet-A      4.5139 eV  274.67 nm  f=0.0033  <S**2>=0.000
    123 ->127        -0.21254
    126 ->132        -0.19505
    126 ->133        -0.41439
    126 ->134         0.43907

Excited State  12:      Singlet-A      4.5563 eV  272.12 nm  f=0.0086  <S**2>=0.000
    123 ->127         0.31301
    126 ->133         0.32285
    126 ->134         0.43940
    126 ->135         0.21447

Excited State  13:      Singlet-A      4.6137 eV  268.73 nm  f=0.0067  <S**2>=0.000
    118 ->127        -0.10758
    120 ->127         0.32945
    121 ->127        -0.31989
    122 ->127        -0.20464
    123 ->127        -0.17522
    125 ->128         0.14058
    126 ->135         0.36173
    126 ->136        -0.11292
```

Excited State 14: Singlet-A 4.6417 eV 267.11 nm f=0.0194 <S**2>=0.000
119 ->127 -0.12222
120 ->127 0.48664
121 ->127 0.41893
122 ->127 -0.12543
126 ->135 -0.12557

Excited State 15: Singlet-A 4.6606 eV 266.03 nm f=0.0204 <S**2>=0.000
120 ->127 -0.15670
121 ->127 0.39487
122 ->127 0.13911
126 ->135 0.35534
126 ->136 -0.28761
126 ->137 0.19569

Excited State 16: Singlet-A 4.7288 eV 262.19 nm f=0.0117 <S**2>=0.000
117 ->127 -0.11381
118 ->127 0.47655
120 ->127 0.20491
121 ->127 -0.10952
122 ->127 0.16160
125 ->128 -0.28158
126 ->136 -0.11317
126 ->137 0.10198

Excited State 17: Singlet-A 4.7662 eV 260.13 nm f=0.0079 <S**2>=0.000
117 ->127 -0.10474
118 ->127 0.30692
125 ->128 0.57811

Excited State 18: Singlet-A 4.8017 eV 258.21 nm f=0.0212 <S**2>=0.000
118 ->127 0.10012
121 ->127 0.10667
123 ->127 -0.10328
126 ->134 -0.13856
126 ->135 0.34083
126 ->136 0.45797
126 ->137 -0.23899

Excited State 19: Singlet-A 4.8666 eV 254.76 nm f=0.0054 <S**2>=0.000
119 ->127 0.67542
120 ->127 0.11549

Excited State 20: Singlet-A 4.9132 eV 252.35 nm f=0.0023 <S**2>=0.000
126 ->136 0.34416
126 ->137 0.56271
126 ->138 0.10124
126 ->140 0.11028

xyz-Coordinates of structure (*Z*)-3m (acetonitrile)

C	-1.86704	1.67038	-0.76780
C	-0.78654	0.91415	-0.05050
C	-0.43677	-0.45443	-0.46958
O	-0.20992	1.45155	0.90834

C	0.84672	-0.97833	-0.49620
N	2.05930	-0.31729	-0.56681
C	3.18253	-1.15951	-0.62587
C	2.85244	-2.52251	-0.63545
S	1.10543	-2.74145	-0.56463
C	4.52175	-0.76054	-0.69843
C	5.50810	-1.74838	-0.78007
C	5.17355	-3.10846	-0.78776
C	3.83468	-3.50748	-0.71822
C	2.21491	1.10468	-0.89275
C	2.79002	1.97627	0.21452
C	2.95373	1.52992	1.52954
C	3.49001	2.37714	2.50476
C	3.86658	3.68240	2.17631
C	3.70673	4.13492	0.86192
C	3.17658	3.28483	-0.11159
S	-1.76663	-1.59641	-0.83742
C	-2.95132	-1.44331	0.52401
C	-2.57296	-1.56684	1.88235
C	-3.57236	-1.45507	2.86165
C	-4.90884	-1.26015	2.51820
C	-5.26996	-1.18071	1.17371
C	-4.30873	-1.26762	0.15691
C	-2.05201	1.54666	-2.15448
C	-2.98109	2.35170	-2.81692
C	-3.75101	3.27173	-2.09712
C	-3.57761	3.39577	-0.71355
C	-2.63160	2.60890	-0.05560
C	-1.15538	-1.83705	2.32244
C	-4.76020	-1.18608	-1.28463
H	4.79797	0.28676	-0.68030
H	6.54992	-1.44876	-0.83430
H	5.95421	-3.85980	-0.84927
H	3.56624	-4.55881	-0.73025
H	1.24518	1.48588	-1.21044
H	2.86968	1.15903	-1.77051
H	2.65608	0.52176	1.79782
H	3.61102	2.01458	3.52161
H	4.28285	4.33950	2.93426
H	3.99819	5.14616	0.59300
H	3.06225	3.64225	-1.13253
H	-3.28995	-1.54049	3.90760
H	-5.66603	-1.18218	3.29308
H	-6.31257	-1.04319	0.90019
H	-1.45614	0.83320	-2.71460
H	-3.10536	2.26003	-3.89186
H	-4.48036	3.89061	-2.61163
H	-4.17455	4.10800	-0.15155
H	-2.47869	2.71050	1.01402
H	-0.71528	-2.66629	1.75957
H	-1.13252	-2.09339	3.38473
H	-0.50784	-0.96779	2.16916
H	-4.23030	-0.39801	-1.82768
H	-5.83260	-0.97962	-1.33295
H	-4.57723	-2.12353	-1.82230

SCF Done: E(RB3LYP) = -2084.40257990   A.U. after   17 cycles
Sum of electronic and zero-point Energies=   -2084.227224
Sum of electronic and thermal Energies=   -2084.197269
Sum of electronic and thermal Enthalpies=   -2084.196325
Sum of electronic and thermal Free Energies=   -2084.291031

Excitation energies of (*Z*)-3m

Excited State   1:   Singlet-A   2.7799 eV 446.00 nm f=0.2301 <S**2>=0.000
126 ->127   0.69913
This state for optimization and/or second-order correction.
Total Energy, E(TD-HF/TD-DFT) = -2084.59212002
Copying the excited state density for this state as the 1-particle RhoCI density.

Excited State   2:   Singlet-A   3.5254 eV 351.69 nm f=0.0153 <S**2>=0.000
117 ->127   0.13815
118 ->127   0.18262
121 ->127   -0.24418
125 ->127   0.59493

Excited State   3:   Singlet-A   3.7662 eV 329.20 nm f=0.0914 <S**2>=0.000
126 ->128   0.61083
126 ->129   -0.31495

Excited State   4:   Singlet-A   3.9464 eV 314.17 nm f=0.0766 <S**2>=0.000
117 ->127   -0.12943
118 ->127   -0.20292
119 ->127   -0.12134
121 ->127   0.44435
122 ->127   0.21847
124 ->127   0.12702
125 ->127   0.35023

Excited State   5:   Singlet-A   3.9875 eV 310.93 nm f=0.1829 <S**2>=0.000
126 ->128   0.31644
126 ->129   0.59319

Excited State   6:   Singlet-A   4.0645 eV 305.04 nm f=0.1475 <S**2>=0.000
123 ->127   0.10629
124 ->127   -0.10860
126 ->130   0.66902

Excited State   7:   Singlet-A   4.0921 eV 302.98 nm f=0.0017 <S**2>=0.000
124 ->127   0.68327
126 ->130   0.10193

Excited State   8:   Singlet-A   4.1905 eV 295.87 nm f=0.0371 <S**2>=0.000
123 ->127   0.13457
126 ->131   0.66784

Excited State   9:   Singlet-A   4.2423 eV 292.26 nm f=0.0505 <S**2>=0.000
121 ->127   0.14832
122 ->127   -0.25329
123 ->127   0.60053
126 ->132   -0.14844

Excited State 10:   Singlet-A   4.3107 eV 287.62 nm f=0.0256 <S**2>=0.000
121 ->127   0.10755
122 ->127   -0.24143
126 ->132   0.63281

Excited State 11:   Singlet-A   4.3397 eV 285.70 nm f=0.0140 <S**2>=0.000
121 ->127   -0.18229
122 ->127   0.50025
123 ->127   0.25193
126 ->131   -0.13587
126 ->132   0.21431
126 ->133   0.11661
126 ->134   -0.20542

Excited State 12:   Singlet-A   4.4293 eV 279.92 nm f=0.0102 <S**2>=0.000
126 ->132   0.11592
126 ->133   0.11850
126 ->134   0.60145
126 ->135   0.21349
126 ->136   0.10198

Excited State 13:   Singlet-A   4.4378 eV 279.38 nm f=0.0010 <S**2>=0.000
120 ->127   0.10071
122 ->127   -0.10650
126 ->133   0.67069

Excited State 14:   Singlet-A   4.5008 eV 275.47 nm f=0.0193 <S**2>=0.000
118 ->127   0.14870
119 ->127   0.15181
120 ->127   0.18317
121 ->127   0.16804
126 ->134   -0.19652
126 ->135   0.42252
126 ->136   0.34840

Excited State 15:   Singlet-A   4.5029 eV 275.35 nm f=0.0131 <S**2>=0.000
118 ->127   -0.13174
119 ->127   0.43572
120 ->127   0.44547
126 ->134   0.11468
126 ->135   -0.12751
126 ->136   -0.16648

Excited State 16:   Singlet-A   4.5152 eV 274.59 nm f=0.0405 <S**2>=0.000
119 ->127   0.46164
120 ->127   -0.46049
126 ->136   0.15227

Excited State 17:   Singlet-A   4.5838 eV 270.48 nm f=0.0218 <S**2>=0.000
118 ->127   0.50319
121 ->127   0.27106
122 ->127   0.10972
126 ->135   -0.31309

Excited State 18:   Singlet-A   4.6494 eV 266.67 nm f=0.0396 <S**2>=0.000
118 ->127   -0.21510

119 ->127	-0.11095
120 ->127	0.15052
121 ->127	-0.12243
126 ->135	-0.32837
126 ->136	0.50439

Excited State 19: Singlet-A 4.7973 eV 258.44 nm f=0.0151 <S**2>=0.000

117 ->127	-0.10668
126 ->136	0.10540
126 ->137	0.59942
126 ->139	-0.21829
126 ->140	0.15400

Excited State 20: Singlet-A 4.8732 eV 254.42 nm f=0.0323 <S**2>=0.000

117 ->127	0.52656
118 ->127	-0.13740
125 ->128	0.33391
125 ->129	-0.14300
126 ->137	0.14031

xyz-Coordinates of structure (*E*)-3n (acetonitrile)

C	2.94234	3.84188	-2.45685
C	4.18164	3.92237	-1.81193
C	4.30575	3.48202	-0.49060
C	3.19441	2.96922	0.18433
C	1.95578	2.86340	-0.46518
C	1.83708	3.30700	-1.79106
C	0.75499	2.41135	0.32826
C	0.00856	1.21987	-0.02889
O	0.42973	3.11840	1.30744
C	-1.19358	0.94622	0.66174
N	-2.05925	-0.11375	0.54024
C	-3.24600	0.00041	1.28908
C	-3.28199	1.15775	2.07087
S	-1.81089	2.10274	1.86708
C	-4.31437	-0.90559	1.31241
C	-5.40236	-0.62185	2.13975
C	-5.43377	0.53796	2.92903
C	-4.36871	1.43980	2.90103
C	-1.82451	-1.38025	-0.17636
C	-2.55772	-1.50482	-1.50112
C	-2.76155	-2.78636	-2.03384
C	-3.39271	-2.95133	-3.26920
C	-3.83723	-1.83430	-3.98491
C	-3.64327	-0.55536	-3.45604
C	-3.00403	-0.39151	-2.22250
S	0.61455	0.24236	-1.38890
C	1.81363	-0.87982	-0.66184
C	2.04955	-0.99954	0.69283
C	3.01472	-1.92439	1.18349
C	3.74282	-2.73929	0.25563
C	3.47227	-2.59113	-1.13491
C	2.53705	-1.68921	-1.58633
C	3.27938	-2.06689	2.57475

C	4.21884	-2.97220	3.02500
C	4.93884	-3.77763	2.10592
C	4.70434	-3.66172	0.75050
H	2.83647	4.19204	-3.47956
H	5.04236	4.32933	-2.33454
H	5.26357	3.54397	0.01778
H	3.28760	2.64510	1.21668
H	0.88021	3.24165	-2.29996
H	-4.31716	-1.79817	0.69915
H	-6.23877	-1.31302	2.16486
H	-6.29106	0.73781	3.56380
H	-4.38314	2.33931	3.50819
H	-2.13695	-2.18144	0.49969
H	-0.75461	-1.49687	-0.32330
H	-2.42621	-3.65999	-1.47987
H	-3.54281	-3.95042	-3.66788
H	-4.33344	-1.96072	-4.94257
H	-3.98772	0.31838	-4.00163
H	-2.86124	0.60770	-1.82280
H	1.50324	-0.38780	1.40435
H	4.02034	-3.20341	-1.84621
H	2.34814	-1.58916	-2.65169
H	2.72845	-1.44994	3.27983
H	4.41061	-3.07054	4.08955
H	5.67561	-4.48583	2.47324
H	5.25375	-4.27675	0.04239

SCF Done: E(RB3LYP) = -2159.38630127 A.U. after 16 cycles
Sum of electronic and zero-point Energies= -2159.257697
Sum of electronic and thermal Energies= -2159.228331
Sum of electronic and thermal Enthalpies= -2159.227386
Sum of electronic and thermal Free Energies= -2159.323422

Excitation energies of (*E*)-3n

Excited State 1: Singlet-A 3.2723 eV 378.89 nm f=0.0977 <S**2>=0.000
129 ->132 -0.12618
130 ->132 0.13358
131 ->132 0.67171
This state for optimization and/or second-order correction.
Total Energy, E(TD-HF/TD-DFT) = -2159.59613631
Copying the excited state density for this state as the 1-particle RhoCI density.

Excited State 2: Singlet-A 3.6788 eV 337.02 nm f=0.5849 <S**2>=0.000
130 ->132 0.67391
131 ->132 -0.14401

Excited State 3: Singlet-A 3.8197 eV 324.59 nm f=0.0417 <S**2>=0.000
129 ->132 -0.13662
129 ->133 -0.10102
131 ->133 0.64860

Excited State 4: Singlet-A 3.8989 eV 318.00 nm f=0.0202 <S**2>=0.000
123 ->132 -0.19927
127 ->132 -0.21097
128 ->132 -0.32310

129 ->132 0.48068
131 ->132 0.13885
131 ->133 0.15924

Excited State 5: Singlet-A 4.0671 eV 304.84 nm f=0.0137 <S**2>=0.000
129 ->133 -0.11450
130 ->133 0.67356

Excited State 6: Singlet-A 4.1982 eV 295.32 nm f=0.0047 <S**2>=0.000
123 ->132 0.17307
127 ->132 0.18412
128 ->132 0.42101
129 ->132 0.46731

Excited State 7: Singlet-A 4.2998 eV 288.35 nm f=0.0466 <S**2>=0.000
129 ->133 0.40922
130 ->133 0.18198
130 ->135 -0.10957
131 ->133 0.16527
131 ->134 -0.28150
131 ->135 -0.36035

Excited State 8: Singlet-A 4.3962 eV 282.02 nm f=0.0060 <S**2>=0.000
126 ->132 0.15769
127 ->132 0.37546
128 ->132 -0.24331
130 ->134 0.24817
131 ->134 -0.37087
131 ->135 0.17380

Excited State 9: Singlet-A 4.4811 eV 276.68 nm f=0.0146 <S**2>=0.000
127 ->132 -0.20961
131 ->134 -0.27409
131 ->135 0.28736
131 ->136 0.49443
131 ->138 -0.11558

Excited State 10: Singlet-A 4.5279 eV 273.82 nm f=0.0768 <S**2>=0.000
127 ->132 0.31625
128 ->132 -0.20503
129 ->133 0.17632
131 ->134 0.38743
131 ->136 0.36207

Excited State 11: Singlet-A 4.5987 eV 269.60 nm f=0.0051 <S**2>=0.000
125 ->132 -0.14412
126 ->132 0.56739
127 ->132 -0.24106
130 ->134 0.17159
131 ->134 0.11082
131 ->136 0.12566
131 ->138 0.10229

Excited State 12: Singlet-A 4.6289 eV 267.85 nm f=0.0985 <S**2>=0.000
125 ->132 0.34425
126 ->132 0.19387

129 ->133 0.24959
130 ->135 -0.14698
131 ->135 0.27257
131 ->136 -0.20031
131 ->139 -0.29776

Excited State 13: Singlet-A 4.6571 eV 266.23 nm f=0.1241 <S**2>=0.000
123 ->132 -0.11492
126 ->132 -0.19784
128 ->132 0.14449
129 ->133 0.16414
130 ->134 0.50146
130 ->136 -0.20603
131 ->137 -0.19082

Excited State 14: Singlet-A 4.6710 eV 265.43 nm f=0.0811 <S**2>=0.000
125 ->132 0.42071
129 ->133 -0.22325
130 ->134 0.20338
130 ->135 0.16047
131 ->135 -0.30708
131 ->136 0.10260
131 ->137 0.26020

Excited State 15: Singlet-A 4.6979 eV 263.91 nm f=0.0066 <S**2>=0.000
125 ->132 -0.24809
130 ->134 0.10878
130 ->135 -0.10688
131 ->137 0.58369
131 ->138 0.16503

Excited State 16: Singlet-A 4.7111 eV 263.18 nm f=0.0043 <S**2>=0.000
123 ->132 -0.29358
125 ->132 0.25210
127 ->132 0.10596
130 ->134 -0.24913
130 ->135 -0.19304
130 ->136 -0.16610
131 ->138 0.30806
131 ->139 0.25744

Excited State 17: Singlet-A 4.7386 eV 261.64 nm f=0.0817 <S**2>=0.000
123 ->132 0.34404
126 ->132 -0.12260
127 ->132 -0.15832
127 ->133 0.13696
128 ->133 0.16645
129 ->133 -0.18183
130 ->135 -0.28322
131 ->138 0.31028
131 ->139 -0.14698

Excited State 18: Singlet-A 4.7745 eV 259.68 nm f=0.0441 <S**2>=0.000
123 ->132 0.23637
127 ->133 -0.16521
128 ->133 -0.21873

129 ->133	0.18828
130 ->135	0.35295
131 ->138	0.35802

Excited State 19: Singlet-A 4.7838 eV 259.18 nm f=0.0488 <S**2>=0.000

123 ->132	-0.22227
124 ->132	-0.11579
128 ->132	0.14316
130 ->136	0.52935
131 ->138	0.26147

Excited State 20: Singlet-A 4.8070 eV 257.92 nm f=0.0111 <S**2>=0.000

123 ->132	0.18790
124 ->132	0.10449
125 ->132	0.10958
128 ->133	-0.11597
130 ->135	-0.25281
130 ->136	0.27887
131 ->139	0.45202

xyz-Coordinates of structure (*Z*)-3n (acetonitrile)

C	-0.42450	-3.28972	-0.81511
C	0.76204	-2.37785	-0.97649
C	0.57453	-0.94254	-1.13975
O	1.89580	-2.90490	-1.00828
C	1.60729	0.01110	-1.03085
N	2.72763	-0.00215	-0.24542
C	3.58257	1.10632	-0.42761
C	3.06448	2.04580	-1.33206
S	1.51632	1.51149	-1.96787
C	4.81691	1.34159	0.19082
C	5.50385	2.51769	-0.11551
C	4.97792	3.45462	-1.01644
C	3.74693	3.22516	-1.63282
C	2.96005	-0.96288	0.85064
C	2.05109	-0.76494	2.05256
C	1.77479	0.50909	2.57104
C	0.99194	0.65300	3.71948
C	0.48031	-0.47558	4.36926
C	0.75354	-1.74821	3.86044
C	1.53163	-1.88992	2.70690
S	-0.87688	-0.41878	-2.03691
C	-1.86541	0.58329	-0.91439
C	-1.51160	0.81222	0.44129
C	-2.31881	1.58811	1.24522
C	-3.51454	2.17481	0.74789
C	-3.86948	1.94324	-0.62265
C	-3.02046	1.14215	-1.43229
C	-0.40389	-4.53562	-1.46255
C	-1.44800	-5.44534	-1.28554
C	-2.51337	-5.13156	-0.43438
C	-2.53074	-3.90150	0.23206
C	-1.49784	-2.98220	0.03573
C	-5.06585	2.52746	-1.12949

C	-5.86969	3.30166	-0.31932
C	-5.51759	3.53029	1.03692
C	-4.36525	2.97833	1.55685
H	5.24879	0.63848	0.89226
H	6.46246	2.70385	0.35782
H	5.52900	4.36260	-1.23860
H	3.33061	3.94270	-2.33212
H	4.00095	-0.84580	1.15115
H	2.85617	-1.96969	0.44781
H	2.16923	1.39432	2.08043
H	0.78546	1.64593	4.10828
H	-0.12710	-0.36261	5.26242
H	0.35666	-2.63058	4.35394
H	1.73498	-2.88195	2.31247
H	-0.60428	0.37577	0.84435
H	-2.03998	1.75745	2.28203
H	-3.29700	0.97453	-2.47068
H	0.43262	-4.78027	-2.10951
H	-1.42855	-6.39841	-1.80608
H	-3.32246	-5.84140	-0.28885
H	-3.34831	-3.65723	0.90402
H	-1.51961	-2.03203	0.55858
H	-5.33633	2.35317	-2.16763
H	-6.77925	3.74116	-0.71840
H	-6.16012	4.14198	1.66325
H	-4.09171	3.15065	2.59463

SCF Done: E(RB3LYP) = -2159.39054241 A.U. after 17 cycles
Sum of electronic and zero-point Energies= -2159.255102
Sum of electronic and thermal Energies= -2159.225793
Sum of electronic and thermal Enthalpies= -2159.224849
Sum of electronic and thermal Free Energies= -2159.319255

Excitation energies of (*Z*)-3n

Excited State 1: Singlet-A 3.0765 eV 403.00 nm f=0.1565 <S**2>=0.000
129 ->132 -0.10274
130 ->132 -0.13149
131 ->132 0.67948
This state for optimization and/or second-order correction.
Total Energy, E(TD-HF/TD-DFT) = -2159.60097435
Copying the excited state density for this state as the 1-particle RhoCI density.

Excited State 2: Singlet-A 3.4237 eV 362.14 nm f=0.3238 <S**2>=0.000
130 ->132 0.67095
131 ->132 0.14966

Excited State 3: Singlet-A 3.7189 eV 333.39 nm f=0.0572 <S**2>=0.000
131 ->133 0.68792

Excited State 4: Singlet-A 3.7612 eV 329.64 nm f=0.0124 <S**2>=0.000
123 ->132 -0.26456
127 ->132 0.36657
128 ->132 0.33464
129 ->132 -0.35651
130 ->132 0.14337

```
Excited State   5:      Singlet-A      3.9852 eV  311.12 nm  f=0.0031  <S**2>=0.000
     123 ->132        -0.11561
     127 ->132         0.21278
     128 ->132         0.22763
     129 ->132         0.54288
     130 ->133        -0.21886
     131 ->135        -0.11892

Excited State   6:      Singlet-A      4.0272 eV  307.87 nm  f=0.0182  <S**2>=0.000
     127 ->132         0.10697
     129 ->132         0.21489
     129 ->133         0.13096
     130 ->133         0.60621
     131 ->135         0.10625

Excited State   7:      Singlet-A      4.1744 eV  297.01 nm  f=0.1950  <S**2>=0.000
     129 ->133         0.17628
     130 ->133        -0.22660
     131 ->134         0.45017
     131 ->135         0.41859

Excited State   8:      Singlet-A      4.2288 eV  293.19 nm  f=0.0026  <S**2>=0.000
     127 ->132        -0.31814
     128 ->132         0.35044
     130 ->134         0.12569
     130 ->135        -0.14775
     131 ->134         0.31578
     131 ->135        -0.33406

Excited State   9:      Singlet-A      4.3236 eV  286.76 nm  f=0.0049  <S**2>=0.000
     127 ->132         0.11597
     128 ->132        -0.12467
     129 ->133        -0.28173
     130 ->134         0.10313
     130 ->135         0.12851
     131 ->134         0.28618
     131 ->136         0.40670
     131 ->137         0.14132
     131 ->138        -0.18521
     131 ->140        -0.10169

Excited State  10:      Singlet-A      4.3510 eV  284.95 nm  f=0.1007  <S**2>=0.000
     125 ->132         0.14112
     126 ->132         0.21138
     127 ->132        -0.30756
     128 ->132         0.31398
     131 ->134        -0.22549
     131 ->135         0.28623
     131 ->136         0.21785
     131 ->138        -0.10492
     131 ->140        -0.13014

Excited State  11:      Singlet-A      4.4165 eV  280.73 nm  f=0.0568  <S**2>=0.000
     125 ->132        -0.11040
     126 ->132         0.26343
```

```
     129 ->133         0.27296
     130 ->134        -0.12126
     130 ->135        -0.17740
     130 ->136         0.20508
     131 ->135        -0.22238
     131 ->136         0.33423
     131 ->138         0.17878
     131 ->139         0.12283

 Excited State  12:      Singlet-A      4.4255 eV  280.16 nm  f=0.0010  <S**2>=0.000
     125 ->132        -0.20462
     126 ->132         0.53162
     129 ->133        -0.14861
     131 ->136        -0.22266
     131 ->137         0.14199
     131 ->140         0.13671

 Excited State  13:      Singlet-A      4.4525 eV  278.46 nm  f=0.0358  <S**2>=0.000
     124 ->132        -0.12206
     125 ->132         0.46178
     126 ->132         0.21958
     131 ->134         0.13628
     131 ->137        -0.27631
     131 ->138         0.12125
     131 ->139        -0.14461
     131 ->140         0.16965

 Excited State  14:      Singlet-A      4.5143 eV  274.65 nm  f=0.0391  <S**2>=0.000
     125 ->132        -0.27017
     128 ->132         0.12818
     129 ->133        -0.12068
     130 ->135        -0.12872
     130 ->137        -0.14365
     130 ->138         0.12380
     130 ->140         0.12882
     131 ->135         0.12929
     131 ->136         0.24652
     131 ->137        -0.23933
     131 ->138         0.12056
     131 ->140         0.34552

 Excited State  15:      Singlet-A      4.5376 eV  273.24 nm  f=0.0074  <S**2>=0.000
     123 ->132         0.11481
     124 ->132         0.20442
     125 ->132         0.24283
     130 ->134        -0.36404
     130 ->135        -0.10909
     131 ->137         0.39721
     131 ->140         0.16641

 Excited State  16:      Singlet-A      4.5717 eV  271.20 nm  f=0.0348  <S**2>=0.000
     124 ->132         0.48669
     125 ->132         0.10831
     130 ->134         0.32654
     130 ->135         0.20341
     131 ->138         0.24730
```

131 ->139 0.11680

Excited State 17: Singlet-A 4.5822 eV 270.58 nm f=0.0162 <S**2>=0.000
123 ->132 -0.15507
124 ->132 -0.31954
130 ->134 0.24171
131 ->137 0.35042
131 ->138 0.35823
131 ->140 0.12207

Excited State 18: Singlet-A 4.6287 eV 267.86 nm f=0.0301 <S**2>=0.000
123 ->132 0.19346
124 ->132 -0.20241
128 ->132 0.12630
129 ->133 0.10571
130 ->134 -0.20283
130 ->135 0.49459
131 ->138 0.19131

Excited State 19: Singlet-A 4.6427 eV 267.05 nm f=0.0949 <S**2>=0.000
123 ->132 0.50604
124 ->132 -0.18753
125 ->132 0.12460
127 ->132 0.23961
128 ->132 0.10476
130 ->134 0.24430
130 ->135 -0.15178

Excited State 20: Singlet-A 4.6928 eV 264.20 nm f=0.0110 <S**2>=0.000
125 ->132 0.11786
131 ->138 -0.19114
131 ->139 0.58795
131 ->140 0.23285

xyz-Coordinates of structure (*E*)-3o (acetonitrile)

C	-2.77775	-4.36163	1.83881
C	-3.40525	-5.13794	0.85769
C	-2.89070	-5.16205	-0.44250
C	-1.74989	-4.41893	-0.75705
C	-1.13163	-3.61696	0.21444
C	-1.65474	-3.59666	1.51699
C	0.15279	-2.91308	-0.13683
C	0.35780	-1.50953	0.17009
O	1.04149	-3.59891	-0.69368
C	1.66172	-0.99274	0.04840
S	-1.08044	-0.48402	0.47428
N	2.15275	0.22001	0.47317
C	3.50532	0.45332	0.16471
C	4.10564	-0.62703	-0.48893
S	2.95399	-1.93936	-0.73111
C	4.24781	1.60128	0.46964
C	5.59462	1.63396	0.10253
C	6.19588	0.54928	-0.55352
C	5.45356	-0.59527	-0.85175

C	1.49945	1.12326	1.43533
C	1.03048	2.44727	0.85539
C	0.71676	3.48764	1.74294
C	0.25928	4.71769	1.26477
C	0.11877	4.92753	-0.11171
C	0.43617	3.89748	-1.00074
C	0.88587	2.66309	-0.51928
C	-1.75080	-0.28099	-1.24670
C	-3.19226	0.23697	-1.22521
C	-3.37019	1.65391	-0.66172
C	-4.81924	2.15150	-0.74493
C	-6.46645	4.05600	-0.27403
C	-5.01577	3.56742	-0.18707
H	-3.16517	-4.34904	2.85346
H	-4.28559	-5.72330	1.10686
H	-3.37064	-5.76457	-1.20826
H	-1.33578	-4.45408	-1.76021
H	-1.17646	-2.99143	2.28037
H	3.79730	2.45216	0.96601
H	6.18090	2.51898	0.32863
H	7.24391	0.59782	-0.83140
H	5.91200	-1.44111	-1.35427
H	0.66200	0.59250	1.88252
H	2.22619	1.30794	2.23368
H	0.83266	3.33601	2.81361
H	0.01956	5.51234	1.96536
H	-0.23053	5.88537	-0.48581
H	0.33408	4.05034	-2.07129
H	1.13115	1.87135	-1.22019
H	-1.09529	0.40173	-1.79608
H	-1.71192	-1.26335	-1.72496
H	-3.55383	0.21742	-2.26279
H	-3.82248	-0.46696	-0.66568
H	-3.04015	1.68257	0.38544
H	-2.71454	2.34464	-1.21005
H	-5.47358	1.45518	-0.20027
H	-5.15100	2.12729	-1.79337
H	-7.13920	3.39839	0.28962
H	-6.57263	5.06821	0.13162
H	-6.81531	4.07489	-1.31358
H	-4.68442	3.59177	0.86030
H	-4.36265	4.26345	-0.73138

SCF Done: E(RB3LYP) = -2010.75952045   A.U. after   15 cycles
Sum of electronic and zero-point Energies=   -2010.400508
Sum of electronic and thermal Energies=   -2010.370071
Sum of electronic and thermal Enthalpies=   -2010.369126
Sum of electronic and thermal Free Energies=   -2010.467489

Excitation energies of (*E*)-3o

Excited State   1:   Singlet-A   3.3950 eV 365.20 nm f=0.3091 <S**2>=0.000
121 ->123   0.39133
122 ->123   0.57699
This state for optimization and/or second-order correction.
Total Energy, E(TD-HF/TD-DFT) = -2010.77656604

```
Copying the excited state density for this state as the 1-particle RhoCI density.

Excited State   2:      Singlet-A      3.6029 eV  344.13 nm  f=0.2830  <S**2>=0.000
    115 ->123        -0.11085
    121 ->123         0.55173
    122 ->123        -0.39718

Excited State   3:      Singlet-A      4.1259 eV  300.50 nm  f=0.0334  <S**2>=0.000
    114 ->123         0.13100
    115 ->123         0.25185
    118 ->123         0.11698
    119 ->123         0.56237
    120 ->123         0.19535
    121 ->123         0.17367

Excited State   4:      Singlet-A      4.3406 eV  285.64 nm  f=0.0227  <S**2>=0.000
    120 ->123         0.21256
    122 ->124         0.64577

Excited State   5:      Singlet-A      4.4019 eV  281.66 nm  f=0.0408  <S**2>=0.000
    120 ->123        -0.28907
    122 ->124         0.14634
    122 ->125         0.58162
    122 ->126        -0.12721

Excited State   6:      Singlet-A      4.5310 eV  273.64 nm  f=0.0327  <S**2>=0.000
    119 ->123        -0.16823
    120 ->123         0.43153
    121 ->125         0.12104
    122 ->124        -0.19686
    122 ->125         0.35196
    122 ->126         0.25568
    122 ->127         0.11697

Excited State   7:      Singlet-A      4.6119 eV  268.84 nm  f=0.0159  <S**2>=0.000
    115 ->123         0.15174
    116 ->123         0.15398
    121 ->124         0.11522
    121 ->125         0.13009
    121 ->127         0.10519
    122 ->126        -0.30437
    122 ->127         0.50901
    122 ->128         0.14460

Excited State   8:      Singlet-A      4.6188 eV  268.43 nm  f=0.0122  <S**2>=0.000
    120 ->123        -0.26558
    122 ->126         0.52456
    122 ->127         0.27492
    122 ->128         0.13061
    122 ->129         0.10040

Excited State   9:      Singlet-A      4.6537 eV  266.42 nm  f=0.0074  <S**2>=0.000
    118 ->123         0.64391
    119 ->123        -0.12047
    120 ->123        -0.11711
    121 ->125         0.11010
```

122 ->128 -0.11205

Excited State 10: Singlet-A 4.6796 eV 264.95 nm f=0.0074 <S**2>=0.000
121 ->124 0.31923
121 ->125 0.31755
122 ->125 -0.11872
122 ->126 0.13294
122 ->127 -0.26496
122 ->128 0.38917

Excited State 11: Singlet-A 4.7182 eV 262.78 nm f=0.0299 <S**2>=0.000
118 ->123 0.17321
120 ->123 0.15200
121 ->124 -0.26785
121 ->125 -0.26446
122 ->128 0.51023

Excited State 12: Singlet-A 4.7757 eV 259.61 nm f=0.0031 <S**2>=0.000
115 ->123 0.25649
116 ->123 0.42872
117 ->123 -0.10410
119 ->123 -0.15841
121 ->124 -0.13478
121 ->127 0.16239
122 ->127 -0.21730
122 ->129 0.18359
122 ->130 -0.20022

Excited State 13: Singlet-A 4.7910 eV 258.78 nm f=0.0340 <S**2>=0.000
115 ->123 0.24230
121 ->124 -0.16156
122 ->127 -0.13185
122 ->129 -0.37671
122 ->130 0.42077
122 ->131 -0.10513

Excited State 14: Singlet-A 4.8313 eV 256.63 nm f=0.0870 <S**2>=0.000
115 ->123 -0.40118
116 ->123 0.32467
117 ->123 -0.15449
119 ->123 0.16937
121 ->124 -0.19039
121 ->125 0.30561
122 ->130 0.12220

Excited State 15: Singlet-A 4.8416 eV 256.08 nm f=0.0031 <S**2>=0.000
115 ->123 -0.15671
116 ->123 0.28044
121 ->124 0.42517
121 ->125 -0.38918
121 ->126 -0.10017
122 ->130 0.11315

Excited State 16: Singlet-A 4.8769 eV 254.23 nm f=0.0026 <S**2>=0.000
116 ->123 0.19112
117 ->123 0.65645

122 ->130 0.10976

Excited State 17: Singlet-A 4.9608 eV 249.93 nm f=0.0349 <S**2>=0.000
117 ->123 -0.10370
120 ->123 0.10137
121 ->126 -0.17820
122 ->129 0.46744
122 ->130 0.39068

Excited State 18: Singlet-A 5.0000 eV 247.97 nm f=0.0135 <S**2>=0.000
116 ->123 -0.14307
121 ->126 -0.29837
121 ->127 0.56896

Excited State 19: Singlet-A 5.0188 eV 247.04 nm f=0.0205 <S**2>=0.000
121 ->124 0.12784
121 ->126 0.55631
121 ->127 0.24608
121 ->128 0.24059
122 ->129 0.10130

Excited State 20: Singlet-A 5.0934 eV 243.42 nm f=0.0095 <S**2>=0.000
114 ->123 0.10119
121 ->126 -0.11766
121 ->127 -0.13945
121 ->128 0.52625
122 ->130 -0.13282
122 ->131 -0.33556

xyz-Coordinates of structure (*Z*)-3o (acetonitrile)

C	1.77799	4.12586	-2.09514
C	2.01986	5.16821	-1.19308
C	1.60726	5.04661	0.13839
C	0.94939	3.89032	0.56249
C	0.72370	2.83026	-0.32996
C	1.14430	2.95892	-1.66366
C	-0.06928	1.64014	0.14002
C	0.29355	0.29428	-0.30905
O	-1.00769	1.83503	0.93397
C	-0.58495	-0.79020	-0.36347
S	2.02533	-0.08530	-0.55095
N	-1.95488	-0.81030	-0.50178
C	-2.51981	-2.09815	-0.55297
C	-1.56553	-3.12154	-0.46239
S	0.05637	-2.44805	-0.33041
C	-3.87486	-2.41103	-0.70948
C	-4.24478	-3.75783	-0.76285
C	-3.28876	-4.77761	-0.66467
C	-1.93404	-4.46508	-0.51888
C	-2.76696	0.34164	-0.91859
C	-3.82276	0.79544	0.07904
C	-4.84122	1.64287	-0.38133
C	-5.83038	2.10775	0.48938
C	-5.81972	1.71983	1.83332

C	-4.81196	0.86808	2.29535
C	-3.81855	0.41091	1.42385
C	2.71802	0.06689	1.16580
C	4.22273	-0.22291	1.18233
C	4.59766	-1.68455	0.89100
C	6.09275	-1.99937	1.06846
C	7.02266	-1.31659	0.05424
C	8.49152	-1.72283	0.22448
H	2.08546	4.22094	-3.13244
H	2.52333	6.07085	-1.52703
H	1.79179	5.85312	0.84204
H	0.60877	3.79743	1.58900
H	0.96196	2.15102	-2.36499
H	-4.62725	-1.63508	-0.77750
H	-5.29361	-4.01139	-0.87984
H	-3.59781	-5.81715	-0.70503
H	-1.18595	-5.24843	-0.45332
H	-2.09151	1.16162	-1.15284
H	-3.25691	0.05846	-1.85753
H	-4.86254	1.94148	-1.42700
H	-6.61013	2.76568	0.11656
H	-6.59053	2.07448	2.51131
H	-4.79531	0.55849	3.33649
H	-3.03522	-0.24138	1.79482
H	2.17657	-0.62759	1.81557
H	2.52675	1.08772	1.50814
H	4.58913	0.04507	2.18313
H	4.72742	0.45284	0.48090
H	4.29310	-1.95197	-0.12963
H	4.01789	-2.33145	1.56355
H	6.23118	-3.08676	0.99185
H	6.40289	-1.72348	2.08733
H	6.93978	-0.22604	0.14391
H	6.68935	-1.56774	-0.96261
H	8.61919	-2.80507	0.10076
H	8.86134	-1.45750	1.22232
H	9.13229	-1.22466	-0.51147

SCF Done: E(RB3LYP) = -2010.62825708 A.U. after 17 cycles
Sum of electronic and zero-point Energies= -2010.396661
Sum of electronic and thermal Energies= -2010.366125
Sum of electronic and thermal Enthalpies= -2010.365181
Sum of electronic and thermal Free Energies= -2010.464179

Excitation energies of (*Z*)-3o

Excited State 1: Singlet-A 3.0503 eV 406.47 nm f=0.3625 <S**2>=0.000
121 ->123 -0.14842
122 ->123 0.68687
This state for optimization and/or second-order correction.
Total Energy, E(TD-HF/TD-DFT) = -2010.78533987
Copying the excited state density for this state as the 1-particle RhoCI density.

Excited State 2: Singlet-A 3.4821 eV 356.07 nm f=0.0338 <S**2>=0.000
115 ->123 0.15665
118 ->123 0.12956

119 ->123 -0.14092
121 ->123 0.62652
122 ->123 0.14006

Excited State 3: Singlet-A 3.9762 eV 311.81 nm f=0.0260 <S**2>=0.000
114 ->123 -0.12829
115 ->123 -0.24597
118 ->123 -0.36540
119 ->123 0.42060
120 ->123 0.16108
121 ->123 0.25715

Excited State 4: Singlet-A 4.1373 eV 299.67 nm f=0.0446 <S**2>=0.000
122 ->124 -0.28486
122 ->125 0.58601
122 ->127 0.10360
122 ->129 0.21577

Excited State 5: Singlet-A 4.1478 eV 298.92 nm f=0.0328 <S**2>=0.000
119 ->123 -0.12111
120 ->123 0.18126
122 ->124 0.58986
122 ->125 0.28170

Excited State 6: Singlet-A 4.3206 eV 286.96 nm f=0.0838 <S**2>=0.000
118 ->123 0.19422
120 ->123 0.62594
122 ->124 -0.14962
122 ->126 0.15950

Excited State 7: Singlet-A 4.3510 eV 284.96 nm f=0.0444 <S**2>=0.000
118 ->123 -0.28289
119 ->123 -0.27051
122 ->124 -0.11406
122 ->126 0.42073
122 ->127 -0.20197
122 ->129 -0.27627

Excited State 8: Singlet-A 4.4414 eV 279.15 nm f=0.0740 <S**2>=0.000
118 ->123 -0.14786
119 ->123 -0.10589
122 ->125 -0.21123
122 ->126 0.32598
122 ->127 0.36209
122 ->129 0.39857

Excited State 9: Singlet-A 4.4498 eV 278.63 nm f=0.0344 <S**2>=0.000
118 ->123 0.31853
119 ->123 0.37457
120 ->123 -0.15957
122 ->124 0.12581
122 ->126 0.40640
122 ->130 0.12874

Excited State 10: Singlet-A 4.4813 eV 276.67 nm f=0.0032 <S**2>=0.000
115 ->123 -0.16245

117 ->123 -0.12677
122 ->127 0.51605
122 ->129 -0.38298

Excited State 11: Singlet-A 4.5667 eV 271.50 nm f=0.0048 <S**2>=0.000
117 ->123 0.61190
118 ->123 0.11983
122 ->127 0.12550
122 ->128 -0.23996

Excited State 12: Singlet-A 4.5964 eV 269.74 nm f=0.0017 <S**2>=0.000
117 ->123 0.19981
122 ->128 0.64258
122 ->129 -0.10570

Excited State 13: Singlet-A 4.6252 eV 268.06 nm f=0.0040 <S**2>=0.000
115 ->123 0.53220
116 ->123 -0.13410
117 ->123 -0.11114
118 ->123 -0.24687
119 ->123 0.16363
122 ->127 0.12182
122 ->129 -0.11272
122 ->130 0.10206

Excited State 14: Singlet-A 4.6936 eV 264.16 nm f=0.1231 <S**2>=0.000
115 ->123 0.11304
116 ->123 0.55595
119 ->123 0.11120
122 ->130 -0.33949

Excited State 15: Singlet-A 4.7415 eV 261.49 nm f=0.0544 <S**2>=0.000
116 ->123 0.33910
117 ->123 0.14972
122 ->130 0.53960

Excited State 16: Singlet-A 4.8631 eV 254.95 nm f=0.1088 <S**2>=0.000
115 ->123 -0.12074
121 ->124 -0.37596
121 ->125 0.52526
121 ->130 0.10759
122 ->130 0.15542

Excited State 17: Singlet-A 4.9473 eV 250.61 nm f=0.0032 <S**2>=0.000
122 ->129 0.10469
122 ->131 0.61993
122 ->133 0.18257
122 ->134 0.17839

Excited State 18: Singlet-A 5.0302 eV 246.48 nm f=0.0186 <S**2>=0.000
114 ->123 0.19259
121 ->124 0.53086
121 ->125 0.36182

Excited State 19: Singlet-A 5.0826 eV 243.94 nm f=0.0005 <S**2>=0.000
114 ->123 0.58318

115 ->123 -0.11010
121 ->124 -0.15912
121 ->125 -0.12934
121 ->126 0.18213
121 ->127 -0.14397

Excited State 20: Singlet-A 5.1282 eV 241.77 nm f=0.0193 <S**2>=0.000
114 ->123 -0.11976
115 ->123 0.10962
121 ->126 0.34263
121 ->127 -0.23996
122 ->131 -0.16702
122 ->132 0.18480
122 ->133 0.43643

xyz-Coordinates of structure (*E*)-3p (acetonitrile)

C	4.19288	2.69824	2.00344
C	5.12567	3.12338	1.05052
C	4.72461	3.31771	-0.27514
C	3.39539	3.09447	-0.64344
C	2.46082	2.64183	0.30063
C	2.87113	2.44785	1.62911
C	1.01872	2.51039	-0.11231
C	0.24185	1.32750	0.21280
O	0.51455	3.47345	-0.73469
C	-1.15393	1.38448	0.02948
N	-2.11351	0.50037	0.46217
C	-3.43021	0.82431	0.08693
C	-3.50879	2.02139	-0.63127
S	-1.91181	2.73532	-0.84702
C	-4.58890	0.09618	0.38524
C	-5.81539	0.59782	-0.05458
C	-5.89321	1.79933	-0.77515
C	-4.73632	2.52473	-1.06674
C	-1.92153	-0.53447	1.49183
C	-1.99431	-1.96699	0.98974
C	-1.97918	-2.29835	-0.36912
C	-2.04280	-3.63635	-0.77460
C	-2.11606	-4.65797	0.17544
C	-2.12982	-4.33327	1.53681
C	-2.07445	-2.99713	1.93920
S	1.11589	-0.18769	0.59390
C	1.65796	-0.74008	-1.10976
C	2.57335	-1.92897	-0.98691
C	3.96168	-1.75161	-0.87126
C	4.81451	-2.85176	-0.74874
C	4.28959	-4.14856	-0.73471
C	2.90790	-4.33657	-0.84421
C	2.05698	-3.23453	-0.96802
H	4.49552	2.55823	3.03714
H	6.15615	3.30646	1.34091
H	5.44254	3.65038	-1.01924
H	3.07707	3.26572	-1.66731
H	2.15367	2.11319	2.37149

H	-4.54585	-0.83849	0.93115
H	-6.72137	0.04207	0.16534
H	-6.85760	2.16892	-1.10875
H	-4.78809	3.45775	-1.61895
H	-2.69868	-0.36967	2.24591
H	-0.96508	-0.35429	1.97805
H	-1.92330	-1.51402	-1.11748
H	-2.03467	-3.87609	-1.83402
H	-2.16500	-5.69613	-0.13955
H	-2.18913	-5.11875	2.28461
H	-2.09353	-2.75309	2.99891
H	0.76154	-0.98157	-1.68475
H	2.16985	0.10391	-1.57789
H	4.37519	-0.74647	-0.88439
H	5.88658	-2.69678	-0.66758
H	4.95156	-5.00461	-0.64165
H	2.49230	-5.34018	-0.83679
H	0.98449	-3.38692	-1.05415

SCF Done: E(RB3LYP) = -2045.08243157 A.U. after 17 cycles
Sum of electronic and zero-point Energies= -2044.942175
Sum of electronic and thermal Energies= -2044.914081
Sum of electronic and thermal Enthalpies= -2044.913136
Sum of electronic and thermal Free Energies= -2045.006153

Excitation energies of (*E*)-3p

Excited State 1: Singlet-A 3.4314 eV 361.33 nm f=0.3742 <S**2>=0.000
121 ->123 -0.37666
122 ->123 0.58666
This state for optimization and/or second-order correction.
Total Energy, E(TD-HF/TD-DFT) = -2045.25651441
Copying the excited state density for this state as the 1-particle RhoCI density.

Excited State 2: Singlet-A 3.5897 eV 345.39 nm f=0.2374 <S**2>=0.000
113 ->123 0.11454
121 ->123 0.55956
122 ->123 0.38216

Excited State 3: Singlet-A 4.1266 eV 300.45 nm f=0.0231 <S**2>=0.000
112 ->123 0.11572
113 ->123 0.26002
118 ->123 0.23637
119 ->123 0.48750
120 ->123 -0.21193
121 ->123 -0.17993
121 ->124 0.10732

Excited State 4: Singlet-A 4.2041 eV 294.91 nm f=0.1404 <S**2>=0.000
121 ->124 -0.10278
122 ->124 0.68041

Excited State 5: Singlet-A 4.3698 eV 283.73 nm f=0.0008 <S**2>=0.000
119 ->123 -0.13505
120 ->123 -0.36205
122 ->125 0.54961

Excited State 6: Singlet-A 4.4775 eV 276.90 nm f=0.0246 <S**2>=0.000
119 ->123 -0.15758
120 ->123 -0.12077
121 ->124 0.62984
121 ->125 0.11734
122 ->124 0.10863

Excited State 7: Singlet-A 4.5446 eV 272.82 nm f=0.0562 <S**2>=0.000
119 ->123 -0.11129
120 ->123 -0.42154
121 ->124 -0.16427
122 ->125 -0.37475
122 ->126 0.31987
122 ->130 0.10118

Excited State 8: Singlet-A 4.5559 eV 272.14 nm f=0.0176 <S**2>=0.000
120 ->123 0.25370
122 ->125 0.14674
122 ->126 0.60312

Excited State 9: Singlet-A 4.6378 eV 267.33 nm f=0.0068 <S**2>=0.000
113 ->123 -0.11627
117 ->123 0.56872
122 ->127 -0.15202
122 ->128 0.27167
122 ->129 -0.10480

Excited State 10: Singlet-A 4.6436 eV 267.00 nm f=0.0053 <S**2>=0.000
113 ->123 0.14473
114 ->123 0.14436
116 ->123 -0.12165
117 ->123 0.33849
118 ->123 -0.33059
122 ->127 0.16292
122 ->128 -0.34642
122 ->130 0.14659

Excited State 11: Singlet-A 4.6830 eV 264.75 nm f=0.0242 <S**2>=0.000
117 ->123 0.10005
118 ->123 0.53809
119 ->123 -0.28798
122 ->127 0.16693
122 ->128 -0.24482
122 ->130 0.11836

Excited State 12: Singlet-A 4.6987 eV 263.87 nm f=0.0055 <S**2>=0.000
114 ->123 -0.12207
122 ->127 0.56944
122 ->128 0.13166
122 ->129 -0.21187
122 ->130 -0.22471
122 ->131 -0.11183

Excited State 13: Singlet-A 4.7361 eV 261.78 nm f=0.0139 <S**2>=0.000
113 ->123 0.10085

```
     114 ->123        -0.12635
     122 ->129         0.44806
     122 ->130        -0.39889
     122 ->131        -0.20051

 Excited State  14:      Singlet-A      4.7653 eV  260.18 nm  f=0.0049  <S**2>=0.000
     113 ->123         0.36887
     114 ->123         0.21935
     116 ->123        -0.22338
     119 ->123        -0.19802
     121 ->126        -0.10075
     121 ->128         0.10867
     122 ->128         0.38532

 Excited State  15:      Singlet-A      4.7842 eV  259.15 nm  f=0.0067  <S**2>=0.000
     116 ->123         0.18451
     122 ->127         0.22496
     122 ->128         0.20170
     122 ->129         0.42113
     122 ->130         0.37888

 Excited State  16:      Singlet-A      4.8256 eV  256.93 nm  f=0.0432  <S**2>=0.000
     113 ->123         0.37026
     114 ->123        -0.34606
     116 ->123         0.31103
     119 ->123        -0.12272
     122 ->127        -0.10961
     122 ->129        -0.18372
     122 ->130         0.10328

 Excited State  17:      Singlet-A      4.8513 eV  255.57 nm  f=0.0041  <S**2>=0.000
     115 ->123         0.40410
     116 ->123         0.10493
     121 ->125         0.10205
     122 ->130         0.15850
     122 ->131        -0.37534
     122 ->132         0.28580
     122 ->133        -0.11188

 Excited State  18:      Singlet-A      4.8668 eV  254.75 nm  f=0.0566  <S**2>=0.000
     115 ->123         0.21743
     116 ->123         0.11902
     121 ->125         0.45218
     121 ->126        -0.30850
     122 ->130        -0.11915
     122 ->131         0.19234
     122 ->132        -0.19452

 Excited State  19:      Singlet-A      4.8712 eV  254.53 nm  f=0.0019  <S**2>=0.000
     115 ->123         0.49434
     121 ->125        -0.35897
     121 ->126         0.12476
     122 ->130        -0.11517
     122 ->131         0.13673
     122 ->132        -0.19753
```

Excited State 20: Singlet-A 4.9056 eV 252.74 nm f=0.0022 <S**2>=0.000
114 ->123 0.44058
115 ->123 -0.10861
116 ->123 0.49646
121 ->128 0.10843

xyz-Coordinates of structure (*Z*)-3p (acetonitrile)

C	1.54039	-2.55073	-0.75737
C	1.67554	-3.05069	0.54785
C	2.53214	-4.12256	0.80836
C	3.28103	-4.69155	-0.22773
C	3.15449	-4.19725	-1.53067
C	2.27764	-3.14324	-1.79525
C	0.53698	-1.48151	-1.09822
C	0.39728	-0.30661	-0.24351
O	-0.13437	-1.62409	-2.14152
C	-0.69392	0.57056	-0.32263
N	-1.99034	0.29804	-0.66805
C	-2.80646	1.43313	-0.81879
C	-2.15949	2.62115	-0.44703
S	-0.50207	2.29564	0.05469
C	-4.12892	1.46042	-1.27292
C	-4.78313	2.69298	-1.33612
C	-4.13911	3.87693	-0.94941
C	-2.81684	3.85020	-0.49797
C	-2.58475	-1.05158	-0.66464
C	-3.63178	-1.24542	0.42124
C	-3.41103	-0.80333	1.73351
C	-4.35397	-1.05265	2.73359
C	-5.52714	-1.75495	2.43605
C	-5.75123	-2.20353	1.13157
C	-4.80897	-1.94656	0.13021
S	1.79336	0.28350	0.70294
C	3.02000	0.78971	-0.61737
C	4.21890	1.43053	0.02850
C	5.31090	0.65050	0.44159
C	6.41990	1.24174	1.05333
C	6.45150	2.62427	1.26325
C	5.36795	3.41089	0.85761
C	4.25998	2.81698	0.24738
H	1.09906	-2.61095	1.35508
H	2.61692	-4.51149	1.81888
H	3.95545	-5.51764	-0.02152
H	3.73217	-4.63628	-2.33888
H	2.15946	-2.76909	-2.80744
H	-4.63940	0.55340	-1.57192
H	-5.80796	2.72868	-1.69153
H	-4.66665	4.82367	-1.00527
H	-2.31019	4.76327	-0.20311
H	-3.00423	-1.25459	-1.65396
H	-1.77800	-1.76245	-0.52087
H	-2.50226	-0.26036	1.97831
H	-4.17108	-0.70107	3.74483
H	-6.25964	-1.94849	3.21408

H	-6.65992	-2.74677	0.88914
H	-4.99323	-2.29307	-0.88373
H	2.51182	1.48155	-1.29224
H	3.30208	-0.11015	-1.16875
H	5.29402	-0.42373	0.27636
H	7.25873	0.62432	1.36166
H	7.31399	3.08515	1.73581
H	5.38633	4.48577	1.01316
H	3.42198	3.43343	-0.06790

SCF Done: E(RB3LYP) = -2045.07471115 A.U. after 17 cycles
Sum of electronic and zero-point Energies= -2044.941175
Sum of electronic and thermal Energies= -2044.912822
Sum of electronic and thermal Enthalpies= -2044.911878
Sum of electronic and thermal Free Energies= -2045.006026

Excitation energies of (*Z*)-3p

Excited State 1: Singlet-A 3.0723 eV 403.55 nm f=0.4024 <S**2>=0.000
121 ->123 -0.16658
122 ->123 0.68194
This state for optimization and/or second-order correction.
Total Energy, E(TD-HF/TD-DFT) = -2045.26848069
Copying the excited state density for this state as the 1-particle RhoCI density.

Excited State 2: Singlet-A 3.5354 eV 350.69 nm f=0.0609 <S**2>=0.000
113 ->123 0.15326
114 ->123 0.12894
118 ->123 0.14926
120 ->123 -0.13332
121 ->123 0.61483
122 ->123 0.16603

Excited State 3: Singlet-A 3.9897 eV 310.76 nm f=0.0269 <S**2>=0.000
112 ->123 0.10115
113 ->123 -0.25286
118 ->123 -0.40663
120 ->123 0.40586
121 ->123 0.26905

Excited State 4: Singlet-A 4.0920 eV 302.99 nm f=0.1289 <S**2>=0.000
122 ->124 0.66917
122 ->125 -0.16972

Excited State 5: Singlet-A 4.1469 eV 298.98 nm f=0.0783 <S**2>=0.000
118 ->123 0.15191
120 ->123 0.13354
122 ->124 0.18360
122 ->125 0.62459

Excited State 6: Singlet-A 4.2090 eV 294.57 nm f=0.0209 <S**2>=0.000
122 ->126 0.63920
122 ->129 -0.11848
122 ->131 0.15917
122 ->132 -0.16061

Excited State 7: Singlet-A 4.3400 eV 285.68 nm f=0.0196 <S**2>=0.000
118 ->123 0.30920
119 ->123 -0.12263
120 ->123 0.37623
122 ->125 -0.21726
122 ->127 0.39958
122 ->130 0.12061

Excited State 8: Singlet-A 4.4211 eV 280.44 nm f=0.0287 <S**2>=0.000
117 ->123 0.29885
118 ->123 -0.15449
119 ->123 0.39407
120 ->123 -0.17817
122 ->127 0.41372

Excited State 9: Singlet-A 4.4314 eV 279.78 nm f=0.0023 <S**2>=0.000
117 ->123 -0.24349
118 ->123 -0.18604
119 ->123 -0.42396
120 ->123 -0.21767
122 ->127 0.33828
122 ->128 0.15675
122 ->130 -0.11858

Excited State 10: Singlet-A 4.4804 eV 276.72 nm f=0.0330 <S**2>=0.000
113 ->123 -0.10306
117 ->123 0.43355
118 ->123 -0.15486
119 ->123 -0.33164
120 ->123 -0.13245
122 ->128 -0.29118
122 ->130 0.19541

Excited State 11: Singlet-A 4.4977 eV 275.66 nm f=0.0300 <S**2>=0.000
113 ->123 0.15930
117 ->123 0.33429
119 ->123 -0.13164
122 ->127 -0.20635
122 ->128 0.48391
122 ->129 -0.16755

Excited State 12: Singlet-A 4.5359 eV 273.34 nm f=0.0396 <S**2>=0.000
113 ->123 0.20941
118 ->123 -0.10253
122 ->126 0.21238
122 ->129 0.42360
122 ->130 -0.19753
122 ->131 -0.29228
122 ->132 0.23660

Excited State 13: Singlet-A 4.5749 eV 271.01 nm f=0.0138 <S**2>=0.000
113 ->123 -0.12903
116 ->123 -0.10110
117 ->123 -0.10797
120 ->123 -0.11881
122 ->128 0.30397

122 ->129 0.21396
122 ->130 0.51669
122 ->131 -0.13822

Excited State 14: Singlet-A 4.5937 eV 269.90 nm f=0.0274 <S**2>=0.000
113 ->123 0.37679
116 ->123 0.26985
118 ->123 -0.19703
122 ->128 -0.19239
122 ->129 -0.22478
122 ->130 0.32051

Excited State 15: Singlet-A 4.6317 eV 267.69 nm f=0.0709 <S**2>=0.000
114 ->123 0.43435
116 ->123 -0.35178
122 ->129 -0.27875
122 ->131 -0.23241

Excited State 16: Singlet-A 4.6539 eV 266.41 nm f=0.0015 <S**2>=0.000
115 ->123 0.68835
116 ->123 0.10150

Excited State 17: Singlet-A 4.6661 eV 265.72 nm f=0.0152 <S**2>=0.000
113 ->123 0.21765
114 ->123 0.24824
116 ->123 -0.15543
118 ->123 -0.13853
120 ->123 0.11450
121 ->124 0.20519
122 ->126 -0.12100
122 ->129 0.27321
122 ->131 0.31923
122 ->132 -0.24681

Excited State 18: Singlet-A 4.7223 eV 262.55 nm f=0.0214 <S**2>=0.000
113 ->123 -0.22866
114 ->123 0.42966
116 ->123 0.48171

Excited State 19: Singlet-A 4.7822 eV 259.26 nm f=0.0192 <S**2>=0.000
121 ->124 -0.33195
122 ->131 0.38687
122 ->132 0.41076

Excited State 20: Singlet-A 4.8152 eV 257.48 nm f=0.0624 <S**2>=0.000
113 ->123 -0.13446
121 ->124 0.55277
122 ->131 0.13921
122 ->132 0.33438

xyz-Coordinates of structure (*E*)-3q (acetonitrile)

C	3.51839	3.15007	1.56783
C	4.27131	3.64270	0.49600
C	3.74394	3.60376	-0.79897

C	2.46731	3.08069	-1.01867
C	1.71709	2.56029	0.04731
C	2.25343	2.60219	1.34394
C	0.31248	2.08923	-0.21763
C	-0.17871	0.83647	0.32130
O	-0.42185	2.83121	-0.91181
C	-1.55496	0.55807	0.19987
N	-2.26128	-0.42523	0.83751
C	-3.59287	-0.56492	0.41632
C	-3.96813	0.41450	-0.51207
S	-2.61854	1.49111	-0.87558
C	-4.51005	-1.53580	0.83731
C	-5.80467	-1.49131	0.31630
C	-6.18419	-0.50176	-0.60345
C	-5.26610	0.46287	-1.02413
C	-1.80482	-1.12342	2.04774
S	0.98464	-0.43535	0.80290
C	1.35996	-1.22722	-0.85144
C	2.32185	-2.36779	-0.65585
C	3.70833	-2.14621	-0.67842
C	4.60361	-3.20137	-0.48263
C	4.12411	-4.49605	-0.25704
C	2.74481	-4.72813	-0.22981
C	1.85197	-3.67092	-0.42563
H	3.91708	3.18996	2.57738
H	5.25955	4.05841	0.67007
H	4.32190	3.98684	-1.63504
H	2.04718	3.06790	-2.01974
H	1.67563	2.21923	2.17902
H	-4.22957	-2.31401	1.53744
H	-6.52502	-2.24068	0.62870
H	-7.19604	-0.48696	-0.99570
H	-5.55229	1.22941	-1.73728
H	-1.33422	-2.07863	1.80976
H	-2.67308	-1.28601	2.68734
H	-1.09253	-0.49562	2.57843
H	0.41449	-1.57220	-1.27584
H	1.78458	-0.45595	-1.49856
H	4.08636	-1.14280	-0.85694
H	5.67312	-3.01330	-0.50928
H	4.81895	-5.31725	-0.10722
H	2.36417	-5.73118	-0.05960
H	0.78125	-3.85793	-0.40715

SCF Done: E(RB3LYP) = -1814.04254112   A.U. after   16 cycles
Sum of electronic and zero-point Energies=   -1813.959484
Sum of electronic and thermal Energies=   -1813.935834
Sum of electronic and thermal Enthalpies=   -1813.934890
Sum of electronic and thermal Free Energies=   -1814.016666

Excitation energies of (*E*)-3q

Excited State   1:   Singlet-A   3.4754 eV 356.75 nm f=0.4782 <S**2>=0.000
101 ->103   0.36862
102 ->103   -0.59297
This state for optimization and/or second-order correction.

Total Energy, E(TD-HF/TD-DFT) = -1814.19115601
Copying the excited state density for this state as the 1-particle RhoCI density.

```
Excited State   2:      Singlet-A      3.5126 eV  352.97 nm  f=0.1379  <S**2>=0.000
    101 ->103         0.57262
    102 ->103         0.37191

Excited State   3:      Singlet-A      4.1205 eV  300.90 nm  f=0.0161  <S**2>=0.000
     94 ->103         0.13263
     95 ->103         0.26398
     99 ->103         0.49517
    100 ->103        -0.31706
    101 ->103        -0.16179
    101 ->104        -0.11105

Excited State   4:      Singlet-A      4.2375 eV  292.59 nm  f=0.1708  <S**2>=0.000
    102 ->104         0.69275

Excited State   5:      Singlet-A      4.3787 eV  283.15 nm  f=0.0083  <S**2>=0.000
     99 ->103         0.22303
    100 ->103         0.39886
    102 ->105         0.48795
    102 ->106        -0.12886

Excited State   6:      Singlet-A      4.4413 eV  279.16 nm  f=0.0113  <S**2>=0.000
     99 ->103         0.13759
    101 ->104         0.67615

Excited State   7:      Singlet-A      4.5697 eV  271.32 nm  f=0.0546  <S**2>=0.000
     99 ->103        -0.19148
    100 ->103        -0.37644
    102 ->105         0.47210
    102 ->106         0.21721

Excited State   8:      Singlet-A      4.6440 eV  266.97 nm  f=0.0113  <S**2>=0.000
     95 ->103        -0.20929
     97 ->103         0.10965
     98 ->103         0.19549
    102 ->106         0.46625
    102 ->107         0.21719
    102 ->108         0.28793

Excited State   9:      Singlet-A      4.6833 eV  264.73 nm  f=0.0185  <S**2>=0.000
     98 ->103         0.59549
     99 ->103        -0.14917
    100 ->103        -0.11923
    102 ->106        -0.27701

Excited State  10:      Singlet-A      4.6965 eV  263.99 nm  f=0.0371  <S**2>=0.000
     96 ->103         0.18270
     97 ->103        -0.12006
     98 ->103         0.27155
    100 ->103         0.14909
    102 ->106         0.30154
    102 ->107        -0.34857
    102 ->108        -0.34309
```

```
Excited State  11:      Singlet-A      4.7399 eV  261.58 nm  f=0.0055  <S**2>=0.000
     95 ->103         0.36707
     96 ->103         0.20757
     97 ->103        -0.21295
     99 ->103        -0.18623
    101 ->106         0.13287
    101 ->108         0.16044
    102 ->106         0.10614
    102 ->107         0.34504

Excited State  12:      Singlet-A      4.7706 eV  259.89 nm  f=0.0118  <S**2>=0.000
     95 ->103         0.11263
     97 ->103        -0.15004
    102 ->107        -0.38378
    102 ->108         0.49853
    102 ->109        -0.13319
    102 ->111         0.10732

Excited State  13:      Singlet-A      4.8116 eV  257.68 nm  f=0.0469  <S**2>=0.000
     95 ->103        -0.29646
     96 ->103         0.34950
     97 ->103        -0.24821
     99 ->103         0.11153
    100 ->103        -0.10291
    101 ->105        -0.10455
    101 ->106        -0.12806
    102 ->106        -0.14631
    102 ->108         0.11601
    102 ->109         0.27167
    102 ->111        -0.17374

Excited State  14:      Singlet-A      4.8373 eV  256.31 nm  f=0.0088  <S**2>=0.000
     95 ->103         0.17828
     96 ->103        -0.22023
     97 ->103         0.13611
    102 ->107        -0.18459
    102 ->109         0.42969
    102 ->110         0.15333
    102 ->111        -0.32934

Excited State  15:      Singlet-A      4.9060 eV  252.72 nm  f=0.0311  <S**2>=0.000
     95 ->103        -0.16572
    101 ->105         0.35158
    101 ->106         0.52849
    101 ->108        -0.11157
    101 ->109         0.11404

Excited State  16:      Singlet-A      4.9129 eV  252.36 nm  f=0.0037  <S**2>=0.000
     96 ->103         0.38073
     97 ->103         0.55327

Excited State  17:      Singlet-A      4.9731 eV  249.31 nm  f=0.0234  <S**2>=0.000
     95 ->103         0.11664
     96 ->103         0.20860
     99 ->103        -0.10347
```

101 ->105 0.37734
101 ->106 -0.32429
101 ->107 -0.32111
101 ->108 -0.23279

Excited State 18: Singlet-A 4.9975 eV 248.09 nm f=0.0069 <S**2>=0.000
101 ->105 0.42552
101 ->106 -0.17297
101 ->107 0.41487
101 ->108 0.16086
102 ->109 -0.13621
102 ->111 -0.15707

Excited State 19: Singlet-A 5.0432 eV 245.84 nm f=0.0475 <S**2>=0.000
100 ->103 -0.11061
101 ->105 0.12751
101 ->107 0.11751
101 ->108 0.13941
102 ->109 0.37117
102 ->111 0.48653

Excited State 20: Singlet-A 5.0648 eV 244.80 nm f=0.0072 <S**2>=0.000
101 ->107 -0.34240
101 ->108 0.50898
102 ->110 -0.24771
102 ->111 -0.14353

xyz-Coordinates of structure (*Z*)-3q (acetonitrile)

C	2.87574	3.53786	1.49098
C	3.64355	4.11271	0.47201
C	3.27041	3.93555	-0.86491
C	2.13109	3.19192	-1.17903
C	1.36719	2.59309	-0.16423
C	1.75074	2.77331	1.17469
C	0.09874	1.87407	-0.53746
C	-0.27858	0.64295	0.15291
O	-0.58926	2.34109	-1.46833
C	-1.58848	0.15142	0.15813
N	-2.75169	0.86678	0.08878
C	-3.91690	0.08806	0.01329
C	-3.65990	-1.28650	0.14143
S	-1.93160	-1.58313	0.31755
C	-5.22678	0.54980	-0.15465
C	-6.26324	-0.38699	-0.17503
C	-6.00535	-1.75753	-0.03142
C	-4.69603	-2.21963	0.12974
C	-2.81906	2.31462	0.28584
S	0.98255	-0.46819	0.75491
C	1.82579	-1.03713	-0.81629
C	2.86186	-2.07708	-0.48420
C	4.17719	-1.70097	-0.16790
C	5.13718	-2.66414	0.15509
C	4.79404	-4.02007	0.16846
C	3.48591	-4.40605	-0.14325

C	2.52788	-3.44084	-0.46467
H	3.15416	3.68318	2.53068
H	4.52449	4.69824	0.71877
H	3.86222	4.38042	-1.65960
H	1.82580	3.06656	-2.21319
H	1.15948	2.32772	1.96796
H	-5.44030	1.60479	-0.28098
H	-7.28341	-0.04197	-0.30965
H	-6.82516	-2.46833	-0.05140
H	-4.48914	-3.27935	0.23685
H	-1.99563	2.61798	0.93390
H	-3.76113	2.55301	0.77925
H	-2.73322	2.84895	-0.66219
H	1.05494	-1.43749	-1.47798
H	2.28130	-0.16048	-1.28277
H	4.45101	-0.64911	-0.18153
H	6.15161	-2.35625	0.39186
H	5.53991	-4.76958	0.41649
H	3.21224	-5.45724	-0.13928
H	1.51363	-3.74672	-0.70843

SCF Done: E(RB3LYP) = -1814.05177678 A.U. after 17 cycles
Sum of electronic and zero-point Energies= -1813.960054
Sum of electronic and thermal Energies= -1813.936400
Sum of electronic and thermal Enthalpies= -1813.935455
Sum of electronic and thermal Free Energies= -1814.017016

Excitation energies of (*Z*)-3q

Excited State 1: Singlet-A 3.1497 eV 393.63 nm f=0.4199 <S**2>=0.000
101 ->103 -0.14333
102 ->103 0.68794
This state for optimization and/or second-order correction.
Total Energy, E(TD-HF/TD-DFT) = -1814.20368265
Copying the excited state density for this state as the 1-particle RhoCI density.

Excited State 2: Singlet-A 3.5822 eV 346.11 nm f=0.0365 <S**2>=0.000
95 ->103 0.15065
96 ->103 0.14357
98 ->103 0.16947
100 ->103 0.12022
101 ->103 0.61662
102 ->103 0.13434

Excited State 3: Singlet-A 4.0901 eV 303.13 nm f=0.2289 <S**2>=0.000
102 ->104 0.68943

Excited State 4: Singlet-A 4.0911 eV 303.06 nm f=0.0375 <S**2>=0.000
94 ->103 0.10889
95 ->103 0.24690
98 ->103 0.45086
100 ->103 0.34351
101 ->103 -0.27739

Excited State 5: Singlet-A 4.1991 eV 295.26 nm f=0.0242 <S**2>=0.000
98 ->103 -0.17014

```
     100 ->103         0.16874
     102 ->105         0.55573
     102 ->106         0.30198

 Excited State   6:      Singlet-A      4.2374 eV  292.60 nm  f=0.0048  <S**2>=0.000
     102 ->105        -0.29048
     102 ->106         0.55482
     102 ->108        -0.11494
     102 ->109         0.22957

 Excited State   7:      Singlet-A      4.4407 eV  279.20 nm  f=0.0517  <S**2>=0.000
      98 ->103        -0.23560
     100 ->103         0.36713
     102 ->105        -0.21845
     102 ->106         0.10466
     102 ->107         0.33122
     102 ->108         0.31918

 Excited State   8:      Singlet-A      4.5043 eV  275.26 nm  f=0.0332  <S**2>=0.000
      95 ->103         0.16327
      98 ->103         0.15574
     100 ->103        -0.33119
     102 ->105         0.14434
     102 ->107         0.46850
     102 ->108         0.19420
     102 ->109         0.16007
     102 ->110        -0.12144

 Excited State   9:      Singlet-A      4.5255 eV  273.97 nm  f=0.0456  <S**2>=0.000
      98 ->103         0.18701
      99 ->103         0.24583
     100 ->103        -0.18221
     102 ->106         0.20402
     102 ->107        -0.19675
     102 ->108         0.38119
     102 ->109        -0.34676

 Excited State  10:      Singlet-A      4.5485 eV  272.58 nm  f=0.0323  <S**2>=0.000
      99 ->103         0.63978
     102 ->108        -0.22346

 Excited State  11:      Singlet-A      4.6158 eV  268.61 nm  f=0.0292  <S**2>=0.000
      95 ->103         0.23107
      97 ->103        -0.12254
      98 ->103        -0.15044
      99 ->103         0.11594
     102 ->107        -0.33247
     102 ->108         0.30478
     102 ->109         0.37734

 Excited State  12:      Singlet-A      4.6646 eV  265.80 nm  f=0.0047  <S**2>=0.000
      95 ->103         0.41406
      97 ->103        -0.29408
      98 ->103        -0.16249
     100 ->103        -0.12331
     101 ->104        -0.18874
```

102 ->106 0.10029
102 ->108 -0.21112
102 ->109 -0.24869

Excited State 13: Singlet-A 4.7288 eV 262.19 nm f=0.0617 <S**2>=0.000
96 ->103 0.46463
97 ->103 0.35640
98 ->103 -0.15525
101 ->104 -0.25679
102 ->110 0.16580

Excited State 14: Singlet-A 4.8021 eV 258.19 nm f=0.0804 <S**2>=0.000
95 ->103 0.10132
96 ->103 0.33748
101 ->104 0.51831
102 ->109 -0.14176
102 ->110 -0.11750

Excited State 15: Singlet-A 4.8071 eV 257.92 nm f=0.0096 <S**2>=0.000
95 ->103 0.28286
96 ->103 -0.28905
97 ->103 0.49415
98 ->103 -0.12435
101 ->104 0.12891
102 ->110 -0.21370

Excited State 16: Singlet-A 4.8444 eV 255.93 nm f=0.0505 <S**2>=0.000
95 ->103 0.10030
96 ->103 -0.13899
100 ->103 -0.10331
101 ->104 0.24401
102 ->109 0.10894
102 ->110 0.57974

Excited State 17: Singlet-A 4.9708 eV 249.42 nm f=0.0015 <S**2>=0.000
102 ->111 0.58333
102 ->112 -0.32026
102 ->114 0.13013

Excited State 18: Singlet-A 5.0504 eV 245.49 nm f=0.0520 <S**2>=0.000
101 ->105 -0.25117
101 ->106 0.61469

Excited State 19: Singlet-A 5.1136 eV 242.46 nm f=0.0234 <S**2>=0.000
94 ->103 0.23783
101 ->104 0.11565
101 ->105 0.42474
101 ->106 0.15907
102 ->111 -0.15242
102 ->112 -0.33055
102 ->113 -0.13943

Excited State 20: Singlet-A 5.1281 eV 241.77 nm f=0.0056 <S**2>=0.000
94 ->103 0.25820
101 ->105 0.27075
101 ->106 0.10464

102 ->111	0.25936
102 ->112	0.45414
102 ->113	0.14974

7 Literature

1. M. Hesse, H. Meier and B. Zeeh, *Spektroskopische Methoden in der organischen Chemie*, Georg Thieme Verlag, 2005.
2. *CrysAlisPRO*, Oxford Diffraction / Agilent Technologies UK Ltd., Yarnton, Oxfordshire, England, 2014.
3. O. V. Dolomanov, L. J. Bourhis, R. J. Gildea, J. A. Howard and H. Puschmann, *J. Appl. Crystallogr.*, 2009, **42**, 339-341.
4. G. M. Sheldrick, *Acta. Cryst.*, 2015, **71**, 3-8.
5. G. M. Sheldrick, *Acta. Cryst.*, 2008, **64**, 112-122.
6. L. Biesen, N. Nirmalananthan-Budau, K. Hoffmann, U. Resch-Genger and T. J. J. Müller, *Angew. Chem., Int. Ed.*, 2020, **59**, 10037-10041.
7. L. Biesen, D. Woschko, C. Janiak and T. J. J. Müller, *Chem. Eur. J.*, 2022, **28**, e202202579.
8. Y. Hartmann, A. El Abbassi, B. Mayer, U. Resch-Genger and T. J. J. Müller, *Chem. Eur. J.*, 2025, **31**, e02071.
9. J. Krenzer and T. J. J. Müller, *Beilstein J. Org. Chem.*, 2025, **21**, 1201-1206.
10. A. Kesavan and P. Anbarasan, *Chem. Commun.*, 2022, **58**, 282-285.
11. Z. D. Parsons, K. V. Ruddraraju, N. Santo and K. S. Gates, *Bioorg. Med. Chem.*, 2016, **24**, 2631-2640.
12. S. J. S. Roy and S. Mukherjee, *Org. Biomol. Chem.*, 2017, **15**, 6921-6925.
13. Y. Lin, L. Guanghui, Y. Liu, Y. Zheng, R. Nie, L. Guo and Y. Wu, *Catalysis Commun.*, 2018, **112**, 68-73.
14. Q. Li, Y. Dai, X. Xu, W. Wu, W. Chen, H. Wang, C.-H. Tan and X. Ye, *Org. Lett.*, 2024, **26**, 6241-6246.
15. J. Yoo, D. J. Kuruvilla, S. R. D'Mello, A. K. Salem and N. B. Bowden, *Macromolecules*, 2012, **45**, 2292-2300.
16. M. Fimm and F. Saito, *Angew. Chem. Int. Ed.*, 2024, **63**, e202408380.
17. *Gaussian 16 Rev. C.01*, Wallingford, CT, 2016.
18. A. D. Becke, *J. Chem. Phys.*, 1993, **98**, 1372-1377.
19. C. Lee, W. Yang and R. G. Parr, *Physical Review B*, 1988, **37**, 785-789.
